# Changes in IgA-targeted microbiota following fecal transplantation for recurrent *Clostridioides difficile* infection

**DOI:** 10.1080/19490976.2020.1862027

**Published:** 2020-12-31

**Authors:** Kelsey E Huus, Marcin Frankowski, Maja Pučić-Baković, Frano Vučković, Gordan Lauc, Benjamin H Mullish, Julian R Marchesi, Tanya M Monaghan, Dina Kao, B. Brett Finlay

**Affiliations:** a Michael Smith Laboratories and the Department of Microbiology and Immunology, University of British Columbia, Vancouver, British Columbia, Canada; b Faculty of Chemistry, Adam Mickiewicz University, Poznań, Poland; c Glycoscience Research Laboratory, Genos Ltd., Zagreb, Croatia; d Pharmacy and Biochemistry, University of Zagreb, Zagreb, Croatia; e Division of Digestive Diseases, Department of Metabolism, Digestion and Reproduction, Imperial College London, London, UK; f School of Biosciences, Cardiff University, Cardiff, UK; g NIHR Nottingham Biomedical Research Centre, University of Nottingham, Nottingham, UK; h Nottingham Digestive Diseases Centre, School of Medicine, University of Nottingham, Nottingham, UK; i Division of Gastroenterology,Department of Medicine, University of Alberta, Edmonton, Alberta, Canada

**Keywords:** Immunoglobulin A, microbiota, *Clostridioides difficile*, fecal microbiota transplant

## Abstract

Secretory immunoglobulin A (IgA) interacts with intestinal microbiota and promotes mucosal homeostasis. IgA-bacteria interactions are altered during inflammatory diseases, but how these interactions are shaped by bacterial, host, and environmental factors remains unclear. In this study, we utilized IgA-SEQ to profile IgA-bound fecal bacteria in 48 recurrent *Clostridioides difficile* patients before and after successful fecal microbiota transplantation (FMT) to gain further insight. Prior to FMT, *Escherichia coli* was the most highly IgA-targeted taxon; following restoration of the microbiota by FMT, highly IgA-targeted taxa included multiple *Firmicutes* species. Post-FMT IgA-targeting was unaffected by the route of FMT delivery (colonoscopy versus capsule), suggesting that both methods lead to the establishment of healthy immune–bacterial interactions in the gut. Interestingly, IgA-targeting in FMT recipients closely resembled the IgA-targeting patterns of the donors, and fecal donor identity was significantly associated with IgA-targeting of the recipient microbiota. These data support the concept that intrinsic bacterial properties drive IgA recognition across genetically distinct human hosts. Together, this study suggests that IgA-bacterial interactions are reestablished in human FMT recipients to resemble that of the healthy fecal donor.

## Introduction

The mucosal antibody immunoglobulin A (IgA) helps to maintain homeostasis of the intestinal microbiota. IgA excludes pathogens, promotes the adhesion of commensals, and alters microbial gene expression, shaping both the composition and the function of the microbiota.^1^ IgA-bacterial interactions are often highly strain-specific, differentiating between closely related amplicon sequence variants (ASVs).^2–4^ However, the factors which determine these IgA-bacterial interactions in the human gut remain poorly understood. IgA-targeting patterns are conserved between human feces and gnotobiotic mouse recipients, suggesting that intrinsic bacterial properties drive antibody recognition.^2,4,5^ Mouse genotype also influences IgA-microbiota recognition, however, through variation in major histocompatibility complex (MHC) binding and other immune factors.^6–8^ Further, environmental factors such as host nutrition have been recently shown to influence IgA-bacterial binding in mice.^3^ IgA-targeting of the human microbiota is altered during inflammatory diseases and undernutrition,^2,9–11^ but the extent to which these changes are driven by bacterial properties, host immunity, or environmental factors is unknown.

When intestinal homeostasis is disrupted due to repeated antibiotic exposure, the gut ecosystem becomes susceptible to *Clostridioides difficile*, a major cause of hospital-acquired and recurrent infections in North America and Europe.^12,13^ Fecal microbiota transplant (FMT) from healthy donors is the most effective treatment for recurrent *C. difficile* infection (rCDI), as it restores a diverse microbial community and associated colonization resistance.^14–16^ FMT has also allowed researchers to study the establishment of intestinal microbial communities in humans. Interestingly, the microbiota of FMT recipients is an emergent mixture of donor strains and preexisting patient strains, and its composition can be successfully predicted by machine learning techniques.^17,18^ The establishment of IgA–microbiota interactions in the human intestinal tract following FMT has never been studied.

Here we characterize the IgA-targeted microbiota before and after FMT from 48 of 116 patients who participated in a clinical trial and were randomized to either colonoscopy or oral capsule delivered FMT for rCDI.^14^ We evaluate the influence of delivery method, donor and pre-transplant microbiota, host anthropometrics, and systemic metabolites on IgA-bacterial targeting in FMT recipients. The dysbiotic microbiota of pre-FMT rCDI patients was characterized by IgA-bound *E. coli*, similar to observations in other inflammatory intestinal conditions. In contrast, post-FMT recipients exhibited a diverse community of IgA-bound bacteria that included multiple *Firmicutes*. IgA-targeting was not influenced by delivery method, indicating that both colonoscopy and oral capsule lead to the establishment of healthy immune–bacterial interactions in the fecal microbiota. Furthermore, IgA-targeting patterns in FMT recipients closely resemble that of their donors, suggesting that intrinsic bacterial properties determine IgA–microbiota interactions in the human intestine.

## Results

### IgA responses before and after FMT

Fecal samples from 48 randomly selected patients recruited from Edmonton and cured of rCDI were obtained before and after FMT.^14^ IgA-targeting of the microbiota was quantified by IgA-SEQ: briefly, fecal bacteria were sorted into IgA-positive (IgA+) and IgA-negative (IgA-) populations by flow cytometry, and subsequently characterized by 16S rDNA amplicon sequencing to identify IgA-bound bacteria.^3,10^ After filtering and rarefaction, a total of 36 patients (mean [SD] age, 57.9 [18.5] y; 22 women [61%]; 17 [47%] colonoscopy-delivered FMT; 1 patient had underlying ulcerative colitis and 1 had Crohn’s disease) had high-quality 16S data in both sorted fractions pre- and post-FMT. An IgA Index was calculated as previously described,^3,10^ representing the enrichment of bacteria in the IgA+ fraction compared to the IgA- fraction (see Methods for further details). For comparison with the IgA Index, we further estimated overall relative abundance of each taxon as its combined abundance in the IgA+ and the IgA- sorted fractions.

As expected, the total microbiota of rCDI patients pre-FMT exhibited patterns of antibiotics-driven dysbiosis, including very low alpha-diversity (Fig S1A), enrichment of *E. coli* (Fig S1B) and separation from healthy donors by principle coordinate analysis (PCoA) (Fig S1C). We did not detect *C. difficile* in the pre-FMT gut in this dataset, likely due to vancomycin suppression all patients received before transplantation. In FMT recipients, alpha diversity was increased to more closely resemble that of the fecal donors (Fig S1A), and there was a substantial shift in microbiota composition reflecting the restored abundances of multiple *Firmicutes* and *Bacteroidetes* members (Fig S1B-C). These data are consistent with previous characterizations of the total microbiota in rCDI patients before and after FMT.^14–16^

**Figure 1.**
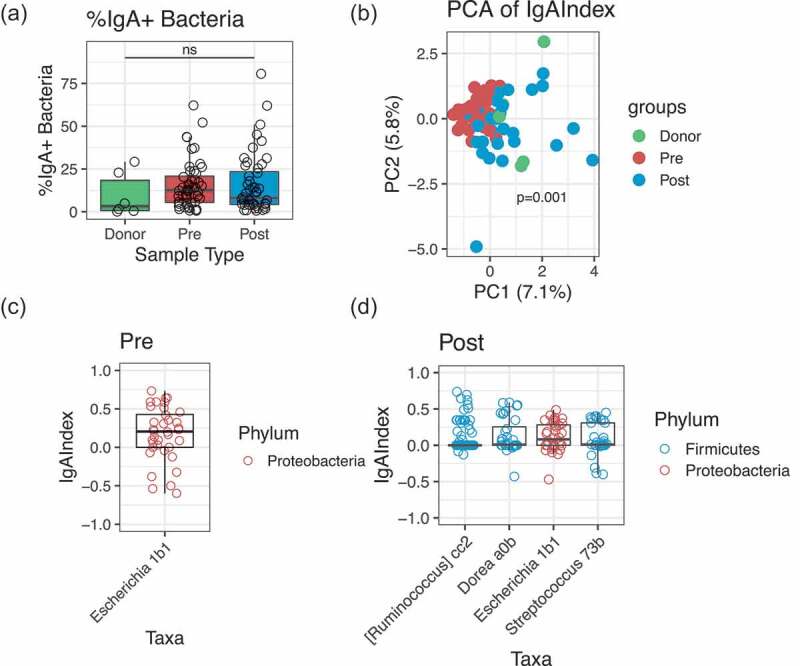
Differences in IgA-targeting of the microbiota before and after FMT. (a) Percentage of IgA-positive (%IgA+) fecal bacteria in 48 patients pre-FMT (Pre) and post-FMT (Post), and in 6 FMT donor samples from 4 individual donors. Significance determined by Kruskal-Wallis. (b) Principal Component Analysis based on Euclidean distance of the IgA Index. Significance determined by PERMANOVA. (c–d) Most highly IgA-targeted taxa pre-FMT (c) and post-FMT (d), as determined by an IgA Index significantly greater than zero by one-sided Wilcoxon rank sum test. Each data point in (c-d) represents an individual patient. In (b-d), *n* = 36 patients pre- and post-FMT, and n=5 donor fecal samples from 3 individual donors

The proportion of fecal bacteria bound by IgA was not different between donors and pre- or post-transplant patients (Figure 1(a)). However, broad changes in IgA-targeting were apparent before and after FMT, according to a principle component analysis (PCA) of the IgA Index (Figure 1(b), *p* = .001 by PERMANOVA). Prior to FMT, *E. coli* was the most highly IgA-targeted member of the fecal microbiota, as defined by an IgA Index significantly greater than zero (Figure 1(c)). Following FMT, several *Firmicutes* members were among the most IgA-targeted taxa, including *Ruminococcus* and *Dorea* (Figure 1(d)). *E. coli* is known to be highly IgA-targeted in the inflamed intestine,^9,10^ whereas *Firmicutes* species in general, and *Ruminococcus* and *Dorea* in particular, are common targets of IgA in healthy adults.^5,19^ Overall, these data suggest that healthy IgA–microbiota interactions are restored in rCDI patients following FMT.

Importantly, IgA-targeting reflects immune recognition of bacteria independently of their taxonomic abundance. IgA-targeting in FMT recipients was dependent on strain identity and varied between closely related ASV, including many abundant yet IgA-negative strains (Fig S2A-B). Indeed, consistent with previous reports, there was no correlation between taxonomic abundance and IgA-targeting in this dataset (Fig S2C-D). These data therefore indicate that in addition to total microbiota diversity, healthy IgA–microbiota interactions are restored in rCDI patients following FMT.

### IgA response by FMT delivery

**Figure 2.**
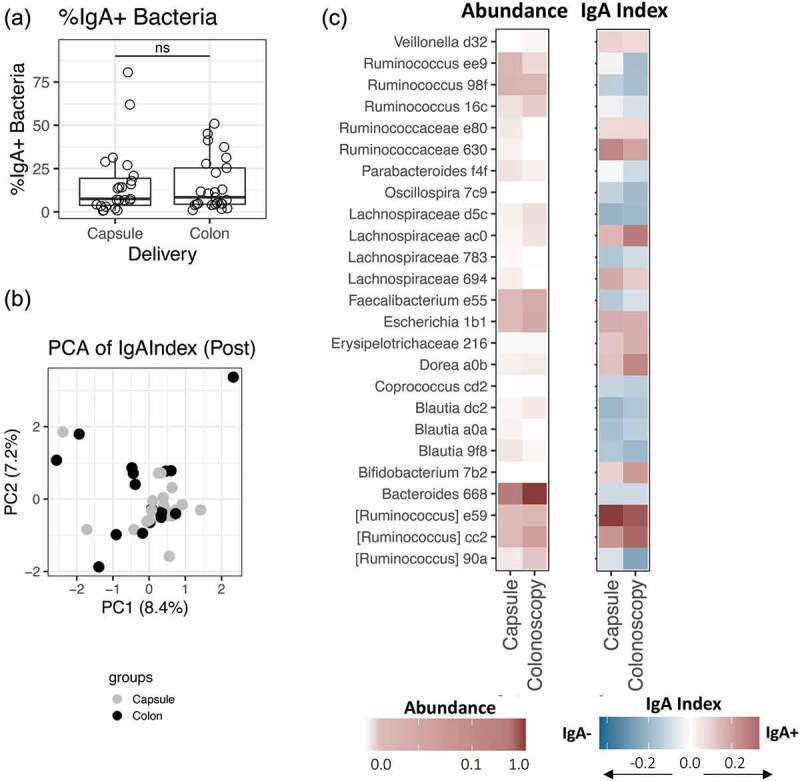
IgA-targeting of microbiota post-FMT by delivery method. (a) Percentage of IgA-positive (%IgA+) fecal bacteria in 48 FMT recipients (*n* = 23 capsule delivery and *n* = 25 colonoscopy delivery). Significance determined by Wilcoxon rank sum test. (b) Principal Component Analysis based on Euclidean distance of the IgA Index. Non-significant by PERMANOVA (*p* > .1). (c) Heatmap depicting average relative abundance and average IgA Index in post-transplant patients by delivery method. No taxa were significantly different between capsule and colonoscopy recipients, as determined by Wilcoxon rank sum test with FDR-corrected *p*-value < 0.05. Letters and numbers after the genus designate the first characters of a unique ASV code used by qiime2. For (b–c), *n* = 36 FMT recipients (*n* = 19 capsule delivery and *n* = 17 colonoscopy delivery)

FMT was delivered in this study via oral capsule or colonoscopy, which we previously showed did not have an impact on success of FMT or resultant composition of the microbiota.^14^ However, colonic and small intestinal IgA responses differ greatly; route of delivery could thus conceivably alter the initiation of an immune response. We confirm here that there were no differences in IgA-bacterial interactions by delivery method. Both capsule and colonoscopy recipients had comparable IgA-coating levels of the microbiota (Figure 2(a)). There was no difference in distribution of the IgA Index by delivery method according to PERMANOVA analysis (Figure 2(b); *p* > .05). Further, no ASV differed in abundance or IgA-targeting by Wilcoxon Rank Sum Test at FDR<0.1 (Figure 2(c)). These data suggest that the route of delivery does not influence fecal IgA-bacterial interactions post-FMT.

### IgA responses are influenced by fecal donor

**Figure 3.**
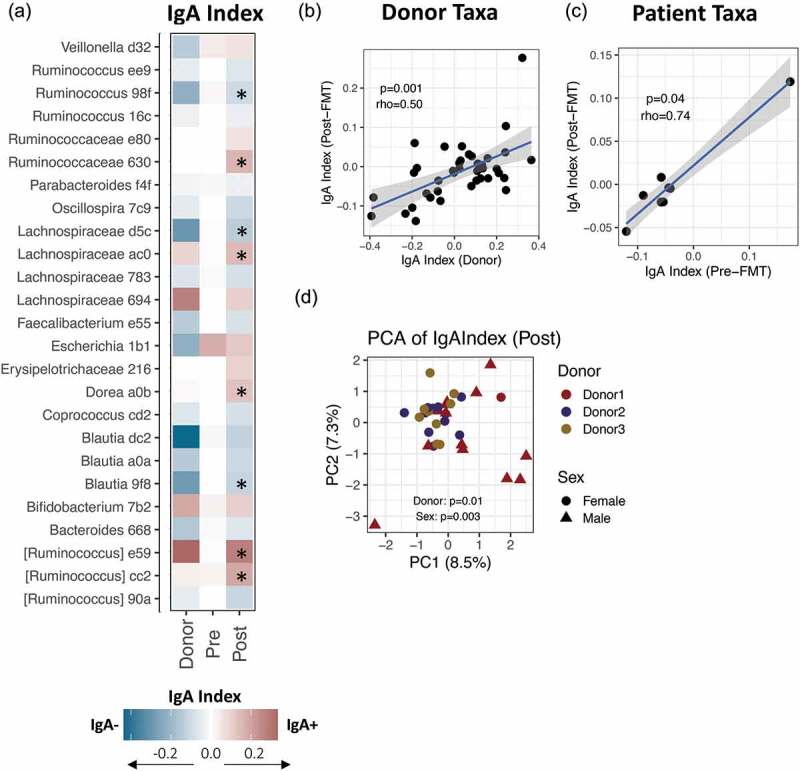
IgA-targeting of microbiota post-FMT is influenced by donor microbiota (a) Heatmap depicting average IgA-targeting of taxa in donors, pre- and post-transplant patients. A star (*) indicates taxa that were significantly different post-FMT compared to pre-FMT, as determined by paired Wilcoxon rank sum test with FDR-corrected *p*-value < 0.05. Letters and numbers after the genus designate the first characters of a unique ASV code used by qiime2. *N* = 36 patients pre- and post-FMT, and *n* = 5 donor fecal samples from 3 individual donors. (b) Correlation between IgA Index of donors and post-FMT patients, for taxa that were detected in donors but not in pre-FMT patients. Each data point represents the average IgA Index of a single ASV. Significance determined by Spearman’s correlation. (c) Correlation between IgA Index of pre-FMT and post-FMT patients, for taxa that were abundant in pre-FMT patients. Each data point represents the average IgA Index of a single ASV. Significance determined by Spearman’s correlation. (d) Principal Coordinate Analysis based on Euclidean distance of the IgA Index post-FMT, by donor identity and patient sex. Significance determined by PERMANOVA. *N* = 35 FMT recipients (*n* = 14 of donor 1; *n* = 12 of donor 2; *n* = 9 of donor 3). FMT recipients from a fourth donor were excluded as the sample size was too small for statistical comparison (*n* = 1)

Post-FMT microbiota samples clustered together with donor samples by PCA analysis of the IgA Index (Figure 1(b)). Indeed, the majority of taxa showed similar IgA-targeting status between donors and FMT recipients (Figure 3(a), Fig S3A). This association was most notable for taxa which engrafted from the donor microbiota, i.e., taxa which were undetected in the pre-FMT patient microbiota but were detected in the donor and the post-FMT microbiota. For such ‘donor taxa’, there was a strong correlation between donor IgA Index and post-FMT IgA Index (Figure 3(b)). For example, *Ruminococcus torques* was missing in pre-FMT patients, but was IgA-positive in the donors; both relative abundance and high IgA-recognition of this taxon were restored in patients post-FMT (Fig S3B-C).

In contrast, taxa that were detected in the dysbiotic rCDI microbiota pre-FMT showed poor correlation with donor IgA-targeting (Fig S3D). Instead, IgA-targeting of these ‘patient taxa’ after FMT correlated with their pre-FMT IgA-targeting (Figure 3(b)). For example, *E. coli* was highly IgA-targeted in pre-FMT patients, but was not bound by IgA in healthy fecal donors; rather than reverting to donor status, *E. coli* remained highly IgA-targeted in post-FMT patients (Figure 1(d), Fig S3E-F). The post-FMT microbiota is known to contain an emergent mixture of patient and donor strains; although strain-level resolution is not possible in this dataset, we speculate that abundant patient strains persisted post-FMT and thus retained their IgA-targeting status.

Given the overall similarity of the recipient and donor IgA-targeting patterns, we next examined whether IgA-targeting of the post-FMT microbiome was associated with IgA-targeting of the individual donor microbiome. Four different stool donors were used in this cohort; three donors had a large enough number of patient recipients for statistical analysis (n = 14, n = 12 and n = 9 FMT recipients with valid IgA Index data for donors 1, 2 and 3, respectively) and the fourth was excluded (n = 1 FMT recipient). As expected, the total microbiota was significantly different between recipients of the three different stool donors. This included significant changes in both alpha- and beta-diversity, and in the relative abundance of many strains, between recipients of different FMT donors (Fig S4A-C).

The proportion of IgA-bound bacteria did not differ significantly in FMT recipients by donor identity (Fig S4D). However, there was a significant separation of samples by donor identity according to principle coordinate analysis of the IgA Index (Figure 3(d); *p* = .01 by PERMANOVA analysis), indicating that overall IgA-targeting patterns also differ in the recipients of different fecal microbiotas. Several taxa showed significantly different IgA-targeting patterns in the recipients of the different fecal donors (Fig S4B). Only one of these, *Ruminococcus* ASV 16c, was differentially abundant in the same patients (Fig S4B). These data suggest that IgA-targeting patterns vary according to the donor microbiota composition, but not necessarily as a result of altered taxonomic abundance. Rather, strain-specific differences between ASV from different donors might explain the different IgA-targeting observed in recipients.

Importantly, since there was sex-matching between stool donor and recipient, testing for sex differences in post-FMT patients also yields similar differentially targeted taxa (Figure 3(d) and supplemental R markdown). We cannot, therefore, exclude the possibility that sex is a driver of IgA-targeting post-FMT, particularly as immune function is known to differ significantly between males and females.^20^ However, we did not see any differences in microbiota or IgA-targeting by sex prior to FMT (supplemental R markdown).

Collectively, we find that the IgA-targeting of the human microbiota after FMT is significantly influenced by IgA-targeting of the donor microbiota. This is independent of taxonomic abundance itself, and may reflect other properties of the engrafted strains. Together, these data support the importance of intrinsic microbial properties in determining IgA-targeting patterns of the human microbiota.

### Variability of IgA-targeted microbiota

**Figure 4.**
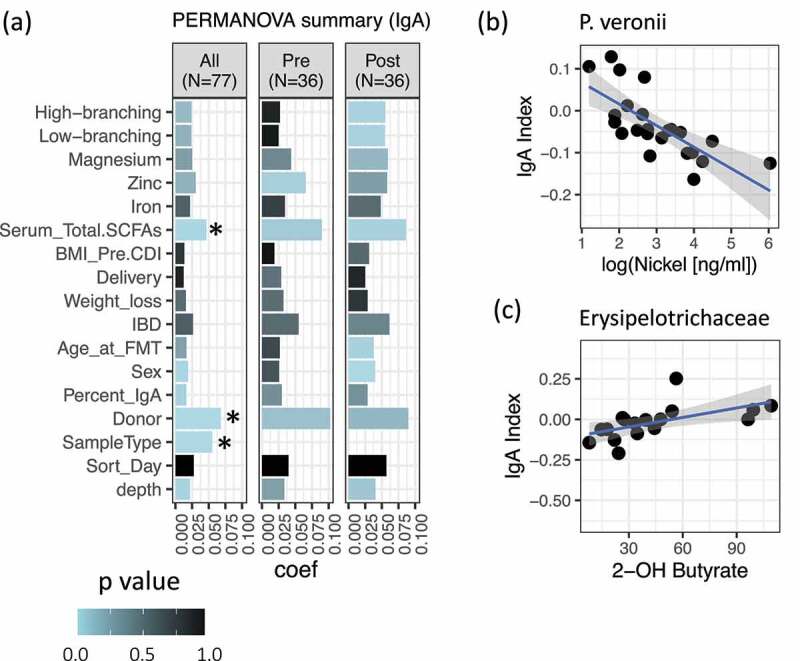
Variability of IgA-targeted microbiota. (a) PERMANOVA analysis, based on Euclidean distance of the IgA Index, was performed on the full dataset (*n* = 77, including donor samples), or on pre- and post-transplant patients (*n* = 36). A star (*) indicates significance at FDR-correction <0.05. Color represents raw *p* value, and ‘coef’ is the coefficient of variance. Permutations were constrained by day of sorting to account for a possible batch effect. (b) Correlation between IgA Index of *Pseudomonas veronii* and serum nickel concentration in post-FMT patients. Linear fit is shown. Significant by Spearman’s correlation at FDR-adjusted *p* < .05. *N*=23. (c) Correlation between IgA Index of *Erysipelotrichaceae* and serum 2-hydroxy butyrate in post-FMT patients. Linear fit is shown. Significant by Spearman’s correlation at raw *p* <. 05 but does not pass FDR. *N*=16

To identify other factors which might influence IgA-targeting in FMT recipients, we performed a PERMANOVA analysis of the IgA Index across multiple host and metabolic variables. Patient age, weight (body mass index (BMI) prior to FMT, and weight loss associated with rCDI) and underlying IBD did not measurably influence the IgA Index in this dataset (Figure 4(a)).

Restoration of the fecal microbiota in rCDI patients correlates with systemic metabolite changes, including increases in circulating short chain fatty acids (SCFA), decreases in complexity of serum *N*-glycan structures, and decreases in serum selenium and copper levels.^21–26^ Since SCFA, glycosylation, and nutrition are known to impact IgA responses to the microbiota, we also looked for the impact of these metabolites on IgA-targeting (Figure 4(a)). However, no metabolite reached statistical significance by PERMANOVA after FDR correction. We did not find the IgA-targeting of any individual taxon to correlate significantly with systemic metabolites, with the exception of a negative correlation between *Pseudomonas veronii* and nickel concentrations in post-FMT patients (Figure 4(b)). There were also several positive correlations between SCFA levels and the IgA-targeting of *Firmicutes* species post-FMT, which is interesting given that SCFA are known to stimulate IgA responses;^27^ however, these correlations did not pass FDR correction (Figure 4(c) and Supplemental R Markdown).

The correlations between IgA-targeting and specific metabolites observed here are interesting and warrant further study. However, the small sample size for analysis means that these associations should be interpreted with caution. Taken together, these data do not support a strong role for host or metabolic factors in determining microbiota IgA-targeting. We conclude that microbiota composition itself is a major determinant of IgA-targeting across genetically distinct human hosts.

## Discussion

This is the first study to examine the changing patterns of IgA-targeted microbiota in rCDI patients before and after FMT. These findings confirm that in addition to broad changes in the total microbiota, IgA-bacterial interactions are profoundly altered in rCDI patients. IgA-targeting of the microbiota is largely restored by FMT to resemble that of the healthy fecal donor. Mode of delivery does not influence IgA-bacterial targeting in FMT recipients, consistent with the clinical efficacy of both methods; this provides further support of the use of either colonoscopy or capsule as a viable FMT delivery method.

Previous studies of IgA-targeting in human populations have noted high inter-individual variability.^5,11^ It has been unclear whether this is driven by differences in microbiota composition, host genetics, diet, or a combination of factors. Here we show that IgA-bacterial binding patterns in FMT recipients closely resemble that of the donors and are influenced by donor identity. This is consistent with studies showing conserved IgA-binding patterns between fecal microbiota donors and gnotobiotic mouse recipients.^5^ Together, these data support the concept that specific bacterial properties shape IgA recognition of the human microbiota.

Notably, taxa that were abundant in patients prior to FMT retained their IgA-targeting status post-FMT. The microbiota of pre-FMT rCDI patients was characterized by highly IgA-targeted *E. coli*, and *E. coli* remained highly IgA-targeted after FMT despite low recognition of this taxon in healthy donor samples (Figure 3(a)). High IgA-targeting of *E. coli* has been observed in inflammatory bowel disease patients and in children with severe acute undernutrition, and correlates with the presence of *E. coli* virulence factors; moreover, IgA-targeted *E. coli* strains have been shown to induce inflammation and intestinal damage when transplanted into gnotobiotic mice.^2,9,10^ Thus, the high IgA-targeting of *E. coli* observed here suggests expansion of pro-inflammatory pathobionts in the rCDI patients, which may not be fully cleared by FMT. Alternatively, IgA recognition of *E. coli* may reflect a heightened host immune response to *E. coli* in rCDI patients, which persists post-FMT despite engraftment of the donor microbiota. In the future, resolving strain-level taxonomic differences through deep metagenomic sequencing would help to differentiate donor and patient strains and thus provide improved understanding of IgA-targeting variation by bacterial identity. Given the importance of IgA in maintaining intestinal homeostasis, a better understanding of these interactions and their recovery after FMT could help improve the rational design of microbial therapies for rCDI infection.^28^

This study has several limitations. The study cohort consisted of a small sample size from a single center. Additionally, the donors were all drawn from a healthy Western population and often showed similar IgA-coating patterns; in contrast, important differences in both strain identity and IgA-targeting may exist between geographically distinct populations and by disease state.^11^ Variation in IgA responses was poorly explained by available host and metabolite data, but the small sample size may have prevented the detection of more subtle influences on IgA-targeting. Also, although there was a significant effect of patient sex, this variable was inseparable from donor identity due to sex-matching of the patient and donor pairs. In the future, controlling for sex and accounting for host genetic and immune variation may be necessary to understand the role of the host in IgA-targeting of the microbiota.

IgA is fundamentally important in maintaining homeostasis of the intestinal microbiota, but the variation of IgA-targeting in health and disease remains poorly understood. This study shows that dysbiotic IgA-targeting of the microbiota in rCDI patients is largely, but not completely, restored by FMT. Furthermore, bacterial identity is a major determinant of IgA-targeting across unrelated human hosts. Together, these data provide valuable insights into immune recognition of the rCDI microbiota before and after FMT, and into our fundamental understanding of IgA-microbial homeostasis.

## Materials and methods

### Study participants and sample collection

Adult patients with at least 2 CDI recurrences were recruited in Alberta between 2014 and 2016 as previously described.^14^ Each patient was maintained on vancomycin suppression till 24 hours prior to the assigned FMT treatment. Stool samples were collected at screening (pre-FMT), and at 4 weeks after FMT (post-FMT), and were stored at −80 C as previously reported. Ethical approval for this study was obtained by the Health Research Ethics Board, University of Alberta (Pro00049006) and the Clinical Research Ethics Board, University of British Columbia (H18-00880), and all patients provided informed consent.

### IgA-sorting

IgA-sequencing was performed as described previously.^3^ Approximately 50 mg of each fecal sample (± 10 mg) was homogenized in 1 mL of phosphate-buffered saline (PBS; HyClone DPBS-/-, SH30028.02) and spun gently to settle debris; intestinal bacteria were then filtered through a 0.7 µm filter. A volume of suspension equal to 5 mg of sample was washed in FACS buffer (PBS containing 1% bovine serum albumin) and blocked for 20 minutes in FACS buffer containing 10% fetal bovine serum. Samples were then stained with anti-human IgA-PE (Miltenyl 130–093-128) or an isotype control (eBioscience, 12–4714-42) at 1:25 dilution for 30 minutes in the dark. Samples were washed twice more and fixed overnight in 2% paraformaldehyde (PFA) at 4°C in the dark without shaking. The next day, the PFA was washed off and samples were stained with SYTO-BC (1:4000 dilution) for bacterial DNA, washed again and sorted by flow cytometry into IgA-positive (FITC+PE+) and IgA-negative (FITC+PE-) populations. A minimum of 50 000 events are collected in the IgA+ and IgA- fraction and frozen at −20°C for further analysis. Each sample was stained with both an anti-human IgA antibody and an isotype control, and the final percentage of IgA-positive bacteria was reported after subtraction of the isotype-positive population. Samples in which the isotype and antibody-specific populations could not be distinguished were excluded from further sequencing analysis.

### Bioinformatics analysis of 16S rDNA data

Demultiplexed forward reads were analyzed in QIIME2 (https://qiime2.org/)^29^, using the Dada2 option^30^ for sequence quality control and trimming to 250 bp. Taxonomic assignment was performed using the SILVA database.^31^ Further filtering was then performed in R using phyloseq.^32^ Filtering included removal of unintended targets (archaea, mitochondria, and chloroplast) as well as common contaminants of Ig-SEQ datasets (*Alphaproteobacteria*),^11,33^ removal of singleton taxa, and rarefaction to 5000 reads.

A log-adjusted IgA index was calculated as described previously:^3,10^ $IgA_{Index}=-\left(\frac{\log\left(IgA^{+}taxon\right)-\log\left(IgA^{-}taxon\right)}{\log\left(IgA^{+}taxon\right)+\log\left(IgA^{-}taxon\right)}\right)$. A pseudocount of 0.0000001 relative abundance was added before log adjustment to allow for zero values. Positive IgA Index values (up to a maximum of 1.0) represent enrichment of a taxon in the IgA-positive-sorted fraction compared to the IgA-negative fraction, i.e., IgA-targeting of the bacterium. Conversely, negative values (down to a minimum of −1.0) indicate depletion in the IgA-positive fraction, or lack of IgA-targeting.

Taxa were maintained at the ASV level for calculation of the IgA Index. IgA Index data was further filtered for prevalence within each pre-FMT and post-FMT dataset, by excluding taxa in which ≥75% of samples had zero values. Relative abundance was estimated by summing the IgA-positive and IgA-negative relative abundance. We did not find any evidence of a sorting batch effect on the IgA Index (Supplemental R Markdown). Raw sequencing data has been deposited to the Sequence Read Archive (SRA) under BioProject PRJNA650203.

### 16S rDNA library preparation

Sorted bacterial suspensions were boiled for 15 min at 100°C and 2 µL of lysate was used as template for 16S PCR, using Illumina-tagged and barcoded primers specific for the 16S V4 region.^34^ PCR was performed with Phusion polymerase under the following cycling conditions: 5 minutes initial denaturation at 98°C, 30 cycles of 20 seconds at 98°C, 15 seconds at 55°C, 30 seconds at 72°C, and 10 minutes final extension at 72°C. Reactions were run on a gel to ensure successful amplification, and were purified and normalized using the 96well Sequel-Prep kit (ThermoFisher A1051001). All reactions were subsequently pooled and gel extracted (GeneJet K0692) to remover primer-dimers. Sequencing was performed on an Illumina MiSeq using a v2 kit for 2 × 250 bp reads with 30% PhiX at the Biomedical Research Center (BRC) Sequencing Core of the University of British Columbia.

### Detection and quantification of short-chain fatty acids (SCFAs) in feces and serum

Targeted gas chromatography-mass spectrometry (GC-MS) for SCFA detection, identification, and quantification was performed using adaptation of previously described protocols for the analysis of samples of stool and serum.^25,35,36^ Samples analysis was performed on an Agilent 7890B GC system coupled to an Agilent 5977A mass selective detector (Agilent, USA). Analysis of data was performed using MassHunter software (Agilent), with SCFA concentrations being integrated from a freshly prepared calibration curve for each standard.

### Biometal profiling

Selected trace metal (iron, selenium, zinc, cadmium, cobalt, copper, magnesium, manganese, nickel, and lead) concentrations in plasma were determined by Inductively Coupled Plasma Mass Spectrometry (ICP-MS) analytical technique, using the ICPMS-2030 spectrometer (Shimadzu, Japan). Details regarding ICP-MS measurement conditions and parameters are described in previous studies.^37^ Briefly, 100 µL serum was digested in a combination of 300 µL nitric acid (70% purified by redistillation, Sigma-Aldrich), 100 µL hydrogen peroxide solution (25–35% for ultratrace analysis, Sigma Aldrich), and 100 µL hydrochloric acid (30% suprapure, Merck). Serial dilutions of ICP-Multi-element standard solution IV (Merck, Certipur) and ICP-MS selenium single standard solution (Sigma-Aldrich, TraceCERT) were used for calibration of all the biometals analytes. A certified reference material, BCR 637 (Institute for Reference Materials and Measurements) as well as reference material ERM-DA120 (European Reference Materials) were analyzed to validate the calibration. A solution containing scandium, yttrium, terbium, tellurium, and rhodium (Sigma-Aldrich, TraceCERT) in 1% nitric acid was used as an internal standard (automatically added during analysis through T-piece).

### Serum N-glycome analysis

Analysis of total serum *N*-glycome was performed as previously described.^23^ Briefly, serum N-glycans were enzymatically released from proteins by PNGase F, fluorescently labeled with 2-aminobenzamide, and cleaned-up from the excess of reagents by hydrophilic interaction liquid chromatography solid phase extraction (HILIC-SPE), as previously described. Fluorescently labeled and purified N-glycans were separated by HILIC on a Waters BEH Glycan chromatography column, 150 × 2.1 mm i.d., 1.7 μm BEH particles, installed on an Acquity ultra-performance liquid chromatography (UPLC) H-class system (Waters), consisting of a quaternary solvent manager, sample manager and a fluorescence detector set with excitation and emission wavelengths of 250 nm and 428 nm, respectively. Obtained chromatograms were separated into 39 peaks. The amount of N-glycans in each chromatographic peak was expressed as a percentage of total-integrated area. From 39 directly measured glycan peaks we calculated 12 derived traits which average particular glycosylation traits like galactosylation, sialylation, and branching across different individual glycan structures and are, consequently, more closely related to individual enzymatic activities and underlying genetic polymorphisms. Derived traits used were: the proportion of low branching (LB) and high branching (HB) N-glycans, the proportion of a-, mono-, di-, tri- and tetra-galactosylated N-glycans (G0, G1, G2, G3 and G4, respectively), and a-, mono-, di-, tri- and tetra-sialylated N-glycans (S0, S1, S2, S3 and S4, respectively).

### Statistical analysis

All statistical analysis was performed in R studio using the phyloseq and ggplot2 packages as indicated in the attached R markdown file (Supplemental File 1). Data structure of the IgA Index was explored using a Principle Component Analysis based on Euclidean distance. A PERMANOVA analysis was iteratively applied to each variable of interest to determine its contribution to the distribution of the IgA Index. Non-parametric Wilcoxon rank sum tests were used to compare data with two groups, and data were paired by patient for pre-FMT versus post-FMT comparisons. A one-sided Wilcoxon rank sum test was used to identify taxa with an IgA Index significantly different from zero. Spearman’s correlation was applied for continuous variables, including serum metabolites. Unless otherwise indicated, multiple correction of statistical tests was applied using the False Discovery Rate (FDR).

## Supplementary Material

Supplemental Material
