# Supplementary material for: Changes in IgA-targeted microbiota following fecal transplantation for recurrent Clostridioides difficile infection
Source: Gut Microbes. 2020 Dec 31;13(1):1862027. doi: 10.1080/19490976.2020.1862027 (PMC7781654; doi:10.1080/19490976.2020.1862027)
Supplement: Supplemental Material [file KGMI_A_1862027_SM4814.zip › supplementary/FileS1_SupplementalRCode_revised.html]

Huus2020\_CdiffIgA\_Supplement\_revised11v2


# Huus2020\_CdiffIgA\_Supplement\_revised11v2

# Set Up

```
library(phyloseq)
library(naniar)
library(plyr)
library(dplyr)
library(tidyr)
library(vegan)
library(ggplot2)
library(ggbiplot)
library(gplots)
library(FSA)
library(psych)
library(reshape2)
library(gvlma)
library(boot)
```

# Percent IgA Analysis

Absolute percent IgA values

```
#import metadata
metaIgA1 <- read.table("FilesForR/cdiff-sample-metadata-FULL-JUNE2020.txt", header=TRUE, sep="\t")
metaIgA1 <- filter(metaIgA1, Percent_IgA!="NA")
metaIgA1 <- filter(metaIgA1, !duplicated(Stool_no))
dim(metaIgA1)
```

```
## [1] 106 149
```

```
#a little clean up of metadata names / order
metaIgA1$SampleType <- factor(metaIgA1$SampleType, 
                             levels=c("Donor", "PreTransplant", "PostTransplant"))
metaIgA1$SampleType2 <- metaIgA1$SampleType
metaIgA1$SampleType2 <- gsub("PreTransplant", "Pre", metaIgA1$SampleType2)
metaIgA1$SampleType2 <- gsub("PostTransplant", "Post", metaIgA1$SampleType2)
metaIgA1$SampleType2 <- factor(metaIgA1$SampleType2, 
                              levels=c("Donor", "Pre", "Post"))
metaIgA1Pre <- filter(metaIgA1, SampleType=="PreTransplant")
metaIgA1Post <- filter(metaIgA1, SampleType=="PostTransplant")

#pair the data
metaIgA1Pre <- metaIgA1Pre[which(metaIgA1Pre$Patient%in%metaIgA1Post$Patient),]
metaIgA1Post <- metaIgA1Post[which(metaIgA1Post$Patient%in%metaIgA1Pre$Patient),]

metaIgA1Pre <- metaIgA1Pre[order(metaIgA1Pre$Patient),]
metaIgA1Post <- metaIgA1Post[order(metaIgA1Post$Patient),]

metaIgA1Pre$Patient == metaIgA1Post$Patient #samples are paired
```

```
##  [1] TRUE TRUE TRUE TRUE TRUE TRUE TRUE TRUE TRUE TRUE TRUE TRUE TRUE TRUE TRUE
## [16] TRUE TRUE TRUE TRUE TRUE TRUE TRUE TRUE TRUE TRUE TRUE TRUE TRUE TRUE TRUE
## [31] TRUE TRUE TRUE TRUE TRUE TRUE TRUE TRUE TRUE TRUE TRUE TRUE TRUE TRUE TRUE
## [46] TRUE TRUE TRUE
```

```
metaIgA2 <- metaIgA1[(metaIgA1$SampleType!="Donor"&metaIgA1$Patient %in% metaIgA1Pre$Patient)|(metaIgA1$SampleType=="Donor"),]

##Differences by sample type
#sample sizes by sample type
dplyr::count(metaIgA2, SampleType)
```

```
##       SampleType  n
## 1          Donor  6
## 2  PreTransplant 48
## 3 PostTransplant 48
```

```
#sample type - including donor
kruskal.test(metaIgA2$Percent_IgA~metaIgA2$SampleType)
```

```
## 
##  Kruskal-Wallis rank sum test
## 
## data:  metaIgA2$Percent_IgA by metaIgA2$SampleType
## Kruskal-Wallis chi-squared = 2.3027, df = 2, p-value = 0.3162
```

```
#sample type - not including donor
metaIgA2_nodonor <- filter(metaIgA2, SampleType %in% c("PreTransplant", "PostTransplant"))
wilcox.test(metaIgA2_nodonor$Percent_IgA~metaIgA2_nodonor$SampleType)
```

```
## 
##  Wilcoxon rank sum exact test
## 
## data:  metaIgA2_nodonor$Percent_IgA by metaIgA2_nodonor$SampleType
## W = 1247, p-value = 0.4904
## alternative hypothesis: true location shift is not equal to 0
```

```
#plot boxplot: %IgA by Sample Type
p <- ggplot(metaIgA2) + geom_boxplot(aes(x=SampleType2, y=Percent_IgA, fill=SampleType),
                                    outlier.shape=NA, outlier.size=NA)
p <- p + geom_jitter(aes(x=SampleType2, y=Percent_IgA),
                     width=0.2, height=0, shape=1, size=3)
p <- p + ggtitle("%IgA+ Bacteria")
p <- p + theme_bw(base_size=15)
p <- p + ylab("%IgA+ Bacteria") + xlab("Sample Type")
p <- p + scale_fill_manual(values=c("seagreen3", "indianred3", "deepskyblue3"))
p <- p + guides(colour=FALSE, size=FALSE, shape=FALSE, fill=FALSE)
p <- p + annotate("text", x = 2, y=95, label = "ns", size=4)
p <- p + annotate("segment", x=1, xend=3, y=90, yend=90)
p
```

```
#plot boxplot: %IgA by Sample Type - no donor, paired
p <- ggplot(metaIgA2_nodonor) + geom_boxplot(aes(x=SampleType2, y=Percent_IgA, fill=SampleType2),
                                    outlier.shape=NA, outlier.size=NA)
p <- p + geom_point(aes(x=SampleType2, y=Percent_IgA),
                     shape=1, size=3)
p <- p + geom_line(aes(x=SampleType2, y=Percent_IgA, group=Patient))
p <- p + ggtitle("%IgA+ Bacteria")
p <- p + theme_bw(base_size=15)
p <- p + ylab("%IgA+ Bacteria") + xlab("Sample Type")
p <- p + scale_fill_manual(values=c("indianred3", "deepskyblue3"))
p <- p + guides(colour=FALSE, size=FALSE, shape=FALSE, fill=FALSE)
#p <- p + annotate("text", x = 2, y=95, label = "ns", size=4)
#p <- p + annotate("segment", x=1, xend=3, y=90, yend=90)
p
```

```
#sample sizes by donor recipient
dplyr::count(metaIgA1Post, Donor.ID)
```

```
##   Donor.ID  n
## 1        2 15
## 2        7 17
## 3       20 14
## 4       21  2
```

```
#post-transplant by donor recipient
a1 <- kruskal.test(metaIgA1Post$Percent_IgA ~ as.factor(metaIgA1Post$Donor.ID))
a1
```

```
## 
##  Kruskal-Wallis rank sum test
## 
## data:  metaIgA1Post$Percent_IgA by as.factor(metaIgA1Post$Donor.ID)
## Kruskal-Wallis chi-squared = 2.7598, df = 3, p-value = 0.4302
```

```
posthoc <- dunnTest(Percent_IgA ~ as.factor(Donor.ID),
              data=metaIgA1Post,
              method="bh") 
posthoc
```

```
## Dunn (1964) Kruskal-Wallis multiple comparison
```

```
##   p-values adjusted with the Benjamini-Hochberg method.
```

```
##   Comparison          Z   P.unadj     P.adj
## 1     2 - 20  0.4851088 0.6275991 0.7531190
## 2     2 - 21  0.4902511 0.6239562 0.9359343
## 3    20 - 21  0.2497265 0.8027988 0.8027988
## 4      2 - 7 -1.0160849 0.3095889 0.9287668
## 5     20 - 7 -1.4968392 0.1344351 0.8066106
## 6     21 - 7 -0.9751809 0.3294705 0.6589411
```

```
#post-transplant excluding donor 21 because there are only 2 recipients
metaIgA1Post_filt <- metaIgA1Post[metaIgA1Post$Donor!=21,]
a2 <- kruskal.test(metaIgA1Post_filt$Percent_IgA ~ as.factor(metaIgA1Post_filt$Donor.ID))
a2
```

```
## 
##  Kruskal-Wallis rank sum test
## 
## data:  metaIgA1Post_filt$Percent_IgA by as.factor(metaIgA1Post_filt$Donor.ID)
## Kruskal-Wallis chi-squared = 2.3474, df = 2, p-value = 0.3092
```

```
posthoc2 <- dunnTest(Percent_IgA ~ as.factor(Donor.ID),
              data=metaIgA1Post_filt,
              method="bh") 
posthoc2
```

```
## Dunn (1964) Kruskal-Wallis multiple comparison
##   p-values adjusted with the Benjamini-Hochberg method.
```

```
##   Comparison          Z   P.unadj     P.adj
## 1     2 - 20  0.4391491 0.6605535 0.6605535
## 2      2 - 7 -1.0432976 0.2968105 0.4452157
## 3     20 - 7 -1.4762265 0.1398831 0.4196493
```

```
#recode a simpler donor ID (1,2,3)
metaIgA1Post_filt$Donor.ID3 <- metaIgA1Post_filt$Donor.ID
metaIgA1Post_filt$Donor.ID3 <- gsub(20, 3, metaIgA1Post_filt$Donor.ID3)
metaIgA1Post_filt$Donor.ID3 <- gsub(2, 1, metaIgA1Post_filt$Donor.ID3)
metaIgA1Post_filt$Donor.ID3 <- gsub(7, 2, metaIgA1Post_filt$Donor.ID3)


#plot boxplot: %IgA vs Donor
p <- ggplot(metaIgA1Post_filt) + geom_boxplot(aes(x=as.factor(Donor.ID3), y=Percent_IgA))
p <- p + geom_jitter(aes(x=as.factor(Donor.ID3), y=Percent_IgA),
                     width=0.2, height=0, size=3)
p <- p + ggtitle("%IgA+ Bacteria (Post)")
p <- p + theme_bw(base_size=15)
p <- p + theme(title=element_text(size=14))
p <- p + ylab("%IgA+ Bacteria") + xlab("Donor ID")
#p <- p + scale_fill_manual(values=c("lightskyblue1", "deepskyblue", "dodgerblue3", "plum4"))
#p <- p + guides(colour=FALSE, size=FALSE, shape=FALSE, fill=FALSE)
p <- p + annotate("text", x = 2, y=95, label = "ns", size=4)
p <- p + annotate("segment", x=1, xend=3, y=90, yend=90)
p
```

```
#does the same pattern show up pre-transplant?
#pre-transplant excluding donor 21 because there are only 2 recipients
metaIgA1Pre_filt <- metaIgA1Pre[metaIgA1Pre$Donor!=21,]
a2 <- kruskal.test(metaIgA1Pre_filt$Percent_IgA ~ as.factor(metaIgA1Pre_filt$Donor.ID))
a2
```

```
## 
##  Kruskal-Wallis rank sum test
## 
## data:  metaIgA1Pre_filt$Percent_IgA by as.factor(metaIgA1Pre_filt$Donor.ID)
## Kruskal-Wallis chi-squared = 0.83154, df = 2, p-value = 0.6598
```

```
posthoc2 <- dunnTest(Percent_IgA ~ as.factor(Donor.ID),
              data=metaIgA1Post_filt,
              method="bh") 
posthoc2
```

```
## Dunn (1964) Kruskal-Wallis multiple comparison
##   p-values adjusted with the Benjamini-Hochberg method.
```

```
##   Comparison          Z   P.unadj     P.adj
## 1     2 - 20  0.4391491 0.6605535 0.6605535
## 2      2 - 7 -1.0432976 0.2968105 0.4452157
## 3     20 - 7 -1.4762265 0.1398831 0.4196493
```

```
#recode a simpler donor ID (1,2,3)
metaIgA1Pre_filt$Donor.ID3 <- metaIgA1Pre_filt$Donor.ID
metaIgA1Pre_filt$Donor.ID3 <- gsub(20, 3, metaIgA1Pre_filt$Donor.ID3)
metaIgA1Pre_filt$Donor.ID3 <- gsub(2, 1, metaIgA1Pre_filt$Donor.ID3)
metaIgA1Pre_filt$Donor.ID3 <- gsub(7, 2, metaIgA1Pre_filt$Donor.ID3)

p <- ggplot(metaIgA1Pre_filt) + geom_boxplot(aes(x=as.factor(Donor.ID3), y=Percent_IgA, fill=as.factor(Donor.ID3)),
                                       outlier.size=NA, outlier.shape=NA)
p <- p + geom_jitter(aes(x=as.factor(Donor.ID3), y=Percent_IgA),
                     width=0.2, height=0, shape=1, size=3)
p <- p + ggtitle("%IgA+ Bacteria (Pre)")
p <- p + theme_bw(base_size=15)
p <- p + ylab("%IgA+ Bacteria") + xlab("Donor ID")
p <- p + scale_fill_manual(values=c("lightskyblue1", "deepskyblue", "dodgerblue3", "plum4"))
p <- p + guides(colour=FALSE, size=FALSE, shape=FALSE, fill=FALSE)
p <- p + annotate("text", x = 2.5, y=95, label = "ns", size=4)
p <- p + annotate("segment", x=1, xend=3, y=90, yend=90)
p
```

```
#post transplant by delivery (colon vs capsule)
dplyr::count(metaIgA1Post, Delivery)
```

```
##   Delivery  n
## 1  Capsule 23
## 2    Colon 25
```

```
wilcox.test(metaIgA1Post$Percent_IgA ~ metaIgA1Post$Delivery)
```

```
## 
##  Wilcoxon rank sum exact test
## 
## data:  metaIgA1Post$Percent_IgA by metaIgA1Post$Delivery
## W = 282, p-value = 0.9186
## alternative hypothesis: true location shift is not equal to 0
```

```
p <- ggplot(metaIgA1Post) + geom_boxplot(aes(x=Delivery, y=Percent_IgA),
                                         outlier.size=NA, outlier.shape=NA)
p <- p + geom_jitter(aes(x=Delivery, y=Percent_IgA),
                     width=0.2, height=0, shape=1, size=3)
p <- p + ggtitle("%IgA+ Bacteria")
p <- p + theme_bw(base_size=15)
p <- p + ylab("%IgA+ Bacteria") + xlab("Delivery")
#p <- p + scale_fill_manual(values=c("lightskyblue1", "deepskyblue", "dodgerblue3", "plum4"))
p <- p + guides(colour=FALSE, size=FALSE, shape=FALSE, fill=FALSE)
p <- p + annotate("text", x = 1.5, y=95, label = "ns", size=4)
p <- p + annotate("segment", x=1, xend=2, y=90, yend=90)
p
```

Batch effect and relative percent IgA values

```
kruskal.test(metaIgA2$Percent_IgA, metaIgA2$Sort_Day)
```

```
## 
##  Kruskal-Wallis rank sum test
## 
## data:  metaIgA2$Percent_IgA and metaIgA2$Sort_Day
## Kruskal-Wallis chi-squared = 38.239, df = 2, p-value = 4.971e-09
```

```
kruskal.test(metaIgA1Pre$Percent_IgA, metaIgA1Pre$Sort_Day)
```

```
## 
##  Kruskal-Wallis rank sum test
## 
## data:  metaIgA1Pre$Percent_IgA and metaIgA1Pre$Sort_Day
## Kruskal-Wallis chi-squared = 15.112, df = 2, p-value = 0.0005229
```

```
kruskal.test(metaIgA1Post$Percent_IgA, metaIgA1Post$Sort_Day)
```

```
## 
##  Kruskal-Wallis rank sum test
## 
## data:  metaIgA1Post$Percent_IgA and metaIgA1Post$Sort_Day
## Kruskal-Wallis chi-squared = 17.993, df = 2, p-value = 0.0001238
```

```
dplyr::count(metaIgA1Post, Sort_Day)
```

```
##   Sort_Day  n
## 1   12-Jun 11
## 2   13-Jun 20
## 3   14-Jun 17
```

```
#plot %IgA by daily batch effect
p <- ggplot(metaIgA2, aes(x=Sort_Day, y=Percent_IgA)) + geom_boxplot()
p <- p + ggtitle("%IgA+ Bacteria")
p <- p + theme_bw(base_size=16)
p <- p + ylab("%IgA+ Bacteria") + xlab("Sort Day")
p <- p + guides(colour=FALSE, size=FALSE, shape=FALSE, fill=FALSE)
p <- p + annotate("text", x = 2, y=95, label = "****", size=4)
p <- p + annotate("segment", x=1, xend=3, y=90, yend=90)
p
```

```
p <- ggplot(metaIgA2, aes(x=Sort_Day, y=Percent_IgA, colour=SampleType2)) + geom_boxplot()
p <- p + ggtitle("%IgA+ Bacteria")
p <- p + theme_bw(base_size=16)
p <- p + ylab("%IgA+ Bacteria") + xlab("Sort Day")
p <- p + scale_colour_manual(values=c("seagreen3", "indianred3", "deepskyblue3"))
p
```

```
#are the main outcomes (sample type or donor ID) confounded by batch
fisher.test(metaIgA2$SampleType, metaIgA2$Sort_Day)
```

```
## 
##  Fisher's Exact Test for Count Data
## 
## data:  metaIgA2$SampleType and metaIgA2$Sort_Day
## p-value = 0.8308
## alternative hypothesis: two.sided
```

```
fisher.test(metaIgA1Post$Donor.ID, metaIgA1Post$Sort_Day) #p=0.056
```

```
## 
##  Fisher's Exact Test for Count Data
## 
## data:  metaIgA1Post$Donor.ID and metaIgA1Post$Sort_Day
## p-value = 0.06663
## alternative hypothesis: two.sided
```

```
p <- ggplot(metaIgA1Post, aes(x=Sort_Day, y=Percent_IgA, colour=as.factor(Donor.ID))) + geom_boxplot()
p <- p + ggtitle("%IgA+ Bacteria")
p <- p + theme_bw(base_size=16)
p <- p + ylab("%IgA+ Bacteria") + xlab("Sort Day")
p <- p + scale_colour_manual(values=c("lightskyblue1", "deepskyblue", "dodgerblue3", "plum4"))
p
```

```
#sample type, controlling for batch
m1 <- lm(Percent_IgA~Sort_Day + SampleType, data=metaIgA2)
summary(m1)
```

```
## 
## Call:
## lm(formula = Percent_IgA ~ Sort_Day + SampleType, data = metaIgA2)
## 
## Residuals:
##     Min      1Q  Median      3Q     Max 
## -24.474  -6.464  -2.994   4.092  51.349 
## 
## Coefficients:
##                          Estimate Std. Error t value Pr(>|t|)    
## (Intercept)                23.129      5.836   3.963 0.000141 ***
## Sort_Day13-Jun            -13.398      3.434  -3.902 0.000176 ***
## Sort_Day14-Jun            -22.073      3.485  -6.333  7.5e-09 ***
## SampleTypePreTransplant     6.045      5.798   1.043 0.299679    
## SampleTypePostTransplant    6.142      5.798   1.059 0.292028    
## ---
## Signif. codes:  0 '***' 0.001 '**' 0.01 '*' 0.05 '.' 0.1 ' ' 1
## 
## Residual standard error: 13.3 on 97 degrees of freedom
## Multiple R-squared:  0.2984, Adjusted R-squared:  0.2694 
## F-statistic: 10.31 on 4 and 97 DF,  p-value: 5.323e-07
```

```
#donor effect, controlling for batch
m2 <- lm(Percent_IgA~Sort_Day + as.factor(Donor.ID), data=metaIgA1Post)
summary(m2)
```

```
## 
## Call:
## lm(formula = Percent_IgA ~ Sort_Day + as.factor(Donor.ID), data = metaIgA1Post)
## 
## Residuals:
##     Min      1Q  Median      3Q     Max 
## -27.769  -8.606  -1.624   2.529  46.171 
## 
## Coefficients:
##                       Estimate Std. Error t value Pr(>|t|)    
## (Intercept)            28.0457     5.5912   5.016 1.01e-05 ***
## Sort_Day13-Jun        -14.2953     5.8727  -2.434 0.019257 *  
## Sort_Day14-Jun        -23.0967     6.3179  -3.656 0.000708 ***
## as.factor(Donor.ID)7    6.4033     5.3441   1.198 0.237560    
## as.factor(Donor.ID)20  -0.2439     5.7767  -0.042 0.966525    
## as.factor(Donor.ID)21  -5.6224    11.3830  -0.494 0.623932    
## ---
## Signif. codes:  0 '***' 0.001 '**' 0.01 '*' 0.05 '.' 0.1 ' ' 1
## 
## Residual standard error: 14.92 on 42 degrees of freedom
## Multiple R-squared:  0.3412, Adjusted R-squared:  0.2627 
## F-statistic:  4.35 on 5 and 42 DF,  p-value: 0.002798
```

```
#normalize all samples to median %IgA of that day
day1_median <- median((metaIgA2[which(metaIgA2$Sort_Day=="12-Jun"),])$Percent_IgA)
day2_median <-median((metaIgA2[which(metaIgA2$Sort_Day=="13-Jun"),])$Percent_IgA) 
day3_median <- median((metaIgA2[which(metaIgA2$Sort_Day=="14-Jun"),])$Percent_IgA) 

metaIgA2 <- metaIgA2[order(metaIgA2$Sort_Day),]
day1_perIgA_normalized <- (metaIgA2[which(metaIgA2$Sort_Day=="12-Jun"),]$Percent_IgA)/day1_median
day2_perIgA_normalized <- (metaIgA2[which(metaIgA2$Sort_Day=="13-Jun"),]$Percent_IgA)/day2_median
day3_perIgA_normalized <- (metaIgA2[which(metaIgA2$Sort_Day=="14-Jun"),]$Percent_IgA)/day3_median

metaIgA2$perIgA_normalized <- c(day1_perIgA_normalized, day2_perIgA_normalized, day3_perIgA_normalized)
metaIgA2 <- metaIgA2[order(metaIgA2$Patient),]

#save this data with patient info to include again later
df_PerIgA <- metaIgA2 %>% select(Patient, SampleType, Percent_IgA, perIgA_normalized)

#separate again
metaIgA2Pre <- filter(metaIgA2, SampleType=="PreTransplant")
metaIgA2Post <- filter(metaIgA2, SampleType=="PostTransplant")
metaIgA2_nodonor <- metaIgA2[metaIgA2$SampleType%in%c("PreTransplant", "PostTransplant"),]

#plot normalized %IgA by daily batch effect
p <- ggplot(metaIgA2, aes(x=Sort_Day, y=perIgA_normalized, colour=SampleType2)) + geom_boxplot()
p <- p + ggtitle("%IgA+ Bacteria (normalized)")
p <- p + theme_bw(base_size=16)
p <- p + ylab("%IgA+ Bacteria") + xlab("Sort Day")
p <- p + scale_colour_manual(values=c("seagreen3", "indianred3", "deepskyblue3"))
p
```

```
#paired
p <- ggplot(metaIgA2_nodonor) + geom_boxplot(aes(x=SampleType2, y=perIgA_normalized, fill=SampleType2),
                                    outlier.shape=NA, outlier.size=NA)
p <- p + geom_point(aes(x=SampleType2, y=perIgA_normalized),
                     shape=1, size=3)
p <- p + geom_line(aes(x=SampleType2, y=perIgA_normalized, group=Patient))
p <- p + ggtitle("%IgA+ Bacteria")
p <- p + theme_bw(base_size=15)
p <- p + ylab("%IgA+ Bacteria") + xlab("Sample Type")
p <- p + scale_fill_manual(values=c("indianred3", "deepskyblue3"))
p <- p + guides(colour=FALSE, size=FALSE, shape=FALSE, fill=FALSE)
#p <- p + annotate("text", x = 2, y=95, label = "ns", size=4)
#p <- p + annotate("segment", x=1, xend=3, y=90, yend=90)
p
```

```
#differences by sample type
#sample type - including donor
kruskal.test(metaIgA2$perIgA_normalized~metaIgA2$SampleType)
```

```
## 
##  Kruskal-Wallis rank sum test
## 
## data:  metaIgA2$perIgA_normalized by metaIgA2$SampleType
## Kruskal-Wallis chi-squared = 3.7757, df = 2, p-value = 0.1514
```

```
#sample type - not including donor
metaIgA2_nodonor <- filter(metaIgA2, SampleType %in% c("PreTransplant", "PostTransplant"))
wilcox.test(metaIgA2_nodonor$perIgA_normalized~metaIgA2_nodonor$SampleType)
```

```
## 
##  Wilcoxon rank sum test with continuity correction
## 
## data:  metaIgA2_nodonor$perIgA_normalized by metaIgA2_nodonor$SampleType
## W = 1245.5, p-value = 0.4956
## alternative hypothesis: true location shift is not equal to 0
```

```
#plot boxplot: %IgA by Sample Type
p <- ggplot(metaIgA2) + geom_boxplot(aes(x=SampleType2, y=perIgA_normalized, fill=SampleType2),
                                    outlier.shape=NA, outlier.size=NA)
p <- p + geom_jitter(aes(x=SampleType2, y=perIgA_normalized),
                     width=0.2, height=0, shape=1, size=3)
p <- p + ggtitle("%IgA+ Bacteria (Norm.)")
p <- p + theme_bw(base_size=14)
p <- p + ylab("%IgA+ Bacteria") + xlab("SampleType")
p <- p + scale_fill_manual(values=c("seagreen3", "indianred3", "deepskyblue3"))
p <- p + guides(colour=FALSE, size=FALSE, shape=FALSE, fill=FALSE)
p <- p + annotate("text", x = 2, y=15, label = "ns", size=4)
p <- p + annotate("segment", x=1, xend=3, y=14, yend=14)
p
```

```
#post-transplant by donor recipient
a1 <- kruskal.test(metaIgA2Post$perIgA_normalized ~ as.factor(metaIgA2Post$Donor.ID))
a1
```

```
## 
##  Kruskal-Wallis rank sum test
## 
## data:  metaIgA2Post$perIgA_normalized by as.factor(metaIgA2Post$Donor.ID)
## Kruskal-Wallis chi-squared = 2.7266, df = 3, p-value = 0.4357
```

```
posthoc <- dunnTest(perIgA_normalized ~ as.factor(Donor.ID),
              data=metaIgA2Post,
              method="bh") 
posthoc
```

```
## Dunn (1964) Kruskal-Wallis multiple comparison
```

```
##   p-values adjusted with the Benjamini-Hochberg method.
```

```
##   Comparison          Z   P.unadj     P.adj
## 1     2 - 20 -0.9757095 0.3292085 0.6584169
## 2     2 - 21  0.9551989 0.3394771 0.5092157
## 3    20 - 21  1.4308655 0.1524688 0.9148126
## 4      2 - 7 -0.7812388 0.4346620 0.5215944
## 5     20 - 7  0.2378311 0.8120121 0.8120121
## 6     21 - 7 -1.3320915 0.1828301 0.5484904
```

```
#plot boxplot: %IgA vs Donor
p <- ggplot(metaIgA2Post) + geom_boxplot(aes(x=as.factor(Donor.ID), y=perIgA_normalized, fill=as.factor(Donor.ID)),
                                        outlier.size=NA, outlier.shape=NA)
p <- p + geom_jitter(aes(x=as.factor(Donor.ID), y=perIgA_normalized),
                     width=0.2, height=0, shape=1, size=3)
p <- p + ggtitle("%IgA+ Bacteria (Post)")
p <- p + theme_bw(base_size=16)
p <- p + ylab("%IgA+ Bacteria") + xlab("Donor ID")
p <- p + scale_fill_manual(values=c("lightskyblue1", "deepskyblue", "dodgerblue3", "plum4"))
p <- p + guides(colour=FALSE, size=FALSE, shape=FALSE, fill=FALSE)
#p <- p + annotate("text", x = 1.5, y=45, label = "p=2.338e-05", size=4)
p
```

```
#compare to pre-transplant distribution
p <- ggplot(metaIgA2Pre) + geom_boxplot(aes(x=as.factor(Donor.ID), y=perIgA_normalized, fill=as.factor(Donor.ID)),
                                        outlier.size=NA, outlier.shape=NA)
p <- p + geom_jitter(aes(x=as.factor(Donor.ID), y=perIgA_normalized),
                     width=0.2, height=0, shape=1, size=3)
p <- p + ggtitle("%IgA+ Bacteria (Pre)")
p <- p + theme_bw(base_size=16)
p <- p + ylab("%IgA+ Bacteria") + xlab("Donor ID")
p <- p + scale_fill_manual(values=c("lightskyblue1", "deepskyblue", "dodgerblue3", "plum4"))
p <- p + guides(colour=FALSE, size=FALSE, shape=FALSE, fill=FALSE)
#p <- p + annotate("text", x = 1.5, y=45, label = "p=2.338e-05", size=4)
p
```

```
#gender?
wilcox.test(metaIgA2$perIgA_normalized~metaIgA2$Gender)
```

```
## 
##  Wilcoxon rank sum test with continuity correction
## 
## data:  metaIgA2$perIgA_normalized by metaIgA2$Gender
## W = 1107.5, p-value = 0.5189
## alternative hypothesis: true location shift is not equal to 0
```

```
p <- ggplot(metaIgA2) + geom_boxplot(aes(x=Gender, y=perIgA_normalized),
                                        outlier.size=NA, outlier.shape=NA)
p <- p + geom_jitter(aes(x=Gender, y=perIgA_normalized),
                     width=0.2, height=0, shape=1, size=3)
p <- p + ggtitle("%IgA+ Bacteria")
p <- p + theme_bw(base_size=16)
p <- p + ylab("%IgA+ Bacteria") + xlab("Gender")
p <- p + scale_fill_manual(values=c("lightskyblue1", "deepskyblue", "dodgerblue3", "plum4"))
p <- p + guides(colour=FALSE, size=FALSE, shape=FALSE, fill=FALSE)
#p <- p + annotate("text", x = 1.5, y=45, label = "p=2.338e-05", size=4)
p
```

```
#post transplant by delivery (colon vs capsule)
wilcox.test(metaIgA2Post$perIgA_normalized ~ metaIgA2Post$Delivery)
```

```
## 
##  Wilcoxon rank sum exact test
## 
## data:  metaIgA2Post$perIgA_normalized by metaIgA2Post$Delivery
## W = 289, p-value = 0.9837
## alternative hypothesis: true location shift is not equal to 0
```

```
p <- ggplot(metaIgA2Post) + geom_boxplot(aes(x=Delivery, y=perIgA_normalized),
                                         outlier.size=NA, outlier.shape=NA)
p <- p + geom_jitter(aes(x=Delivery, y=perIgA_normalized),
                     width=0.2, height=0, shape=1, size=3)
p <- p + ggtitle("%IgA+ Bacteria (Post)")
p <- p + theme_bw(base_size=15)
p <- p + ylab("%IgA+ Bacteria") + xlab("Delivery")
#p <- p + scale_fill_manual(values=c("lightskyblue1", "deepskyblue", "dodgerblue3", "plum4"))
p <- p + guides(colour=FALSE, size=FALSE, shape=FALSE, fill=FALSE)
p <- p + annotate("text", x = 1.5, y=10, label = "ns", size=4)
p <- p + annotate("segment", x=1, xend=2, y=9, yend=9)
p
```

# Process 16S data

```
#import from qiime
biom<-("FilesForR/feat-with-taxonomy-json.biom")
featurebiom<-import_biom(biom, parseFunction = parse_taxonomy_greengenes)
Metadata<-import_qiime_sample_data("FilesForR/cdiff-sample-metadata-FULL-JUNE2020.txt")
tree <- read_tree_greengenes("FilesForR/tree.nwk")
merge<-merge_phyloseq(featurebiom,Metadata,tree)
merge
```

```
## phyloseq-class experiment-level object
## otu_table()   OTU Table:         [ 1231 taxa and 215 samples ]
## sample_data() Sample Data:       [ 215 samples by 149 sample variables ]
## tax_table()   Taxonomy Table:    [ 1231 taxa by 7 taxonomic ranks ]
## phy_tree()    Phylogenetic Tree: [ 1231 tips and 1226 internal nodes ]
```

```
#prune unwanted taxa
taxotu <- data.frame(OTU = row.names(otu_table(merge)), tax_table(merge))
halotus <- subset(taxotu, Kingdom == "Archaea"  | Genus == "Halomonas" | Genus == "Shewanella" | 
                    Family == "mitochondria" | Class == "chloroplast" | Class == "Alphaproteobacteria")
halotus <- as.character(halotus[["OTU"]])

Table <- prune_taxa(setdiff(row.names(otu_table(merge)), halotus), merge)
Table <- prune_taxa(taxa_sums(Table) > 0, Table)

#prune low read count samples
Table_pruned1 <- prune_samples(sample_sums(Table)>=900, Table) #left with 189 samples

#also prune out corresponding sorted pair
samples_pruned <- prune_samples(sample_sums(Table)<900, Table)
pairs_to_exclude1 <- as.data.frame(sample_data(samples_pruned))$Patient
Table_pruned2 = subset_samples(Table_pruned1, !(Patient %in% pairs_to_exclude1)) 

#remove samples for missing paired data
Table_pruned2b = subset_samples(Table_pruned2, !(Patient%in%c("EDM-001","EDM-004",
                                                              "EDM-005")))

#remove singletons 
Table_pruned3 = prune_taxa(taxa_sums(Table_pruned2b) > 1, Table_pruned2b)
#Table_pruned3b = prune_taxa(taxa_sums(Table_pruned1) > 1, Table_pruned1) #to keep uneven sample pairs

#save sampling depth information before rarefying
sample_data(Table_pruned3)$sample_sums <- sample_sums(Table_pruned3)

#rarefy to 5000 reads
set.seed(3) #do a loop?
Table_pruned4 <- rarefy_even_depth(Table_pruned3, sample.size = 5000)
#and the corresponding pairs
samples_pruned <- prune_samples(sample_sums(Table_pruned3)<5000, Table_pruned3)
pairs_to_exclude2 <- as.data.frame(sample_data(samples_pruned))$Patient
Table_pruned5 = subset_samples(Table_pruned4, !(Patient %in% pairs_to_exclude2))

#transform into rel abund (rarefied)
Ig <- transform_sample_counts(Table_pruned5, function(OTU) OTU/sum(OTU))
#Table_pruned3b <- transform_sample_counts(Table_pruned3b, function(OTU) OTU/sum(OTU))
#transform into rel abund (un-rarefied)
#Ig <- transform_sample_counts(Table_pruned3, function(OTU) OTU/sum(OTU))

#add a pseudocount to transformed samples
otu_table(Ig) <- otu_table(Ig) + 0.0000001
```

Check included patient IDs

```
meta_kept <- as.data.frame(sample_data(Table_pruned5))
dplyr::count(meta_kept, SampleType) #note that everything is still duplicated for IgApos and IgAneg
```

```
## Warning in class(x) <- c(setdiff(subclass, tibble_class), tibble_class): Setting
## class(x) to multiple strings ("tbl_df", "tbl", ...); result will no longer be an
## S4 object
```

```
## Warning in class(x) <- c(setdiff(subclass, tibble_class), tibble_class): Setting
## class(x) to multiple strings ("grouped_df", "tbl_df", ...); result will no
## longer be an S4 object
```

```
## $SampleType
## [1] "Donor"          "PostTransplant" "PreTransplant" 
## 
## $n
## [1] 11 72 72
## 
## attr(,"row.names")
## [1] 1 2 3
## attr(,".S3Class")
## [1] "data.frame"
## attr(,"class")
## [1] "sample_data"
## attr(,"class")attr(,"package")
## [1] "phyloseq"
```

```
meta_kept_pre <- meta_kept[which(meta_kept$SampleType=="PreTransplant"),]
meta_kept_post <- meta_kept[which(meta_kept$SampleType=="PostTransplant"),]
meta_kept_donor <- meta_kept[which(meta_kept$SampleType=="Donor"),]

meta_kept_pre <- meta_kept_pre[order(meta_kept_pre$Patient),]
meta_kept_post <- meta_kept_post[order(meta_kept_post$Patient),]
meta_kept_pre$Patient==meta_kept_post$Patient #data is correctly paired
```

```
##  [1] TRUE TRUE TRUE TRUE TRUE TRUE TRUE TRUE TRUE TRUE TRUE TRUE TRUE TRUE TRUE
## [16] TRUE TRUE TRUE TRUE TRUE TRUE TRUE TRUE TRUE TRUE TRUE TRUE TRUE TRUE TRUE
## [31] TRUE TRUE TRUE TRUE TRUE TRUE TRUE TRUE TRUE TRUE TRUE TRUE TRUE TRUE TRUE
## [46] TRUE TRUE TRUE TRUE TRUE TRUE TRUE TRUE TRUE TRUE TRUE TRUE TRUE TRUE TRUE
## [61] TRUE TRUE TRUE TRUE TRUE TRUE TRUE TRUE TRUE TRUE TRUE TRUE
```

```
meta_kept_pre[which(meta_kept_pre$Sort=="IgApos"),]$Patient
```

```
##  [1] "EDM-003" "EDM-008" "EDM-009" "EDM-010" "EDM-011" "EDM-012" "EDM-013"
##  [8] "EDM-014" "EDM-016" "EDM-018" "EDM-019" "EDM-020" "EDM-021" "EDM-024"
## [15] "EDM-025" "EDM-026" "EDM-027" "EDM-028" "EDM-029" "EDM-030" "EDM-031"
## [22] "EDM-032" "EDM-034" "EDM-035" "EDM-036" "EDM-037" "EDM-038" "EDM-040"
## [29] "EDM-042" "EDM-043" "EDM-044" "EDM-046" "EDM-047" "EDM-057" "EDM-058"
## [36] "EDM-064"
```

```
meta_kept_post[which(meta_kept_post$Sort=="IgApos"),]$Patient
```

```
##  [1] "EDM-003" "EDM-008" "EDM-009" "EDM-010" "EDM-011" "EDM-012" "EDM-013"
##  [8] "EDM-014" "EDM-016" "EDM-018" "EDM-019" "EDM-020" "EDM-021" "EDM-024"
## [15] "EDM-025" "EDM-026" "EDM-027" "EDM-028" "EDM-029" "EDM-030" "EDM-031"
## [22] "EDM-032" "EDM-034" "EDM-035" "EDM-036" "EDM-037" "EDM-038" "EDM-040"
## [29] "EDM-042" "EDM-043" "EDM-044" "EDM-046" "EDM-047" "EDM-057" "EDM-058"
## [36] "EDM-064"
```

```
meta_kept_donor[which(meta_kept_donor$Sort=="IgApos"),]$Patient
```

```
## [1] "Donor2"  "Donor20" "Donor7"  "Donor7"  "Donor2"
```

```
meta_kept_donor[which(meta_kept_donor$Sort=="IgAneg"),]$Patient
```

```
## [1] "Donor7"  "Donor2"  "Donor2"  "Donor20" "Donor7"
```

```
#note that there is are two samples each for Donor 2 and for Donor 7 (two different fecal samples used for FMT, but from the same person)

#recode a simpler donor ID (1,2,3)
sample_data(Ig)$Donor.ID3 <- sample_data(Ig)$Donor.ID
sample_data(Ig)$Donor.ID3 <- gsub(20, 3, sample_data(Ig)$Donor.ID3)
sample_data(Ig)$Donor.ID3<- gsub(2, 1, sample_data(Ig)$Donor.ID3)
sample_data(Ig)$Donor.ID3 <- gsub(7, 2, sample_data(Ig)$Donor.ID3)
```

# Calculate IgA Index & an analagous relative abundance estimate

Pre- and Post- filtered separately

```
#subset for pre-transplant
IgPreTransplant <- subset_samples(Ig, SampleType=="PreTransplant")
#subset for sort
IgNeg_forindexPre = subset_samples(IgPreTransplant, Sort %in% c("IgAneg"))
IgPos_forindexPre = subset_samples(IgPreTransplant, Sort %in% c("IgApos"))
#make into dataframes
IgNegOTU <- data.frame(otu_table(IgNeg_forindexPre))
IgPosOTU <- data.frame(otu_table(IgPos_forindexPre))
#now the dataframes should be the same dimensions.
dim(IgNegOTU)
```

```
## [1] 1042   36
```

```
dim(IgPosOTU)
```

```
## [1] 1042   36
```

```
row.names(IgNegOTU)==row.names(IgPosOTU)
```

```
##    [1] TRUE TRUE TRUE TRUE TRUE TRUE TRUE TRUE TRUE TRUE TRUE TRUE TRUE TRUE
##   [15] TRUE TRUE TRUE TRUE TRUE TRUE TRUE TRUE TRUE TRUE TRUE TRUE TRUE TRUE
##   [29] TRUE TRUE TRUE TRUE TRUE TRUE TRUE TRUE TRUE TRUE TRUE TRUE TRUE TRUE
##   [43] TRUE TRUE TRUE TRUE TRUE TRUE TRUE TRUE TRUE TRUE TRUE TRUE TRUE TRUE
##   [57] TRUE TRUE TRUE TRUE TRUE TRUE TRUE TRUE TRUE TRUE TRUE TRUE TRUE TRUE
##   [71] TRUE TRUE TRUE TRUE TRUE TRUE TRUE TRUE TRUE TRUE TRUE TRUE TRUE TRUE
##   [85] TRUE TRUE TRUE TRUE TRUE TRUE TRUE TRUE TRUE TRUE TRUE TRUE TRUE TRUE
##   [99] TRUE TRUE TRUE TRUE TRUE TRUE TRUE TRUE TRUE TRUE TRUE TRUE TRUE TRUE
##  [113] TRUE TRUE TRUE TRUE TRUE TRUE TRUE TRUE TRUE TRUE TRUE TRUE TRUE TRUE
##  [127] TRUE TRUE TRUE TRUE TRUE TRUE TRUE TRUE TRUE TRUE TRUE TRUE TRUE TRUE
##  [141] TRUE TRUE TRUE TRUE TRUE TRUE TRUE TRUE TRUE TRUE TRUE TRUE TRUE TRUE
##  [155] TRUE TRUE TRUE TRUE TRUE TRUE TRUE TRUE TRUE TRUE TRUE TRUE TRUE TRUE
##  [169] TRUE TRUE TRUE TRUE TRUE TRUE TRUE TRUE TRUE TRUE TRUE TRUE TRUE TRUE
##  [183] TRUE TRUE TRUE TRUE TRUE TRUE TRUE TRUE TRUE TRUE TRUE TRUE TRUE TRUE
##  [197] TRUE TRUE TRUE TRUE TRUE TRUE TRUE TRUE TRUE TRUE TRUE TRUE TRUE TRUE
##  [211] TRUE TRUE TRUE TRUE TRUE TRUE TRUE TRUE TRUE TRUE TRUE TRUE TRUE TRUE
##  [225] TRUE TRUE TRUE TRUE TRUE TRUE TRUE TRUE TRUE TRUE TRUE TRUE TRUE TRUE
##  [239] TRUE TRUE TRUE TRUE TRUE TRUE TRUE TRUE TRUE TRUE TRUE TRUE TRUE TRUE
##  [253] TRUE TRUE TRUE TRUE TRUE TRUE TRUE TRUE TRUE TRUE TRUE TRUE TRUE TRUE
##  [267] TRUE TRUE TRUE TRUE TRUE TRUE TRUE TRUE TRUE TRUE TRUE TRUE TRUE TRUE
##  [281] TRUE TRUE TRUE TRUE TRUE TRUE TRUE TRUE TRUE TRUE TRUE TRUE TRUE TRUE
##  [295] TRUE TRUE TRUE TRUE TRUE TRUE TRUE TRUE TRUE TRUE TRUE TRUE TRUE TRUE
##  [309] TRUE TRUE TRUE TRUE TRUE TRUE TRUE TRUE TRUE TRUE TRUE TRUE TRUE TRUE
##  [323] TRUE TRUE TRUE TRUE TRUE TRUE TRUE TRUE TRUE TRUE TRUE TRUE TRUE TRUE
##  [337] TRUE TRUE TRUE TRUE TRUE TRUE TRUE TRUE TRUE TRUE TRUE TRUE TRUE TRUE
##  [351] TRUE TRUE TRUE TRUE TRUE TRUE TRUE TRUE TRUE TRUE TRUE TRUE TRUE TRUE
##  [365] TRUE TRUE TRUE TRUE TRUE TRUE TRUE TRUE TRUE TRUE TRUE TRUE TRUE TRUE
##  [379] TRUE TRUE TRUE TRUE TRUE TRUE TRUE TRUE TRUE TRUE TRUE TRUE TRUE TRUE
##  [393] TRUE TRUE TRUE TRUE TRUE TRUE TRUE TRUE TRUE TRUE TRUE TRUE TRUE TRUE
##  [407] TRUE TRUE TRUE TRUE TRUE TRUE TRUE TRUE TRUE TRUE TRUE TRUE TRUE TRUE
##  [421] TRUE TRUE TRUE TRUE TRUE TRUE TRUE TRUE TRUE TRUE TRUE TRUE TRUE TRUE
##  [435] TRUE TRUE TRUE TRUE TRUE TRUE TRUE TRUE TRUE TRUE TRUE TRUE TRUE TRUE
##  [449] TRUE TRUE TRUE TRUE TRUE TRUE TRUE TRUE TRUE TRUE TRUE TRUE TRUE TRUE
##  [463] TRUE TRUE TRUE TRUE TRUE TRUE TRUE TRUE TRUE TRUE TRUE TRUE TRUE TRUE
##  [477] TRUE TRUE TRUE TRUE TRUE TRUE TRUE TRUE TRUE TRUE TRUE TRUE TRUE TRUE
##  [491] TRUE TRUE TRUE TRUE TRUE TRUE TRUE TRUE TRUE TRUE TRUE TRUE TRUE TRUE
##  [505] TRUE TRUE TRUE TRUE TRUE TRUE TRUE TRUE TRUE TRUE TRUE TRUE TRUE TRUE
##  [519] TRUE TRUE TRUE TRUE TRUE TRUE TRUE TRUE TRUE TRUE TRUE TRUE TRUE TRUE
##  [533] TRUE TRUE TRUE TRUE TRUE TRUE TRUE TRUE TRUE TRUE TRUE TRUE TRUE TRUE
##  [547] TRUE TRUE TRUE TRUE TRUE TRUE TRUE TRUE TRUE TRUE TRUE TRUE TRUE TRUE
##  [561] TRUE TRUE TRUE TRUE TRUE TRUE TRUE TRUE TRUE TRUE TRUE TRUE TRUE TRUE
##  [575] TRUE TRUE TRUE TRUE TRUE TRUE TRUE TRUE TRUE TRUE TRUE TRUE TRUE TRUE
##  [589] TRUE TRUE TRUE TRUE TRUE TRUE TRUE TRUE TRUE TRUE TRUE TRUE TRUE TRUE
##  [603] TRUE TRUE TRUE TRUE TRUE TRUE TRUE TRUE TRUE TRUE TRUE TRUE TRUE TRUE
##  [617] TRUE TRUE TRUE TRUE TRUE TRUE TRUE TRUE TRUE TRUE TRUE TRUE TRUE TRUE
##  [631] TRUE TRUE TRUE TRUE TRUE TRUE TRUE TRUE TRUE TRUE TRUE TRUE TRUE TRUE
##  [645] TRUE TRUE TRUE TRUE TRUE TRUE TRUE TRUE TRUE TRUE TRUE TRUE TRUE TRUE
##  [659] TRUE TRUE TRUE TRUE TRUE TRUE TRUE TRUE TRUE TRUE TRUE TRUE TRUE TRUE
##  [673] TRUE TRUE TRUE TRUE TRUE TRUE TRUE TRUE TRUE TRUE TRUE TRUE TRUE TRUE
##  [687] TRUE TRUE TRUE TRUE TRUE TRUE TRUE TRUE TRUE TRUE TRUE TRUE TRUE TRUE
##  [701] TRUE TRUE TRUE TRUE TRUE TRUE TRUE TRUE TRUE TRUE TRUE TRUE TRUE TRUE
##  [715] TRUE TRUE TRUE TRUE TRUE TRUE TRUE TRUE TRUE TRUE TRUE TRUE TRUE TRUE
##  [729] TRUE TRUE TRUE TRUE TRUE TRUE TRUE TRUE TRUE TRUE TRUE TRUE TRUE TRUE
##  [743] TRUE TRUE TRUE TRUE TRUE TRUE TRUE TRUE TRUE TRUE TRUE TRUE TRUE TRUE
##  [757] TRUE TRUE TRUE TRUE TRUE TRUE TRUE TRUE TRUE TRUE TRUE TRUE TRUE TRUE
##  [771] TRUE TRUE TRUE TRUE TRUE TRUE TRUE TRUE TRUE TRUE TRUE TRUE TRUE TRUE
##  [785] TRUE TRUE TRUE TRUE TRUE TRUE TRUE TRUE TRUE TRUE TRUE TRUE TRUE TRUE
##  [799] TRUE TRUE TRUE TRUE TRUE TRUE TRUE TRUE TRUE TRUE TRUE TRUE TRUE TRUE
##  [813] TRUE TRUE TRUE TRUE TRUE TRUE TRUE TRUE TRUE TRUE TRUE TRUE TRUE TRUE
##  [827] TRUE TRUE TRUE TRUE TRUE TRUE TRUE TRUE TRUE TRUE TRUE TRUE TRUE TRUE
##  [841] TRUE TRUE TRUE TRUE TRUE TRUE TRUE TRUE TRUE TRUE TRUE TRUE TRUE TRUE
##  [855] TRUE TRUE TRUE TRUE TRUE TRUE TRUE TRUE TRUE TRUE TRUE TRUE TRUE TRUE
##  [869] TRUE TRUE TRUE TRUE TRUE TRUE TRUE TRUE TRUE TRUE TRUE TRUE TRUE TRUE
##  [883] TRUE TRUE TRUE TRUE TRUE TRUE TRUE TRUE TRUE TRUE TRUE TRUE TRUE TRUE
##  [897] TRUE TRUE TRUE TRUE TRUE TRUE TRUE TRUE TRUE TRUE TRUE TRUE TRUE TRUE
##  [911] TRUE TRUE TRUE TRUE TRUE TRUE TRUE TRUE TRUE TRUE TRUE TRUE TRUE TRUE
##  [925] TRUE TRUE TRUE TRUE TRUE TRUE TRUE TRUE TRUE TRUE TRUE TRUE TRUE TRUE
##  [939] TRUE TRUE TRUE TRUE TRUE TRUE TRUE TRUE TRUE TRUE TRUE TRUE TRUE TRUE
##  [953] TRUE TRUE TRUE TRUE TRUE TRUE TRUE TRUE TRUE TRUE TRUE TRUE TRUE TRUE
##  [967] TRUE TRUE TRUE TRUE TRUE TRUE TRUE TRUE TRUE TRUE TRUE TRUE TRUE TRUE
##  [981] TRUE TRUE TRUE TRUE TRUE TRUE TRUE TRUE TRUE TRUE TRUE TRUE TRUE TRUE
##  [995] TRUE TRUE TRUE TRUE TRUE TRUE TRUE TRUE TRUE TRUE TRUE TRUE TRUE TRUE
## [1009] TRUE TRUE TRUE TRUE TRUE TRUE TRUE TRUE TRUE TRUE TRUE TRUE TRUE TRUE
## [1023] TRUE TRUE TRUE TRUE TRUE TRUE TRUE TRUE TRUE TRUE TRUE TRUE TRUE TRUE
## [1037] TRUE TRUE TRUE TRUE TRUE TRUE
```

```
#taxa are already ordered; order by patient
IgNegOTU <- t(IgNegOTU)
IgNeg_Order <- IgNegOTU[order(row.names(IgNegOTU)), ]
IgPosOTU <- t(IgPosOTU)
IgPos_Order <- IgPosOTU[order(row.names(IgPosOTU)), ]

row.names(IgNeg_Order)
```

```
##  [1] "KH.00978neg" "KH.01090neg" "KH.01114neg" "KH.01127neg" "KH.01151neg"
##  [6] "KH.01163neg" "KH.01164neg" "KH.01190neg" "KH.01203neg" "KH.01229neg"
## [11] "KH.01241neg" "KH.01271neg" "KH.01296neg" "KH.01356neg" "KH.01357neg"
## [16] "KH.01380neg" "KH.01413neg" "KH.01443neg" "KH.01472neg" "KH.01506neg"
## [21] "KH.01513neg" "KH.01522neg" "KH.01529neg" "KH.01535neg" "KH.01551neg"
## [26] "KH.01578neg" "KH.01594neg" "KH.01644neg" "KH.01666neg" "KH.01681neg"
## [31] "KH.01682neg" "KH.01709neg" "KH.01720neg" "KH.01870neg" "KH.01877neg"
## [36] "KH.01986neg"
```

```
row.names(IgPos_Order)
```

```
##  [1] "KH.00978pos" "KH.01090pos" "KH.01114pos" "KH.01127pos" "KH.01151pos"
##  [6] "KH.01163pos" "KH.01164pos" "KH.01190pos" "KH.01203pos" "KH.01229pos"
## [11] "KH.01241pos" "KH.01271pos" "KH.01296pos" "KH.01356pos" "KH.01357pos"
## [16] "KH.01380pos" "KH.01413pos" "KH.01443pos" "KH.01472pos" "KH.01506pos"
## [21] "KH.01513pos" "KH.01522pos" "KH.01529pos" "KH.01535pos" "KH.01551pos"
## [26] "KH.01578pos" "KH.01594pos" "KH.01644pos" "KH.01666pos" "KH.01681pos"
## [31] "KH.01682pos" "KH.01709pos" "KH.01720pos" "KH.01870pos" "KH.01877pos"
## [36] "KH.01986pos"
```

```
#Rel abund estimate
AbundPre <- IgPos_Order + IgNeg_Order

#Log transform and calculate IgA Index.
IgNegLog <- log(IgNeg_Order)
IgPosLog <- log(IgPos_Order)
IgAIndexPre <- -(IgPosLog - IgNegLog)/(IgPosLog + IgNegLog)

#filter IgA Index
IgA_t <- as.data.frame(t(IgAIndexPre))
IgAPre_trim75 <- IgA_t[rowSums(IgA_t==0) <=(length(IgA_t)*0.75), ]
dim(IgAPre_trim75)
```

```
## [1] 42 36
```

```
#more stringent filtering for certain analyses
IgAPre_trim50 <- IgA_t[rowSums(IgA_t==0) <=(length(IgA_t)*0.50), ]
dim(IgAPre_trim50)
```

```
## [1] 21 36
```

```
#add full taxonomy ID to the IgA Index
IgTAX <- data.frame(tax_table(IgPos_forindexPre))
IgTAX$ASV <- row.names(IgTAX)
IgTAX$combined <- paste(IgTAX$Rank1, IgTAX$Kingdom, IgTAX$Phylum, IgTAX$Class, IgTAX$Order, IgTAX$Family, IgTAX$Genus, IgTAX$Species, IgTAX$ASV, sep="|")

TaxaToKeep75Pre <- row.names(IgAPre_trim75)
IgTAX75 <- filter(IgTAX, ASV %in% TaxaToKeep75Pre)
IgAPre_trim75 <- IgAPre_trim75[order(row.names(IgAPre_trim75)),]
IgTAX75 <- IgTAX75[order(IgTAX75$ASV),]
row.names(IgAPre_trim75) <- IgTAX75$combined

IgAPre_trim75_t <- as.data.frame(t(IgAPre_trim75))

TaxaToKeep50Pre <- row.names(IgAPre_trim50)
IgTAX50 <- filter(IgTAX, ASV %in% TaxaToKeep50Pre)
IgAPre_trim50 <- IgAPre_trim50[order(row.names(IgAPre_trim50)),]
IgTAX50 <- IgTAX50[order(IgTAX50$ASV),]
row.names(IgAPre_trim50) <- IgTAX50$combined

IgAPre_trim50_t <- as.data.frame(t(IgAPre_trim50))

#filter abundance estimate to keep the exact same taxa
Abund_t <- as.data.frame(t(AbundPre))
AbundPre_trim75 <- Abund_t[row.names(Abund_t)%in%TaxaToKeep75Pre, ]
dim(AbundPre_trim75)
```

```
## [1] 42 36
```

```
AbundPre_trim75 <- AbundPre_trim75[order(row.names(AbundPre_trim75)),]
row.names(AbundPre_trim75) <- IgTAX75$combined

AbundPre_trim50 <- Abund_t[row.names(Abund_t)%in%TaxaToKeep50Pre, ]
dim(AbundPre_trim50)
```

```
## [1] 21 36
```

```
AbundPre_trim50 <- AbundPre_trim50[order(row.names(AbundPre_trim50)),]
row.names(AbundPre_trim50) <- IgTAX50$combined

#transform to relative abundance by sample
AbundPre_trim75_t <- as.data.frame(t(AbundPre_trim75))
AbundPre_trim75_t <- AbundPre_trim75_t/rowSums(AbundPre_trim75_t)

AbundPre_trim50_t <- as.data.frame(t(AbundPre_trim50))
AbundPre_trim50_t <- AbundPre_trim50_t/rowSums(AbundPre_trim50_t)

#metadata
metaIgAPre <- as.data.frame(sample_data(IgPos_forindexPre))
metaIgAPre <- metaIgAPre[order(metaIgAPre$SampleID), ]

row.names(IgAPre_trim75_t)==gsub("-", ".", metaIgAPre$SampleID)
```

```
##  [1] TRUE TRUE TRUE TRUE TRUE TRUE TRUE TRUE TRUE TRUE TRUE TRUE TRUE TRUE TRUE
## [16] TRUE TRUE TRUE TRUE TRUE TRUE TRUE TRUE TRUE TRUE TRUE TRUE TRUE TRUE TRUE
## [31] TRUE TRUE TRUE TRUE TRUE TRUE
```

```
row.names(AbundPre_trim75_t)==gsub("-", ".", metaIgAPre$SampleID)
```

```
##  [1] TRUE TRUE TRUE TRUE TRUE TRUE TRUE TRUE TRUE TRUE TRUE TRUE TRUE TRUE TRUE
## [16] TRUE TRUE TRUE TRUE TRUE TRUE TRUE TRUE TRUE TRUE TRUE TRUE TRUE TRUE TRUE
## [31] TRUE TRUE TRUE TRUE TRUE TRUE
```

```
row.names(IgAPre_trim75_t) <- metaIgAPre$Patient
row.names(AbundPre_trim75_t) <- metaIgAPre$Patient

row.names(IgAPre_trim50_t)==gsub("-", ".", metaIgAPre$SampleID)
```

```
##  [1] TRUE TRUE TRUE TRUE TRUE TRUE TRUE TRUE TRUE TRUE TRUE TRUE TRUE TRUE TRUE
## [16] TRUE TRUE TRUE TRUE TRUE TRUE TRUE TRUE TRUE TRUE TRUE TRUE TRUE TRUE TRUE
## [31] TRUE TRUE TRUE TRUE TRUE TRUE
```

```
row.names(AbundPre_trim50_t)==gsub("-", ".", metaIgAPre$SampleID)
```

```
##  [1] TRUE TRUE TRUE TRUE TRUE TRUE TRUE TRUE TRUE TRUE TRUE TRUE TRUE TRUE TRUE
## [16] TRUE TRUE TRUE TRUE TRUE TRUE TRUE TRUE TRUE TRUE TRUE TRUE TRUE TRUE TRUE
## [31] TRUE TRUE TRUE TRUE TRUE TRUE
```

```
row.names(IgAPre_trim50_t) <- metaIgAPre$Patient
row.names(AbundPre_trim50_t) <- metaIgAPre$Patient

##POST
#subset1
IgPostTransplant <- subset_samples(Ig, SampleType=="PostTransplant")
#subset2
IgNeg_forindexPost = subset_samples(IgPostTransplant, Sort %in% c("IgAneg"))
IgPos_forindexPost = subset_samples(IgPostTransplant, Sort %in% c("IgApos"))
#make into dataframes
IgNegOTU <- data.frame(otu_table(IgNeg_forindexPost))
IgPosOTU <- data.frame(otu_table(IgPos_forindexPost))
#now the dataframes should be the same dimensions.
dim(IgNegOTU)
```

```
## [1] 1042   36
```

```
dim(IgPosOTU)
```

```
## [1] 1042   36
```

```
#but they don't seem to be in the same order. Transpose, then "order".
IgNegOTU <- t(IgNegOTU)
IgNeg_Order <- IgNegOTU[ order(row.names(IgNegOTU)), ]
IgPosOTU <- t(IgPosOTU)
IgPos_Order <- IgPosOTU[ order(row.names(IgPosOTU)), ]

#Rel abund estimate
AbundPost <- IgPos_Order + IgNeg_Order

#Log transform and calculate IgA Index.
IgNegLog <- log(IgNeg_Order)
IgPosLog <- log(IgPos_Order)
IgAIndexPost <- -(IgPosLog - IgNegLog)/(IgPosLog + IgNegLog)

#filter
IgA_t <- as.data.frame(t(IgAIndexPost))
IgAPost_trim75 <- IgA_t[rowSums(IgA_t==0) <=(length(IgA_t)*0.75), ]
dim(IgAPost_trim75)
```

```
## [1] 180  36
```

```
#more stringent filtering for certain analyses
IgAPost_trim50 <- IgA_t[rowSums(IgA_t==0) <=(length(IgA_t)*0.50), ]
dim(IgAPost_trim50)
```

```
## [1] 68 36
```

```
#add taxonomy
IgTAX <- data.frame(tax_table(IgPos_forindexPost))
IgTAX$ASV <- row.names(IgTAX)
IgTAX$combined <- paste(IgTAX$Rank1, IgTAX$Kingdom, IgTAX$Phylum, IgTAX$Class, IgTAX$Order, IgTAX$Family, IgTAX$Genus, IgTAX$Species, IgTAX$ASV, sep="|")

TaxaToKeep75Post <- row.names(IgAPost_trim75)
IgTAX75 <- filter(IgTAX, ASV %in% TaxaToKeep75Post)
IgAPost_trim75 <- IgAPost_trim75[order(row.names(IgAPost_trim75)),]
IgTAX75 <- IgTAX75[order(IgTAX75$ASV),]
row.names(IgAPost_trim75) <- IgTAX75$combined

IgAPost_trim75_t <- as.data.frame(t(IgAPost_trim75))

TaxaToKeep50Post <- row.names(IgAPost_trim50)
IgTAX50 <- filter(IgTAX, ASV %in% TaxaToKeep50Post)
IgAPost_trim50 <- IgAPost_trim50[order(row.names(IgAPost_trim50)),]
IgTAX50 <- IgTAX50[order(IgTAX50$ASV),]
row.names(IgAPost_trim50) <- IgTAX50$combined

IgAPost_trim50_t <- as.data.frame(t(IgAPost_trim50))

#filter abundance estimate to keep the exact same taxa
Abund_t <- as.data.frame(t(AbundPost))
AbundPost_trim75 <- Abund_t[row.names(Abund_t)%in%TaxaToKeep75Post, ]
dim(AbundPost_trim75)
```

```
## [1] 180  36
```

```
AbundPost_trim75 <- AbundPost_trim75[order(row.names(AbundPost_trim75)),]
row.names(AbundPost_trim75) <- IgTAX75$combined

AbundPost_trim50 <- Abund_t[row.names(Abund_t)%in%TaxaToKeep50Post, ]
dim(AbundPost_trim50)
```

```
## [1] 68 36
```

```
AbundPost_trim50 <- AbundPost_trim50[order(row.names(AbundPost_trim50)),]
row.names(AbundPost_trim50) <- IgTAX50$combined

#transform to relative abundance by sample
AbundPost_trim75_t <- as.data.frame(t(AbundPost_trim75))
AbundPost_trim75_t <- AbundPost_trim75_t/rowSums(AbundPost_trim75_t)

AbundPost_trim50_t <- as.data.frame(t(AbundPost_trim50))
AbundPost_trim50_t <- AbundPost_trim50_t/rowSums(AbundPost_trim50_t)

#metadata
metaIgAPost <- as.data.frame(sample_data(IgPos_forindexPost))
metaIgAPost <- metaIgAPost[ order(metaIgAPost$SampleID), ]

row.names(IgAPost_trim75_t)==gsub("-", ".", metaIgAPost$SampleID)
```

```
##  [1] TRUE TRUE TRUE TRUE TRUE TRUE TRUE TRUE TRUE TRUE TRUE TRUE TRUE TRUE TRUE
## [16] TRUE TRUE TRUE TRUE TRUE TRUE TRUE TRUE TRUE TRUE TRUE TRUE TRUE TRUE TRUE
## [31] TRUE TRUE TRUE TRUE TRUE TRUE
```

```
row.names(AbundPost_trim75_t)==gsub("-", ".", metaIgAPost$SampleID)
```

```
##  [1] TRUE TRUE TRUE TRUE TRUE TRUE TRUE TRUE TRUE TRUE TRUE TRUE TRUE TRUE TRUE
## [16] TRUE TRUE TRUE TRUE TRUE TRUE TRUE TRUE TRUE TRUE TRUE TRUE TRUE TRUE TRUE
## [31] TRUE TRUE TRUE TRUE TRUE TRUE
```

```
row.names(IgAPost_trim75_t) <- metaIgAPost$Patient
row.names(AbundPost_trim75_t) <- metaIgAPost$Patient

row.names(IgAPost_trim50_t)==gsub("-", ".", metaIgAPost$SampleID)
```

```
##  [1] TRUE TRUE TRUE TRUE TRUE TRUE TRUE TRUE TRUE TRUE TRUE TRUE TRUE TRUE TRUE
## [16] TRUE TRUE TRUE TRUE TRUE TRUE TRUE TRUE TRUE TRUE TRUE TRUE TRUE TRUE TRUE
## [31] TRUE TRUE TRUE TRUE TRUE TRUE
```

```
row.names(AbundPost_trim50_t)==gsub("-", ".", metaIgAPost$SampleID)
```

```
##  [1] TRUE TRUE TRUE TRUE TRUE TRUE TRUE TRUE TRUE TRUE TRUE TRUE TRUE TRUE TRUE
## [16] TRUE TRUE TRUE TRUE TRUE TRUE TRUE TRUE TRUE TRUE TRUE TRUE TRUE TRUE TRUE
## [31] TRUE TRUE TRUE TRUE TRUE TRUE
```

```
row.names(IgAPost_trim50_t) <- metaIgAPost$Patient
row.names(AbundPost_trim50_t) <- metaIgAPost$Patient
```

Full dataset together

```
#subset
IgNeg_forindex = subset_samples(Ig, Sort %in% c("IgAneg"))
IgPos_forindex = subset_samples(Ig, Sort %in% c("IgApos"))
#make into dataframes
IgNegOTU <- data.frame(otu_table(IgNeg_forindex))
IgPosOTU <- data.frame(otu_table(IgPos_forindex))
#now the dataframes should be the same dimensions.
dim(IgNegOTU)
```

```
## [1] 1042   77
```

```
dim(IgPosOTU)
```

```
## [1] 1042   77
```

```
#but they don't seem to be in the same order. Transpose, then "order".
IgNegOTU <- t(IgNegOTU)
IgNeg_Order <- IgNegOTU[ order(row.names(IgNegOTU)), ]
IgPosOTU <- t(IgPosOTU)
IgPos_Order <- IgPosOTU[ order(row.names(IgPosOTU)), ]
#Log transform and calculate IgA Index.
IgNegLog <- log(IgNeg_Order)
IgPosLog <- log(IgPos_Order)
IgAIndex <- -(IgPosLog - IgNegLog)/(IgPosLog + IgNegLog)

#Rel abund estimate
Abund <- IgPos_Order + IgNeg_Order

#
IgA_t <- as.data.frame(t(IgAIndex))
Abund_t <- as.data.frame(t(Abund))

#add full taxonomy ID to the IgA Index & abundance
IgTAX <- data.frame(tax_table(IgPos_forindex))
IgTAX$ASV <- row.names(IgTAX)
IgTAX$combined <- paste(IgTAX$Rank1, IgTAX$Kingdom, IgTAX$Phylum, IgTAX$Class, IgTAX$Order, IgTAX$Family, IgTAX$Genus, IgTAX$Species, IgTAX$ASV, sep="|")

#metadata
metaIgA <- as.data.frame(sample_data(IgPos_forindex))
metaIgA <- metaIgA[ order(metaIgA$SampleID), ]

#filter by taxa prevalent within each sampletype (pre vs post transplant)
pretaxa75 <- row.names(IgAPre_trim75)
posttaxa75 <- row.names(IgAPost_trim75)

pretaxa50 <- row.names(IgAPre_trim50)
posttaxa50 <- row.names(IgAPost_trim50)

head(row.names(IgA_t)==IgTAX$ASV, 50)
```

```
##  [1] TRUE TRUE TRUE TRUE TRUE TRUE TRUE TRUE TRUE TRUE TRUE TRUE TRUE TRUE TRUE
## [16] TRUE TRUE TRUE TRUE TRUE TRUE TRUE TRUE TRUE TRUE TRUE TRUE TRUE TRUE TRUE
## [31] TRUE TRUE TRUE TRUE TRUE TRUE TRUE TRUE TRUE TRUE TRUE TRUE TRUE TRUE TRUE
## [46] TRUE TRUE TRUE TRUE TRUE
```

```
IgA_t$Taxa <- IgTAX$combined
IgA_settrim75 <- filter(IgA_t, Taxa %in% pretaxa75 | Taxa %in% posttaxa75) 
row.names(IgA_settrim75) <- IgA_settrim75$Taxa
IgA_settrim75$Taxa <- NULL

IgA_settrim75_t <- as.data.frame(t(IgA_settrim75))
dim(IgA_settrim75_t)
```

```
## [1]  77 197
```

```
IgA_settrim50 <- filter(IgA_t, Taxa %in% pretaxa50 | Taxa %in% posttaxa50) 
row.names(IgA_settrim50) <- IgA_settrim50$Taxa
IgA_settrim50$Taxa <- NULL

IgA_settrim75_t <- as.data.frame(t(IgA_settrim75))
dim(IgA_settrim75_t)
```

```
## [1]  77 197
```

```
IgA_settrim50_t <- as.data.frame(t(IgA_settrim50))
dim(IgA_settrim50_t)
```

```
## [1] 77 75
```

```
#filter abundance estimate to keep the exact same taxa
head(row.names(Abund_t)==IgTAX$ASV, 50)
```

```
##  [1] TRUE TRUE TRUE TRUE TRUE TRUE TRUE TRUE TRUE TRUE TRUE TRUE TRUE TRUE TRUE
## [16] TRUE TRUE TRUE TRUE TRUE TRUE TRUE TRUE TRUE TRUE TRUE TRUE TRUE TRUE TRUE
## [31] TRUE TRUE TRUE TRUE TRUE TRUE TRUE TRUE TRUE TRUE TRUE TRUE TRUE TRUE TRUE
## [46] TRUE TRUE TRUE TRUE TRUE
```

```
Abund_t$Taxa <- IgTAX$combined
Abund_settrim75 <- filter(Abund_t, Taxa %in% pretaxa75 | Taxa %in% posttaxa75) 
row.names(Abund_settrim75) <- Abund_settrim75$Taxa
Abund_settrim75$Taxa <- NULL

Abund_settrim50 <- filter(Abund_t, Taxa %in% pretaxa50 | Taxa %in% posttaxa50) 
row.names(Abund_settrim50) <- Abund_settrim50$Taxa
Abund_settrim50$Taxa <- NULL

#transform to relative abundance by sample
Abund_settrim75_t <- as.data.frame(t(Abund_settrim75))
Abund_settrim75_t <- Abund_settrim75_t/rowSums(Abund_settrim75_t)

Abund_settrim50_t <- as.data.frame(t(Abund_settrim50))
Abund_settrim50_t <- Abund_settrim50_t/rowSums(Abund_settrim50_t)
```

# Relative abundance alpha and beta diversity

```
#alpha
shannon_diversity <- diversity(Abund_settrim75_t, index = "shannon")
shannon_diversity <- data.frame(SampleID=row.names(Abund_settrim75_t), shannon_diversity=shannon_diversity)

#alpha significance
shannon_diversity$SampleID==gsub("-", ".", metaIgA$SampleID, fixed=TRUE)
```

```
##  [1] TRUE TRUE TRUE TRUE TRUE TRUE TRUE TRUE TRUE TRUE TRUE TRUE TRUE TRUE TRUE
## [16] TRUE TRUE TRUE TRUE TRUE TRUE TRUE TRUE TRUE TRUE TRUE TRUE TRUE TRUE TRUE
## [31] TRUE TRUE TRUE TRUE TRUE TRUE TRUE TRUE TRUE TRUE TRUE TRUE TRUE TRUE TRUE
## [46] TRUE TRUE TRUE TRUE TRUE TRUE TRUE TRUE TRUE TRUE TRUE TRUE TRUE TRUE TRUE
## [61] TRUE TRUE TRUE TRUE TRUE TRUE TRUE TRUE TRUE TRUE TRUE TRUE TRUE TRUE TRUE
## [76] TRUE TRUE
```

```
pdata <- data.frame(shannon_diversity, metaIgA)
a3 <- kruskal.test(pdata$shannon_diversity ~ pdata$SampleType)
a3
```

```
## 
##  Kruskal-Wallis rank sum test
## 
## data:  pdata$shannon_diversity by pdata$SampleType
## Kruskal-Wallis chi-squared = 43.498, df = 2, p-value = 3.586e-10
```

```
posthoc <- dunnTest(shannon_diversity~SampleType, data=pdata, method='bh')
posthoc
```

```
##                       Comparison           Z      P.unadj        P.adj
## 1         Donor - PostTransplant -0.01665021 9.867157e-01 9.867157e-01
## 2          Donor - PreTransplant  3.14168680 1.679776e-03 2.519664e-03
## 3 PostTransplant - PreTransplant  6.39514503 1.603950e-10 4.811851e-10
```

```
#alpha plot
pdata$SampleType2 <- pdata$SampleType
pdata$SampleType2 <- gsub("PreTransplant", "Pre", pdata$SampleType2)
pdata$SampleType2 <- gsub("PostTransplant", "Post", pdata$SampleType2)
pdata$SampleType2 <- factor(pdata$SampleType2, 
                              levels=c("Donor", "Pre", "Post"))


p <- ggplot(pdata) + geom_boxplot(aes(x=SampleType2, y=shannon_diversity, fill=SampleType2),
                                  outlier.shape=1, outlier.size=4)
p <- p + ggtitle("Alpha diversity")
p <- p + theme_bw(base_size=15)
p <- p + scale_fill_manual(values=c("seagreen3", "indianred3", "deepskyblue3"))
p <- p + xlab("SampleType") + ylab("Shannon Diversity")
p <- p + annotate("segment", x=1.1, xend= 1.9, y=4, yend=4)
p <- p + annotate("segment", x=2.1, xend= 2.9, y=4, yend=4)
p <- p + annotate("text", x = 1.5, y=4, label = "**", size=5)
p <- p + annotate("text", x = 2.5, y=4, label = "****", size=5)
p <- p + guides(colour=FALSE, size=FALSE, shape=FALSE, fill=FALSE)
p
```

```
#beta diversity
#bray curtis
meta_abund <- sample_data(metaIgA)


IgTAX_abund <- IgTAX[which(IgTAX$combined %in% row.names(Abund_settrim75)),]
IgTAX_abund$combined == row.names(Abund_settrim75)
```

```
##   [1] TRUE TRUE TRUE TRUE TRUE TRUE TRUE TRUE TRUE TRUE TRUE TRUE TRUE TRUE TRUE
##  [16] TRUE TRUE TRUE TRUE TRUE TRUE TRUE TRUE TRUE TRUE TRUE TRUE TRUE TRUE TRUE
##  [31] TRUE TRUE TRUE TRUE TRUE TRUE TRUE TRUE TRUE TRUE TRUE TRUE TRUE TRUE TRUE
##  [46] TRUE TRUE TRUE TRUE TRUE TRUE TRUE TRUE TRUE TRUE TRUE TRUE TRUE TRUE TRUE
##  [61] TRUE TRUE TRUE TRUE TRUE TRUE TRUE TRUE TRUE TRUE TRUE TRUE TRUE TRUE TRUE
##  [76] TRUE TRUE TRUE TRUE TRUE TRUE TRUE TRUE TRUE TRUE TRUE TRUE TRUE TRUE TRUE
##  [91] TRUE TRUE TRUE TRUE TRUE TRUE TRUE TRUE TRUE TRUE TRUE TRUE TRUE TRUE TRUE
## [106] TRUE TRUE TRUE TRUE TRUE TRUE TRUE TRUE TRUE TRUE TRUE TRUE TRUE TRUE TRUE
## [121] TRUE TRUE TRUE TRUE TRUE TRUE TRUE TRUE TRUE TRUE TRUE TRUE TRUE TRUE TRUE
## [136] TRUE TRUE TRUE TRUE TRUE TRUE TRUE TRUE TRUE TRUE TRUE TRUE TRUE TRUE TRUE
## [151] TRUE TRUE TRUE TRUE TRUE TRUE TRUE TRUE TRUE TRUE TRUE TRUE TRUE TRUE TRUE
## [166] TRUE TRUE TRUE TRUE TRUE TRUE TRUE TRUE TRUE TRUE TRUE TRUE TRUE TRUE TRUE
## [181] TRUE TRUE TRUE TRUE TRUE TRUE TRUE TRUE TRUE TRUE TRUE TRUE TRUE TRUE TRUE
## [196] TRUE TRUE
```

```
row.names(Abund_settrim75) <- IgTAX_abund$ASV
row.names(IgTAX_abund) <- IgTAX_abund$ASV
IgTAX_abund$combined <- NULL
IgTAX_abund$ASV <- NULL
tax_abund <- tax_table(IgTAX_abund)
otu_abund <- otu_table(Abund_settrim75, taxa_are_rows = TRUE)
taxa_names(tax_abund) <- row.names(IgTAX_abund)
sample_names(meta_abund) <- gsub("-", ".", sample_names(meta_abund), fixed=TRUE)

phylo_abund <- merge_phyloseq(otu_abund, tax_abund, meta_abund)
braydist = phyloseq::distance(phylo_abund, method = "bray")
adonis(braydist ~ metaIgA$SampleType, permutations = 4999)
```

```
## 
## Call:
## adonis(formula = braydist ~ metaIgA$SampleType, permutations = 4999) 
## 
## Permutation: free
## Number of permutations: 4999
## 
## Terms added sequentially (first to last)
## 
##                    Df SumsOfSqs MeanSqs F.Model      R2 Pr(>F)    
## metaIgA$SampleType  2     6.132 3.06602   10.37 0.21892  2e-04 ***
## Residuals          74    21.878 0.29565         0.78108           
## Total              76    28.010                 1.00000           
## ---
## Signif. codes:  0 '***' 0.001 '**' 0.01 '*' 0.05 '.' 0.1 ' ' 1
```

```
#ordinations. make a PCoA plot.
expt1.ord <- ordinate(phylo_abund, "PCoA", "bray")
#PCoA Bray-Curtis

p1 = plot_ordination(phylo_abund, expt1.ord, type = "samples", color = "SampleType", title = "PCoA (Bray-Curtis)")
p1 <- p1 +  geom_point(aes(colour=SampleType), size=4)
p1 <- p1 + scale_colour_manual(values=c("seagreen3", "indianred3","deepskyblue3"))
p1 <- p1 + theme_bw(base_size=16)
p1 <- p1 + annotate("text", x = 0.25, y=-0.2, label = "p=0.0002", size=4 )
p1
```

```
#post-transplant only by donor

#alpha
shannon_diversity <- diversity(AbundPost_trim75_t, index = "shannon")
shannon_diversity <- data.frame(SampleID=row.names(AbundPost_trim75_t),
                                shannon_diversity=shannon_diversity)

#alpha significance
shannon_diversity$SampleID==metaIgAPost$Patient
```

```
##  [1] TRUE TRUE TRUE TRUE TRUE TRUE TRUE TRUE TRUE TRUE TRUE TRUE TRUE TRUE TRUE
## [16] TRUE TRUE TRUE TRUE TRUE TRUE TRUE TRUE TRUE TRUE TRUE TRUE TRUE TRUE TRUE
## [31] TRUE TRUE TRUE TRUE TRUE TRUE
```

```
pdata <- data.frame(shannon_diversity, metaIgAPost)
pdata$Donor.ID2 <- paste("Donor", pdata$Donor.ID, sep="")
#exclude donor 21 (as per the IgA analysis - too small sample size)
pdata_filt <- pdata[which(pdata$Donor.ID2!="Donor21"),]
a3 <- kruskal.test(pdata_filt$shannon_diversity ~ pdata_filt$Donor.ID2)
a3
```

```
## 
##  Kruskal-Wallis rank sum test
## 
## data:  pdata_filt$shannon_diversity by pdata_filt$Donor.ID2
## Kruskal-Wallis chi-squared = 9.9411, df = 2, p-value = 0.006939
```

```
posthoc <- dunnTest(shannon_diversity~Donor.ID2, data=pdata_filt, method='bh')
posthoc
```

```
##         Comparison          Z     P.unadj       P.adj
## 1 Donor2 - Donor20  2.9875375 0.002812348 0.008437043
## 2  Donor2 - Donor7  0.3573382 0.720838663 0.720838663
## 3 Donor20 - Donor7 -2.5758413 0.009999653 0.014999479
```

```
#alpha plot
pdata_filt$Donor.ID3 <- pdata_filt$Donor.ID2
pdata_filt$Donor.ID3 <- gsub("Donor20", "Donor3", pdata_filt$Donor.ID3)
pdata_filt$Donor.ID3 <- gsub("Donor2", "Donor1", pdata_filt$Donor.ID3)
pdata_filt$Donor.ID3 <- gsub("Donor7", "Donor2", pdata_filt$Donor.ID3)


p <- ggplot(pdata_filt) + geom_boxplot(aes(x=Donor.ID3, y=shannon_diversity, fill=Donor.ID3),
                                  outlier.shape=1, outlier.size=4)
p <- p + ggtitle("Alpha diversity")
p <- p + theme_bw(base_size=15)
p <- p + scale_fill_manual(values=c("firebrick4", "midnightblue", "goldenrod4"))
p <- p + xlab("SampleType") + ylab("Shannon Diversity")
#p <- p + annotate("segment", x=1.1, xend= 1.9, y=4, yend=4)
p <- p + annotate("segment", x=2.1, xend= 2.9, y=4, yend=4)
#p <- p + annotate("text", x = 1.5, y=4, label = "**", size=5)
p <- p + annotate("text", x = 2.5, y=4, label = "**", size=5)
p <- p + guides(colour=FALSE, size=FALSE, shape=FALSE, fill=FALSE)
p
```

```
#beta diversity
#bray curtis
phylo_abund_post <- subset_samples(phylo_abund, SampleType=="PostTransplant")
phylo_abund_post <- subset_samples(phylo_abund_post, Donor.ID!=21)

braydist_post = phyloseq::distance(phylo_abund_post, method = "bray")
meta_braydist_post <- as.data.frame(sample_data(phylo_abund_post))

adonis(braydist_post ~ as.factor(meta_braydist_post$Donor.ID), permutations = 4999)
```

```
## 
## Call:
## adonis(formula = braydist_post ~ as.factor(meta_braydist_post$Donor.ID),      permutations = 4999) 
## 
## Permutation: free
## Number of permutations: 4999
## 
## Terms added sequentially (first to last)
## 
##                                        Df SumsOfSqs MeanSqs F.Model      R2
## as.factor(meta_braydist_post$Donor.ID)  2    1.5010 0.75048  2.7059 0.14466
## Residuals                              32    8.8752 0.27735         0.85534
## Total                                  34   10.3762                 1.00000
##                                        Pr(>F)    
## as.factor(meta_braydist_post$Donor.ID)  4e-04 ***
## Residuals                                        
## Total                                            
## ---
## Signif. codes:  0 '***' 0.001 '**' 0.01 '*' 0.05 '.' 0.1 ' ' 1
```

```
#ordinations. make a PCoA plot.
expt2.ord <- ordinate(phylo_abund_post, "PCoA", "bray")

#PCoA Bray-Curtis

p1 = plot_ordination(phylo_abund_post, expt2.ord, type = "samples", title = "PCoA (Bray-Curtis)")
p1 <- p1 +  geom_point(aes(colour=as.factor(Donor.ID)), size=4)
p1 <- p1 + scale_colour_manual(values=c("firebrick4", "midnightblue", "goldenrod4"))
p1 <- p1 + theme_bw(base_size=16)
p1 <- p1 + annotate("text", x = -0.25, y=0.25, label = "p=0.0002", size=4 )
p1
```

# IgA Index PCA & PERMANOVA

Full dataset PCA and PERMANOVA (ASV Index)

```
metaIgA <- as.data.frame(as.matrix(metaIgA)) #for errors that it is still a "sample_data" object
metaIgA$SampleID <- gsub("-", ".", metaIgA$SampleID, fixed=TRUE)
row.names(IgA_settrim75_t)==metaIgA$SampleID
```

```
##  [1] TRUE TRUE TRUE TRUE TRUE TRUE TRUE TRUE TRUE TRUE TRUE TRUE TRUE TRUE TRUE
## [16] TRUE TRUE TRUE TRUE TRUE TRUE TRUE TRUE TRUE TRUE TRUE TRUE TRUE TRUE TRUE
## [31] TRUE TRUE TRUE TRUE TRUE TRUE TRUE TRUE TRUE TRUE TRUE TRUE TRUE TRUE TRUE
## [46] TRUE TRUE TRUE TRUE TRUE TRUE TRUE TRUE TRUE TRUE TRUE TRUE TRUE TRUE TRUE
## [61] TRUE TRUE TRUE TRUE TRUE TRUE TRUE TRUE TRUE TRUE TRUE TRUE TRUE TRUE TRUE
## [76] TRUE TRUE
```

```
metaIgA$Donor.ID2 <- paste("Donor", metaIgA$Donor, sep="")

iga.pca <- prcomp(IgA_settrim75_t)

metaIgA$SampleType <- factor(metaIgA$SampleType, levels=c("Donor", "PreTransplant", "PostTransplant"))
metaIgA$SampleType2 <- metaIgA$SampleType
metaIgA$SampleType2 <- gsub("PreTransplant", "Pre", metaIgA$SampleType2)
metaIgA$SampleType2 <- gsub("PostTransplant", "Post", metaIgA$SampleType2)
metaIgA$SampleType2 <- factor(metaIgA$SampleType2, 
                              levels=c("Donor", "Pre", "Post"))

#sample size by type
dplyr::count(metaIgA, SampleType)
```

```
##       SampleType  n
## 1          Donor  5
## 2  PreTransplant 36
## 3 PostTransplant 36
```

```
#adding sample sums / depth
#need to be careful about sample order hence the dataframe merge
sample_sums_pos <- data.frame(Stool_no=sample_data(IgPos_forindex)$Stool_no, 
                              sample_sums_pos=sample_data(IgPos_forindex)$sample_sums)
sample_sums_neg <- data.frame(Stool_no=sample_data(IgNeg_forindex)$Stool_no, 
                              sample_sums_neg=sample_data(IgNeg_forindex)$sample_sums)
sample_sums <- merge(sample_sums_pos, sample_sums_neg, by="Stool_no")
sample_sums$depth <- sample_sums$sample_sums_pos / sample_sums$sample_sums_neg
as.numeric(metaIgA$Stool_no)==sample_sums$Stool_no
```

```
##  [1] TRUE TRUE TRUE TRUE TRUE TRUE TRUE TRUE TRUE TRUE TRUE TRUE TRUE TRUE TRUE
## [16] TRUE TRUE TRUE TRUE TRUE TRUE TRUE TRUE TRUE TRUE TRUE TRUE TRUE TRUE TRUE
## [31] TRUE TRUE TRUE TRUE TRUE TRUE TRUE TRUE TRUE TRUE TRUE TRUE TRUE TRUE TRUE
## [46] TRUE TRUE TRUE TRUE TRUE TRUE TRUE TRUE TRUE TRUE TRUE TRUE TRUE TRUE TRUE
## [61] TRUE TRUE TRUE TRUE TRUE TRUE TRUE TRUE TRUE TRUE TRUE TRUE TRUE TRUE TRUE
## [76] TRUE TRUE
```

```
metaIgA$depth <-  sample_sums$depth

#simple PERMANOVA 
data_adonis <- data.frame(metaIgA, IgA_settrim75_t)

adonis.index <- adonis(IgA_settrim75_t ~ SampleType,
                       data = data_adonis, method='eu', sqrt.dist = FALSE)
adonis.index
```

```
## 
## Call:
## adonis(formula = IgA_settrim75_t ~ SampleType, data = data_adonis,      method = "eu", sqrt.dist = FALSE) 
## 
## Permutation: free
## Number of permutations: 999
## 
## Terms added sequentially (first to last)
## 
##            Df SumsOfSqs MeanSqs F.Model      R2 Pr(>F)    
## SampleType  2     18.17  9.0874  2.1628 0.05523  0.001 ***
## Residuals  74    310.92  4.2016         0.94477           
## Total      76    329.10                 1.00000           
## ---
## Signif. codes:  0 '***' 0.001 '**' 0.01 '*' 0.05 '.' 0.1 ' ' 1
```

```
p <- ggbiplot(iga.pca,ellipse=FALSE,var.axes=FALSE,  
              groups=metaIgA$SampleType2)
p <- p + geom_point(aes(colour=metaIgA$SampleType2), size=4)
p <- p + scale_color_manual(values=c("seagreen3", "indianred3", "deepskyblue3"))
p <- p + ggtitle("PCA of IgAIndex")
p <- p + theme_bw(base_size=15)
p <- p + annotate("text", x = 2, y=-3, label = "p=0.001", size=4 )
p <- p + xlab("PC1 (7.1%)") + ylab ("PC2 (5.8%)")
#p <- p + guides(colour=FALSE, size=FALSE, shape=FALSE, fill=FALSE)
p
```

```
#adding possible confounders
#remove Donors because I have no metadata about them
data_adonis_filt <- data_adonis[data_adonis$SampleType %in% c("PreTransplant", "PostTransplant"),]
IgA_settrim75_t_filt <- IgA_settrim75_t[row.names(IgA_settrim75_t)%in% data_adonis_filt$SampleID,]
row.names(IgA_settrim75_t_filt)==data_adonis_filt$SampleID
```

```
##  [1] TRUE TRUE TRUE TRUE TRUE TRUE TRUE TRUE TRUE TRUE TRUE TRUE TRUE TRUE TRUE
## [16] TRUE TRUE TRUE TRUE TRUE TRUE TRUE TRUE TRUE TRUE TRUE TRUE TRUE TRUE TRUE
## [31] TRUE TRUE TRUE TRUE TRUE TRUE TRUE TRUE TRUE TRUE TRUE TRUE TRUE TRUE TRUE
## [46] TRUE TRUE TRUE TRUE TRUE TRUE TRUE TRUE TRUE TRUE TRUE TRUE TRUE TRUE TRUE
## [61] TRUE TRUE TRUE TRUE TRUE TRUE TRUE TRUE TRUE TRUE TRUE TRUE
```

```
adonis.index2 <- adonis(IgA_settrim75_t_filt ~ Sort_Day + depth + 
                          Gender + Donor.ID2 + Percent_IgA +
                          SampleType,
                       data = data_adonis_filt, method='eu', sqrt.dist = FALSE)
adonis.index2
```

```
## 
## Call:
## adonis(formula = IgA_settrim75_t_filt ~ Sort_Day + depth + Gender +      Donor.ID2 + Percent_IgA + SampleType, data = data_adonis_filt,      method = "eu", sqrt.dist = FALSE) 
## 
## Permutation: free
## Number of permutations: 999
## 
## Terms added sequentially (first to last)
## 
##             Df SumsOfSqs MeanSqs F.Model      R2 Pr(>F)
## Sort_Day     2     7.293       4       0 0.02491      1
## depth        1     6.656       7       0 0.02273      1
## Gender       1     5.330       5       0 0.01820      1
## Donor.ID2    3    13.979       5       0 0.04775      1
## Percent_IgA 64   259.530       4       0 0.88641      1
## Residuals    0     0.000     Inf         0.00000       
## Total       71   292.788                 1.00000
```

```
#looping through all variables
metaIgA_reduced <- metaIgA %>% 
  select(depth, Sort_Day, SampleType, Donor.ID2, Delivery, Gender, Age_at_FMT, Percent_IgA,
         IBD, BMI_Pre.CDI, Wt_Loss,
         Serum_Total..without.valerate., #SCFA totals
         Fe_.ug.ml., Mg_.ug.ml.,  Zn_.ug.ml., #most abundant metals
         LB, HB #low branching and high branching serum glycans
         )

dim(metaIgA_reduced)
```

```
## [1] 77 17
```

```
dim(IgA_settrim75_t)
```

```
## [1]  77 197
```

```
#new issue that all of the numeric values have been converted to characters
metaIgA_reduced$depth <- as.numeric(metaIgA_reduced$depth)
metaIgA_reduced$Age_at_FMT <- as.numeric(metaIgA_reduced$Age_at_FMT)
metaIgA_reduced$Percent_IgA <- as.numeric(metaIgA_reduced$Percent_IgA)
metaIgA_reduced$BMI_Pre.CDI <- as.numeric(metaIgA_reduced$BMI_Pre.CDI)
metaIgA_reduced$Wt_Loss <- as.numeric(metaIgA_reduced$Wt_Loss)
metaIgA_reduced$Serum_Total..without.valerate. <- as.numeric(metaIgA_reduced$Serum_Total..without.valerate.)
metaIgA_reduced$Fe_.ug.ml. <- as.numeric(metaIgA_reduced$Fe_.ug.ml.)
metaIgA_reduced$Mg_.ug.ml. <- as.numeric(metaIgA_reduced$Mg_.ug.ml.)
metaIgA_reduced$Zn_.ug.ml. <- as.numeric(metaIgA_reduced$Zn_.ug.ml.)
metaIgA_reduced$LB <- as.numeric(metaIgA_reduced$LB)
metaIgA_reduced$HB <- as.numeric(metaIgA_reduced$HB)

#loop through a permanova of each factor
#although batch was not sig - try constraining permutations by sorting batch effect just in case
permanova.results <- data.frame(Variable=character(),
                                p.val=numeric(), 
                                coef=numeric(), 
                                stringsAsFactors=FALSE) 

set.seed(92483)
for(i in 1:17) {
  meta_filt <- metaIgA_reduced[,i]
  df <- data.frame(meta_filt, IgA_settrim75_t)
  df_filt <- na.omit(df)
  y <- adonis(df_filt[,3:198] ~ df_filt[,1], data = df_filt, method='eu', sqrt.dist = FALSE)
  permanova.results[i,1] <- names(metaIgA_reduced)[i]
  permanova.results[i,2] <- y$aov.tab[1,6]
  permanova.results[i,3] <- y$aov.tab[1,5]
}

#after looping through all variables
permanova.results$fdr <- p.adjust(permanova.results$p.val, method="fdr")
permanova.results$subset <- c("All (N=77)")
permanova.results[which(permanova.results$fdr<0.05),]
```

```
##                          Variable p.val       coef   fdr     subset
## 3                      SampleType 0.001 0.05491790 0.017 All (N=77)
## 12 Serum_Total..without.valerate. 0.002 0.04665563 0.017 All (N=77)
```

```
#strata by sorting batch
permanova.results.strata <- data.frame(Variable=character(),
                                p.val=numeric(), 
                                coef=numeric(), 
                                stringsAsFactors=FALSE) 
for(i in 1:17) {
  meta_filt <- metaIgA_reduced[,i]
  df <- data.frame(meta_filt, metaIgA_reduced$Sort_Day, IgA_settrim75_t)
  df_filt <- na.omit(df)
  y <- adonis(df_filt[,3:199] ~ df_filt[,1], strata=df_filt[,2], data = df_filt, method='eu', sqrt.dist = FALSE)
  permanova.results.strata[i,1] <- names(metaIgA_reduced)[i]
  permanova.results.strata[i,2] <- y$aov.tab[1,6]
  permanova.results.strata[i,3] <- y$aov.tab[1,5]
}

#after looping through all variables
permanova.results.strata$fdr <- p.adjust(permanova.results.strata$p.val, method="fdr")
permanova.results.strata$subset <- c("All (N=77)")
permanova.results.strata[which(permanova.results.strata$fdr<0.05),]
```

```
##                          Variable p.val       coef        fdr     subset
## 3                      SampleType 0.001 0.05522642 0.01700000 All (N=77)
## 4                       Donor.ID2 0.004 0.06816971 0.02833333 All (N=77)
## 12 Serum_Total..without.valerate. 0.005 0.04657599 0.02833333 All (N=77)
```

Pre-transplant PCA and PERMANOVA (ASV Index)

```
iga.pca <- prcomp(IgAPre_trim75_t)
row.names(IgAPre_trim75_t)==metaIgAPre$Patient
```

```
##  [1] TRUE TRUE TRUE TRUE TRUE TRUE TRUE TRUE TRUE TRUE TRUE TRUE TRUE TRUE TRUE
## [16] TRUE TRUE TRUE TRUE TRUE TRUE TRUE TRUE TRUE TRUE TRUE TRUE TRUE TRUE TRUE
## [31] TRUE TRUE TRUE TRUE TRUE TRUE
```

```
metaIgAPre$Donor.ID2 <- paste("Donor", metaIgAPre$Donor, sep="")
metaIgAPre <- as.data.frame(as.matrix(metaIgAPre))

# plot
p <- ggbiplot(iga.pca,ellipse=FALSE,var.axes=FALSE,  
              groups=metaIgAPre$Donor.ID2)
p <- p + geom_point(aes(colour=metaIgAPre$Donor.ID2), size=4)
p <- p + scale_color_manual(values=c("lightskyblue1", "deepskyblue", "dodgerblue3", "plum4"))
p <- p + ggtitle("PCA of IgAIndex PreTransplant")
p <- p + theme_bw(base_size=16)
p
```

```
#also adding sample sums
sample_sums_pos <- data.frame(Stool_no=sample_data(IgPos_forindexPre)$Stool_no, 
                              sample_sums_pos=sample_data(IgPos_forindexPre)$sample_sums)
sample_sums_neg <- data.frame(Stool_no=sample_data(IgNeg_forindexPre)$Stool_no, 
                              sample_sums_neg=sample_data(IgNeg_forindexPre)$sample_sums)
sample_sums_pre <- merge(sample_sums_pos, sample_sums_neg, by="Stool_no")
sample_sums_pre$depth <- sample_sums_pre$sample_sums_pos / sample_sums_pre$sample_sums_neg
as.numeric(metaIgAPre$Stool_no)==sample_sums_pre$Stool_no
```

```
##  [1] TRUE TRUE TRUE TRUE TRUE TRUE TRUE TRUE TRUE TRUE TRUE TRUE TRUE TRUE TRUE
## [16] TRUE TRUE TRUE TRUE TRUE TRUE TRUE TRUE TRUE TRUE TRUE TRUE TRUE TRUE TRUE
## [31] TRUE TRUE TRUE TRUE TRUE TRUE
```

```
metaIgAPre$depth <-  sample_sums_pre$depth

#gather dataframe for adonis
row.names(IgAPre_trim75_t)==metaIgAPre$Patient
```

```
##  [1] TRUE TRUE TRUE TRUE TRUE TRUE TRUE TRUE TRUE TRUE TRUE TRUE TRUE TRUE TRUE
## [16] TRUE TRUE TRUE TRUE TRUE TRUE TRUE TRUE TRUE TRUE TRUE TRUE TRUE TRUE TRUE
## [31] TRUE TRUE TRUE TRUE TRUE TRUE
```

```
data_adonis <- data.frame(IgAPre_trim75_t, metaIgAPre)

#simple permanova
adonis.index <- adonis(IgAPre_trim75_t ~ Donor.ID2,
                       data = data_adonis, method='eu', sqrt.dist = FALSE)
adonis.index
```

```
## 
## Call:
## adonis(formula = IgAPre_trim75_t ~ Donor.ID2, data = data_adonis,      method = "eu", sqrt.dist = FALSE) 
## 
## Permutation: free
## Number of permutations: 999
## 
## Terms added sequentially (first to last)
## 
##           Df SumsOfSqs MeanSqs F.Model      R2 Pr(>F)
## Donor.ID2  3     7.622  2.5406  1.2171 0.10242  0.186
## Residuals 32    66.794  2.0873         0.89758       
## Total     35    74.415                 1.00000
```

```
#possible confounders #note - missing data points for age
adonis.index2 <- adonis(IgAPre_trim75_t ~ depth + Sort_Day  + Gender + Percent_IgA + Donor.ID2,
                       data = data_adonis, method='eu', sqrt.dist = FALSE)
adonis.index2
```

```
## 
## Call:
## adonis(formula = IgAPre_trim75_t ~ depth + Sort_Day + Gender +      Percent_IgA + Donor.ID2, data = data_adonis, method = "eu",      sqrt.dist = FALSE) 
## 
## Permutation: free
## Number of permutations: 999
## 
## Terms added sequentially (first to last)
## 
##             Df SumsOfSqs MeanSqs F.Model      R2 Pr(>F)
## depth        1     2.496       2       0 0.03354      1
## Sort_Day     2     3.196       2       0 0.04295      1
## Gender       1     2.067       2       0 0.02778      1
## Percent_IgA 31    66.656       2       0 0.89573      1
## Residuals    0     0.000     Inf         0.00000       
## Total       35    74.415                 1.00000
```

```
#looping through all possible variables
metaIgAPre_reduced <- metaIgAPre %>% 
  select(depth, Sort_Day, Donor.ID2, Delivery, Gender, Age_at_FMT, Percent_IgA,
         IBD, BMI_Pre.CDI, Wt_Loss,
         Serum_Total..without.valerate., #SCFA totals
         Fe_.ug.ml., Mg_.ug.ml.,  Zn_.ug.ml., #most abundant metals
         LB, HB #low branching and high branching serum glycans
         )

dim(metaIgAPre_reduced)
```

```
## [1] 36 16
```

```
dim(IgAPre_trim75_t)
```

```
## [1] 36 42
```

```
#new issue that all of the numeric values have been converted to characters
metaIgAPre_reduced$depth <- as.numeric(metaIgAPre_reduced$depth)
metaIgAPre_reduced$Age_at_FMT <- as.numeric(metaIgAPre_reduced$Age_at_FMT)
metaIgAPre_reduced$Percent_IgA <- as.numeric(metaIgAPre_reduced$Percent_IgA)
metaIgAPre_reduced$BMI_Pre.CDI <- as.numeric(metaIgAPre_reduced$BMI_Pre.CDI)
metaIgAPre_reduced$Wt_Loss <- as.numeric(metaIgAPre_reduced$Wt_Loss)
metaIgAPre_reduced$Serum_Total..without.valerate. <- as.numeric(metaIgAPre_reduced$Serum_Total..without.valerate.)
metaIgAPre_reduced$Fe_.ug.ml. <- as.numeric(metaIgAPre_reduced$Fe_.ug.ml.)
metaIgAPre_reduced$Mg_.ug.ml. <- as.numeric(metaIgAPre_reduced$Mg_.ug.ml.)
metaIgAPre_reduced$Zn_.ug.ml. <- as.numeric(metaIgAPre_reduced$Zn_.ug.ml.)
metaIgAPre_reduced$LB <- as.numeric(metaIgAPre_reduced$LB)
metaIgAPre_reduced$HB <- as.numeric(metaIgAPre_reduced$HB)

permanova.results.pre <- data.frame(Variable=character(),
                                p.val=numeric(), 
                                coef=numeric(), 
                                stringsAsFactors=FALSE) 

set.seed(92483)
for(i in 1:16) {
  meta_filt <- metaIgAPre_reduced[,i]
  df <- data.frame(meta_filt, IgAPre_trim75_t)
  df_filt <- na.omit(df)
  y <- adonis(df_filt[,3:43] ~ df_filt[,1], data = df_filt, method='eu', sqrt.dist = FALSE)
  permanova.results.pre[i,1] <- names(metaIgAPre_reduced)[i]
  permanova.results.pre[i,2] <- y$aov.tab[1,6]
  permanova.results.pre[i,3] <- y$aov.tab[1,5]
}

#after looping through all variables
permanova.results.pre$fdr <- p.adjust(permanova.results.pre$p.val, method="fdr")
permanova.results.pre$subset <- c("Pre (N=36)")
permanova.results.pre[which(permanova.results.pre$fdr<0.05),]
```

```
## [1] Variable p.val    coef     fdr      subset  
## <0 rows> (or 0-length row.names)
```

```
#strata by sorting batch
permanova.results.pre.strata <- data.frame(Variable=character(),
                                p.val=numeric(), 
                                coef=numeric(), 
                                stringsAsFactors=FALSE) 

for(i in 1:16) {
  meta_filt <- metaIgAPre_reduced[,i]
  df <- data.frame(meta_filt,metaIgAPre_reduced$Sort_Day, IgAPre_trim75_t)
  df_filt <- na.omit(df)
  y <- adonis(df_filt[,3:44] ~ df_filt[,1], strata=df_filt[,2], data = df_filt, method='eu', sqrt.dist = FALSE)
  permanova.results.pre.strata[i,1] <- names(metaIgAPre_reduced)[i]
  permanova.results.pre.strata[i,2] <- y$aov.tab[1,6]
  permanova.results.pre.strata[i,3] <- y$aov.tab[1,5]
}

#after looping through all variables
permanova.results.pre.strata$fdr <- p.adjust(permanova.results.pre.strata$p.val, method="fdr")
permanova.results.pre.strata$subset <- c("Pre (N=36)")
permanova.results.pre.strata[which(permanova.results.pre.strata$fdr<0.05),]
```

```
## [1] Variable p.val    coef     fdr      subset  
## <0 rows> (or 0-length row.names)
```

Post-transplant PCA and PERMANOVA (ASV Index)

```
#IgAPost_trim75_t <- IgAPost_trim75_t[order(row.names(IgAPost_trim75_t)),]
#metaIgAPost <- metaIgAPost[order(metaIgAPost$Patient),]
row.names(IgAPost_trim75_t)==metaIgAPost$Patient
```

```
##  [1] TRUE TRUE TRUE TRUE TRUE TRUE TRUE TRUE TRUE TRUE TRUE TRUE TRUE TRUE TRUE
## [16] TRUE TRUE TRUE TRUE TRUE TRUE TRUE TRUE TRUE TRUE TRUE TRUE TRUE TRUE TRUE
## [31] TRUE TRUE TRUE TRUE TRUE TRUE
```

```
metaIgAPost$Donor <- as.factor(metaIgAPost$Donor)
metaIgAPost$Donor.ID2 <- paste("Donor", metaIgAPost$Donor, sep="")
metaIgAPost$Donor.ID2 <- factor(metaIgAPost$Donor.ID2, 
                                levels=c("Donor2", "Donor7", "Donor20", "Donor21"))
metaIgAPost_filt <- metaIgAPost[which(metaIgAPost$Donor.ID2!="Donor21"),]
#metaIgAPost_filt$Donor.ID3 <- metaIgAPost_filt$Donor.ID2
#metaIgAPost_filt$Donor.ID3 <- gsub("Donor20", "Donor3", metaIgAPost_filt$Donor.ID3)
#metaIgAPost_filt$Donor.ID3 <- gsub("Donor2", "Donor1", metaIgAPost_filt$Donor.ID3)
#metaIgAPost_filt$Donor.ID3 <- gsub("Donor7", "Donor2", metaIgAPost_filt$Donor.ID3)

metaIgAPost_filt$Sex <- metaIgAPost_filt$Gender
metaIgAPost_filt <- as.data.frame(as.matrix(metaIgAPost_filt))

iga.pca <- prcomp(IgAPost_trim75_t)

IgAPost_trim75_t_nodonor21 <- IgAPost_trim75_t[which(row.names(IgAPost_trim75_t)%in%metaIgAPost_filt$Patient),]

iga.pca2 <- prcomp(IgAPost_trim75_t_nodonor21)

row.names(IgAPost_trim75_t_nodonor21)==metaIgAPost_filt$Patient
```

```
##  [1] TRUE TRUE TRUE TRUE TRUE TRUE TRUE TRUE TRUE TRUE TRUE TRUE TRUE TRUE TRUE
## [16] TRUE TRUE TRUE TRUE TRUE TRUE TRUE TRUE TRUE TRUE TRUE TRUE TRUE TRUE TRUE
## [31] TRUE TRUE TRUE TRUE TRUE
```

```
data_adonis_nodonor21 <- data.frame(IgAPost_trim75_t_nodonor21, metaIgAPost_filt)

adonis.index.no21 <- adonis(IgAPost_trim75_t_nodonor21 ~ Donor.ID3,
                       data = data_adonis_nodonor21, method='eu', sqrt.dist = FALSE)
adonis.index.no21
```

```
## 
## Call:
## adonis(formula = IgAPost_trim75_t_nodonor21 ~ Donor.ID3, data = data_adonis_nodonor21,      method = "eu", sqrt.dist = FALSE) 
## 
## Permutation: free
## Number of permutations: 999
## 
## Terms added sequentially (first to last)
## 
##           Df SumsOfSqs MeanSqs F.Model      R2 Pr(>F)  
## Donor.ID3  2    13.549  6.7745  1.2444 0.07216  0.011 *
## Residuals 32   174.214  5.4442         0.92784         
## Total     34   187.763                 1.00000         
## ---
## Signif. codes:  0 '***' 0.001 '**' 0.01 '*' 0.05 '.' 0.1 ' ' 1
```

```
adonis.index.no21 <- adonis(IgAPost_trim75_t_nodonor21 ~ Sex,
                       data = data_adonis_nodonor21, method='eu', sqrt.dist = FALSE)
adonis.index.no21
```

```
## 
## Call:
## adonis(formula = IgAPost_trim75_t_nodonor21 ~ Sex, data = data_adonis_nodonor21,      method = "eu", sqrt.dist = FALSE) 
## 
## Permutation: free
## Number of permutations: 999
## 
## Terms added sequentially (first to last)
## 
##           Df SumsOfSqs MeanSqs F.Model      R2 Pr(>F)   
## Sex        1     8.226  8.2263   1.512 0.04381  0.002 **
## Residuals 33   179.536  5.4405         0.95619          
## Total     34   187.763                 1.00000          
## ---
## Signif. codes:  0 '***' 0.001 '**' 0.01 '*' 0.05 '.' 0.1 ' ' 1
```

```
# plot
p <- ggbiplot(iga.pca2,ellipse=FALSE,var.axes=FALSE,  
              groups=metaIgAPost_filt$Donor.ID3)
p <- p + geom_point(aes(colour=metaIgAPost_filt$Donor.ID3), size=4)
p <- p + scale_colour_manual(values=c("firebrick4", "midnightblue", "goldenrod4"))
p <- p + ggtitle("PCA of IgAIndex (Post)")
p <- p + labs(colour="Donor")
p <- p + xlab("PC1 (8.5%)") + ylab("PC2 (7.3%)")
p <- p + annotate("text", x = 1, y=-3, label = "p=0.01", size=4 )
p <- p + theme_bw(base_size=16)
p
```

```
p <- ggbiplot(iga.pca2,ellipse=FALSE,var.axes=FALSE,  
              groups=metaIgAPost_filt$Donor.ID3)
p <- p + geom_point(aes(colour=metaIgAPost_filt$Donor.ID3, shape=metaIgAPost_filt$Sex), size=4)
p <- p + scale_colour_manual(values=c("firebrick4", "midnightblue", "goldenrod4"))
p <- p + ggtitle("PCA of IgAIndex (Post)")
p <- p + labs(shape="Sex", colour="Donor")
p <- p + xlab("PC1 (8.5%)") + ylab("PC2 (7.3%)")
p <- p + annotate("text", x = 0, y=-2.5, label = "Donor: p=0.01", size=4 )
p <- p + annotate("text", x = 0, y=-3, label = "Sex: p=0.003", size=4 )
p <- p + theme_bw(base_size=16)
p
```

```
# plot 2
p <- ggbiplot(iga.pca,ellipse=FALSE,var.axes=FALSE,  
              groups=metaIgAPost$Delivery)
p <- p + geom_point(aes(colour=metaIgAPost$Delivery), size=4)
p <- p + scale_colour_manual(values=c("grey", "black"))
p <- p + ggtitle("PCA of IgAIndex (Post)")
p <- p + theme_bw(base_size=15)
p <- p + xlab("PC1 (8.4%)") + ylab("PC2 (7.2%)")
p
```

```
#also adding sample sums
sample_sums_pos <- data.frame(Stool_no=sample_data(IgPos_forindexPost)$Stool_no, 
                              sample_sums_pos=sample_data(IgPos_forindexPost)$sample_sums)
sample_sums_neg <- data.frame(Stool_no=sample_data(IgNeg_forindexPost)$Stool_no, 
                              sample_sums_neg=sample_data(IgNeg_forindexPost)$sample_sums)
sample_sums_post <- merge(sample_sums_pos, sample_sums_neg, by="Stool_no")
sample_sums_post$depth <- sample_sums_post$sample_sums_pos / sample_sums_post$sample_sums_neg
as.numeric(metaIgAPost$Stool_no)==sample_sums_post$Stool_no
```

```
##  [1] TRUE TRUE TRUE TRUE TRUE TRUE TRUE TRUE TRUE TRUE TRUE TRUE TRUE TRUE TRUE
## [16] TRUE TRUE TRUE TRUE TRUE TRUE TRUE TRUE TRUE TRUE TRUE TRUE TRUE TRUE TRUE
## [31] TRUE TRUE TRUE TRUE TRUE TRUE
```

```
metaIgAPost$depth <-  sample_sums_post$depth

#gather dataframe for adonis adonis
row.names(IgAPost_trim75_t)==metaIgAPost$Patient
```

```
##  [1] TRUE TRUE TRUE TRUE TRUE TRUE TRUE TRUE TRUE TRUE TRUE TRUE TRUE TRUE TRUE
## [16] TRUE TRUE TRUE TRUE TRUE TRUE TRUE TRUE TRUE TRUE TRUE TRUE TRUE TRUE TRUE
## [31] TRUE TRUE TRUE TRUE TRUE TRUE
```

```
data_adonis <- data.frame(IgAPost_trim75_t, metaIgAPost)

#simple permanova
adonis.index <- adonis(IgAPost_trim75_t ~ Donor.ID2,
                       data = data_adonis, method='eu', sqrt.dist = FALSE)
adonis.index
```

```
## 
## Call:
## adonis(formula = IgAPost_trim75_t ~ Donor.ID2, data = data_adonis,      method = "eu", sqrt.dist = FALSE) 
## 
## Permutation: free
## Number of permutations: 999
## 
## Terms added sequentially (first to last)
## 
##           Df SumsOfSqs MeanSqs F.Model      R2 Pr(>F)
## Donor.ID2  3    17.194  5.7314  1.0528 0.08983  0.301
## Residuals 32   174.214  5.4442         0.91017       
## Total     35   191.408                 1.00000
```

```
#possible confounders #note - missing data points for age
adonis.index2 <- adonis(IgAPost_trim75_t ~ depth + Sort_Day  + Gender 
                       + Percent_IgA + Donor.ID2,
                       data = data_adonis, method='eu', sqrt.dist = FALSE)
adonis.index2
```

```
## 
## Call:
## adonis(formula = IgAPost_trim75_t ~ depth + Sort_Day + Gender +      Percent_IgA + Donor.ID2, data = data_adonis, method = "eu",      sqrt.dist = FALSE) 
## 
## Permutation: free
## Number of permutations: 999
## 
## Terms added sequentially (first to last)
## 
##             Df SumsOfSqs MeanSqs F.Model      R2 Pr(>F)  
## depth        1     7.756  7.7560 1.45241 0.04052  0.039 *
## Sort_Day     2    10.508  5.2541 0.98388 0.05490  0.559  
## Gender       1     7.102  7.1015 1.32984 0.03710  0.018 *
## Percent_IgA  1     4.946  4.9456 0.92612 0.02584  0.615  
## Donor.ID2    3    16.913  5.6378 1.05574 0.08836  0.374  
## Residuals   27   144.183  5.3401         0.75328         
## Total       35   191.408                 1.00000         
## ---
## Signif. codes:  0 '***' 0.001 '**' 0.01 '*' 0.05 '.' 0.1 ' ' 1
```

```
#looping through all the variables
metaIgAPost <- as.data.frame(as.matrix(metaIgAPost))
metaIgAPost_reduced <- metaIgAPost %>% 
  select(depth, Sort_Day, Donor.ID2, Delivery, Gender, Age_at_FMT, Percent_IgA,
         IBD, BMI_Pre.CDI, Wt_Loss,
         Serum_Total..without.valerate., #SCFA totals
         Fe_.ug.ml., Mg_.ug.ml.,  Zn_.ug.ml., #most abundant metals
         LB, HB #low branching and high branching serum glycans
         )

dim(metaIgAPost_reduced)
```

```
## [1] 36 16
```

```
dim(IgAPost_trim75_t)
```

```
## [1]  36 180
```

```
#new issue that all of the numeric values have been converted to characters
metaIgAPost_reduced$depth <- as.numeric(metaIgAPost_reduced$depth)
metaIgAPost_reduced$Age_at_FMT <- as.numeric(metaIgAPost_reduced$Age_at_FMT)
metaIgAPost_reduced$Percent_IgA <- as.numeric(metaIgAPost_reduced$Percent_IgA)
metaIgAPost_reduced$BMI_Pre.CDI <- as.numeric(metaIgAPost_reduced$BMI_Pre.CDI)
metaIgAPost_reduced$Wt_Loss <- as.numeric(metaIgAPost_reduced$Wt_Loss)
metaIgAPost_reduced$Serum_Total..without.valerate. <- as.numeric(metaIgAPost_reduced$Serum_Total..without.valerate.)
metaIgAPost_reduced$Fe_.ug.ml. <- as.numeric(metaIgAPost_reduced$Fe_.ug.ml.)
metaIgAPost_reduced$Mg_.ug.ml. <- as.numeric(metaIgAPost_reduced$Mg_.ug.ml.)
metaIgAPost_reduced$Zn_.ug.ml. <- as.numeric(metaIgAPost_reduced$Zn_.ug.ml.)
metaIgAPost_reduced$LB <- as.numeric(metaIgAPost_reduced$LB)
metaIgAPost_reduced$HB <- as.numeric(metaIgAPost_reduced$HB)

permanova.results.post <- data.frame(Variable=character(),
                                    p.val=numeric(), 
                                    coef=numeric(), 
                                    stringsAsFactors=FALSE) 

set.seed(92483)
for(i in 1:16) {
  meta_filt <- metaIgAPost_reduced[,i]
  df <- data.frame(meta_filt, IgAPost_trim75_t)
  df_filt <- na.omit(df)
  y <- adonis(df_filt[,3:181] ~ df_filt[,1], data = df_filt, method='eu', sqrt.dist = FALSE)
  permanova.results.post[i,1] <- names(metaIgAPost_reduced)[i]
  permanova.results.post[i,2] <- y$aov.tab[1,6]
  permanova.results.post[i,3] <- y$aov.tab[1,5]
}

#after looping through all variables
permanova.results.post$fdr <- p.adjust(permanova.results.post$p.val, method="fdr")
permanova.results.post$subset <- c("Post (N=36)")
permanova.results.post[which(permanova.results.post$fdr<0.05),]
```

```
## [1] Variable p.val    coef     fdr      subset  
## <0 rows> (or 0-length row.names)
```

```
#strata by sorting batch
permanova.results.post.strata <- data.frame(Variable=character(),
                                    p.val=numeric(), 
                                    coef=numeric(), 
                                    stringsAsFactors=FALSE) 

for(i in 1:16) {
  meta_filt <- metaIgAPost_reduced[,i]
  df <- data.frame(meta_filt, metaIgAPost$Sort_Day, IgAPost_trim75_t)
  df_filt <- na.omit(df)
  y <- adonis(df_filt[,3:182] ~ df_filt[,1], strata=df_filt[,2], data = df_filt, method='eu', sqrt.dist = FALSE)
  permanova.results.post.strata[i,1] <- names(metaIgAPost_reduced)[i]
  permanova.results.post.strata[i,2] <- y$aov.tab[1,6]
  permanova.results.post.strata[i,3] <- y$aov.tab[1,5]
}

#after looping through all variables
permanova.results.post.strata$fdr <- p.adjust(permanova.results.post.strata$p.val, method="fdr")
permanova.results.post.strata$subset <- c("Post (N=36)")
permanova.results.post.strata[which(permanova.results.post.strata$fdr<0.05),]
```

```
## [1] Variable p.val    coef     fdr      subset  
## <0 rows> (or 0-length row.names)
```

Plot combined results of PERMANOVA (ASV Index)

```
#combined
permanova.results.combined <- Reduce(full_join, list(permanova.results.strata, permanova.results.pre.strata, permanova.results.post.strata))
```

```
## Joining, by = c("Variable", "p.val", "coef", "fdr", "subset")
## Joining, by = c("Variable", "p.val", "coef", "fdr", "subset")
```

```
#rename variables to clean up the plot
permanova.results.combined$Variable <- gsub("Wt_Loss", "Weight_loss",
                                            permanova.results.combined$Variable)
permanova.results.combined$Variable <- gsub("Fe_.ug.ml.", "Iron",
                                            permanova.results.combined$Variable)
permanova.results.combined$Variable <- gsub("Zn_.ug.ml.", "Zinc",
                                            permanova.results.combined$Variable)
permanova.results.combined$Variable <- gsub("Mg_.ug.ml.", "Magnesium",
                                            permanova.results.combined$Variable)
permanova.results.combined$Variable <- gsub("Serum_Total..without.valerate.", "Serum_Total.SCFAs",
                                            permanova.results.combined$Variable)
permanova.results.combined$Variable <- gsub("LB", "Low-branching",
                                            permanova.results.combined$Variable)
permanova.results.combined$Variable <- gsub("HB", "High-branching",
                                            permanova.results.combined$Variable)
permanova.results.combined$Variable <- gsub("Donor.ID2", "Donor",
                                            permanova.results.combined$Variable)
permanova.results.combined$Variable <- gsub("Gender", "Sex",
                                            permanova.results.combined$Variable)

permanova.results.combined$Variable <- factor(permanova.results.combined$Variable,
                                              levels=c("depth", "Sort_Day", #technical factors
                                                       "SampleType", "Donor", "Percent_IgA",
                                                       "Sex", "Age_at_FMT", "IBD", "Weight_loss", "Delivery", "BMI_Pre.CDI",#biological factors
                                                       "Serum_Total.SCFAs", #SCFA
                                                      
                                                       "Iron", "Zinc", "Magnesium", #serum metals
                                                       "Low-branching", "High-branching" #serum glycans
                                                       
                                                       ))

permanova.results.combined$subset <- factor(permanova.results.combined$subset,
                                            levels=c("All (N=77)", "Pre (N=36)", "Post (N=36)"))

#plot

p <- ggplot(permanova.results.combined, aes(x=Variable,y=coef, fill=p.val)) + geom_bar(stat="identity")
p <- p + ggtitle("PERMANOVA summary (IgA)")
p <- p + theme_bw(base_size = 14)
p <- p + scale_fill_gradient(low = "lightblue", high = "black")
p <- p + theme(axis.text.x = element_text(angle=90, hjust=1, size=10), plot.title = element_text(size = 12))
p <- p + facet_grid(.~subset, labeller=label_wrap_gen(width=2)) 
p <- p + coord_flip()
p
```

```
#significance
permanova.results.combined[which(permanova.results.combined$fdr<0.1),]
```

```
##             Variable p.val       coef        fdr      subset
## 1              depth 0.013 0.02174506 0.05525000  All (N=77)
## 3         SampleType 0.001 0.05522642 0.01700000  All (N=77)
## 4              Donor 0.004 0.06816971 0.02833333  All (N=77)
## 6                Sex 0.021 0.01913995 0.07140000  All (N=77)
## 12 Serum_Total.SCFAs 0.005 0.04657599 0.02833333  All (N=77)
## 38               Sex 0.006 0.04012491 0.09600000 Post (N=36)
```

```
permanova.results.combined[which(permanova.results.combined$fdr<0.05),]
```

```
##             Variable p.val       coef        fdr     subset
## 3         SampleType 0.001 0.05522642 0.01700000 All (N=77)
## 4              Donor 0.004 0.06816971 0.02833333 All (N=77)
## 12 Serum_Total.SCFAs 0.005 0.04657599 0.02833333 All (N=77)
```

# Most Targeted Taxa Pre- and Post-Transplant

Most targeted pre-transplant (ASV)

```
#Pre #using all taxa, for a combined plot
wilcox_pre <- IgA_settrim75_t[which(row.names(IgA_settrim75_t)%in%gsub("-", ".", metaIgAPre$SampleID, fixed=TRUE)),]
#apply wilcox test to test if Index is sig. diff. from 0
wilcox.data1 <- sapply(1:ncol(wilcox_pre), function(i){wilcox.test(wilcox_pre[,i], mu=0)$p.value})
wilcox.data1 <- as.data.frame(wilcox.data1)
rownames(wilcox.data1) <- colnames(wilcox_pre)
#Perform multiple comparison correction using a given method of choice
wilcox.data1$rel.fdr <- p.adjust(wilcox.data1$wilcox.data1, method="fdr")
wilcox.data1$bonferroni <- p.adjust(wilcox.data1$wilcox.data1, method="bonferroni")
#incorporate other metrics of data1 centre and dispersion
wilcox.data1$mean <- t(summarise_each(wilcox_pre,funs(mean)))
wilcox.data1$median <- t(summarise_each(wilcox_pre,funs(median)))
wilcox.data1$variance <- t(summarise_each(wilcox_pre,funs(var))) 
wilcox.data1$IQR <- t(summarise_each(wilcox_pre,funs(IQR)))
wilcox.data1$Taxa <- row.names(wilcox.data1)
wilcox.data1$Subset <- c("Pre")

#Pre #using the filtered subset, to determine 'most targeted' pre-transplant
#apply wilcox test to test if Index is sig. diff. from 0
wilcox.data1.sub <- sapply(1:ncol(IgAPre_trim75_t), function(i){wilcox.test(IgAPre_trim75_t[,i], mu=0)$p.value})
wilcox.data1.sub <- as.data.frame(wilcox.data1.sub)
rownames(wilcox.data1.sub) <- colnames(IgAPre_trim75_t)
#Perform multiple comparison correction using a given method of choice
wilcox.data1.sub$rel.fdr <- p.adjust(wilcox.data1.sub$wilcox.data1.sub, method="fdr")
wilcox.data1.sub$bonferroni <- p.adjust(wilcox.data1.sub$wilcox.data1.sub, method="bonferroni")
#incorporate other metrics of data1 centre and dispersion
wilcox.data1.sub$mean <- t(summarise_each(IgAPre_trim75_t,funs(mean)))
wilcox.data1.sub$median <- t(summarise_each(IgAPre_trim75_t,funs(median)))
wilcox.data1.sub$variance <- t(summarise_each(IgAPre_trim75_t,funs(var))) 
wilcox.data1.sub$IQR <- t(summarise_each(IgAPre_trim75_t,funs(IQR)))
wilcox.data1.sub$Taxa <- row.names(wilcox.data1.sub)
wilcox.data1.sub$Subset <- c("Pre")

#Selecting the most targeted taxa; FDR<0.05, median index >0
most_targeted_pre <- dplyr::filter(wilcox.data1.sub, wilcox.data1.sub<0.05&median>0)
most_targeted_pre
```

```
##                                                                                                                                     wilcox.data1.sub
## |Bacteria|Proteobacteria|Gammaproteobacteria|Enterobacteriales|Enterobacteriaceae|Escherichia|coli|1b158b8b2922d4fcad5d9cea607cbb7d       0.00532425
##                                                                                                                                       rel.fdr
## |Bacteria|Proteobacteria|Gammaproteobacteria|Enterobacteriales|Enterobacteriaceae|Escherichia|coli|1b158b8b2922d4fcad5d9cea607cbb7d 0.1118092
##                                                                                                                                     bonferroni
## |Bacteria|Proteobacteria|Gammaproteobacteria|Enterobacteriales|Enterobacteriaceae|Escherichia|coli|1b158b8b2922d4fcad5d9cea607cbb7d  0.2236185
##                                                                                                                                          mean
## |Bacteria|Proteobacteria|Gammaproteobacteria|Enterobacteriales|Enterobacteriaceae|Escherichia|coli|1b158b8b2922d4fcad5d9cea607cbb7d 0.1731433
##                                                                                                                                        median
## |Bacteria|Proteobacteria|Gammaproteobacteria|Enterobacteriales|Enterobacteriaceae|Escherichia|coli|1b158b8b2922d4fcad5d9cea607cbb7d 0.2054731
##                                                                                                                                      variance
## |Bacteria|Proteobacteria|Gammaproteobacteria|Enterobacteriales|Enterobacteriaceae|Escherichia|coli|1b158b8b2922d4fcad5d9cea607cbb7d 0.1194509
##                                                                                                                                           IQR
## |Bacteria|Proteobacteria|Gammaproteobacteria|Enterobacteriales|Enterobacteriaceae|Escherichia|coli|1b158b8b2922d4fcad5d9cea607cbb7d 0.4276752
##                                                                                                                                                                                                                                                                    Taxa
## |Bacteria|Proteobacteria|Gammaproteobacteria|Enterobacteriales|Enterobacteriaceae|Escherichia|coli|1b158b8b2922d4fcad5d9cea607cbb7d |Bacteria|Proteobacteria|Gammaproteobacteria|Enterobacteriales|Enterobacteriaceae|Escherichia|coli|1b158b8b2922d4fcad5d9cea607cbb7d
##                                                                                                                                     Subset
## |Bacteria|Proteobacteria|Gammaproteobacteria|Enterobacteriales|Enterobacteriaceae|Escherichia|coli|1b158b8b2922d4fcad5d9cea607cbb7d    Pre
```

```
#plot most targeted taxa - pre
#collect IgA Index and taxa data into a data frame
targeted_index <- wilcox_pre[,which(names(wilcox_pre) %in% most_targeted_pre$Taxa)]
targeted_taxa <- IgTAX75[which(IgTAX75$combined %in% most_targeted_pre$Taxa),]
targeted_taxa$Simple <- paste(targeted_taxa$Genus, substr(targeted_taxa$ASV, start=0, stop=3))

pdata <- data.frame(IgAIndex=targeted_index, Taxa=targeted_taxa$Simple, Phylum=targeted_taxa$Phylum)

#pdata <- Reduce(full_join, list(pdata1, pdata2, pdata3, pdata4, pdata5))

#plot - most targeted pre
p <- ggplot(pdata) + geom_jitter(aes(x=Taxa, y=IgAIndex, color=Phylum), 
                                 width=0.2, height=0, shape=1, size=3)
p <- p + geom_boxplot(aes(x=Taxa, y=IgAIndex), color="black", fill=NA, outlier.shape=NA)
p <- p + ggtitle("Pre")
p <- p + theme_bw(base_size=16) 
p <- p + theme(axis.text.x = element_text(angle=45, hjust=1))
p <- p + ylim(c(-1, 1))
p <- p + scale_color_manual(values=c( "indianred3"))
p
```

```
#Selecting the least targeted taxa; FDR<0.05, median index >0
least_targeted_pre <- dplyr::filter(wilcox.data1.sub, wilcox.data1.sub<0.05&median<0)
least_targeted_pre
```

```
##                                                                                                                                    wilcox.data1.sub
## |Bacteria|Proteobacteria|Gammaproteobacteria|Xanthomonadales|Sinobacteraceae|NA|NA|09bedf1ee9478654b581fea611f919e7                      0.04433803
## |Bacteria|Proteobacteria|Gammaproteobacteria|Pseudomonadales|Pseudomonadaceae|NA|NA|1f878f615fcfc8d7bd381a7841ac1e41                     0.03676711
## |Bacteria|Proteobacteria|Gammaproteobacteria|Pseudomonadales|Pseudomonadaceae|Pseudomonas|veronii|402e5913597695a16d7cad415ffff02f       0.00136419
## |Bacteria|Firmicutes|Bacilli|Lactobacillales|Streptococcaceae|Streptococcus|NA|bd2ebc70501f7d867c204f94c4e483da                          0.01317533
##                                                                                                                                       rel.fdr
## |Bacteria|Proteobacteria|Gammaproteobacteria|Xanthomonadales|Sinobacteraceae|NA|NA|09bedf1ee9478654b581fea611f919e7                0.19459747
## |Bacteria|Proteobacteria|Gammaproteobacteria|Pseudomonadales|Pseudomonadaceae|NA|NA|1f878f615fcfc8d7bd381a7841ac1e41               0.19459747
## |Bacteria|Proteobacteria|Gammaproteobacteria|Pseudomonadales|Pseudomonadaceae|Pseudomonas|veronii|402e5913597695a16d7cad415ffff02f 0.05729598
## |Bacteria|Firmicutes|Bacilli|Lactobacillales|Streptococcaceae|Streptococcus|NA|bd2ebc70501f7d867c204f94c4e483da                    0.13834093
##                                                                                                                                    bonferroni
## |Bacteria|Proteobacteria|Gammaproteobacteria|Xanthomonadales|Sinobacteraceae|NA|NA|09bedf1ee9478654b581fea611f919e7                1.00000000
## |Bacteria|Proteobacteria|Gammaproteobacteria|Pseudomonadales|Pseudomonadaceae|NA|NA|1f878f615fcfc8d7bd381a7841ac1e41               1.00000000
## |Bacteria|Proteobacteria|Gammaproteobacteria|Pseudomonadales|Pseudomonadaceae|Pseudomonas|veronii|402e5913597695a16d7cad415ffff02f 0.05729598
## |Bacteria|Firmicutes|Bacilli|Lactobacillales|Streptococcaceae|Streptococcus|NA|bd2ebc70501f7d867c204f94c4e483da                    0.55336372
##                                                                                                                                           mean
## |Bacteria|Proteobacteria|Gammaproteobacteria|Xanthomonadales|Sinobacteraceae|NA|NA|09bedf1ee9478654b581fea611f919e7                -0.08988897
## |Bacteria|Proteobacteria|Gammaproteobacteria|Pseudomonadales|Pseudomonadaceae|NA|NA|1f878f615fcfc8d7bd381a7841ac1e41               -0.04158539
## |Bacteria|Proteobacteria|Gammaproteobacteria|Pseudomonadales|Pseudomonadaceae|Pseudomonas|veronii|402e5913597695a16d7cad415ffff02f -0.05419560
## |Bacteria|Firmicutes|Bacilli|Lactobacillales|Streptococcaceae|Streptococcus|NA|bd2ebc70501f7d867c204f94c4e483da                    -0.12031261
##                                                                                                                                         median
## |Bacteria|Proteobacteria|Gammaproteobacteria|Xanthomonadales|Sinobacteraceae|NA|NA|09bedf1ee9478654b581fea611f919e7                -0.02710518
## |Bacteria|Proteobacteria|Gammaproteobacteria|Pseudomonadales|Pseudomonadaceae|NA|NA|1f878f615fcfc8d7bd381a7841ac1e41               -0.03930549
## |Bacteria|Proteobacteria|Gammaproteobacteria|Pseudomonadales|Pseudomonadaceae|Pseudomonas|veronii|402e5913597695a16d7cad415ffff02f -0.04256908
## |Bacteria|Firmicutes|Bacilli|Lactobacillales|Streptococcaceae|Streptococcus|NA|bd2ebc70501f7d867c204f94c4e483da                    -0.11089487
##                                                                                                                                      variance
## |Bacteria|Proteobacteria|Gammaproteobacteria|Xanthomonadales|Sinobacteraceae|NA|NA|09bedf1ee9478654b581fea611f919e7                0.05696027
## |Bacteria|Proteobacteria|Gammaproteobacteria|Pseudomonadales|Pseudomonadaceae|NA|NA|1f878f615fcfc8d7bd381a7841ac1e41               0.01020758
## |Bacteria|Proteobacteria|Gammaproteobacteria|Pseudomonadales|Pseudomonadaceae|Pseudomonas|veronii|402e5913597695a16d7cad415ffff02f 0.00884602
## |Bacteria|Firmicutes|Bacilli|Lactobacillales|Streptococcaceae|Streptococcus|NA|bd2ebc70501f7d867c204f94c4e483da                    0.07121838
##                                                                                                                                           IQR
## |Bacteria|Proteobacteria|Gammaproteobacteria|Xanthomonadales|Sinobacteraceae|NA|NA|09bedf1ee9478654b581fea611f919e7                0.30856373
## |Bacteria|Proteobacteria|Gammaproteobacteria|Pseudomonadales|Pseudomonadaceae|NA|NA|1f878f615fcfc8d7bd381a7841ac1e41               0.14231289
## |Bacteria|Proteobacteria|Gammaproteobacteria|Pseudomonadales|Pseudomonadaceae|Pseudomonas|veronii|402e5913597695a16d7cad415ffff02f 0.09829887
## |Bacteria|Firmicutes|Bacilli|Lactobacillales|Streptococcaceae|Streptococcus|NA|bd2ebc70501f7d867c204f94c4e483da                    0.30155962
##                                                                                                                                                                                                                                                                  Taxa
## |Bacteria|Proteobacteria|Gammaproteobacteria|Xanthomonadales|Sinobacteraceae|NA|NA|09bedf1ee9478654b581fea611f919e7                               |Bacteria|Proteobacteria|Gammaproteobacteria|Xanthomonadales|Sinobacteraceae|NA|NA|09bedf1ee9478654b581fea611f919e7
## |Bacteria|Proteobacteria|Gammaproteobacteria|Pseudomonadales|Pseudomonadaceae|NA|NA|1f878f615fcfc8d7bd381a7841ac1e41                             |Bacteria|Proteobacteria|Gammaproteobacteria|Pseudomonadales|Pseudomonadaceae|NA|NA|1f878f615fcfc8d7bd381a7841ac1e41
## |Bacteria|Proteobacteria|Gammaproteobacteria|Pseudomonadales|Pseudomonadaceae|Pseudomonas|veronii|402e5913597695a16d7cad415ffff02f |Bacteria|Proteobacteria|Gammaproteobacteria|Pseudomonadales|Pseudomonadaceae|Pseudomonas|veronii|402e5913597695a16d7cad415ffff02f
## |Bacteria|Firmicutes|Bacilli|Lactobacillales|Streptococcaceae|Streptococcus|NA|bd2ebc70501f7d867c204f94c4e483da                                       |Bacteria|Firmicutes|Bacilli|Lactobacillales|Streptococcaceae|Streptococcus|NA|bd2ebc70501f7d867c204f94c4e483da
##                                                                                                                                    Subset
## |Bacteria|Proteobacteria|Gammaproteobacteria|Xanthomonadales|Sinobacteraceae|NA|NA|09bedf1ee9478654b581fea611f919e7                   Pre
## |Bacteria|Proteobacteria|Gammaproteobacteria|Pseudomonadales|Pseudomonadaceae|NA|NA|1f878f615fcfc8d7bd381a7841ac1e41                  Pre
## |Bacteria|Proteobacteria|Gammaproteobacteria|Pseudomonadales|Pseudomonadaceae|Pseudomonas|veronii|402e5913597695a16d7cad415ffff02f    Pre
## |Bacteria|Firmicutes|Bacilli|Lactobacillales|Streptococcaceae|Streptococcus|NA|bd2ebc70501f7d867c204f94c4e483da                       Pre
```

```
#plot least targeted taxa - pre
#collect IgA Index and taxa data into a data frame
targeted_index <- wilcox_pre[,which(names(wilcox_pre) %in% least_targeted_pre$Taxa)]
targeted_taxa <- IgTAX75[which(IgTAX75$combined %in% least_targeted_pre$Taxa),]
targeted_taxa$Simple <- paste(targeted_taxa$Genus, substr(targeted_taxa$ASV, start=0, stop=3))
targeted_taxa$Simple <- ifelse(is.na(targeted_taxa$Genus), yes= paste(targeted_taxa$Family, substr(targeted_taxa$ASV, start=0, stop=3)), no=targeted_taxa$Simple)

pdata1 <- data.frame(IgAIndex=targeted_index[,1], Taxa=targeted_taxa$Simple[1], Phylum=targeted_taxa$Phylum[1])
pdata2 <- data.frame(IgAIndex=targeted_index[,2], Taxa=targeted_taxa$Simple[2], Phylum=targeted_taxa$Phylum[2])
pdata3 <- data.frame(IgAIndex=targeted_index[,3], Taxa=targeted_taxa$Simple[3], Phylum=targeted_taxa$Phylum[3])
pdata4 <- data.frame(IgAIndex=targeted_index[,4], Taxa=targeted_taxa$Simple[4], Phylum=targeted_taxa$Phylum[4])

pdata <- Reduce(full_join, list(pdata1, pdata2, pdata3, pdata4))
```

```
## Joining, by = c("IgAIndex", "Taxa", "Phylum")
## Joining, by = c("IgAIndex", "Taxa", "Phylum")
## Joining, by = c("IgAIndex", "Taxa", "Phylum")
```

```
#plot - least targeted pre
p <- ggplot(pdata) + geom_jitter(aes(x=Taxa, y=IgAIndex, color=Phylum), 
                                 width=0.2, height=0, shape=1, size=3)
p <- p + geom_boxplot(aes(x=Taxa, y=IgAIndex), color="black", fill=NA, outlier.shape=NA)
p <- p + ggtitle("IgA-(Pre)")
p <- p + theme_bw(base_size=16) 
p <- p + theme(axis.text.x = element_text(angle=45, hjust=1, size=10))
p <- p + ylim(c(-1, 1))
p <- p + scale_color_manual(values=c("deepskyblue3", "indianred3"))
p
```

```
#also calculate averages for relative abundance (for bubble plot)
wilcox_preb <- Abund_settrim75_t[which(row.names(Abund_settrim75_t)%in%gsub("-", ".", metaIgAPre$SampleID, fixed=TRUE)),]

wilcox.data1b <- sapply(1:ncol(wilcox_preb), function(i){wilcox.test(wilcox_preb[,i], mu=0)$p.value})
wilcox.data1b <- as.data.frame(wilcox.data1b)
rownames(wilcox.data1b) <- colnames(wilcox_preb)
#Perform multiple comparison correction using a given method of choice
wilcox.data1b$rel.fdr <- p.adjust(wilcox.data1b$wilcox.data1b, method="fdr")
wilcox.data1b$bonferroni <- p.adjust(wilcox.data1b$wilcox.data1b, method="bonferroni")
#incorporate other metrics of data1 centre and dispersion
wilcox.data1b$mean <- t(summarise_each(wilcox_preb,funs(mean)))
wilcox.data1b$median <- t(summarise_each(wilcox_preb,funs(median)))
wilcox.data1b$variance <- t(summarise_each(wilcox_preb,funs(var))) 
wilcox.data1b$IQR <- t(summarise_each(wilcox_preb,funs(IQR)))
wilcox.data1b$Taxa <- row.names(wilcox.data1b)
wilcox.data1b$Subset <- c("Pre")
```

Most targeted post-transplant (ASV)

```
#using all taxa -for combined plot
wilcox_post <- IgA_settrim75_t[which(row.names(IgA_settrim75_t)%in%gsub("-", ".", metaIgAPost$SampleID, fixed=TRUE)),]
#apply wilcox test to test if Index is sig. diff. from 0
wilcox.data2 <- sapply(1:ncol(wilcox_post), function(i){wilcox.test(wilcox_post[,i], mu=0)$p.value})
wilcox.data2 <- as.data.frame(wilcox.data2)
rownames(wilcox.data2) <- colnames(wilcox_post)
#Perform multiple comparison correction using a given method of choice
wilcox.data2$rel.fdr <- p.adjust(wilcox.data2$wilcox.data2, method="fdr")
wilcox.data2$bonferroni <- p.adjust(wilcox.data2$wilcox.data2, method="bonferroni")
#incorporate other metrics of data2 centre and dispersion
wilcox.data2$mean <- t(summarise_each(wilcox_post,funs(mean)))
wilcox.data2$median <- t(summarise_each(wilcox_post,funs(median)))
wilcox.data2$variance <- t(summarise_each(wilcox_post,funs(var))) 
wilcox.data2$IQR <- t(summarise_each(wilcox_post,funs(IQR)))
wilcox.data2$Taxa <- row.names(wilcox.data2)
wilcox.data2$Subset <- c("Post")

#using filtered subset - to calculate most/least targeted
#apply wilcox test to test if Index is sig. diff. from 0
wilcox.data2.sub <- sapply(1:ncol(IgAPost_trim75_t), function(i){wilcox.test(IgAPost_trim75_t[,i], mu=0)$p.value})
wilcox.data2.sub <- as.data.frame(wilcox.data2.sub)
rownames(wilcox.data2.sub) <- colnames(IgAPost_trim75_t)
#Perform multiple comparison correction using a given method of choice
wilcox.data2.sub$rel.fdr <- p.adjust(wilcox.data2.sub$wilcox.data2.sub, method="fdr")
wilcox.data2.sub$bonferroni <- p.adjust(wilcox.data2.sub$wilcox.data2.sub, method="bonferroni")
#incorporate other metrics of data2 centre and dispersion
wilcox.data2.sub$mean <- t(summarise_each(IgAPost_trim75_t,funs(mean)))
wilcox.data2.sub$median <- t(summarise_each(IgAPost_trim75_t,funs(median)))
wilcox.data2.sub$variance <- t(summarise_each(IgAPost_trim75_t,funs(var))) 
wilcox.data2.sub$IQR <- t(summarise_each(IgAPost_trim75_t,funs(IQR)))
wilcox.data2.sub$Taxa <- row.names(wilcox.data2.sub)
wilcox.data2.sub$Subset <- c("Post")


#Selecting the most targeted taxa; p<0.05, median index >0
most_targeted_post <- dplyr::filter(wilcox.data2.sub, wilcox.data2.sub<0.05&median>0)
most_targeted_post$Taxa
```

```
## [1] "|Bacteria|Proteobacteria|Gammaproteobacteria|Enterobacteriales|Enterobacteriaceae|Escherichia|coli|1b158b8b2922d4fcad5d9cea607cbb7d"
## [2] "|Bacteria|Firmicutes|Bacilli|Lactobacillales|Streptococcaceae|Streptococcus|NA|73bf8d1a5983e34a0cb84e3cae127815"                    
## [3] "|Bacteria|Firmicutes|Clostridia|Clostridiales|Lachnospiraceae|Dorea|NA|a0b7d83fb64749f9a4b15b3728425b97"                            
## [4] "|Bacteria|Firmicutes|Clostridia|Clostridiales|Lachnospiraceae|[Ruminococcus]|gnavus|cc2d96099f530b503371e5ddca8c0a58"               
## [5] "|Bacteria|Firmicutes|Clostridia|Clostridiales|Lachnospiraceae|[Ruminococcus]|torques|e59405a47acbc248ce61395366159d8d"
```

```
#collect IgA Index and taxa data into a data frame
targeted_index <- IgAPost_trim75_t[,which(names(IgAPost_trim75_t) %in% most_targeted_post$Taxa)]
targeted_taxa <- IgTAX75[which(IgTAX75$combined %in% most_targeted_post$Taxa),]
targeted_taxa$Simple <- paste(targeted_taxa$Genus, substr(targeted_taxa$ASV, start=0, stop=3))


pdata1 <- data.frame(IgAIndex=targeted_index[,1], Taxa=targeted_taxa$Simple[1], Phylum=targeted_taxa$Phylum[1])
pdata2 <- data.frame(IgAIndex=targeted_index[,2], Taxa=targeted_taxa$Simple[2], Phylum=targeted_taxa$Phylum[2])
pdata3 <- data.frame(IgAIndex=targeted_index[,3], Taxa=targeted_taxa$Simple[3], Phylum=targeted_taxa$Phylum[3])
pdata4 <- data.frame(IgAIndex=targeted_index[,4], Taxa=targeted_taxa$Simple[4], Phylum=targeted_taxa$Phylum[4])
pdata5 <- data.frame(IgAIndex=targeted_index[,4], Taxa=targeted_taxa$Simple[4], Phylum=targeted_taxa$Phylum[5])


pdata <- Reduce(full_join, list(pdata1, pdata2, pdata3, pdata4,pdata5))
```

```
## Joining, by = c("IgAIndex", "Taxa", "Phylum")
## Joining, by = c("IgAIndex", "Taxa", "Phylum")
## Joining, by = c("IgAIndex", "Taxa", "Phylum")
## Joining, by = c("IgAIndex", "Taxa", "Phylum")
```

```
#plot - most targeted post-transplant
p <- ggplot(pdata) + geom_jitter(aes(x=Taxa, y=IgAIndex, colour=Phylum),
                                  width=0.2, height=0, shape=1, size=3)
p <- p + geom_boxplot(aes(x=Taxa, y=IgAIndex), fill=NA, outlier.shape=NA)
p <- p + ggtitle("Post")
p <- p + theme_bw(base_size=16) 
p <- p + theme(axis.text.x = element_text(angle=45, hjust=1))
p <- p + ylim(c(-1, 1))
p <- p + scale_color_manual(values=c("deepskyblue3", "indianred3"))
p
```

```
#Selecting the least targeted taxa; FDR<0.05, median index >0
least_targeted_post <- dplyr::filter(wilcox.data2.sub, wilcox.data2.sub<0.05&median<0)
least_targeted_post$Taxa
```

```
##  [1] "|Bacteria|Bacteroidetes|Bacteroidia|Bacteroidales|Bacteroidaceae|Bacteroides|NA|668fdb718997fc1589c7817655d4bb5f"                 
##  [2] "|Bacteria|Firmicutes|Clostridia|Clostridiales|Lachnospiraceae|NA|NA|78314aca268ae4422bc651192fbc986d"                             
##  [3] "|Bacteria|Firmicutes|Clostridia|Clostridiales|Lachnospiraceae|[Ruminococcus]|gnavus|90a05d597112b554e4480a8eaae4e0aa"             
##  [4] "|Bacteria|Firmicutes|Clostridia|Clostridiales|Ruminococcaceae|Ruminococcus|NA|98ff1f2b1008b5e0971997cd5070fc03"                   
##  [5] "|Bacteria|Firmicutes|Clostridia|Clostridiales|Lachnospiraceae|Blautia|NA|9f8668eb1c5f9d9a992dd49245db090e"                        
##  [6] "|Bacteria|Firmicutes|Clostridia|Clostridiales|Ruminococcaceae|Faecalibacterium|prausnitzii|aed3f59201e3b9d21858f36557f42a80"      
##  [7] "|Bacteria|Firmicutes|Clostridia|Clostridiales|Lachnospiraceae|NA|NA|d5c7d97e6f4f5789d574d321dcca0992"                             
##  [8] "|Bacteria|Firmicutes|Clostridia|Clostridiales|Ruminococcaceae|Faecalibacterium|prausnitzii|e553b9a0bb32467c71c89a4e97e55792"      
##  [9] "|Bacteria|Proteobacteria|Deltaproteobacteria|Desulfovibrionales|Desulfovibrionaceae|Bilophila|NA|ece50a62168f85fc61385d8adb4c6494"
## [10] "|Bacteria|Firmicutes|Clostridia|Clostridiales|Lachnospiraceae|Blautia|obeum|ee293984c0110b2eeceb8427fdf448fb"
```

```
#plot least targeted
targeted_index <- IgAPost_trim75_t[,which(names(IgAPost_trim75_t) %in% least_targeted_post$Taxa)]
targeted_taxa <- IgTAX75[which(IgTAX75$combined %in% least_targeted_post$Taxa),]
targeted_taxa$Simple <- paste(targeted_taxa$Genus, substr(targeted_taxa$ASV, start=0, stop=3))
targeted_taxa$Simple <- ifelse(is.na(targeted_taxa$Genus), yes= paste(targeted_taxa$Family, substr(targeted_taxa$ASV, start=0, stop=3)), no=targeted_taxa$Simple)

pdata1 <- data.frame(IgAIndex=targeted_index[,1], Taxa=targeted_taxa$Simple[1], Phylum=targeted_taxa$Phylum[1])
pdata2 <- data.frame(IgAIndex=targeted_index[,2], Taxa=targeted_taxa$Simple[2], Phylum=targeted_taxa$Phylum[2])
pdata3 <- data.frame(IgAIndex=targeted_index[,3], Taxa=targeted_taxa$Simple[3], Phylum=targeted_taxa$Phylum[3])
pdata4 <- data.frame(IgAIndex=targeted_index[,4], Taxa=targeted_taxa$Simple[4], Phylum=targeted_taxa$Phylum[4])
pdata5 <- data.frame(IgAIndex=targeted_index[,5], Taxa=targeted_taxa$Simple[5], Phylum=targeted_taxa$Phylum[5])
pdata6 <- data.frame(IgAIndex=targeted_index[,6], Taxa=targeted_taxa$Simple[6], Phylum=targeted_taxa$Phylum[6])
pdata7 <- data.frame(IgAIndex=targeted_index[,7], Taxa=targeted_taxa$Simple[7], Phylum=targeted_taxa$Phylum[7])
pdata8 <- data.frame(IgAIndex=targeted_index[,8], Taxa=targeted_taxa$Simple[8], Phylum=targeted_taxa$Phylum[8])
pdata9 <- data.frame(IgAIndex=targeted_index[,9], Taxa=targeted_taxa$Simple[9], Phylum=targeted_taxa$Phylum[9])
pdata10 <- data.frame(IgAIndex=targeted_index[,10], Taxa=targeted_taxa$Simple[10], Phylum=targeted_taxa$Phylum[10])

pdata <- Reduce(full_join, list(pdata1, pdata2, pdata3, pdata4, pdata5,
                                pdata6, pdata7, pdata8, pdata9, pdata10))
```

```
## Joining, by = c("IgAIndex", "Taxa", "Phylum")
## Joining, by = c("IgAIndex", "Taxa", "Phylum")
## Joining, by = c("IgAIndex", "Taxa", "Phylum")
## Joining, by = c("IgAIndex", "Taxa", "Phylum")
## Joining, by = c("IgAIndex", "Taxa", "Phylum")
## Joining, by = c("IgAIndex", "Taxa", "Phylum")
## Joining, by = c("IgAIndex", "Taxa", "Phylum")
## Joining, by = c("IgAIndex", "Taxa", "Phylum")
## Joining, by = c("IgAIndex", "Taxa", "Phylum")
```

```
#plot - least targeted post-transplant
p <- ggplot(pdata) + geom_jitter(aes(x=Taxa, y=IgAIndex, colour=Phylum),
                                 width=0.2, height=0, shape=1, size=3)
p <- p + geom_boxplot(aes(x=Taxa, y=IgAIndex), fill=NA, outlier.shape=NA)
p <- p + ggtitle("IgA- (Post)")
p <- p + theme_bw(base_size=16) 
p <- p + theme(axis.text.x = element_text(angle=45, hjust=1, size=11))
p <- p + ylim(c(-1, 1))
p <- p + scale_color_manual(values=c("forestgreen", "deepskyblue3", "indianred3"))
p
```

```
#also calculate averages for relative abundance
wilcox_postb <- Abund_settrim75_t[which(row.names(Abund_settrim75_t)%in%gsub("-", ".", metaIgAPost$SampleID, fixed=TRUE)),]

wilcox.data2b <- sapply(1:ncol(wilcox_postb), function(i){wilcox.test(wilcox_postb[,i], mu=0)$p.value})
wilcox.data2b <- as.data.frame(wilcox.data2b)
rownames(wilcox.data2b) <- colnames(wilcox_postb)
#Perform multiple comparison correction using a given method of choice
wilcox.data2b$rel.fdr <- p.adjust(wilcox.data2b$wilcox.data2b, method="fdr")
wilcox.data2b$bonferroni <- p.adjust(wilcox.data2b$wilcox.data2b, method="bonferroni")
#incorporate other metrics of data1 centre and dispersion
wilcox.data2b$mean <- t(summarise_each(wilcox_postb,funs(mean)))
wilcox.data2b$median <- t(summarise_each(wilcox_postb,funs(median)))
wilcox.data2b$variance <- t(summarise_each(wilcox_postb,funs(var))) 
wilcox.data2b$IQR <- t(summarise_each(wilcox_postb,funs(IQR)))
wilcox.data2b$Taxa <- row.names(wilcox.data2b)
wilcox.data2b$Subset <- c("Post")
```

Most targeted in donors (ASV)

```
#Most targeted taxa in the donors 
IgA_simplewilcox <- IgA_settrim75_t
row.names(IgA_simplewilcox) == gsub("-", ".", metaIgA$SampleID, fixed=TRUE)
```

```
##  [1] TRUE TRUE TRUE TRUE TRUE TRUE TRUE TRUE TRUE TRUE TRUE TRUE TRUE TRUE TRUE
## [16] TRUE TRUE TRUE TRUE TRUE TRUE TRUE TRUE TRUE TRUE TRUE TRUE TRUE TRUE TRUE
## [31] TRUE TRUE TRUE TRUE TRUE TRUE TRUE TRUE TRUE TRUE TRUE TRUE TRUE TRUE TRUE
## [46] TRUE TRUE TRUE TRUE TRUE TRUE TRUE TRUE TRUE TRUE TRUE TRUE TRUE TRUE TRUE
## [61] TRUE TRUE TRUE TRUE TRUE TRUE TRUE TRUE TRUE TRUE TRUE TRUE TRUE TRUE TRUE
## [76] TRUE TRUE
```

```
row.names(IgA_simplewilcox) <- metaIgA$SampleID

meta_wilcox_filt = filter(metaIgA, SampleType=="Donor")
samples_kept <- meta_wilcox_filt$SampleID
IgA_simplewilcox_filt <- filter(IgA_simplewilcox, rownames(IgA_simplewilcox) %in% samples_kept)
row.names(IgA_simplewilcox_filt) <- samples_kept

#apply wilcox test to test if Index is sig. diff. from 0
wilcox.data3 <- sapply(1:ncol(IgA_simplewilcox_filt), function(i){wilcox.test(IgA_simplewilcox_filt[,i], mu=0)$p.value})
wilcox.data3 <- as.data.frame(wilcox.data3)
rownames(wilcox.data3) <- colnames(IgA_simplewilcox_filt)
#Perform multiple comparison correction using a given method of choice
wilcox.data3$rel.fdr <- p.adjust(wilcox.data3$wilcox.data3, method="fdr")
wilcox.data3$bonferroni <- p.adjust(wilcox.data3$wilcox.data3, method="bonferroni")
#incorporate other metrics of data3 centre and dispersion
wilcox.data3$mean <- t(summarise_each(IgA_simplewilcox_filt,funs(mean)))
wilcox.data3$median <- t(summarise_each(IgA_simplewilcox_filt,funs(median)))
wilcox.data3$variance <- t(summarise_each(IgA_simplewilcox_filt,funs(var))) 
wilcox.data3$IQR <- t(summarise_each(IgA_simplewilcox_filt,funs(IQR)))
wilcox.data3$Taxa <- row.names(wilcox.data3)
wilcox.data3$Subset <- c("Donor")

#Selecting the most targeted taxa; FDR<0.05, median index >0
most_targeted_donor <- dplyr::filter(wilcox.data3, rel.fdr<0.05&median>0)
most_targeted_donor$Taxa
```

```
## character(0)
```

```
most_targeted_donor_mean <- dplyr::filter(wilcox.data3, rel.fdr<0.05&mean>0)
most_targeted_donor_mean$Taxa
```

```
## character(0)
```

```
targeted_donor_median <- dplyr::filter(wilcox.data3, median>0) #bit unfair to apply FDR on so few samples
targeted_donor_median$Taxa
```

```
##  [1] "|Bacteria|Bacteroidetes|Bacteroidia|Bacteroidales|Bacteroidaceae|Bacteroides|NA|51e441cbdcc80da0656e82293ae160b5"                  
##  [2] "|Bacteria|Bacteroidetes|Bacteroidia|Bacteroidales|Bacteroidaceae|Bacteroides|NA|25d727166a36df8d2f6a915a945bf5ac"                  
##  [3] "|Bacteria|Bacteroidetes|Sphingobacteriia|Sphingobacteriales|Sphingobacteriaceae|Pedobacter|NA|e5e45fe5960b838c45765068621efcd5"    
##  [4] "|Bacteria|Bacteroidetes|Bacteroidia|Bacteroidales|Rikenellaceae|NA|NA|e47a63686b619f67783f9b9aa52b86bf"                            
##  [5] "|Bacteria|Bacteroidetes|[Saprospirae]|[Saprospirales]|Chitinophagaceae|Sediminibacterium|NA|a47ed77e3a3ab04be7139730ee5e5a98"      
##  [6] "|Bacteria|Chlamydiae|Chlamydiia|Chlamydiales|Parachlamydiaceae|Parachlamydia|NA|8a592a80ca5be07c6b8d28ae3f835dcd"                  
##  [7] "|Bacteria|Proteobacteria|Gammaproteobacteria|Enterobacteriales|Enterobacteriaceae|NA|NA|bfe54af4c9180d37a0d76f6dafe79a5a"          
##  [8] "|Bacteria|Proteobacteria|Gammaproteobacteria|Pseudomonadales|Pseudomonadaceae|NA|NA|1f878f615fcfc8d7bd381a7841ac1e41"              
##  [9] "|Bacteria|Proteobacteria|Gammaproteobacteria|Pseudomonadales|Pseudomonadaceae|Pseudomonas|veronii|402e5913597695a16d7cad415ffff02f"
## [10] "|Bacteria|Proteobacteria|Deltaproteobacteria|Desulfovibrionales|Desulfovibrionaceae|Bilophila|NA|ece50a62168f85fc61385d8adb4c6494" 
## [11] "|Bacteria|Actinobacteria|Actinobacteria|Actinomycetales|NA|NA|NA|bb6c24bb90dd84f577f7488df6312451"                                 
## [12] "|Bacteria|Actinobacteria|Actinobacteria|Actinomycetales|Actinomycetaceae|Actinomyces|NA|608e6548b1b4cbb6176c8fce090991a5"          
## [13] "|Bacteria|Firmicutes|Clostridia|Clostridiales|Veillonellaceae|Dialister|NA|520c77820886daeb8cf0d6497cfb1344"                       
## [14] "|Bacteria|Firmicutes|Erysipelotrichi|Erysipelotrichales|Erysipelotrichaceae|NA|NA|f95cab37fba4160de15015f4d520839f"                
## [15] "|Bacteria|Firmicutes|Erysipelotrichi|Erysipelotrichales|Erysipelotrichaceae|Holdemania|NA|bc53814d35b2f862562a1e865e923b33"        
## [16] "|Bacteria|Firmicutes|Clostridia|Clostridiales|Clostridiaceae|Clostridium|NA|5c82dc47435864e490625ae01151570e"                      
## [17] "|Bacteria|Firmicutes|Clostridia|Clostridiales|Ruminococcaceae|NA|NA|26f6853f46b06854fe5418317a261fb8"                              
## [18] "|Bacteria|Firmicutes|Clostridia|Clostridiales|Ruminococcaceae|NA|NA|c4995645e0c1545b0e1620144d03772b"                              
## [19] "|Bacteria|Firmicutes|Clostridia|Clostridiales|Ruminococcaceae|Faecalibacterium|prausnitzii|c3bdda568b2c1580d5cce7407ef43909"       
## [20] "|Bacteria|Firmicutes|Clostridia|Clostridiales|Ruminococcaceae|Faecalibacterium|prausnitzii|a3f36ef32153f2fc2aaeac2feb23777f"       
## [21] "|Bacteria|Firmicutes|Clostridia|Clostridiales|Lachnospiraceae|NA|NA|8b8b93b27e9c6cc58aacb0bf38248700"                              
## [22] "|Bacteria|Firmicutes|Clostridia|Clostridiales|Lachnospiraceae|Roseburia|faecis|2d34c22edce4b1f2d8a5228ad78f8ea8"                   
## [23] "|Bacteria|Firmicutes|Clostridia|Clostridiales|Lachnospiraceae|[Ruminococcus]|torques|e59405a47acbc248ce61395366159d8d"             
## [24] "|Bacteria|Firmicutes|Clostridia|Clostridiales|Lachnospiraceae|NA|NA|694d61b8ec78349749c8b6ea59938e0b"                              
## [25] "|Bacteria|Firmicutes|Clostridia|Clostridiales|Lachnospiraceae|Blautia|NA|c1dc9ad5116d96b8ed863458fc0d0aec"                         
## [26] "|Bacteria|Firmicutes|Clostridia|Clostridiales|Lachnospiraceae|NA|NA|eb61cae65bc6cdd2440323bbf603ba5c"                              
## [27] "|Bacteria|Firmicutes|Clostridia|Clostridiales|Lachnospiraceae|Dorea|formicigenerans|afd87e82de329a1ed75b98b5b606843c"
```

```
#also calculate averages for relative abundance
Abund_simplewilcox <- Abund_settrim75_t
row.names(Abund_simplewilcox) == gsub("-", ".", metaIgA$SampleID, fixed=TRUE)
```

```
##  [1] TRUE TRUE TRUE TRUE TRUE TRUE TRUE TRUE TRUE TRUE TRUE TRUE TRUE TRUE TRUE
## [16] TRUE TRUE TRUE TRUE TRUE TRUE TRUE TRUE TRUE TRUE TRUE TRUE TRUE TRUE TRUE
## [31] TRUE TRUE TRUE TRUE TRUE TRUE TRUE TRUE TRUE TRUE TRUE TRUE TRUE TRUE TRUE
## [46] TRUE TRUE TRUE TRUE TRUE TRUE TRUE TRUE TRUE TRUE TRUE TRUE TRUE TRUE TRUE
## [61] TRUE TRUE TRUE TRUE TRUE TRUE TRUE TRUE TRUE TRUE TRUE TRUE TRUE TRUE TRUE
## [76] TRUE TRUE
```

```
row.names(Abund_simplewilcox) <- metaIgA$SampleID
Abund_simplewilcox_filt <- filter(Abund_simplewilcox, rownames(Abund_simplewilcox) %in% samples_kept)
row.names(Abund_simplewilcox_filt) <- samples_kept

wilcox.data3b <- sapply(1:ncol(Abund_simplewilcox_filt), function(i){wilcox.test(Abund_simplewilcox_filt[,i], mu=0)$p.value})
wilcox.data3b <- as.data.frame(wilcox.data3b)
rownames(wilcox.data3b) <- colnames(Abund_simplewilcox_filt)
#Perform multiple comparison correction using a given method of choice
wilcox.data3b$rel.fdr <- p.adjust(wilcox.data3b$wilcox.data3b, method="fdr")
wilcox.data3b$bonferroni <- p.adjust(wilcox.data3b$wilcox.data3b, method="bonferroni")
#incorporate other metrics of data1 centre and dispersion
wilcox.data3b$mean <- t(summarise_each(Abund_simplewilcox_filt,funs(mean)))
wilcox.data3b$median <- t(summarise_each(Abund_simplewilcox_filt,funs(median)))
wilcox.data3b$variance <- t(summarise_each(Abund_simplewilcox_filt,funs(var))) 
wilcox.data3b$IQR <- t(summarise_each(Abund_simplewilcox_filt,funs(IQR)))
wilcox.data3b$Taxa <- row.names(wilcox.data3b)
wilcox.data3b$Subset <- c("Donor")
```

Tile plot of donor, pre and post (ASV) by mean

```
###make a bubble plot of all targeted taxa 
names(wilcox.data1)[names(wilcox.data1) == 'wilcox.data1'] <- 'p.val'
names(wilcox.data2)[names(wilcox.data2) == 'wilcox.data2'] <- 'p.val'
names(wilcox.data3)[names(wilcox.data3) == 'wilcox.data3'] <- 'p.val'
bubble.all <- Reduce(full_join, list(wilcox.data1,wilcox.data2, wilcox.data3))
```

```
## Joining, by = c("p.val", "rel.fdr", "bonferroni", "mean", "median", "variance", "IQR", "Taxa", "Subset")
## Joining, by = c("p.val", "rel.fdr", "bonferroni", "mean", "median", "variance", "IQR", "Taxa", "Subset")
```

```
#simplify by keeping only Taxa with at least one significant value
bubble.simple <- filter(bubble.all, rel.fdr<0.1)
TaxaToKeep_main <- bubble.simple$Taxa
bubble.filter <- filter(bubble.all, Taxa %in% TaxaToKeep_main)
TaxaNames_main <- filter(IgTAX, IgTAX$combined %in% bubble.filter$Taxa)
TaxaNames_main$Simple <- ifelse(is.na(TaxaNames_main$Genus), 
                           yes = as.character(TaxaNames_main$Family), no=TaxaNames_main$Genus)
TaxaNames_main$SimpleASV <- paste(TaxaNames_main$Simple, substr(TaxaNames_main$ASV, start=0, stop=3), sep=" ")
TaxaNames_main$Taxa <- TaxaNames_main$combined
bubble.filter <- full_join(bubble.filter, TaxaNames_main, by="Taxa")

###bubble plot
bubble.filter$Subset <- factor(bubble.filter$Subset, levels = c("Donor", 
                                                                "Pre", 
                                                                "Post"))

#pdf("/Users/kelseyhuus/Dropbox/PhD/Manuscripts/Cdiff_IgA_paper/CdiffFinalAnalysis/Graphs2/TilePlot_SampleType_rarefied.pdf", 
#    width = 2.5, 
#    height = 6)
p <- ggplot(bubble.filter, aes(x=Subset, y=SimpleASV)) + geom_tile(aes(fill=mean))
p <- p + scale_fill_gradient2(low="deepskyblue4",
                              mid="white",
                              high="indianred4",
                              midpoint=0)
p <- p + theme(axis.text.x  = element_text(angle=90, vjust=0.5, size=12), legend.key.size=unit(0.75, "cm"))
p <- p + scale_y_discrete(limits = rev(levels(bubble.filter$Taxa)))
p <- p + theme(strip.background = element_rect(fill="gray85"),
               panel.background = element_rect(fill="white"),
               panel.border = element_rect(colour="black", linetype="solid", fill="transparent") 
)
#p <- p + facet_grid(.~Subset, scales = "free", space = "free",
#                    labeller=label_wrap_gen(width=2))
p <- p + guides(colour=FALSE, size=FALSE, shape=FALSE, fill=FALSE)
p
```

```
#dev.off()

#for legend only
#p <- ggplot(bubble.filter, aes(x=Subset, y=SimpleASV)) + geom_tile(aes(fill=mean))
#p <- p + scale_fill_gradient2(low="deepskyblue4",
 #                             mid="white",
 #                             high="indianred4",
 #                             midpoint=0)
#p <- p + theme(axis.text.x  = element_text(angle=90, vjust=0.5, size=12), legend.key.size=unit(0.75, "cm"))
#p <- p + labs(fill="IgA Index")
#p

#also make a plot for relative abundance for the same taxa
###make a bubble plot of all targeted taxa with FDR<0.05
bubble.all2 <- Reduce(full_join, list(wilcox.data1b,wilcox.data2b, wilcox.data3b))
```

```
## Joining, by = c("rel.fdr", "bonferroni", "mean", "median", "variance", "IQR", "Taxa", "Subset")
```

```
## Joining, by = c("rel.fdr", "bonferroni", "mean", "median", "variance", "IQR", "Taxa", "Subset")
```

```
#simplify by keeping only Taxa with at least one non-zero value 
bubble.filter2 <- filter(bubble.all2, Taxa %in% TaxaToKeep_main)
bubble.filter2 <- full_join(bubble.filter2, TaxaNames_main, by="Taxa")

###bubble plot abundance
bubble.filter2$Subset <- factor(bubble.filter2$Subset, levels = c("Donor", 
                                                                "Pre", 
                                                                "Post"))

#pdf("/Users/kelseyhuus/Dropbox/PhD/Manuscripts/Cdiff_IgA_paper/CdiffFinalAnalysis/Graphs2/TilePlot_SampleType_rarefied_Abund.pdf", 
#    width = 2.5, 
#    height = 6)
p <- ggplot(bubble.filter2, aes(x=Subset, y=SimpleASV)) + geom_tile(aes(fill=mean, line="black"))
p <- p + scale_fill_gradientn(colours=c("white","indianred4"),
                             values=c(0,0.1,0.9,1))
p <- p + scale_y_discrete(limits = rev(levels(bubble.filter$Taxa)))
p <- p + theme(axis.text.x  = element_text(angle=90, vjust=0.5, size=12), legend.key.size=unit(0.75, "cm"))
p <- p + theme(strip.background = element_rect(fill="gray85"),
               panel.background = element_rect(fill="white"),
               panel.border = element_rect(colour="black", linetype="solid", fill="transparent") 
)
#p <- p + facet_grid(.~Subset, scales = "free", space = "free",
#                    labeller=label_wrap_gen(width=2))
p <- p + guides(colour=FALSE, size=FALSE, shape=FALSE, fill=FALSE)
p
```

```
#dev.off()

#with legend
#p <- ggplot(bubble.filter2, aes(x=Subset, y=SimpleASV)) + geom_tile(aes(fill=mean))
#p <- p + scale_fill_gradientn(colours=c("white","indianred4"),
#                            values=c(0,0.1,0.9,1))
#p <- p + theme(axis.text.x  = element_text(angle=90, vjust=0.5, size=12), legend.key.size=unit(0.75, "cm"))
#p <- p + labs(fill="Abundance")
#p
```

Plot specific taxa IgA Index by donor, pre and post

```
metaIgA$SampleType2 <- metaIgA$SampleType
metaIgA$SampleType2 <- gsub("PreTransplant", "Pre", metaIgA$SampleType2)
metaIgA$SampleType2 <- gsub("PostTransplant", "Post", metaIgA$SampleType2)
metaIgA$SampleType2 <- factor(metaIgA$SampleType2, 
                              levels=c("Donor", "Pre", "Post"))


#Escherichia coli IgA
pdata <- data.frame(IgAIndex=IgA_settrim75_t$`|Bacteria|Proteobacteria|Gammaproteobacteria|Enterobacteriales|Enterobacteriaceae|Escherichia|coli|1b158b8b2922d4fcad5d9cea607cbb7d`,
                    SampleType=metaIgA$SampleType2,
                    Patient=metaIgA$Patient)
kruskal.test(pdata$IgAIndex~pdata$SampleType)
```

```
## 
##  Kruskal-Wallis rank sum test
## 
## data:  pdata$IgAIndex by pdata$SampleType
## Kruskal-Wallis chi-squared = 7.9899, df = 2, p-value = 0.01841
```

```
posthoc <- dunnTest(pdata$IgAIndex~pdata$SampleType, data=pdata, method='bh')
posthoc
```

```
## Dunn (1964) Kruskal-Wallis multiple comparison
```

```
##   p-values adjusted with the Benjamini-Hochberg method.
```

```
##     Comparison         Z     P.unadj      P.adj
## 1 Donor - Post -2.225551 0.026044244 0.03906637
## 2  Donor - Pre -2.788125 0.005301409 0.01590423
## 3   Post - Pre -1.139125 0.254651174 0.25465117
```

```
p <- ggplot(pdata) + geom_boxplot(aes(x=SampleType, y=IgAIndex, fill=SampleType), 
                                  outlier.shape=NA, outlier.size=NA)
p <- p + geom_jitter(aes(x=SampleType, y=IgAIndex),
                     width=0.2, height=0, shape=1, size=3)
p <- p + ggtitle("E. coli 1b1")
p <- p + theme(axis.text.x  = element_text(angle=90, vjust=0.5, size=6), plot.title = element_text(size = 9))
p <- p + theme_bw(base_size=15)
p <- p + scale_fill_manual(values=c("seagreen3", "indianred3", "deepskyblue3"))
p <- p +ylim(c(-1, 1))
p <- p + annotate("text", x = 1.5, y=1, label = "*", size=4)
p <- p + annotate("segment", x=1.1, xend=1.9, y=0.9, yend=0.9)
p <- p + annotate("text", x = 2.5, y=1, label = "ns", size=4)
p <- p + annotate("segment", x=2.1, xend=2.9, y=0.9, yend=0.9)
p <- p + guides(colour=FALSE, size=FALSE, shape=FALSE, fill=FALSE)
p
```

```
#E. coli IgA - no donor, paired
pdata_filt <- pdata[which(pdata$SampleType%in%c("Pre", "Post")),]

p <- ggplot(pdata_filt) + geom_boxplot(aes(x=SampleType, y=IgAIndex, fill=SampleType), 
                                  outlier.shape=NA, outlier.size=NA)
p <- p + geom_point(aes(x=SampleType, y=IgAIndex),
                     shape=1, size=3)
p <- p + geom_line(aes(x=SampleType, y=IgAIndex, group=Patient))
p <- p + ggtitle("E. coli 1b1")
p <- p + theme(axis.text.x  = element_text(angle=90, vjust=0.5, size=6), plot.title = element_text(size = 9))
p <- p + theme_bw(base_size=15)
p <- p + scale_fill_manual(values=c("indianred3", "deepskyblue3"))
p <- p + ylim(c(-1, 1))
p <- p + annotate("text", x = 1.5, y=1, label = "ns", size=4)
p <- p + annotate("segment", x=1, xend=2, y=0.9, yend=0.9)
p <- p + guides(colour=FALSE, size=FALSE, shape=FALSE, fill=FALSE)
p
```

```
#Ruminococcus e59
pdata <- data.frame(IgAIndex=IgA_settrim75_t$`|Bacteria|Firmicutes|Clostridia|Clostridiales|Lachnospiraceae|[Ruminococcus]|torques|e59405a47acbc248ce61395366159d8d`,
                    SampleType=metaIgA$SampleType2)
kruskal.test(pdata$IgAIndex~pdata$SampleType)
```

```
## 
##  Kruskal-Wallis rank sum test
## 
## data:  pdata$IgAIndex by pdata$SampleType
## Kruskal-Wallis chi-squared = 39.88, df = 2, p-value = 2.188e-09
```

```
posthoc <- dunnTest(pdata$IgAIndex~pdata$SampleType, data=pdata, method='bh')
posthoc
```

```
## Dunn (1964) Kruskal-Wallis multiple comparison
##   p-values adjusted with the Benjamini-Hochberg method.
```

```
##     Comparison         Z      P.unadj        P.adj
## 1 Donor - Post 0.1049292 9.164320e-01 9.164320e-01
## 2  Donor - Pre 3.1139296 1.846135e-03 2.769203e-03
## 3   Post - Pre 6.0927614 1.109794e-09 3.329382e-09
```

```
p <- ggplot(pdata) + geom_boxplot(aes(x=SampleType, y=IgAIndex, fill=SampleType), 
                                  outlier.shape=NA, outlier.size=NA)
p <- p + geom_jitter(aes(x=SampleType, y=IgAIndex),
                     width=0.2, height=0, shape=1, size=3)
p <- p + ggtitle("R. torques e59")
p <- p + theme(axis.text.x  = element_text(angle=90, vjust=0.5, size=6), plot.title = element_text(size = 9))
p <- p + theme_bw(base_size=15)
p <- p + scale_fill_manual(values=c("seagreen3", "indianred3", "deepskyblue3"))
p <- p + ylim(c(-1, 1))
p <- p + annotate("text", x = 1.5, y=1, label = "**", size=4)
p <- p + annotate("segment", x=1.1, xend=1.9, y=0.9, yend=0.9)
p <- p + annotate("text", x = 2.5, y=1, label = "****", size=4)
p <- p + annotate("segment", x=2.1, xend=2.9, y=0.9, yend=0.9)
p <- p + guides(colour=FALSE, size=FALSE, shape=FALSE, fill=FALSE)
p
```

```
#Ruminococcus 98f
pdata <- data.frame(IgAIndex=IgA_settrim75_t$`|Bacteria|Firmicutes|Clostridia|Clostridiales|Ruminococcaceae|Ruminococcus|NA|98ff1f2b1008b5e0971997cd5070fc03`,
                    SampleType=metaIgA$SampleType2)
kruskal.test(pdata$IgAIndex~pdata$SampleType)
```

```
## 
##  Kruskal-Wallis rank sum test
## 
## data:  pdata$IgAIndex by pdata$SampleType
## Kruskal-Wallis chi-squared = 23.53, df = 2, p-value = 7.774e-06
```

```
posthoc <- dunnTest(pdata$IgAIndex~pdata$SampleType, data=pdata, method='bh')
posthoc
```

```
## Dunn (1964) Kruskal-Wallis multiple comparison
##   p-values adjusted with the Benjamini-Hochberg method.
```

```
##     Comparison         Z      P.unadj        P.adj
## 1 Donor - Post -1.921050 0.0547254669 0.0547254669
## 2  Donor - Pre -3.818382 0.0001343296 0.0002014944
## 3   Post - Pre -3.841806 0.0001221323 0.0003663968
```

```
p <- ggplot(pdata) + geom_boxplot(aes(x=SampleType, y=IgAIndex, fill=SampleType), 
                                  outlier.shape=NA, outlier.size=NA)
p <- p + geom_jitter(aes(x=SampleType, y=IgAIndex),
                     width=0.2, height=0, shape=1, size=3)
p <- p + ggtitle("Ruminococcus 98f")
p <- p + theme(axis.text.x  = element_text(angle=90, vjust=0.5, size=6), plot.title = element_text(size = 9))
p <- p + theme_bw(base_size=15)
p <- p + scale_fill_manual(values=c("seagreen3", "indianred3", "deepskyblue3"))
p <- p + ylim(c(-1, 1))
p <- p + annotate("text", x = 1.5, y=1, label = "***", size=4)
p <- p + annotate("segment", x=1.1, xend=1.9, y=0.9, yend=0.9)
p <- p + annotate("text", x = 2.5, y=1, label = "***", size=4)
p <- p + annotate("segment", x=2.1, xend=2.9, y=0.9, yend=0.9)
p <- p + guides(colour=FALSE, size=FALSE, shape=FALSE, fill=FALSE)
p
```

```
#Escherichia coli Abund
pdata <- data.frame(Abund=Abund_settrim75_t$`|Bacteria|Proteobacteria|Gammaproteobacteria|Enterobacteriales|Enterobacteriaceae|Escherichia|coli|1b158b8b2922d4fcad5d9cea607cbb7d`,
                    SampleType=metaIgA$SampleType2)

kruskal.test(pdata$Abund~pdata$SampleType)
```

```
## 
##  Kruskal-Wallis rank sum test
## 
## data:  pdata$Abund by pdata$SampleType
## Kruskal-Wallis chi-squared = 28.217, df = 2, p-value = 7.461e-07
```

```
posthoc <- dunnTest(pdata$Abund~pdata$SampleType, data=pdata, method='bh')
posthoc
```

```
## Dunn (1964) Kruskal-Wallis multiple comparison
##   p-values adjusted with the Benjamini-Hochberg method.
```

```
##     Comparison         Z      P.unadj        P.adj
## 1 Donor - Post -1.727980 8.399185e-02 8.399185e-02
## 2  Donor - Pre -3.921125 8.813659e-05 1.322049e-04
## 3   Post - Pre -4.440780 8.963328e-06 2.688998e-05
```

```
p <- ggplot(pdata) + geom_boxplot(aes(x=SampleType, y=Abund, fill=SampleType), 
                                  outlier.shape=NA, outlier.size=NA)
p <- p + geom_jitter(aes(x=SampleType, y=Abund),
                     width=0.2, height=0, shape=1, size=3)
p <- p + ggtitle("E. coli 1b1")
p <- p + theme(axis.text.x  = element_text(angle=90, vjust=0.5, size=6), plot.title = element_text(size = 9))
p <- p + theme_bw(base_size=15)
p <- p + scale_fill_manual(values=c("seagreen3", "indianred3", "deepskyblue3"))
p <- p + ylab("Relative Abundance")
p <- p + annotate("text", x = 1.5, y=0.95, label = "****", size=4)
p <- p + annotate("text", x = 2.5, y=0.95, label = "****", size=4)
p <- p + annotate("segment", x=1.1, xend= 1.9, y=0.9, yend=0.9)
p <- p + annotate("segment", x=2.1, xend= 2.9, y=0.9, yend=0.9)
p <- p + guides(colour=FALSE, size=FALSE, shape=FALSE, fill=FALSE)
p
```

```
#Ruminococcus e59 abundance
pdata <- data.frame(Abund=Abund_settrim75_t$`|Bacteria|Firmicutes|Clostridia|Clostridiales|Lachnospiraceae|[Ruminococcus]|torques|e59405a47acbc248ce61395366159d8d`,
                    SampleType=metaIgA$SampleType2)
kruskal.test(pdata$Abund~pdata$SampleType)
```

```
## 
##  Kruskal-Wallis rank sum test
## 
## data:  pdata$Abund by pdata$SampleType
## Kruskal-Wallis chi-squared = 34.474, df = 2, p-value = 3.267e-08
```

```
posthoc <- dunnTest(pdata$Abund~pdata$SampleType, data=pdata, method='bh')
posthoc
```

```
## Dunn (1964) Kruskal-Wallis multiple comparison
##   p-values adjusted with the Benjamini-Hochberg method.
```

```
##     Comparison          Z      P.unadj        P.adj
## 1 Donor - Post -0.3626648 7.168553e-01 7.168553e-01
## 2  Donor - Pre  2.4861011 1.291513e-02 1.937269e-02
## 3   Post - Pre  5.7683112 8.006984e-09 2.402095e-08
```

```
p <- ggplot(pdata) + geom_boxplot(aes(x=SampleType, y=Abund, fill=SampleType), 
                                  outlier.shape=NA, outlier.size=NA)
p <- p + geom_jitter(aes(x=SampleType, y=Abund),
                     width=0.2, height=0, shape=1, size=3)
p <- p + ggtitle("R. torques e59")
p <- p + theme(axis.text.x  = element_text(angle=90, vjust=0.5, size=6), plot.title = element_text(size = 9))
p <- p + theme_bw(base_size=15)
p <- p + scale_fill_manual(values=c("seagreen3", "indianred3", "deepskyblue3"))
p <- p + annotate("text", x = 1.5, y=0.095, label = "*", size=4)
p <- p + annotate("text", x = 2.5, y=0.095, label = "****", size=4)
p <- p + annotate("segment", x=1.1, xend= 1.9, y=0.09, yend=0.09)
p <- p + annotate("segment", x=2.1, xend= 2.9, y=0.09, yend=0.09)
p <- p + ylab("Relative Abundance")
p <- p + guides(colour=FALSE, size=FALSE, shape=FALSE, fill=FALSE)
p
```

```
#Ruminococcus 98f abundance
pdata <- data.frame(Abund=Abund_settrim75_t$`|Bacteria|Firmicutes|Clostridia|Clostridiales|Ruminococcaceae|Ruminococcus|NA|98ff1f2b1008b5e0971997cd5070fc03`,
                    SampleType=metaIgA$SampleType2)
kruskal.test(pdata$Abund~pdata$SampleType)
```

```
## 
##  Kruskal-Wallis rank sum test
## 
## data:  pdata$Abund by pdata$SampleType
## Kruskal-Wallis chi-squared = 45.859, df = 2, p-value = 1.101e-10
```

```
posthoc <- dunnTest(pdata$Abund~pdata$SampleType, data=pdata, method='bh')
posthoc
```

```
## Dunn (1964) Kruskal-Wallis multiple comparison
##   p-values adjusted with the Benjamini-Hochberg method.
```

```
##     Comparison        Z      P.unadj        P.adj
## 1 Donor - Post 0.562989 5.734424e-01 5.734424e-01
## 2  Donor - Pre 3.723948 1.961311e-04 2.941966e-04
## 3   Post - Pre 6.400455 1.549147e-10 4.647441e-10
```

```
p <- ggplot(pdata) + geom_boxplot(aes(x=SampleType, y=Abund, fill=SampleType), 
                                  outlier.shape=NA, outlier.size=NA)
p <- p + geom_jitter(aes(x=SampleType, y=Abund),
                     width=0.2, height=0, shape=1, size=3)
p <- p + ggtitle("Ruminococcus 98f")
p <- p + theme(axis.text.x  = element_text(angle=90, vjust=0.5, size=6), plot.title = element_text(size = 9))
p <- p + theme_bw(base_size=15)
p <- p + scale_fill_manual(values=c("seagreen3", "indianred3", "deepskyblue3"))
p <- p + annotate("text", x = 1.5, y=0.095, label = "***", size=4)
p <- p + annotate("text", x = 2.5, y=0.095, label = "****", size=4)
p <- p + annotate("segment", x=1.1, xend= 1.9, y=0.09, yend=0.09)
p <- p + annotate("segment", x=2.1, xend= 2.9, y=0.09, yend=0.09)
p <- p + ylab("Relative Abundance")
p <- p + guides(colour=FALSE, size=FALSE, shape=FALSE, fill=FALSE)
p
```

Most targeted BY individual donor/donor recipient, post-transplant

```
#samplesizes
dplyr::count(metaIgAPost, Donor.ID2)
```

```
##   Donor.ID2  n
## 1    Donor2 14
## 2   Donor20  9
## 3   Donor21  1
## 4    Donor7 12
```

```
IgA_simplewilcox <- IgA_settrim75_t
row.names(IgA_simplewilcox) == gsub("-", ".", metaIgA$SampleID, fixed=TRUE)
```

```
##  [1] TRUE TRUE TRUE TRUE TRUE TRUE TRUE TRUE TRUE TRUE TRUE TRUE TRUE TRUE TRUE
## [16] TRUE TRUE TRUE TRUE TRUE TRUE TRUE TRUE TRUE TRUE TRUE TRUE TRUE TRUE TRUE
## [31] TRUE TRUE TRUE TRUE TRUE TRUE TRUE TRUE TRUE TRUE TRUE TRUE TRUE TRUE TRUE
## [46] TRUE TRUE TRUE TRUE TRUE TRUE TRUE TRUE TRUE TRUE TRUE TRUE TRUE TRUE TRUE
## [61] TRUE TRUE TRUE TRUE TRUE TRUE TRUE TRUE TRUE TRUE TRUE TRUE TRUE TRUE TRUE
## [76] TRUE TRUE
```

```
row.names(IgA_simplewilcox) <- metaIgA$SampleID

Abund_simplewilcox <- Abund_settrim75_t
row.names(Abund_simplewilcox) == gsub("-", ".", metaIgA$SampleID, fixed=TRUE)
```

```
##  [1] TRUE TRUE TRUE TRUE TRUE TRUE TRUE TRUE TRUE TRUE TRUE TRUE TRUE TRUE TRUE
## [16] TRUE TRUE TRUE TRUE TRUE TRUE TRUE TRUE TRUE TRUE TRUE TRUE TRUE TRUE TRUE
## [31] TRUE TRUE TRUE TRUE TRUE TRUE TRUE TRUE TRUE TRUE TRUE TRUE TRUE TRUE TRUE
## [46] TRUE TRUE TRUE TRUE TRUE TRUE TRUE TRUE TRUE TRUE TRUE TRUE TRUE TRUE TRUE
## [61] TRUE TRUE TRUE TRUE TRUE TRUE TRUE TRUE TRUE TRUE TRUE TRUE TRUE TRUE TRUE
## [76] TRUE TRUE
```

```
row.names(Abund_simplewilcox) <- metaIgA$SampleID

#Donor2 Recipients
meta_wilcox_filt = filter(metaIgA, SampleType=="PostTransplant"&Donor.ID2=="Donor 2")
samples_kept <- meta_wilcox_filt$SampleID
IgA_simplewilcox_filt <- filter(IgA_simplewilcox, rownames(IgA_simplewilcox) %in% samples_kept)

#apply wilcox test to test if Index is sig. diff. from 0
wilcox.data4 <- sapply(1:ncol(IgA_simplewilcox_filt), function(i){wilcox.test(IgA_simplewilcox_filt[,i], mu=0)$p.value})
wilcox.data4 <- as.data.frame(wilcox.data4)
rownames(wilcox.data4) <- colnames(IgA_simplewilcox_filt)
#Perform multiple comparison correction using a given method of choice
wilcox.data4$rel.fdr <- p.adjust(wilcox.data4$wilcox.data4, method="fdr")
#incorporate other metrics of data4 centre and dispersion
wilcox.data4$mean <- t(summarise_each(IgA_simplewilcox_filt,funs(mean)))
wilcox.data4$median <- t(summarise_each(IgA_simplewilcox_filt,funs(median)))
wilcox.data4$variance <- t(summarise_each(IgA_simplewilcox_filt,funs(var))) 
wilcox.data4$IQR <- t(summarise_each(IgA_simplewilcox_filt,funs(IQR)))
wilcox.data4$Taxa <- row.names(wilcox.data4)
wilcox.data4$Subset1 <- c("1") #Donor2
wilcox.data4$Subset2 <- c("R") #Recipient

#Selecting the most targeted taxa; FDR<0.05, median index >0
most_targeted_donor2 <- dplyr::filter(wilcox.data4, wilcox.data4<0.05&median>0)
most_targeted_donor2$Taxa
```

```
## [1] "|Bacteria|Firmicutes|Erysipelotrichi|Erysipelotrichales|Erysipelotrichaceae|NA|NA|216aca81784431f49e9567d23f7391e8"   
## [2] "|Bacteria|Firmicutes|Clostridia|Clostridiales|Lachnospiraceae|[Ruminococcus]|gnavus|cc2d96099f530b503371e5ddca8c0a58" 
## [3] "|Bacteria|Firmicutes|Clostridia|Clostridiales|Lachnospiraceae|NA|NA|ac04fef6eb13ddf89756a0af35b512fe"                 
## [4] "|Bacteria|Firmicutes|Clostridia|Clostridiales|Lachnospiraceae|NA|NA|b20c095fd654b84cebdbfe4faa0a1409"                 
## [5] "|Bacteria|Firmicutes|Clostridia|Clostridiales|Lachnospiraceae|[Ruminococcus]|torques|e59405a47acbc248ce61395366159d8d"
## [6] "|Bacteria|Firmicutes|Clostridia|Clostridiales|Lachnospiraceae|NA|NA|eb61cae65bc6cdd2440323bbf603ba5c"                 
## [7] "|Bacteria|Firmicutes|Clostridia|Clostridiales|Lachnospiraceae|Dorea|NA|a0b7d83fb64749f9a4b15b3728425b97"
```

```
#abundance
Abund_simplewilcox_filt <- filter(Abund_simplewilcox, rownames(Abund_simplewilcox) %in% samples_kept)

#apply wilcox test to test if Index is sig. diff. from 0
wilcox.data4b <- sapply(1:ncol(Abund_simplewilcox_filt), function(i){wilcox.test(Abund_simplewilcox_filt[,i], mu=0)$p.value})
wilcox.data4b <- as.data.frame(wilcox.data4b)
rownames(wilcox.data4b) <- colnames(Abund_simplewilcox_filt)
#Perform multiple comparison correction using a given method of choice
wilcox.data4b$rel.fdr <- p.adjust(wilcox.data4b$wilcox.data4b, method="fdr")
#incorporate other metrics of data4b centre and dispersion
wilcox.data4b$mean <- t(summarise_each(Abund_simplewilcox_filt,funs(mean)))
wilcox.data4b$median <- t(summarise_each(Abund_simplewilcox_filt,funs(median)))
wilcox.data4b$variance <- t(summarise_each(Abund_simplewilcox_filt,funs(var))) 
wilcox.data4b$IQR <- t(summarise_each(Abund_simplewilcox_filt,funs(IQR)))
wilcox.data4b$Taxa <- row.names(wilcox.data4b)
wilcox.data4b$Subset1 <- c("1") #Donor2
wilcox.data4b$Subset2 <- c("R") #Recipient

#Donor7 Recipients
meta_wilcox_filt = filter(metaIgA, SampleType=="PostTransplant"&Donor.ID2=="Donor 7")
samples_kept <- meta_wilcox_filt$SampleID
IgA_simplewilcox_filt <- filter(IgA_simplewilcox, rownames(IgA_simplewilcox) %in% samples_kept)

#apply wilcox test to test if Index is sig. diff. from 0
wilcox.data5 <- sapply(1:ncol(IgA_simplewilcox_filt), function(i){wilcox.test(IgA_simplewilcox_filt[,i], mu=0)$p.value})
wilcox.data5 <- as.data.frame(wilcox.data5)
rownames(wilcox.data5) <- colnames(IgA_simplewilcox_filt)
#Perform multiple comparison correction using a given method of choice
wilcox.data5$rel.fdr <- p.adjust(wilcox.data5$wilcox.data5, method="fdr")
#incorporate other metrics of data5 centre and dispersion
wilcox.data5$mean <- t(summarise_each(IgA_simplewilcox_filt,funs(mean)))
wilcox.data5$median <- t(summarise_each(IgA_simplewilcox_filt,funs(median)))
wilcox.data5$variance <- t(summarise_each(IgA_simplewilcox_filt,funs(var))) 
wilcox.data5$IQR <- t(summarise_each(IgA_simplewilcox_filt,funs(IQR)))
wilcox.data5$Taxa <- row.names(wilcox.data5)
wilcox.data5$Subset1 <- c("2") #Donor7
wilcox.data5$Subset2 <- c("R") #Recipient

#Selecting the most targeted taxa; FDR<0.05, median index >0
most_targeted_donor7 <- dplyr::filter(wilcox.data5, wilcox.data5<0.05&median>0)
most_targeted_donor7$Taxa
```

```
## [1] "|Bacteria|Firmicutes|Clostridia|Clostridiales|Ruminococcaceae|NA|NA|e803ff46adaa0fa149ef151b082378a0"                 
## [2] "|Bacteria|Firmicutes|Clostridia|Clostridiales|Ruminococcaceae|NA|NA|63027647b049a274c881df23a4611b35"                 
## [3] "|Bacteria|Firmicutes|Clostridia|Clostridiales|Ruminococcaceae|NA|NA|2673143ffb9d8ff3ad82fa40e325973c"                 
## [4] "|Bacteria|Firmicutes|Clostridia|Clostridiales|Lachnospiraceae|NA|NA|ac04fef6eb13ddf89756a0af35b512fe"                 
## [5] "|Bacteria|Firmicutes|Clostridia|Clostridiales|Lachnospiraceae|[Ruminococcus]|torques|e59405a47acbc248ce61395366159d8d"
```

```
#abundance
Abund_simplewilcox_filt <- filter(Abund_simplewilcox, rownames(Abund_simplewilcox) %in% samples_kept)

#apply wilcox test to test if Index is sig. diff. from 0
wilcox.data5b <- sapply(1:ncol(Abund_simplewilcox_filt), function(i){wilcox.test(Abund_simplewilcox_filt[,i], mu=0)$p.value})
wilcox.data5b <- as.data.frame(wilcox.data5b)
rownames(wilcox.data5b) <- colnames(Abund_simplewilcox_filt)
#Perform multiple comparison correction using a given method of choice
wilcox.data5b$rel.fdr <- p.adjust(wilcox.data5b$wilcox.data5b, method="fdr")
#incorporate other metrics of data5b centre and dispersion
wilcox.data5b$mean <- t(summarise_each(Abund_simplewilcox_filt,funs(mean)))
wilcox.data5b$median <- t(summarise_each(Abund_simplewilcox_filt,funs(median)))
wilcox.data5b$variance <- t(summarise_each(Abund_simplewilcox_filt,funs(var))) 
wilcox.data5b$IQR <- t(summarise_each(Abund_simplewilcox_filt,funs(IQR)))
wilcox.data5b$Taxa <- row.names(wilcox.data5b)
wilcox.data5b$Subset1 <- c("2") #Donor7
wilcox.data5b$Subset2 <- c("R") #Recipient

#Donor20 Recipients
meta_wilcox_filt = filter(metaIgA, SampleType=="PostTransplant"&Donor.ID2=="Donor20")
samples_kept <- meta_wilcox_filt$SampleID
IgA_simplewilcox_filt <- filter(IgA_simplewilcox, rownames(IgA_simplewilcox) %in% samples_kept)

#apply wilcox test to test if Index is sig. diff. from 0
wilcox.data6 <- sapply(1:ncol(IgA_simplewilcox_filt), function(i){wilcox.test(IgA_simplewilcox_filt[,i], mu=0)$p.value})
wilcox.data6 <- as.data.frame(wilcox.data6)
rownames(wilcox.data6) <- colnames(IgA_simplewilcox_filt)
#Perform multiple comparison correction using a given method of choice
wilcox.data6$rel.fdr <- p.adjust(wilcox.data6$wilcox.data6, method="fdr")
#incorporate other metrics of data6 centre and dispersion
wilcox.data6$mean <- t(summarise_each(IgA_simplewilcox_filt,funs(mean)))
wilcox.data6$median <- t(summarise_each(IgA_simplewilcox_filt,funs(median)))
wilcox.data6$variance <- t(summarise_each(IgA_simplewilcox_filt,funs(var))) 
wilcox.data6$IQR <- t(summarise_each(IgA_simplewilcox_filt,funs(IQR)))
wilcox.data6$Taxa <- row.names(wilcox.data6)
wilcox.data6$Subset1 <- c("3") #Donor20
wilcox.data6$Subset2 <- c("R") #Recipient

#Selecting the most targeted taxa; pval<0.05, median index >0
most_targeted_donor20 <- dplyr::filter(wilcox.data6, wilcox.data6<0.05&median>0)
most_targeted_donor20$Taxa
```

```
## [1] "|Bacteria|Proteobacteria|Gammaproteobacteria|Enterobacteriales|Enterobacteriaceae|Escherichia|coli|1b158b8b2922d4fcad5d9cea607cbb7d"
## [2] "|Bacteria|Firmicutes|Clostridia|Clostridiales|Lachnospiraceae|[Ruminococcus]|torques|e59405a47acbc248ce61395366159d8d"
```

```
#abundance
Abund_simplewilcox_filt <- filter(Abund_simplewilcox, rownames(Abund_simplewilcox) %in% samples_kept)

#apply wilcox test to test if Index is sig. diff. from 0
wilcox.data6b <- sapply(1:ncol(Abund_simplewilcox_filt), function(i){wilcox.test(Abund_simplewilcox_filt[,i], mu=0)$p.value})
wilcox.data6b <- as.data.frame(wilcox.data6b)
rownames(wilcox.data6b) <- colnames(Abund_simplewilcox_filt)
#Perform multiple comparison correction using a given method of choice
wilcox.data6b$rel.fdr <- p.adjust(wilcox.data6b$wilcox.data6b, method="fdr")
#incorporate other metrics of data6b centre and dispersion
wilcox.data6b$mean <- t(summarise_each(Abund_simplewilcox_filt,funs(mean)))
wilcox.data6b$median <- t(summarise_each(Abund_simplewilcox_filt,funs(median)))
wilcox.data6b$variance <- t(summarise_each(Abund_simplewilcox_filt,funs(var))) 
wilcox.data6b$IQR <- t(summarise_each(Abund_simplewilcox_filt,funs(IQR)))
wilcox.data6b$Taxa <- row.names(wilcox.data6b)
wilcox.data6b$Subset1 <- c("3") #Donor20
wilcox.data6b$Subset2 <- c("R") #Recipient


#Donor 2 itself
meta_wilcox_filt = filter(metaIgA, Patient=="Donor2")
samples_kept <- meta_wilcox_filt$SampleID
IgA_simplewilcox_filt <- filter(IgA_simplewilcox, rownames(IgA_simplewilcox) %in% samples_kept)

#apply wilcox test to test if Index is sig. diff. from 0
wilcox.data8 <- sapply(1:ncol(IgA_simplewilcox_filt), function(i){wilcox.test(IgA_simplewilcox_filt[,i], mu=0)$p.value})
wilcox.data8 <- as.data.frame(wilcox.data8)
rownames(wilcox.data8) <- colnames(IgA_simplewilcox_filt)
#incorporate other metrics of data8 centre and dispersion
wilcox.data8$mean <- t(summarise_each(IgA_simplewilcox_filt,funs(mean)))
wilcox.data8$median <- t(summarise_each(IgA_simplewilcox_filt,funs(median)))
wilcox.data8$Taxa <- row.names(wilcox.data8)
wilcox.data8$Subset1 <- c("1") #Donor2
wilcox.data8$Subset2 <- c("D") #Donor

wilcox.data8[which(wilcox.data8$median>0),]$Taxa
```

```
##  [1] "|Bacteria|Bacteroidetes|Bacteroidia|Bacteroidales|Porphyromonadaceae|Parabacteroides|distasonis|2cdb259b754c2db622ed9fb5a6517a37"          
##  [2] "|Bacteria|Bacteroidetes|Bacteroidia|Bacteroidales|Bacteroidaceae|Bacteroides|ovatus|03af966ff07ddef2b87da992b85b600b"                      
##  [3] "|Bacteria|Bacteroidetes|Bacteroidia|Bacteroidales|Bacteroidaceae|Bacteroides|NA|51e441cbdcc80da0656e82293ae160b5"                          
##  [4] "|Bacteria|Bacteroidetes|Bacteroidia|Bacteroidales|Bacteroidaceae|Bacteroides|NA|b65eb19257f7a2bedb5a1c4b42aeb396"                          
##  [5] "|Bacteria|Bacteroidetes|Bacteroidia|Bacteroidales|Rikenellaceae|NA|NA|e47a63686b619f67783f9b9aa52b86bf"                                    
##  [6] "|Bacteria|Bacteroidetes|Bacteroidia|Bacteroidales|Rikenellaceae|NA|NA|619f64f2bf103286f4f70bfd89500ed4"                                    
##  [7] "|Bacteria|Bacteroidetes|Bacteroidia|Bacteroidales|Rikenellaceae|NA|NA|7534fb513a4b404419edc4e91920af3f"                                    
##  [8] "|Bacteria|Bacteroidetes|[Saprospirae]|[Saprospirales]|Chitinophagaceae|Sediminibacterium|NA|372e9cfd6acc38cd00e88aac575f8afa"              
##  [9] "|Bacteria|Bacteroidetes|[Saprospirae]|[Saprospirales]|Chitinophagaceae|Sediminibacterium|NA|a47ed77e3a3ab04be7139730ee5e5a98"              
## [10] "|Bacteria|Chlamydiae|Chlamydiia|Chlamydiales|Parachlamydiaceae|Parachlamydia|NA|8a592a80ca5be07c6b8d28ae3f835dcd"                          
## [11] "|Bacteria|Proteobacteria|Betaproteobacteria|Burkholderiales|Oxalobacteraceae|Ralstonia|NA|dde6d3e36af306c913854a9cf0ddf248"                
## [12] "|Bacteria|Proteobacteria|Gammaproteobacteria|Enterobacteriales|Enterobacteriaceae|NA|NA|bfe54af4c9180d37a0d76f6dafe79a5a"                  
## [13] "|Bacteria|Proteobacteria|Gammaproteobacteria|Xanthomonadales|Sinobacteraceae|NA|NA|09bedf1ee9478654b581fea611f919e7"                       
## [14] "|Bacteria|Proteobacteria|Gammaproteobacteria|Pseudomonadales|Pseudomonadaceae|NA|NA|1f878f615fcfc8d7bd381a7841ac1e41"                      
## [15] "|Bacteria|Proteobacteria|Gammaproteobacteria|Pseudomonadales|Pseudomonadaceae|Pseudomonas|veronii|402e5913597695a16d7cad415ffff02f"        
## [16] "|Bacteria|Proteobacteria|Deltaproteobacteria|Desulfovibrionales|Desulfovibrionaceae|Bilophila|NA|ece50a62168f85fc61385d8adb4c6494"         
## [17] "|Bacteria|Actinobacteria|Coriobacteriia|Coriobacteriales|Coriobacteriaceae|Collinsella|aerofaciens|dfba68ef0fd0e712608eb2a0078013a7"       
## [18] "|Bacteria|Actinobacteria|Actinobacteria|Actinomycetales|NA|NA|NA|bb6c24bb90dd84f577f7488df6312451"                                         
## [19] "|Bacteria|Actinobacteria|Actinobacteria|Bifidobacteriales|Bifidobacteriaceae|Bifidobacterium|NA|7b223719a0af567e7ea99f06f7ea1068"          
## [20] "|Bacteria|Actinobacteria|Actinobacteria|Bifidobacteriales|Bifidobacteriaceae|Bifidobacterium|NA|69e611251f4d8582e312afa5737f033e"          
## [21] "|Bacteria|Actinobacteria|Actinobacteria|Bifidobacteriales|Bifidobacteriaceae|Bifidobacterium|adolescentis|554c761996ebab999befda1b695fd81d"
## [22] "|Bacteria|Actinobacteria|Actinobacteria|Actinomycetales|Actinomycetaceae|Actinomyces|NA|608e6548b1b4cbb6176c8fce090991a5"                  
## [23] "|Bacteria|Firmicutes|Bacilli|Lactobacillales|Streptococcaceae|Streptococcus|NA|73bf8d1a5983e34a0cb84e3cae127815"                           
## [24] "|Bacteria|Firmicutes|Bacilli|Lactobacillales|Carnobacteriaceae|Granulicatella|NA|24a60c6448e70d9198ad6ba93520958c"                         
## [25] "|Bacteria|Firmicutes|Bacilli|Gemellales|Gemellaceae|NA|NA|cda4e6f933bb3108ea3e92f9db411c00"                                                
## [26] "|Bacteria|Firmicutes|Clostridia|Clostridiales|Veillonellaceae|Phascolarctobacterium|NA|e5413f67faa6b8c0c3e63e48836c0b42"                   
## [27] "|Bacteria|Firmicutes|Clostridia|Clostridiales|Veillonellaceae|Megasphaera|NA|2f93e58b78f2842e83abd5fde37ad276"                             
## [28] "|Bacteria|Firmicutes|Clostridia|Clostridiales|Veillonellaceae|Veillonella|dispar|5608c3e6c9de9ceb79610e7786bd0ac4"                         
## [29] "|Bacteria|Firmicutes|Clostridia|Clostridiales|Veillonellaceae|Veillonella|dispar|5b4f8b625d8fbb1268863be7dbc4db5d"                         
## [30] "|Bacteria|Firmicutes|Erysipelotrichi|Erysipelotrichales|Erysipelotrichaceae|NA|NA|f95cab37fba4160de15015f4d520839f"                        
## [31] "|Bacteria|Firmicutes|Erysipelotrichi|Erysipelotrichales|Erysipelotrichaceae|NA|NA|0c3887592dd60a361d43c78e501ba495"                        
## [32] "|Bacteria|Firmicutes|Erysipelotrichi|Erysipelotrichales|Erysipelotrichaceae|Bulleidia|moorei|e083e2f58987c5f8db5d4dd16ddde91f"             
## [33] "|Bacteria|Firmicutes|Clostridia|Clostridiales|NA|NA|NA|df15aa00cffdc5235078831e54deb6db"                                                   
## [34] "|Bacteria|Firmicutes|Clostridia|Clostridiales|Clostridiaceae|Clostridium|NA|5c82dc47435864e490625ae01151570e"                              
## [35] "|Bacteria|Firmicutes|Clostridia|Clostridiales|Ruminococcaceae|NA|NA|0437c9910becc153a3d7838fd8eaa64b"                                      
## [36] "|Bacteria|Firmicutes|Clostridia|Clostridiales|Ruminococcaceae|NA|NA|26f6853f46b06854fe5418317a261fb8"                                      
## [37] "|Bacteria|Firmicutes|Clostridia|Clostridiales|Ruminococcaceae|Oscillospira|NA|a7283edda8770d12b4e25d4dcce112c4"                            
## [38] "|Bacteria|Firmicutes|Clostridia|Clostridiales|Ruminococcaceae|Oscillospira|NA|e13823e500387439450b3826ea191948"                            
## [39] "|Bacteria|Firmicutes|Clostridia|Clostridiales|Ruminococcaceae|Oscillospira|NA|680d83cb233cffcc6405e08c46982042"                            
## [40] "|Bacteria|Firmicutes|Clostridia|Clostridiales|Ruminococcaceae|NA|NA|c4995645e0c1545b0e1620144d03772b"                                      
## [41] "|Bacteria|Firmicutes|Clostridia|Clostridiales|Ruminococcaceae|Oscillospira|NA|f2c958dfcac880c2f53a47e114d994d8"                            
## [42] "|Bacteria|Firmicutes|Clostridia|Clostridiales|Ruminococcaceae|Ruminococcus|bromii|fc02af89d869043b296114729009a5b0"                        
## [43] "|Bacteria|Firmicutes|Clostridia|Clostridiales|Ruminococcaceae|Ruminococcus|NA|d048172b7b49bf1669197f10389312cd"                            
## [44] "|Bacteria|Firmicutes|Clostridia|Clostridiales|Ruminococcaceae|Ruminococcus|NA|8aa94826eb4f90512d52fc7b796354c6"                            
## [45] "|Bacteria|Firmicutes|Clostridia|Clostridiales|Ruminococcaceae|Faecalibacterium|prausnitzii|a1a300c872047a7d8b6b294b17cfb63b"               
## [46] "|Bacteria|Firmicutes|Clostridia|Clostridiales|Ruminococcaceae|Faecalibacterium|prausnitzii|c3bdda568b2c1580d5cce7407ef43909"               
## [47] "|Bacteria|Firmicutes|Clostridia|Clostridiales|Lachnospiraceae|[Ruminococcus]|gnavus|90a05d597112b554e4480a8eaae4e0aa"                      
## [48] "|Bacteria|Firmicutes|Clostridia|Clostridiales|Lachnospiraceae|[Ruminococcus]|gnavus|cc2d96099f530b503371e5ddca8c0a58"                      
## [49] "|Bacteria|Firmicutes|Clostridia|Clostridiales|Lachnospiraceae|NA|NA|8b8b93b27e9c6cc58aacb0bf38248700"                                      
## [50] "|Bacteria|Firmicutes|Clostridia|Clostridiales|Lachnospiraceae|NA|NA|ac04fef6eb13ddf89756a0af35b512fe"                                      
## [51] "|Bacteria|Firmicutes|Clostridia|Clostridiales|Lachnospiraceae|Roseburia|faecis|2d34c22edce4b1f2d8a5228ad78f8ea8"                           
## [52] "|Bacteria|Firmicutes|Clostridia|Clostridiales|Lachnospiraceae|Coprococcus|NA|58c9d27620c4a8f749c75f945fce11a0"                             
## [53] "|Bacteria|Firmicutes|Clostridia|Clostridiales|Lachnospiraceae|Coprococcus|NA|cd287f4187715ce042afa01534b95a9b"                             
## [54] "|Bacteria|Firmicutes|Clostridia|Clostridiales|Lachnospiraceae|Lachnospira|NA|8e90ac2f0df6304022cff8cd278f8218"                             
## [55] "|Bacteria|Firmicutes|Clostridia|Clostridiales|Lachnospiraceae|NA|NA|f54512749b97be9497134dc28c0af837"                                      
## [56] "|Bacteria|Firmicutes|Clostridia|Clostridiales|Lachnospiraceae|NA|NA|b20c095fd654b84cebdbfe4faa0a1409"                                      
## [57] "|Bacteria|Firmicutes|Clostridia|Clostridiales|Lachnospiraceae|NA|NA|d5c7d97e6f4f5789d574d321dcca0992"                                      
## [58] "|Bacteria|Firmicutes|Clostridia|Clostridiales|Lachnospiraceae|[Ruminococcus]|NA|6a6fcf8f9b8bb1ab9e5f8456ee7fb109"                          
## [59] "|Bacteria|Firmicutes|Clostridia|Clostridiales|Lachnospiraceae|[Ruminococcus]|torques|e59405a47acbc248ce61395366159d8d"                     
## [60] "|Bacteria|Firmicutes|Clostridia|Clostridiales|Lachnospiraceae|NA|NA|694d61b8ec78349749c8b6ea59938e0b"                                      
## [61] "|Bacteria|Firmicutes|Clostridia|Clostridiales|Lachnospiraceae|Blautia|NA|7082034a5eec4c381d09cd380594242e"                                 
## [62] "|Bacteria|Firmicutes|Clostridia|Clostridiales|Lachnospiraceae|NA|NA|eb61cae65bc6cdd2440323bbf603ba5c"                                      
## [63] "|Bacteria|Firmicutes|Clostridia|Clostridiales|Lachnospiraceae|NA|NA|d9681bf8373e2635323f1ca0cc434ecf"                                      
## [64] "|Bacteria|Firmicutes|Clostridia|Clostridiales|Lachnospiraceae|Dorea|NA|a0b7d83fb64749f9a4b15b3728425b97"                                   
## [65] "|Bacteria|Firmicutes|Clostridia|Clostridiales|Lachnospiraceae|Dorea|NA|9d21d7d00d7e938a7e1c374a4f4bb833"
```

```
#abundance
Abund_simplewilcox_filt <- filter(Abund_simplewilcox, rownames(Abund_simplewilcox) %in% samples_kept)

#apply wilcox test to test if Index is sig. diff. from 0
wilcox.data8b <- sapply(1:ncol(Abund_simplewilcox_filt), function(i){wilcox.test(Abund_simplewilcox_filt[,i], mu=0)$p.value})
wilcox.data8b <- as.data.frame(wilcox.data8b)
rownames(wilcox.data8b) <- colnames(Abund_simplewilcox_filt)
#Perform multiple comparison correction using a given method of choice
wilcox.data8b$rel.fdr <- p.adjust(wilcox.data8b$wilcox.data8b, method="fdr")
#incorporate other metrics of data8b centre and dispersion
wilcox.data8b$mean <- t(summarise_each(Abund_simplewilcox_filt,funs(mean)))
wilcox.data8b$median <- t(summarise_each(Abund_simplewilcox_filt,funs(median)))
wilcox.data8b$variance <- t(summarise_each(Abund_simplewilcox_filt,funs(var))) 
wilcox.data8b$IQR <- t(summarise_each(Abund_simplewilcox_filt,funs(IQR)))
wilcox.data8b$Taxa <- row.names(wilcox.data8b)
wilcox.data8b$Subset1 <- c("1") #Donor2
wilcox.data8b$Subset2 <- c("D") #Donor

#Donor 7 itself
meta_wilcox_filt = filter(metaIgA, Patient=="Donor7")
samples_kept <- meta_wilcox_filt$SampleID
IgA_simplewilcox_filt <- filter(IgA_simplewilcox, rownames(IgA_simplewilcox) %in% samples_kept)

#apply wilcox test to test if Index is sig. diff. from 0
wilcox.data9 <- sapply(1:ncol(IgA_simplewilcox_filt), function(i){wilcox.test(IgA_simplewilcox_filt[,i], mu=0)$p.value})
wilcox.data9 <- as.data.frame(wilcox.data9)
rownames(wilcox.data9) <- colnames(IgA_simplewilcox_filt)
#incorporate other metrics of data9 centre and dispersion
wilcox.data9$mean <- t(summarise_each(IgA_simplewilcox_filt,funs(mean)))
wilcox.data9$median <- t(summarise_each(IgA_simplewilcox_filt,funs(median)))
wilcox.data9$Taxa <- row.names(wilcox.data9)
wilcox.data9$Subset1 <- c("2") #Donor7
wilcox.data9$Subset2 <- c("D") #Donor 
wilcox.data9[which(wilcox.data9$median>0),]$Taxa
```

```
##  [1] "|Bacteria|Bacteroidetes|Bacteroidia|Bacteroidales|[Barnesiellaceae]|NA|NA|5b0c52a1507deba61b41b7f9d8187386"                        
##  [2] "|Bacteria|Bacteroidetes|Bacteroidia|Bacteroidales|[Barnesiellaceae]|NA|NA|9d0579ce6a317ca0e626a5c68981c725"                        
##  [3] "|Bacteria|Bacteroidetes|Bacteroidia|Bacteroidales|[Barnesiellaceae]|NA|NA|e6cd2fee507eb45ff5f3c0a4191f3876"                        
##  [4] "|Bacteria|Bacteroidetes|Bacteroidia|Bacteroidales|[Barnesiellaceae]|NA|NA|158782cefa6e28406a2a0aeb75220f0a"                        
##  [5] "|Bacteria|Bacteroidetes|Bacteroidia|Bacteroidales|Bacteroidaceae|Bacteroides|ovatus|03af966ff07ddef2b87da992b85b600b"              
##  [6] "|Bacteria|Bacteroidetes|Bacteroidia|Bacteroidales|Bacteroidaceae|Bacteroides|NA|51e441cbdcc80da0656e82293ae160b5"                  
##  [7] "|Bacteria|Bacteroidetes|Bacteroidia|Bacteroidales|Bacteroidaceae|Bacteroides|caccae|ec4075339e16f5cd45fd5a7955596899"              
##  [8] "|Bacteria|Bacteroidetes|Bacteroidia|Bacteroidales|Bacteroidaceae|Bacteroides|NA|25d727166a36df8d2f6a915a945bf5ac"                  
##  [9] "|Bacteria|Bacteroidetes|Bacteroidia|Bacteroidales|Bacteroidaceae|Bacteroides|eggerthii|8ad4a56a5f526df40d97b371ba0ef33e"           
## [10] "|Bacteria|Bacteroidetes|Bacteroidia|Bacteroidales|Bacteroidaceae|Bacteroides|eggerthii|30dd9fd45122f82c12b166a042d1eaf9"           
## [11] "|Bacteria|Bacteroidetes|Bacteroidia|Bacteroidales|Bacteroidaceae|Bacteroides|uniformis|63b26504f32377cd78d6068bffb86b9a"           
## [12] "|Bacteria|Bacteroidetes|Bacteroidia|Bacteroidales|[Paraprevotellaceae]|NA|NA|85fddfaca6687f22a2b1c5ed45a6041c"                     
## [13] "|Bacteria|Bacteroidetes|Bacteroidia|Bacteroidales|[Paraprevotellaceae]|Paraprevotella|NA|37dac50f454a3cf754e83790eee77108"         
## [14] "|Bacteria|Bacteroidetes|Bacteroidia|Bacteroidales|[Odoribacteraceae]|Butyricimonas|NA|0bb0e1802451aace7137a4d598f775f5"            
## [15] "|Bacteria|Bacteroidetes|Bacteroidia|Bacteroidales|[Odoribacteraceae]|Odoribacter|NA|3ea67969a01ec419ec1d4784d379ad1d"              
## [16] "|Bacteria|Bacteroidetes|Bacteroidia|Bacteroidales|Rikenellaceae|NA|NA|e47a63686b619f67783f9b9aa52b86bf"                            
## [17] "|Bacteria|Bacteroidetes|Bacteroidia|Bacteroidales|Rikenellaceae|NA|NA|a231814083060142816974207844eff2"                            
## [18] "|Bacteria|Bacteroidetes|[Saprospirae]|[Saprospirales]|Chitinophagaceae|Sediminibacterium|NA|a47ed77e3a3ab04be7139730ee5e5a98"      
## [19] "|Bacteria|Bacteroidetes|[Saprospirae]|[Saprospirales]|Chitinophagaceae|Sediminibacterium|NA|1ee27fee30b2e6832a2be5e28f0978f3"      
## [20] "|Bacteria|Proteobacteria|Gammaproteobacteria|Enterobacteriales|Enterobacteriaceae|NA|NA|677929eac52be081cdae08a0b9c70eb2"          
## [21] "|Bacteria|Proteobacteria|Gammaproteobacteria|Enterobacteriales|Enterobacteriaceae|NA|NA|bfe54af4c9180d37a0d76f6dafe79a5a"          
## [22] "|Bacteria|Proteobacteria|Gammaproteobacteria|Xanthomonadales|Xanthomonadaceae|Stenotrophomonas|NA|891019e319f5d8d088ed4f9379fd63ef"
## [23] "|Bacteria|Proteobacteria|Deltaproteobacteria|Desulfovibrionales|Desulfovibrionaceae|Bilophila|NA|ece50a62168f85fc61385d8adb4c6494" 
## [24] "|Bacteria|Actinobacteria|Actinobacteria|Actinomycetales|Brevibacteriaceae|Brevibacterium|aureum|2861b60feb98b96bfdfae4152ea84ae7"  
## [25] "|Bacteria|Actinobacteria|Actinobacteria|Actinomycetales|Pseudonocardiaceae|Pseudonocardia|NA|78c07a3480fde312c999698b581e8c5f"     
## [26] "|Bacteria|Firmicutes|Clostridia|Clostridiales|Veillonellaceae|Dialister|NA|520c77820886daeb8cf0d6497cfb1344"                       
## [27] "|Bacteria|Firmicutes|Erysipelotrichi|Erysipelotrichales|Erysipelotrichaceae|NA|NA|f95cab37fba4160de15015f4d520839f"                
## [28] "|Bacteria|Firmicutes|Erysipelotrichi|Erysipelotrichales|Erysipelotrichaceae|NA|NA|0c3887592dd60a361d43c78e501ba495"                
## [29] "|Bacteria|Firmicutes|Erysipelotrichi|Erysipelotrichales|Erysipelotrichaceae|Holdemania|NA|bc53814d35b2f862562a1e865e923b33"        
## [30] "|Bacteria|Firmicutes|Clostridia|Clostridiales|NA|NA|NA|fd2130b6a8a1db45c0f5381012b2e5e7"                                           
## [31] "|Bacteria|Firmicutes|Clostridia|Clostridiales|NA|NA|NA|6b40524e525db04087e414f058bc1a5e"                                           
## [32] "|Bacteria|Firmicutes|Clostridia|Clostridiales|NA|NA|NA|d917386c5f307a4f652e389dadd7cef5"                                           
## [33] "|Bacteria|Firmicutes|Clostridia|Clostridiales|NA|NA|NA|dff390a58455e2599a43c5e400a35518"                                           
## [34] "|Bacteria|Firmicutes|Clostridia|Clostridiales|Ruminococcaceae|NA|NA|c4995645e0c1545b0e1620144d03772b"                              
## [35] "|Bacteria|Firmicutes|Clostridia|Clostridiales|Ruminococcaceae|Oscillospira|NA|3e2bfdd1acae4723c96e344f91b262d8"                    
## [36] "|Bacteria|Firmicutes|Clostridia|Clostridiales|Ruminococcaceae|NA|NA|2673143ffb9d8ff3ad82fa40e325973c"                              
## [37] "|Bacteria|Firmicutes|Clostridia|Clostridiales|Ruminococcaceae|Faecalibacterium|prausnitzii|7859f0d526ad56fb4d8383d136b3bae6"       
## [38] "|Bacteria|Firmicutes|Clostridia|Clostridiales|Ruminococcaceae|Faecalibacterium|prausnitzii|c3bdda568b2c1580d5cce7407ef43909"       
## [39] "|Bacteria|Firmicutes|Clostridia|Clostridiales|Ruminococcaceae|Faecalibacterium|prausnitzii|a3f36ef32153f2fc2aaeac2feb23777f"       
## [40] "|Bacteria|Firmicutes|Clostridia|Clostridiales|Lachnospiraceae|NA|NA|bc8fb99545fba27899a16f1ade967276"                              
## [41] "|Bacteria|Firmicutes|Clostridia|Clostridiales|Lachnospiraceae|NA|NA|8b8b93b27e9c6cc58aacb0bf38248700"                              
## [42] "|Bacteria|Firmicutes|Clostridia|Clostridiales|Lachnospiraceae|Anaerostipes|NA|75622b8ee0f6a2b8a796bbfd264ca9fa"                    
## [43] "|Bacteria|Firmicutes|Clostridia|Clostridiales|Lachnospiraceae|Roseburia|NA|c335a7c6e1e3f03c2c532274ec80a0c5"                       
## [44] "|Bacteria|Firmicutes|Clostridia|Clostridiales|Lachnospiraceae|Roseburia|NA|7d285be20e3ad3812eb21be379357ef1"                       
## [45] "|Bacteria|Firmicutes|Clostridia|Clostridiales|Lachnospiraceae|Roseburia|faecis|2d34c22edce4b1f2d8a5228ad78f8ea8"                   
## [46] "|Bacteria|Firmicutes|Clostridia|Clostridiales|Lachnospiraceae|Coprococcus|NA|58c9d27620c4a8f749c75f945fce11a0"                     
## [47] "|Bacteria|Firmicutes|Clostridia|Clostridiales|Lachnospiraceae|Coprococcus|eutactus|53ee2a5c83806c73f3e5419690ac4241"               
## [48] "|Bacteria|Firmicutes|Clostridia|Clostridiales|Lachnospiraceae|Lachnospira|NA|5610b04f19aaac78ac2469a3feb707e9"                     
## [49] "|Bacteria|Firmicutes|Clostridia|Clostridiales|Lachnospiraceae|Lachnospira|NA|d32f24bb89dd024a38ac0a7a5962c771"                     
## [50] "|Bacteria|Firmicutes|Clostridia|Clostridiales|Lachnospiraceae|Coprococcus|NA|00979c6b417b7978c1c656e263cdd054"                     
## [51] "|Bacteria|Firmicutes|Clostridia|Clostridiales|Lachnospiraceae|NA|NA|fcd2073fc9628403d2dc4aa2b66b64a5"                              
## [52] "|Bacteria|Firmicutes|Clostridia|Clostridiales|Lachnospiraceae|NA|NA|fb798da6858b8b3f34077164a4ec9315"                              
## [53] "|Bacteria|Firmicutes|Clostridia|Clostridiales|Lachnospiraceae|NA|NA|f54512749b97be9497134dc28c0af837"                              
## [54] "|Bacteria|Firmicutes|Clostridia|Clostridiales|Lachnospiraceae|NA|NA|b20c095fd654b84cebdbfe4faa0a1409"                              
## [55] "|Bacteria|Firmicutes|Clostridia|Clostridiales|Lachnospiraceae|[Ruminococcus]|NA|00565f9981f176f2eae21aff895a72ff"                  
## [56] "|Bacteria|Firmicutes|Clostridia|Clostridiales|Lachnospiraceae|[Ruminococcus]|NA|6a6fcf8f9b8bb1ab9e5f8456ee7fb109"                  
## [57] "|Bacteria|Firmicutes|Clostridia|Clostridiales|Lachnospiraceae|[Ruminococcus]|torques|e59405a47acbc248ce61395366159d8d"             
## [58] "|Bacteria|Firmicutes|Clostridia|Clostridiales|Lachnospiraceae|NA|NA|694d61b8ec78349749c8b6ea59938e0b"                              
## [59] "|Bacteria|Firmicutes|Clostridia|Clostridiales|Lachnospiraceae|Blautia|NA|c1dc9ad5116d96b8ed863458fc0d0aec"                         
## [60] "|Bacteria|Firmicutes|Clostridia|Clostridiales|Lachnospiraceae|Blautia|NA|7082034a5eec4c381d09cd380594242e"                         
## [61] "|Bacteria|Firmicutes|Clostridia|Clostridiales|Lachnospiraceae|NA|NA|ebbe8017bca36f0a3b02532a2b4ed0d2"                              
## [62] "|Bacteria|Firmicutes|Clostridia|Clostridiales|Lachnospiraceae|NA|NA|eb61cae65bc6cdd2440323bbf603ba5c"                              
## [63] "|Bacteria|Firmicutes|Clostridia|Clostridiales|Lachnospiraceae|NA|NA|d9681bf8373e2635323f1ca0cc434ecf"                              
## [64] "|Bacteria|Firmicutes|Clostridia|Clostridiales|Lachnospiraceae|Dorea|formicigenerans|afd87e82de329a1ed75b98b5b606843c"
```

```
#abundance
Abund_simplewilcox_filt <- filter(Abund_simplewilcox, rownames(Abund_simplewilcox) %in% samples_kept)

#apply wilcox test to test if Index is sig. diff. from 0
wilcox.data9b <- sapply(1:ncol(Abund_simplewilcox_filt), function(i){wilcox.test(Abund_simplewilcox_filt[,i], mu=0)$p.value})
wilcox.data9b <- as.data.frame(wilcox.data9b)
rownames(wilcox.data9b) <- colnames(Abund_simplewilcox_filt)
#Perform multiple comparison correction using a given method of choice
wilcox.data9b$rel.fdr <- p.adjust(wilcox.data9b$wilcox.data9b, method="fdr")
#incorporate other metrics of data9b centre and dispersion
wilcox.data9b$mean <- t(summarise_each(Abund_simplewilcox_filt,funs(mean)))
wilcox.data9b$median <- t(summarise_each(Abund_simplewilcox_filt,funs(median)))
wilcox.data9b$variance <- t(summarise_each(Abund_simplewilcox_filt,funs(var))) 
wilcox.data9b$IQR <- t(summarise_each(Abund_simplewilcox_filt,funs(IQR)))
wilcox.data9b$Taxa <- row.names(wilcox.data9b)
wilcox.data9b$Subset1 <- c("2") #Donor7
wilcox.data9b$Subset2 <- c("D") #Donor

#Donor 20 itself
meta_wilcox_filt = filter(metaIgA, Patient=="Donor20")
samples_kept <- meta_wilcox_filt$SampleID
IgA_simplewilcox_filt <- filter(IgA_simplewilcox, rownames(IgA_simplewilcox) %in% samples_kept)

#apply wilcox test to test if Index is sig. diff. from 0
wilcox.data10 <- sapply(1:ncol(IgA_simplewilcox_filt), function(i){wilcox.test(IgA_simplewilcox_filt[,i], mu=0)$p.value})
wilcox.data10 <- as.data.frame(wilcox.data10)
rownames(wilcox.data10) <- colnames(IgA_simplewilcox_filt)
#incorporate other metrics of data10 centre and dispersion
wilcox.data10$mean <- t(summarise_each(IgA_simplewilcox_filt,funs(mean)))
wilcox.data10$median <- t(summarise_each(IgA_simplewilcox_filt,funs(median)))
wilcox.data10$Taxa <- row.names(wilcox.data10)
wilcox.data10$Subset1 <- c("3") #Donor20
wilcox.data10$Subset2 <- c("D")

wilcox.data10[which(wilcox.data10$median>0),]$Taxa
```

```
##  [1] "|Bacteria|Bacteroidetes|Bacteroidia|Bacteroidales|Porphyromonadaceae|Parabacteroides|distasonis|f4f297232da0f8d7dae7f9c432501e22"              
##  [2] "|Bacteria|Bacteroidetes|Bacteroidia|Bacteroidales|Bacteroidaceae|Bacteroides|NA|25d727166a36df8d2f6a915a945bf5ac"                              
##  [3] "|Bacteria|Bacteroidetes|Sphingobacteriia|Sphingobacteriales|Sphingobacteriaceae|Pedobacter|NA|e5e45fe5960b838c45765068621efcd5"                
##  [4] "|Bacteria|Bacteroidetes|Bacteroidia|Bacteroidales|Rikenellaceae|NA|NA|76d4dadc006ff1d530d38dd3cfac300c"                                        
##  [5] "|Bacteria|Bacteroidetes|Bacteroidia|Bacteroidales|Rikenellaceae|NA|NA|e47a63686b619f67783f9b9aa52b86bf"                                        
##  [6] "|Bacteria|Chlamydiae|Chlamydiia|Chlamydiales|Parachlamydiaceae|Parachlamydia|NA|8a592a80ca5be07c6b8d28ae3f835dcd"                              
##  [7] "|Bacteria|Proteobacteria|Betaproteobacteria|Burkholderiales|Alcaligenaceae|Sutterella|NA|7d17d197d7978be0b6684209ce257e20"                     
##  [8] "|Bacteria|Proteobacteria|Gammaproteobacteria|Pseudomonadales|Pseudomonadaceae|NA|NA|1f878f615fcfc8d7bd381a7841ac1e41"                          
##  [9] "|Bacteria|Proteobacteria|Gammaproteobacteria|Pseudomonadales|Pseudomonadaceae|Pseudomonas|veronii|402e5913597695a16d7cad415ffff02f"            
## [10] "|Bacteria|Proteobacteria|Gammaproteobacteria|Xanthomonadales|Xanthomonadaceae|Stenotrophomonas|acidaminiphila|3588479e8e7443fdc3657f4b81cebf4c"
## [11] "|Bacteria|Proteobacteria|Gammaproteobacteria|Xanthomonadales|Xanthomonadaceae|Stenotrophomonas|NA|891019e319f5d8d088ed4f9379fd63ef"            
## [12] "|Bacteria|Actinobacteria|Actinobacteria|Actinomycetales|NA|NA|NA|bb6c24bb90dd84f577f7488df6312451"                                             
## [13] "|Bacteria|Actinobacteria|Actinobacteria|Bifidobacteriales|Bifidobacteriaceae|Bifidobacterium|NA|7b223719a0af567e7ea99f06f7ea1068"              
## [14] "|Bacteria|Actinobacteria|Actinobacteria|Actinomycetales|Actinomycetaceae|Actinomyces|NA|608e6548b1b4cbb6176c8fce090991a5"                      
## [15] "|Bacteria|Verrucomicrobia|Verrucomicrobiae|Verrucomicrobiales|Verrucomicrobiaceae|Akkermansia|muciniphila|8f98fb8693ed59c21399d83ce2d10724"    
## [16] "|Bacteria|Firmicutes|Bacilli|Lactobacillales|Streptococcaceae|Streptococcus|NA|bd2ebc70501f7d867c204f94c4e483da"                               
## [17] "|Bacteria|Firmicutes|Bacilli|Lactobacillales|Streptococcaceae|Streptococcus|NA|a5189f77a2cfeab3bc1602ff5c8ac3e9"                               
## [18] "|Bacteria|Firmicutes|Clostridia|Clostridiales|Veillonellaceae|Dialister|NA|520c77820886daeb8cf0d6497cfb1344"                                   
## [19] "|Bacteria|Firmicutes|Erysipelotrichi|Erysipelotrichales|Erysipelotrichaceae|Holdemania|NA|bc53814d35b2f862562a1e865e923b33"                    
## [20] "|Bacteria|Firmicutes|Clostridia|Clostridiales|Clostridiaceae|Clostridium|NA|5c82dc47435864e490625ae01151570e"                                  
## [21] "|Bacteria|Firmicutes|Clostridia|Clostridiales|Ruminococcaceae|NA|NA|26f6853f46b06854fe5418317a261fb8"                                          
## [22] "|Bacteria|Firmicutes|Clostridia|Clostridiales|Ruminococcaceae|Oscillospira|NA|9e1d23e474a9f1e4e10387a23f601dde"                                
## [23] "|Bacteria|Firmicutes|Clostridia|Clostridiales|Ruminococcaceae|NA|NA|c88e37c00a5abc483dada3afc5da38a3"                                          
## [24] "|Bacteria|Firmicutes|Clostridia|Clostridiales|Lachnospiraceae|NA|NA|bc8fb99545fba27899a16f1ade967276"                                          
## [25] "|Bacteria|Firmicutes|Clostridia|Clostridiales|Lachnospiraceae|NA|NA|8b8b93b27e9c6cc58aacb0bf38248700"                                          
## [26] "|Bacteria|Firmicutes|Clostridia|Clostridiales|Lachnospiraceae|NA|NA|ac04fef6eb13ddf89756a0af35b512fe"                                          
## [27] "|Bacteria|Firmicutes|Clostridia|Clostridiales|Lachnospiraceae|Roseburia|faecis|2d34c22edce4b1f2d8a5228ad78f8ea8"                               
## [28] "|Bacteria|Firmicutes|Clostridia|Clostridiales|Lachnospiraceae|Lachnospira|NA|3cfa76e0da34a56a641ca33770efb588"                                 
## [29] "|Bacteria|Firmicutes|Clostridia|Clostridiales|Lachnospiraceae|Coprococcus|NA|c9ea71f39bda8752713c8e90dff2b875"                                 
## [30] "|Bacteria|Firmicutes|Clostridia|Clostridiales|Lachnospiraceae|Coprococcus|NA|00979c6b417b7978c1c656e263cdd054"                                 
## [31] "|Bacteria|Firmicutes|Clostridia|Clostridiales|Lachnospiraceae|NA|NA|51dd453b71a4aba20d9f349601ea5203"                                          
## [32] "|Bacteria|Firmicutes|Clostridia|Clostridiales|Lachnospiraceae|[Ruminococcus]|torques|e59405a47acbc248ce61395366159d8d"                         
## [33] "|Bacteria|Firmicutes|Clostridia|Clostridiales|Lachnospiraceae|Blautia|NA|c1dc9ad5116d96b8ed863458fc0d0aec"                                     
## [34] "|Bacteria|Firmicutes|Clostridia|Clostridiales|Lachnospiraceae|NA|NA|eb61cae65bc6cdd2440323bbf603ba5c"                                          
## [35] "|Bacteria|Firmicutes|Clostridia|Clostridiales|Lachnospiraceae|Dorea|formicigenerans|afd87e82de329a1ed75b98b5b606843c"                          
## [36] "|Bacteria|Firmicutes|Clostridia|Clostridiales|Lachnospiraceae|NA|NA|392aa3c45e128c7432f91b3de84541f6"
```

```
#abundance
Abund_simplewilcox_filt <- filter(Abund_simplewilcox, rownames(Abund_simplewilcox) %in% samples_kept)

#apply wilcox test to test if Index is sig. diff. from 0
wilcox.data10b <- sapply(1:ncol(Abund_simplewilcox_filt), function(i){wilcox.test(Abund_simplewilcox_filt[,i], mu=0)$p.value})
wilcox.data10b <- as.data.frame(wilcox.data10b)
rownames(wilcox.data10b) <- colnames(Abund_simplewilcox_filt)
#Perform multiple comparison correction using a given method of choice
wilcox.data10b$rel.fdr <- p.adjust(wilcox.data10b$wilcox.data10b, method="fdr")
#incorporate other metrics of data10b centre and dispersion
wilcox.data10b$mean <- t(summarise_each(Abund_simplewilcox_filt,funs(mean)))
wilcox.data10b$median <- t(summarise_each(Abund_simplewilcox_filt,funs(median)))
wilcox.data10b$variance <- t(summarise_each(Abund_simplewilcox_filt,funs(var))) 
wilcox.data10b$IQR <- t(summarise_each(Abund_simplewilcox_filt,funs(IQR)))
wilcox.data10b$Taxa <- row.names(wilcox.data10b)
wilcox.data10b$Subset1 <- c("3") #Donor20
wilcox.data10b$Subset2 <- c("D") #Donor
```

Most targeted in capsule vs colonoscopy recipients, post-transplant

```
#count
dplyr::count(metaIgAPost, Delivery)
```

```
##   Delivery  n
## 1  Capsule 19
## 2    Colon 17
```

```
#Capsule Recipients
IgA_simplewilcox <- IgA_settrim75_t
meta_wilcox_filt = filter(metaIgA, SampleType=="PostTransplant"&Delivery=="Capsule")
samples_kept <- meta_wilcox_filt$SampleID
IgA_simplewilcox_filt <- filter(IgA_simplewilcox, rownames(IgA_simplewilcox) %in% samples_kept)

#apply wilcox test to test if Index is sig. diff. from 0
wilcox.data.cap <- sapply(1:ncol(IgA_simplewilcox_filt), function(i){wilcox.test(IgA_simplewilcox_filt[,i], mu=0)$p.value})
wilcox.data.cap <- as.data.frame(wilcox.data.cap)
rownames(wilcox.data.cap) <- colnames(IgA_simplewilcox_filt)
#Perform multiple comparison correction using a given method of choice
wilcox.data.cap$rel.fdr <- p.adjust(wilcox.data.cap$wilcox.data.cap, method="fdr")
#incorporate other metrics of data.cap centre and dispersion
wilcox.data.cap$mean <- t(summarise_each(IgA_simplewilcox_filt,funs(mean)))
wilcox.data.cap$median <- t(summarise_each(IgA_simplewilcox_filt,funs(median)))
wilcox.data.cap$variance <- t(summarise_each(IgA_simplewilcox_filt,funs(var))) 
wilcox.data.cap$IQR <- t(summarise_each(IgA_simplewilcox_filt,funs(IQR)))
wilcox.data.cap$Taxa <- row.names(wilcox.data.cap)
wilcox.data.cap$Subset <- c("Capsule")

#Selecting the most targeted taxa; FDR<0.05, median index >0
most_targeted_capsule <- dplyr::filter(wilcox.data.cap, wilcox.data.cap<0.05&median>0)
most_targeted_capsule$Taxa
```

```
## [1] "|Bacteria|Proteobacteria|Gammaproteobacteria|Enterobacteriales|Enterobacteriaceae|Escherichia|coli|1b158b8b2922d4fcad5d9cea607cbb7d"
## [2] "|Bacteria|Firmicutes|Clostridia|Clostridiales|Ruminococcaceae|NA|NA|63027647b049a274c881df23a4611b35"                               
## [3] "|Bacteria|Firmicutes|Clostridia|Clostridiales|Lachnospiraceae|[Ruminococcus]|torques|e59405a47acbc248ce61395366159d8d"              
## [4] "|Bacteria|Firmicutes|Clostridia|Clostridiales|Lachnospiraceae|NA|NA|694d61b8ec78349749c8b6ea59938e0b"
```

```
most_targeted_capsule_mean <- dplyr::filter(wilcox.data.cap, wilcox.data.cap<0.05&mean>0)
most_targeted_capsule_mean$Taxa
```

```
## [1] "|Bacteria|Proteobacteria|Gammaproteobacteria|Enterobacteriales|Enterobacteriaceae|Escherichia|coli|1b158b8b2922d4fcad5d9cea607cbb7d"
## [2] "|Bacteria|Firmicutes|Clostridia|Clostridiales|Ruminococcaceae|NA|NA|e803ff46adaa0fa149ef151b082378a0"                               
## [3] "|Bacteria|Firmicutes|Clostridia|Clostridiales|Ruminococcaceae|NA|NA|63027647b049a274c881df23a4611b35"                               
## [4] "|Bacteria|Firmicutes|Clostridia|Clostridiales|Lachnospiraceae|[Ruminococcus]|gnavus|cc2d96099f530b503371e5ddca8c0a58"               
## [5] "|Bacteria|Firmicutes|Clostridia|Clostridiales|Lachnospiraceae|[Ruminococcus]|torques|e59405a47acbc248ce61395366159d8d"              
## [6] "|Bacteria|Firmicutes|Clostridia|Clostridiales|Lachnospiraceae|NA|NA|694d61b8ec78349749c8b6ea59938e0b"
```

```
#abundance too
Abund_simplewilcox <- Abund_settrim75_t
Abund_simplewilcox_filt <- filter(Abund_simplewilcox, rownames(Abund_simplewilcox) %in% gsub("-", ".", samples_kept, fixed=TRUE))

wilcox.data.cap2 <- sapply(1:ncol(Abund_simplewilcox_filt), function(i){wilcox.test(Abund_simplewilcox_filt[,i], mu=0)$p.value})
wilcox.data.cap2 <- as.data.frame(wilcox.data.cap2)
rownames(wilcox.data.cap2) <- colnames(Abund_simplewilcox_filt)
#Perform multiple comparison correction using a given method of choice
wilcox.data.cap2$rel.fdr <- p.adjust(wilcox.data.cap2$wilcox.data.cap2, method="fdr")
#incorporate other metrics of data.cap centre and dispersion
wilcox.data.cap2$mean <- t(summarise_each(Abund_simplewilcox_filt,funs(mean)))
wilcox.data.cap2$median <- t(summarise_each(Abund_simplewilcox_filt,funs(median)))
wilcox.data.cap2$variance <- t(summarise_each(Abund_simplewilcox_filt,funs(var))) 
wilcox.data.cap2$IQR <- t(summarise_each(Abund_simplewilcox_filt,funs(IQR)))
wilcox.data.cap2$Taxa <- row.names(wilcox.data.cap2)
wilcox.data.cap2$Subset <- c("Capsule")


#Colonoscopy Recipients
meta_wilcox_filt = filter(metaIgA, SampleType=="PostTransplant"&Delivery=="Colon")
samples_kept <- meta_wilcox_filt$SampleID
IgA_simplewilcox_filt <- filter(IgA_simplewilcox, rownames(IgA_simplewilcox) %in% samples_kept)

#apply wilcox test to test if Index is sig. diff. from 0
wilcox.data.col <- sapply(1:ncol(IgA_simplewilcox_filt), function(i){wilcox.test(IgA_simplewilcox_filt[,i], mu=0)$p.value})
wilcox.data.col <- as.data.frame(wilcox.data.col)
rownames(wilcox.data.col) <- colnames(IgA_simplewilcox_filt)
#Perform multiple comparison correction using a given method of choice
wilcox.data.col$rel.fdr <- p.adjust(wilcox.data.col$wilcox.data.col, method="fdr")
#incorporate other metrics of data.col centre and dispersion
wilcox.data.col$mean <- t(summarise_each(IgA_simplewilcox_filt,funs(mean)))
wilcox.data.col$median <- t(summarise_each(IgA_simplewilcox_filt,funs(median)))
wilcox.data.col$variance <- t(summarise_each(IgA_simplewilcox_filt,funs(var))) 
wilcox.data.col$IQR <- t(summarise_each(IgA_simplewilcox_filt,funs(IQR)))
wilcox.data.col$Taxa <- row.names(wilcox.data.col)
wilcox.data.col$Subset <- c("Colonoscopy")

#Selecting the most targeted taxa; FDR<0.05, median index >0
most_targeted_colon <- dplyr::filter(wilcox.data.col, wilcox.data.col<0.05&median>0)
most_targeted_colon$Taxa
```

```
## [1] "|Bacteria|Bacteroidetes|Bacteroidia|Bacteroidales|Rikenellaceae|NA|NA|a231814083060142816974207844eff2"                             
## [2] "|Bacteria|Proteobacteria|Gammaproteobacteria|Enterobacteriales|Enterobacteriaceae|Escherichia|coli|1b158b8b2922d4fcad5d9cea607cbb7d"
## [3] "|Bacteria|Firmicutes|Clostridia|Clostridiales|Lachnospiraceae|[Ruminococcus]|gnavus|cc2d96099f530b503371e5ddca8c0a58"               
## [4] "|Bacteria|Firmicutes|Clostridia|Clostridiales|Lachnospiraceae|NA|NA|ac04fef6eb13ddf89756a0af35b512fe"                               
## [5] "|Bacteria|Firmicutes|Clostridia|Clostridiales|Lachnospiraceae|[Ruminococcus]|torques|e59405a47acbc248ce61395366159d8d"              
## [6] "|Bacteria|Firmicutes|Clostridia|Clostridiales|Lachnospiraceae|Dorea|NA|a0b7d83fb64749f9a4b15b3728425b97"
```

```
most_targeted_colon_mean <- dplyr::filter(wilcox.data.col, wilcox.data.col<0.05&mean>0)
most_targeted_colon_mean$Taxa
```

```
##  [1] "|Bacteria|Bacteroidetes|Bacteroidia|Bacteroidales|Rikenellaceae|NA|NA|a231814083060142816974207844eff2"                                    
##  [2] "|Bacteria|Proteobacteria|Gammaproteobacteria|Enterobacteriales|Enterobacteriaceae|Escherichia|coli|1b158b8b2922d4fcad5d9cea607cbb7d"       
##  [3] "|Bacteria|Actinobacteria|Actinobacteria|Bifidobacteriales|Bifidobacteriaceae|Bifidobacterium|NA|7b223719a0af567e7ea99f06f7ea1068"          
##  [4] "|Bacteria|Verrucomicrobia|Verrucomicrobiae|Verrucomicrobiales|Verrucomicrobiaceae|Akkermansia|muciniphila|8f98fb8693ed59c21399d83ce2d10724"
##  [5] "|Bacteria|Firmicutes|Clostridia|Clostridiales|Veillonellaceae|Veillonella|parvula|d3247c936f8f4735909a8526ebf2f49e"                        
##  [6] "|Bacteria|Firmicutes|Clostridia|Clostridiales|Ruminococcaceae|NA|NA|63027647b049a274c881df23a4611b35"                                      
##  [7] "|Bacteria|Firmicutes|Clostridia|Clostridiales|Lachnospiraceae|[Ruminococcus]|gnavus|cc2d96099f530b503371e5ddca8c0a58"                      
##  [8] "|Bacteria|Firmicutes|Clostridia|Clostridiales|Lachnospiraceae|NA|NA|ac04fef6eb13ddf89756a0af35b512fe"                                      
##  [9] "|Bacteria|Firmicutes|Clostridia|Clostridiales|Lachnospiraceae|[Ruminococcus]|torques|e59405a47acbc248ce61395366159d8d"                     
## [10] "|Bacteria|Firmicutes|Clostridia|Clostridiales|Lachnospiraceae|Dorea|NA|a0b7d83fb64749f9a4b15b3728425b97"
```

```
#Selecting the most targeted taxa; FDR<0.05, median index >0
most_targeted_capsule <- dplyr::filter(wilcox.data.cap, wilcox.data.cap<0.05&median>0)
most_targeted_capsule$Taxa
```

```
## [1] "|Bacteria|Proteobacteria|Gammaproteobacteria|Enterobacteriales|Enterobacteriaceae|Escherichia|coli|1b158b8b2922d4fcad5d9cea607cbb7d"
## [2] "|Bacteria|Firmicutes|Clostridia|Clostridiales|Ruminococcaceae|NA|NA|63027647b049a274c881df23a4611b35"                               
## [3] "|Bacteria|Firmicutes|Clostridia|Clostridiales|Lachnospiraceae|[Ruminococcus]|torques|e59405a47acbc248ce61395366159d8d"              
## [4] "|Bacteria|Firmicutes|Clostridia|Clostridiales|Lachnospiraceae|NA|NA|694d61b8ec78349749c8b6ea59938e0b"
```

```
most_targeted_capsule_mean <- dplyr::filter(wilcox.data.cap, wilcox.data.cap<0.05&mean>0)
most_targeted_capsule_mean$Taxa
```

```
## [1] "|Bacteria|Proteobacteria|Gammaproteobacteria|Enterobacteriales|Enterobacteriaceae|Escherichia|coli|1b158b8b2922d4fcad5d9cea607cbb7d"
## [2] "|Bacteria|Firmicutes|Clostridia|Clostridiales|Ruminococcaceae|NA|NA|e803ff46adaa0fa149ef151b082378a0"                               
## [3] "|Bacteria|Firmicutes|Clostridia|Clostridiales|Ruminococcaceae|NA|NA|63027647b049a274c881df23a4611b35"                               
## [4] "|Bacteria|Firmicutes|Clostridia|Clostridiales|Lachnospiraceae|[Ruminococcus]|gnavus|cc2d96099f530b503371e5ddca8c0a58"               
## [5] "|Bacteria|Firmicutes|Clostridia|Clostridiales|Lachnospiraceae|[Ruminococcus]|torques|e59405a47acbc248ce61395366159d8d"              
## [6] "|Bacteria|Firmicutes|Clostridia|Clostridiales|Lachnospiraceae|NA|NA|694d61b8ec78349749c8b6ea59938e0b"
```

```
#abundance too
Abund_simplewilcox <- Abund_settrim75_t
Abund_simplewilcox_filt <- filter(Abund_simplewilcox, rownames(Abund_simplewilcox) %in% gsub("-", ".", samples_kept, fixed=TRUE))

wilcox.data.col2 <- sapply(1:ncol(Abund_simplewilcox_filt), function(i){wilcox.test(Abund_simplewilcox_filt[,i], mu=0)$p.value})
wilcox.data.col2 <- as.data.frame(wilcox.data.col2)
rownames(wilcox.data.col2) <- colnames(Abund_simplewilcox_filt)
#Perform multiple comparison correction using a given method of choice
wilcox.data.col2$rel.fdr <- p.adjust(wilcox.data.col2$wilcox.data.col2, method="fdr")
#incorporate other metrics of data.cap centre and dispersion
wilcox.data.col2$mean <- t(summarise_each(Abund_simplewilcox_filt,funs(mean)))
wilcox.data.col2$median <- t(summarise_each(Abund_simplewilcox_filt,funs(median)))
wilcox.data.col2$variance <- t(summarise_each(Abund_simplewilcox_filt,funs(var))) 
wilcox.data.col2$IQR <- t(summarise_each(Abund_simplewilcox_filt,funs(IQR)))
wilcox.data.col2$Taxa <- row.names(wilcox.data.cap)
wilcox.data.col2$Subset <- c("Colonoscopy")
```

Tile plots capsule vs colonoscopy

```
###make a bubble plot of all targeted taxa with median >0 and p<0.05 
names(wilcox.data.cap)[names(wilcox.data.cap) == 'wilcox.data.cap'] <- 'p.val'
names(wilcox.data.col)[names(wilcox.data.col) == 'wilcox.data.col'] <- 'p.val'

bubble.all <- Reduce(full_join, list(wilcox.data.cap, wilcox.data.col))
```

```
## Joining, by = c("p.val", "rel.fdr", "mean", "median", "variance", "IQR", "Taxa", "Subset")
```

```
#take raw p value rather than fdr correction
bubble.filter <- filter(bubble.all, Taxa %in% TaxaToKeep_main) #use the same taxa as Fig 1 (sig in either pre, post or donor)
bubble.filter <- full_join(bubble.filter, TaxaNames_main, by="Taxa") #similarly can use same taxa names

###bubble plot

#pdf("/Users/kelseyhuus/Dropbox/PhD/Manuscripts/Cdiff_IgA_paper/CdiffFinalAnalysis/Graphs2/TilePlot_Delivery.pdf", 
#    width =2.2, 
#    height = 6)
p <- ggplot(bubble.filter, aes(x=Subset, y=SimpleASV)) + geom_tile(aes(fill=mean))
p <- p + scale_fill_gradient2(low="deepskyblue4",
                              mid="white",
                              high="indianred4",
                              midpoint=0)
p <- p + theme(axis.text.x  = element_text(angle=90, vjust=0.5, hjust=1, size=12))
p <- p + scale_y_discrete(limits = rev(levels(bubble.filter$Taxa)))
p <- p + theme(strip.background = element_rect(fill="gray85"),
               panel.background = element_rect(fill="white"),
               panel.border = element_rect(colour="black", linetype="solid", fill="transparent") 
)
#p <- p + facet_grid(.~Subset, scales = "free", space = "free", 
#                    labeller=label_wrap_gen(width=2))
p <- p + guides(colour=FALSE, size=FALSE, shape=FALSE, fill=FALSE)
p
```

```
#dev.off()

#legend only
p <- ggplot(bubble.filter, aes(x=Subset, y=SimpleASV)) + geom_tile(aes(fill=mean))
p <- p + scale_fill_gradient2(low="deepskyblue4",
                              mid="white",
                              high="indianred4",
                              midpoint=0)
p
```

```
#Abundance tile plot
bubble.all2 <- Reduce(full_join, list(wilcox.data.cap2,wilcox.data.col2))
```

```
## Joining, by = c("rel.fdr", "mean", "median", "variance", "IQR", "Taxa", "Subset")
```

```
#simplify by keeping only Taxa with at least one non-zero value 
bubble.filter2 <- filter(bubble.all2, Taxa %in% TaxaToKeep_main)
bubble.filter2 <- full_join(bubble.filter2, TaxaNames_main, by="Taxa")

#pdf("/Users/kelseyhuus/Dropbox/PhD/Manuscripts/Cdiff_IgA_paper/CdiffFinalAnalysis/Graphs2/TilePlot_Delivery_Abund.pdf", 
##    width = 2.2, 
 #   height = 6)
p <- ggplot(bubble.filter2, aes(x=Subset, y=SimpleASV)) + geom_tile(aes(fill=mean))
p <- p + scale_fill_gradientn(colours=c("white","indianred4"),
                             values=c(0,0.1,0.9,1))
p <- p + theme(axis.text.x  = element_text(angle=90, vjust=0.5, hjust=1, size=12))
p <- p + scale_y_discrete(limits = rev(levels(bubble.filter$Taxa)))
p <- p + theme(strip.background = element_rect(fill="gray85"),
               panel.background = element_rect(fill="white"),
               panel.border = element_rect(colour="black", linetype="solid", fill="transparent") 
)
#p <- p + facet_grid(.~Subset, scales = "free", space = "free", 
#                    labeller=label_wrap_gen(width=2))
p <- p + guides(colour=FALSE, size=FALSE, shape=FALSE, fill=FALSE)
p
```

```
#dev.off()

#legend only
#p <- ggplot(bubble.filter2, aes(x=Subset, y=SimpleASV)) + geom_tile(aes(fill=mean))
#p <- p + scale_fill_gradientn(colours=c("white","indianred4"),
#                             values=c(0,0.1,0.9,1))
#p
```

#Correlation check between IgA Index and abundance in donors, pre and post

```
#from bubble plot section
wilcox.data1$Taxa==wilcox.data2$Taxa
```

```
##   [1] TRUE TRUE TRUE TRUE TRUE TRUE TRUE TRUE TRUE TRUE TRUE TRUE TRUE TRUE TRUE
##  [16] TRUE TRUE TRUE TRUE TRUE TRUE TRUE TRUE TRUE TRUE TRUE TRUE TRUE TRUE TRUE
##  [31] TRUE TRUE TRUE TRUE TRUE TRUE TRUE TRUE TRUE TRUE TRUE TRUE TRUE TRUE TRUE
##  [46] TRUE TRUE TRUE TRUE TRUE TRUE TRUE TRUE TRUE TRUE TRUE TRUE TRUE TRUE TRUE
##  [61] TRUE TRUE TRUE TRUE TRUE TRUE TRUE TRUE TRUE TRUE TRUE TRUE TRUE TRUE TRUE
##  [76] TRUE TRUE TRUE TRUE TRUE TRUE TRUE TRUE TRUE TRUE TRUE TRUE TRUE TRUE TRUE
##  [91] TRUE TRUE TRUE TRUE TRUE TRUE TRUE TRUE TRUE TRUE TRUE TRUE TRUE TRUE TRUE
## [106] TRUE TRUE TRUE TRUE TRUE TRUE TRUE TRUE TRUE TRUE TRUE TRUE TRUE TRUE TRUE
## [121] TRUE TRUE TRUE TRUE TRUE TRUE TRUE TRUE TRUE TRUE TRUE TRUE TRUE TRUE TRUE
## [136] TRUE TRUE TRUE TRUE TRUE TRUE TRUE TRUE TRUE TRUE TRUE TRUE TRUE TRUE TRUE
## [151] TRUE TRUE TRUE TRUE TRUE TRUE TRUE TRUE TRUE TRUE TRUE TRUE TRUE TRUE TRUE
## [166] TRUE TRUE TRUE TRUE TRUE TRUE TRUE TRUE TRUE TRUE TRUE TRUE TRUE TRUE TRUE
## [181] TRUE TRUE TRUE TRUE TRUE TRUE TRUE TRUE TRUE TRUE TRUE TRUE TRUE TRUE TRUE
## [196] TRUE TRUE
```

```
wilcox.data2$Taxa==wilcox.data3$Taxa
```

```
##   [1] TRUE TRUE TRUE TRUE TRUE TRUE TRUE TRUE TRUE TRUE TRUE TRUE TRUE TRUE TRUE
##  [16] TRUE TRUE TRUE TRUE TRUE TRUE TRUE TRUE TRUE TRUE TRUE TRUE TRUE TRUE TRUE
##  [31] TRUE TRUE TRUE TRUE TRUE TRUE TRUE TRUE TRUE TRUE TRUE TRUE TRUE TRUE TRUE
##  [46] TRUE TRUE TRUE TRUE TRUE TRUE TRUE TRUE TRUE TRUE TRUE TRUE TRUE TRUE TRUE
##  [61] TRUE TRUE TRUE TRUE TRUE TRUE TRUE TRUE TRUE TRUE TRUE TRUE TRUE TRUE TRUE
##  [76] TRUE TRUE TRUE TRUE TRUE TRUE TRUE TRUE TRUE TRUE TRUE TRUE TRUE TRUE TRUE
##  [91] TRUE TRUE TRUE TRUE TRUE TRUE TRUE TRUE TRUE TRUE TRUE TRUE TRUE TRUE TRUE
## [106] TRUE TRUE TRUE TRUE TRUE TRUE TRUE TRUE TRUE TRUE TRUE TRUE TRUE TRUE TRUE
## [121] TRUE TRUE TRUE TRUE TRUE TRUE TRUE TRUE TRUE TRUE TRUE TRUE TRUE TRUE TRUE
## [136] TRUE TRUE TRUE TRUE TRUE TRUE TRUE TRUE TRUE TRUE TRUE TRUE TRUE TRUE TRUE
## [151] TRUE TRUE TRUE TRUE TRUE TRUE TRUE TRUE TRUE TRUE TRUE TRUE TRUE TRUE TRUE
## [166] TRUE TRUE TRUE TRUE TRUE TRUE TRUE TRUE TRUE TRUE TRUE TRUE TRUE TRUE TRUE
## [181] TRUE TRUE TRUE TRUE TRUE TRUE TRUE TRUE TRUE TRUE TRUE TRUE TRUE TRUE TRUE
## [196] TRUE TRUE
```

```
head(wilcox.data1$Subset)
```

```
## [1] "Pre" "Pre" "Pre" "Pre" "Pre" "Pre"
```

```
head(wilcox.data2$Subset)
```

```
## [1] "Post" "Post" "Post" "Post" "Post" "Post"
```

```
head(wilcox.data3$Subset)
```

```
## [1] "Donor" "Donor" "Donor" "Donor" "Donor" "Donor"
```

```
pdata <- data.frame(mean_pre=wilcox.data1$mean,
                    mean_post=wilcox.data2$mean,
                    mean_donor=wilcox.data3$mean,
                    median_pre=wilcox.data1$median,
                    median_post=wilcox.data2$median,
                    median_donor=wilcox.data3$median,
                    Taxa=wilcox.data1$Taxa)

TaxaNames_main <- filter(IgTAX, IgTAX$combined %in% pdata$Taxa)
TaxaNames_main$combined==pdata$Taxa
```

```
##   [1] TRUE TRUE TRUE TRUE TRUE TRUE TRUE TRUE TRUE TRUE TRUE TRUE TRUE TRUE TRUE
##  [16] TRUE TRUE TRUE TRUE TRUE TRUE TRUE TRUE TRUE TRUE TRUE TRUE TRUE TRUE TRUE
##  [31] TRUE TRUE TRUE TRUE TRUE TRUE TRUE TRUE TRUE TRUE TRUE TRUE TRUE TRUE TRUE
##  [46] TRUE TRUE TRUE TRUE TRUE TRUE TRUE TRUE TRUE TRUE TRUE TRUE TRUE TRUE TRUE
##  [61] TRUE TRUE TRUE TRUE TRUE TRUE TRUE TRUE TRUE TRUE TRUE TRUE TRUE TRUE TRUE
##  [76] TRUE TRUE TRUE TRUE TRUE TRUE TRUE TRUE TRUE TRUE TRUE TRUE TRUE TRUE TRUE
##  [91] TRUE TRUE TRUE TRUE TRUE TRUE TRUE TRUE TRUE TRUE TRUE TRUE TRUE TRUE TRUE
## [106] TRUE TRUE TRUE TRUE TRUE TRUE TRUE TRUE TRUE TRUE TRUE TRUE TRUE TRUE TRUE
## [121] TRUE TRUE TRUE TRUE TRUE TRUE TRUE TRUE TRUE TRUE TRUE TRUE TRUE TRUE TRUE
## [136] TRUE TRUE TRUE TRUE TRUE TRUE TRUE TRUE TRUE TRUE TRUE TRUE TRUE TRUE TRUE
## [151] TRUE TRUE TRUE TRUE TRUE TRUE TRUE TRUE TRUE TRUE TRUE TRUE TRUE TRUE TRUE
## [166] TRUE TRUE TRUE TRUE TRUE TRUE TRUE TRUE TRUE TRUE TRUE TRUE TRUE TRUE TRUE
## [181] TRUE TRUE TRUE TRUE TRUE TRUE TRUE TRUE TRUE TRUE TRUE TRUE TRUE TRUE TRUE
## [196] TRUE TRUE
```

```
pdata$Phylum <- TaxaNames_main$Phylum
pdata$Family <- TaxaNames_main$Family
pdata$Order <- TaxaNames_main$Order

#1: taxa present pre-transplant (should correlate post-transplant)
pdata1 <- pdata[which(pdata$mean_pre!=0),]
pdata1b <- pdata[which(pdata$median_pre!=0),]

cor.test(pdata1b$mean_pre, pdata1b$mean_post, method='spearman')
```

```
## 
##  Spearman's rank correlation rho
## 
## data:  pdata1b$mean_pre and pdata1b$mean_post
## S = 22, p-value = 0.04583
## alternative hypothesis: true rho is not equal to 0
## sample estimates:
##       rho 
## 0.7380952
```

```
p1 <- ggplot(pdata1b, aes(x=mean_pre, y=mean_post)) + geom_point(size=3) + geom_smooth(method='lm')
p1 <- p1 + xlab("IgA Index (Pre-FMT)") + ylab("IgA Index (Post-FMT)")
p1 <- p1 + theme_bw(base_size=12)
p1 <- p1 + annotate("text", x = -0.05, y=0.1, label = "p=0.04", size=4)
p1 <- p1 + annotate("text", x = -0.05, y=0.08, label = "rho=0.74", size=4)
p1
```

```
# taxa present pre-transplant should NOT correlate with donor
cor.test(pdata1b$mean_donor, pdata1b$mean_post, method='spearman')
```

```
## 
##  Spearman's rank correlation rho
## 
## data:  pdata1b$mean_donor and pdata1b$mean_post
## S = 100, p-value = 0.6646
## alternative hypothesis: true rho is not equal to 0
## sample estimates:
##        rho 
## -0.1904762
```

```
p1 <- ggplot(pdata1b, aes(x=mean_donor, y=mean_post)) + geom_point(size=3) + geom_smooth(method='lm')
p1 <- p1 + xlab("IgA Index (Donor)") + ylab("IgA Index (Post-FMT)")
p1 <- p1 + theme_bw(base_size=12)
p1 <- p1 + annotate("text", x = -0.05, y=0.1, label = "p=0.66", size=4)
p1 <- p1 + annotate("text", x = -0.05, y=0.08, label = "rho=-0.19", size=4)
p1
```

```
#2: taxa present in donor 
pdata2b <- pdata[which(pdata$median_donor!=0),]

cor.test(pdata2b$mean_donor, pdata2b$mean_post, method='spearman')
```

```
## 
##  Spearman's rank correlation rho
## 
## data:  pdata2b$mean_donor and pdata2b$mean_post
## S = 9376, p-value = 0.003774
## alternative hypothesis: true rho is not equal to 0
## sample estimates:
##     rho 
## 0.42177
```

```
p <- ggplot(pdata2b, aes(x=mean_donor, y=mean_post)) + geom_point(size=3) + geom_smooth(method='lm')
p <- p + xlab("IgA Index (Donor)") + ylab("IgA Index (Post-FMT)")
p <- p + theme_bw(base_size=12)
p <- p + annotate("text", x = -0.25, y=0.2, label = "p=0.004", size=4)
p <- p + annotate("text", x = -0.26, y=0.16, label = "rho=0.42", size=4)
p
```

```
#correlation of these taxa with pre-transplant
cor.test(pdata2b$mean_pre, pdata2b$mean_post, method='spearman')
```

```
## 
##  Spearman's rank correlation rho
## 
## data:  pdata2b$mean_pre and pdata2b$mean_post
## S = 11377, p-value = 0.04403
## alternative hypothesis: true rho is not equal to 0
## sample estimates:
##       rho 
## 0.2983355
```

```
p <- ggplot(pdata2b, aes(x=mean_pre, y=mean_post)) + geom_point(size=3) + geom_smooth(method='lm')
p <- p + xlab("IgA Index (Pre-FMT)") + ylab("IgA Index (Post-FMT)")
p <- p + theme_bw(base_size=12)
p <- p + annotate("text", x = -0.05, y=0.2, label = "p=0.04", size=4)
p <- p + annotate("text", x = -0.05, y=0.16, label = "rho=0.30", size=4)
p
```

```
#3: taxa prevalent in donor but NOT pre-transplant
pdata3 <- pdata[which(pdata$median_donor!=0&pdata$median_pre==0),]
cor.test(pdata3$mean_donor, pdata3$mean_post, method='spearman')
```

```
## 
##  Spearman's rank correlation rho
## 
## data:  pdata3$mean_donor and pdata3$mean_post
## S = 4950, p-value = 0.001403
## alternative hypothesis: true rho is not equal to 0
## sample estimates:
##       rho 
## 0.4989879
```

```
p <- ggplot(pdata3, aes(x=mean_donor, y=mean_post)) + geom_point(size=3) + geom_smooth(method='lm')
p <- p + xlab("IgA Index (Donor)") + ylab("IgA Index (Post-FMT)")
p <- p + theme_bw(base_size=12)
p <- p + annotate("text", x = -0.25, y=0.2, label = "p=0.001", size=4)
p <- p + annotate("text", x = -0.26, y=0.16, label = "rho=0.50", size=4)
p
```

```
#average abundance versus IgA Index for the same taxa.
wilcox.data1b$Taxa==wilcox.data2b$Taxa
```

```
##   [1] TRUE TRUE TRUE TRUE TRUE TRUE TRUE TRUE TRUE TRUE TRUE TRUE TRUE TRUE TRUE
##  [16] TRUE TRUE TRUE TRUE TRUE TRUE TRUE TRUE TRUE TRUE TRUE TRUE TRUE TRUE TRUE
##  [31] TRUE TRUE TRUE TRUE TRUE TRUE TRUE TRUE TRUE TRUE TRUE TRUE TRUE TRUE TRUE
##  [46] TRUE TRUE TRUE TRUE TRUE TRUE TRUE TRUE TRUE TRUE TRUE TRUE TRUE TRUE TRUE
##  [61] TRUE TRUE TRUE TRUE TRUE TRUE TRUE TRUE TRUE TRUE TRUE TRUE TRUE TRUE TRUE
##  [76] TRUE TRUE TRUE TRUE TRUE TRUE TRUE TRUE TRUE TRUE TRUE TRUE TRUE TRUE TRUE
##  [91] TRUE TRUE TRUE TRUE TRUE TRUE TRUE TRUE TRUE TRUE TRUE TRUE TRUE TRUE TRUE
## [106] TRUE TRUE TRUE TRUE TRUE TRUE TRUE TRUE TRUE TRUE TRUE TRUE TRUE TRUE TRUE
## [121] TRUE TRUE TRUE TRUE TRUE TRUE TRUE TRUE TRUE TRUE TRUE TRUE TRUE TRUE TRUE
## [136] TRUE TRUE TRUE TRUE TRUE TRUE TRUE TRUE TRUE TRUE TRUE TRUE TRUE TRUE TRUE
## [151] TRUE TRUE TRUE TRUE TRUE TRUE TRUE TRUE TRUE TRUE TRUE TRUE TRUE TRUE TRUE
## [166] TRUE TRUE TRUE TRUE TRUE TRUE TRUE TRUE TRUE TRUE TRUE TRUE TRUE TRUE TRUE
## [181] TRUE TRUE TRUE TRUE TRUE TRUE TRUE TRUE TRUE TRUE TRUE TRUE TRUE TRUE TRUE
## [196] TRUE TRUE
```

```
wilcox.data2b$Taxa==wilcox.data3b$Taxa
```

```
##   [1] TRUE TRUE TRUE TRUE TRUE TRUE TRUE TRUE TRUE TRUE TRUE TRUE TRUE TRUE TRUE
##  [16] TRUE TRUE TRUE TRUE TRUE TRUE TRUE TRUE TRUE TRUE TRUE TRUE TRUE TRUE TRUE
##  [31] TRUE TRUE TRUE TRUE TRUE TRUE TRUE TRUE TRUE TRUE TRUE TRUE TRUE TRUE TRUE
##  [46] TRUE TRUE TRUE TRUE TRUE TRUE TRUE TRUE TRUE TRUE TRUE TRUE TRUE TRUE TRUE
##  [61] TRUE TRUE TRUE TRUE TRUE TRUE TRUE TRUE TRUE TRUE TRUE TRUE TRUE TRUE TRUE
##  [76] TRUE TRUE TRUE TRUE TRUE TRUE TRUE TRUE TRUE TRUE TRUE TRUE TRUE TRUE TRUE
##  [91] TRUE TRUE TRUE TRUE TRUE TRUE TRUE TRUE TRUE TRUE TRUE TRUE TRUE TRUE TRUE
## [106] TRUE TRUE TRUE TRUE TRUE TRUE TRUE TRUE TRUE TRUE TRUE TRUE TRUE TRUE TRUE
## [121] TRUE TRUE TRUE TRUE TRUE TRUE TRUE TRUE TRUE TRUE TRUE TRUE TRUE TRUE TRUE
## [136] TRUE TRUE TRUE TRUE TRUE TRUE TRUE TRUE TRUE TRUE TRUE TRUE TRUE TRUE TRUE
## [151] TRUE TRUE TRUE TRUE TRUE TRUE TRUE TRUE TRUE TRUE TRUE TRUE TRUE TRUE TRUE
## [166] TRUE TRUE TRUE TRUE TRUE TRUE TRUE TRUE TRUE TRUE TRUE TRUE TRUE TRUE TRUE
## [181] TRUE TRUE TRUE TRUE TRUE TRUE TRUE TRUE TRUE TRUE TRUE TRUE TRUE TRUE TRUE
## [196] TRUE TRUE
```

```
pdata_abund <- data.frame(mean_pre=wilcox.data1b$mean,
                    mean_post=wilcox.data2b$mean,
                    mean_donor=wilcox.data3b$mean,
                    median_pre=wilcox.data1b$median,
                    median_post=wilcox.data2b$median,
                    median_donor=wilcox.data3b$median,
                    Taxa=wilcox.data1b$Taxa)

TaxaNames_main$combined==pdata_abund$Taxa
```

```
##   [1] TRUE TRUE TRUE TRUE TRUE TRUE TRUE TRUE TRUE TRUE TRUE TRUE TRUE TRUE TRUE
##  [16] TRUE TRUE TRUE TRUE TRUE TRUE TRUE TRUE TRUE TRUE TRUE TRUE TRUE TRUE TRUE
##  [31] TRUE TRUE TRUE TRUE TRUE TRUE TRUE TRUE TRUE TRUE TRUE TRUE TRUE TRUE TRUE
##  [46] TRUE TRUE TRUE TRUE TRUE TRUE TRUE TRUE TRUE TRUE TRUE TRUE TRUE TRUE TRUE
##  [61] TRUE TRUE TRUE TRUE TRUE TRUE TRUE TRUE TRUE TRUE TRUE TRUE TRUE TRUE TRUE
##  [76] TRUE TRUE TRUE TRUE TRUE TRUE TRUE TRUE TRUE TRUE TRUE TRUE TRUE TRUE TRUE
##  [91] TRUE TRUE TRUE TRUE TRUE TRUE TRUE TRUE TRUE TRUE TRUE TRUE TRUE TRUE TRUE
## [106] TRUE TRUE TRUE TRUE TRUE TRUE TRUE TRUE TRUE TRUE TRUE TRUE TRUE TRUE TRUE
## [121] TRUE TRUE TRUE TRUE TRUE TRUE TRUE TRUE TRUE TRUE TRUE TRUE TRUE TRUE TRUE
## [136] TRUE TRUE TRUE TRUE TRUE TRUE TRUE TRUE TRUE TRUE TRUE TRUE TRUE TRUE TRUE
## [151] TRUE TRUE TRUE TRUE TRUE TRUE TRUE TRUE TRUE TRUE TRUE TRUE TRUE TRUE TRUE
## [166] TRUE TRUE TRUE TRUE TRUE TRUE TRUE TRUE TRUE TRUE TRUE TRUE TRUE TRUE TRUE
## [181] TRUE TRUE TRUE TRUE TRUE TRUE TRUE TRUE TRUE TRUE TRUE TRUE TRUE TRUE TRUE
## [196] TRUE TRUE
```

```
pdata_abund$Phylum <- TaxaNames_main$Phylum
pdata_abund$Family <- TaxaNames_main$Family
pdata_abund$Order <- TaxaNames_main$Order

pdata$Taxa==pdata_abund$Taxa
```

```
##   [1] TRUE TRUE TRUE TRUE TRUE TRUE TRUE TRUE TRUE TRUE TRUE TRUE TRUE TRUE TRUE
##  [16] TRUE TRUE TRUE TRUE TRUE TRUE TRUE TRUE TRUE TRUE TRUE TRUE TRUE TRUE TRUE
##  [31] TRUE TRUE TRUE TRUE TRUE TRUE TRUE TRUE TRUE TRUE TRUE TRUE TRUE TRUE TRUE
##  [46] TRUE TRUE TRUE TRUE TRUE TRUE TRUE TRUE TRUE TRUE TRUE TRUE TRUE TRUE TRUE
##  [61] TRUE TRUE TRUE TRUE TRUE TRUE TRUE TRUE TRUE TRUE TRUE TRUE TRUE TRUE TRUE
##  [76] TRUE TRUE TRUE TRUE TRUE TRUE TRUE TRUE TRUE TRUE TRUE TRUE TRUE TRUE TRUE
##  [91] TRUE TRUE TRUE TRUE TRUE TRUE TRUE TRUE TRUE TRUE TRUE TRUE TRUE TRUE TRUE
## [106] TRUE TRUE TRUE TRUE TRUE TRUE TRUE TRUE TRUE TRUE TRUE TRUE TRUE TRUE TRUE
## [121] TRUE TRUE TRUE TRUE TRUE TRUE TRUE TRUE TRUE TRUE TRUE TRUE TRUE TRUE TRUE
## [136] TRUE TRUE TRUE TRUE TRUE TRUE TRUE TRUE TRUE TRUE TRUE TRUE TRUE TRUE TRUE
## [151] TRUE TRUE TRUE TRUE TRUE TRUE TRUE TRUE TRUE TRUE TRUE TRUE TRUE TRUE TRUE
## [166] TRUE TRUE TRUE TRUE TRUE TRUE TRUE TRUE TRUE TRUE TRUE TRUE TRUE TRUE TRUE
## [181] TRUE TRUE TRUE TRUE TRUE TRUE TRUE TRUE TRUE TRUE TRUE TRUE TRUE TRUE TRUE
## [196] TRUE TRUE
```

```
names(pdata) <- c("IgAIndex_mean_pre", "IgAIndex_mean_post", "IgAIndex_mean_donor", "IgAIndex_median_pre", "IgAIndex_median_post",
                  "IgAIndex_median_donor", "Taxa", "Phylum", "Family", "Order")
names(pdata_abund) <- c("Abund_mean_pre", "Abund_mean_post", "Abund_mean_donor", "Abund_median_pre", "Abund_median_post",
                  "Abund_median_donor", "Taxa", "Phylum", "Family", "Order")

pdata_index_abund <- full_join(pdata, pdata_abund)

#abund versus index for pre-FMT

cor.test(pdata_index_abund$IgAIndex_mean_pre, pdata_index_abund$Abund_mean_pre, method='spearman') #p=0.6
```

```
## 
##  Spearman's rank correlation rho
## 
## data:  pdata_index_abund$IgAIndex_mean_pre and pdata_index_abund$Abund_mean_pre
## S = 1225691, p-value = 0.5954
## alternative hypothesis: true rho is not equal to 0
## sample estimates:
##        rho 
## 0.03806689
```

```
p <- ggplot(pdata_index_abund, aes(x=IgAIndex_mean_pre, y=Abund_mean_pre)) + geom_point(size=3) + geom_smooth(method='lm')
p <- p + theme_bw(base_size=12)
#p <- p + scale_colour_manual(values=c("purple", "forestgreen", "goldenrod", "deepskyblue", "red", "turquoise"))
p <- p + annotate("text", x = 0.1, y=1e-05, label = "p=0.59", size=4)
p <- p + annotate("text", x = 0.1, y=2e-06, label = "rho=0.04", size=4)
p <- p + xlab("IgA Index (Pre-FMT)") + ylab("Abundance (Pre-FMT)")
p <- p +scale_y_log10()
p
```

```
#abund versus index for post-FMT
cor.test(pdata_index_abund$IgAIndex_mean_post, pdata_index_abund$Abund_mean_post, method='spearman') #p=0.7
```

```
## 
##  Spearman's rank correlation rho
## 
## data:  pdata_index_abund$IgAIndex_mean_post and pdata_index_abund$Abund_mean_post
## S = 1310264, p-value = 0.6929
## alternative hypothesis: true rho is not equal to 0
## sample estimates:
##         rho 
## -0.02830674
```

```
p <- ggplot(pdata_index_abund, aes(x=IgAIndex_mean_post, y=Abund_mean_post)) + geom_point(size=3) + geom_smooth(method='lm')
p <- p + theme_bw(base_size=12)
#p <- p + scale_colour_manual(values=c("purple", "forestgreen", "goldenrod", "deepskyblue", "red", "turquoise"))
p <- p + annotate("text", x = 0.2, y=1e-05, label = "p=0.69", size=4)
p <- p + annotate("text", x = 0.2, y=2e-06, label = "rho=-0.28", size=4)
p <- p + xlab("IgA Index (Post-FMT)") + ylab("Abundance (Post-FMT)")
p <- p +scale_y_log10()
p
```

# Differences by sort batch or seq depth?

```
names(IgAPost_trim75)==gsub("-", ".", metaIgAPost$SampleID)
```

```
##  [1] TRUE TRUE TRUE TRUE TRUE TRUE TRUE TRUE TRUE TRUE TRUE TRUE TRUE TRUE TRUE
## [16] TRUE TRUE TRUE TRUE TRUE TRUE TRUE TRUE TRUE TRUE TRUE TRUE TRUE TRUE TRUE
## [31] TRUE TRUE TRUE TRUE TRUE TRUE
```

```
#multiple kruskal-wallis by sort day
MW.p = apply(IgAPost_trim75,1,
             function(x) kruskal.test(c(x)~metaIgAPost$Sort_Day)$p.value)
p.res = data.frame(taxa=row.names(IgAPost_trim75),MW.p)
# Perform multiple comparison correction using a given method of choice
p.res$rel.fdr <- p.adjust(p.res$MW.p, method="fdr") 

#hits
batch_hits <- p.res[which(p.res$rel.fdr<0.1),]
batch_hits
```

```
## [1] taxa    MW.p    rel.fdr
## <0 rows> (or 0-length row.names)
```

```
#multiple spearman by sequencing depth
MW.p = apply(IgAPost_trim75,1,
             function(x) kruskal.test(c(x)~metaIgAPost$depth)$p.value)
p.res = data.frame(taxa=row.names(IgAPost_trim75),MW.p)
# Perform multiple comparison correction using a given method of choice
p.res$rel.fdr <- p.adjust(p.res$MW.p, method="fdr") 

#hits
depth_hits <- p.res[which(p.res$rel.fdr<0.1),]
depth_hits
```

```
## [1] taxa    MW.p    rel.fdr
## <0 rows> (or 0-length row.names)
```

# Differences in IgA-targeting between pre & post transplant (paired analysis)

Test

```
row.names(IgA_settrim75_t)==gsub("-", ".", metaIgA$SampleID)
```

```
##  [1] TRUE TRUE TRUE TRUE TRUE TRUE TRUE TRUE TRUE TRUE TRUE TRUE TRUE TRUE TRUE
## [16] TRUE TRUE TRUE TRUE TRUE TRUE TRUE TRUE TRUE TRUE TRUE TRUE TRUE TRUE TRUE
## [31] TRUE TRUE TRUE TRUE TRUE TRUE TRUE TRUE TRUE TRUE TRUE TRUE TRUE TRUE TRUE
## [46] TRUE TRUE TRUE TRUE TRUE TRUE TRUE TRUE TRUE TRUE TRUE TRUE TRUE TRUE TRUE
## [61] TRUE TRUE TRUE TRUE TRUE TRUE TRUE TRUE TRUE TRUE TRUE TRUE TRUE TRUE TRUE
## [76] TRUE TRUE
```

```
data_adonis <- data.frame(metaIgA, IgA_settrim75_t)

data_paired <- dplyr::filter(data_adonis, Patient %in% metaIgAPre$Patient & Patient %in% metaIgAPost$Patient)
data_paired <-  data_paired[order(data_paired$Patient),]
data_paired <-  data_paired[order(data_paired$SampleType),]
names(data_paired)
```

```
##   [1] "SampleID"                                                                                                                                       
##   [2] "Sort"                                                                                                                                           
##   [3] "Patient"                                                                                                                                        
##   [4] "Stool_no"                                                                                                                                       
##   [5] "SampleType"                                                                                                                                     
##   [6] "Patient_Number"                                                                                                                                 
##   [7] "Gender"                                                                                                                                         
##   [8] "DOB"                                                                                                                                            
##   [9] "Tx_Date"                                                                                                                                        
##  [10] "Donor"                                                                                                                                          
##  [11] "IBD"                                                                                                                                            
##  [12] "Delivery"                                                                                                                                       
##  [13] "Stool.no..t0"                                                                                                                                   
##  [14] "Stool.no..t4"                                                                                                                                   
##  [15] "Age_at_FMT"                                                                                                                                     
##  [16] "Height_.cm."                                                                                                                                    
##  [17] "Height_.m."                                                                                                                                     
##  [18] "FMT_Date"                                                                                                                                       
##  [19] "Cap_vs._Col"                                                                                                                                    
##  [20] "Wt_Post.CDI"                                                                                                                                    
##  [21] "Wt_Loss"                                                                                                                                        
##  [22] "Wt_Pre.CDI"                                                                                                                                     
##  [23] "X._Wt_Loss"                                                                                                                                     
##  [24] "BMI_Pre.CDI"                                                                                                                                    
##  [25] "Wt_Post.FMT_1"                                                                                                                                  
##  [26] "BMI_1"                                                                                                                                          
##  [27] "Wt_Post.FMT_4"                                                                                                                                  
##  [28] "BMI_4"                                                                                                                                          
##  [29] "Wt_Post.FMT_12"                                                                                                                                 
##  [30] "BMI_12"                                                                                                                                         
##  [31] "Wt_Post.FMT_24"                                                                                                                                 
##  [32] "BMI_24"                                                                                                                                         
##  [33] "Wt_Post.FMT_48"                                                                                                                                 
##  [34] "BMI_48"                                                                                                                                         
##  [35] "Data_collection"                                                                                                                                
##  [36] "Percent_IgA"                                                                                                                                    
##  [37] "Sort_Day"                                                                                                                                       
##  [38] "Column1"                                                                                                                                        
##  [39] "Column2"                                                                                                                                        
##  [40] "Cd_.ng.ml."                                                                                                                                     
##  [41] "Co_.ng.ml."                                                                                                                                     
##  [42] "Cu_.ng.ml."                                                                                                                                     
##  [43] "Fe_.ug.ml."                                                                                                                                     
##  [44] "Mg_.ug.ml."                                                                                                                                     
##  [45] "Mn_.ng.ml."                                                                                                                                     
##  [46] "Ni_.ng.ml."                                                                                                                                     
##  [47] "Pb_.ng.ml."                                                                                                                                     
##  [48] "Se_.ng.ml."                                                                                                                                     
##  [49] "Zn_.ug.ml."                                                                                                                                     
##  [50] "Visit"                                                                                                                                          
##  [51] "Randomization_Group"                                                                                                                            
##  [52] "Age_at_Tx"                                                                                                                                      
##  [53] "Sex"                                                                                                                                            
##  [54] "Cd2"                                                                                                                                            
##  [55] "Pb2"                                                                                                                                            
##  [56] "Original.ID"                                                                                                                                    
##  [57] "Participant"                                                                                                                                    
##  [58] "Timepoint"                                                                                                                                      
##  [59] "Serum_2.hydroxybutyrate"                                                                                                                        
##  [60] "Serum_2.methylbutyrate"                                                                                                                         
##  [61] "Serum_Acetate"                                                                                                                                  
##  [62] "Serum_Butyrate"                                                                                                                                 
##  [63] "Serum_Caproate"                                                                                                                                 
##  [64] "Serum_Isobutyrate"                                                                                                                              
##  [65] "Serum_Isovalerate"                                                                                                                              
##  [66] "Serum_Lactate"                                                                                                                                  
##  [67] "Serum_Propionate"                                                                                                                               
##  [68] "Serum_Total..without.valerate."                                                                                                                 
##  [69] "Serum_Valerate"                                                                                                                                 
##  [70] "Fecal_Acetate"                                                                                                                                  
##  [71] "Fecal_Butyrate"                                                                                                                                 
##  [72] "Fecal_Isobutyrate"                                                                                                                              
##  [73] "Fecal_Isovalerate"                                                                                                                              
##  [74] "Fecal_Propionate"                                                                                                                               
##  [75] "Fecal_Valerate"                                                                                                                                 
##  [76] "Fecal_Total.SCFAs"                                                                                                                              
##  [77] "Serum_Ursodeoxycholic_acid"                                                                                                                     
##  [78] "Serum_Ursocholanic_acid"                                                                                                                        
##  [79] "Serum_Tauroursodeoxycholic_acid"                                                                                                                
##  [80] "Serum_Taurolithocholic_acid"                                                                                                                    
##  [81] "Serum_Taurohyodeoxycholic_acid"                                                                                                                 
##  [82] "Serum_Taurohyocholic_acid"                                                                                                                      
##  [83] "Serum_Taurodeoxycholic_acid"                                                                                                                    
##  [84] "Serum_Taurocholic_acid"                                                                                                                         
##  [85] "Serum_Taurochenodeoxycholic_acid"                                                                                                               
##  [86] "Serum_Lithocholic_acid"                                                                                                                         
##  [87] "Serum_Lithocholenic_acid"                                                                                                                       
##  [88] "Serum_Isodeoxycholic_acid"                                                                                                                      
##  [89] "Serum_Hyodeoxycholic_acid"                                                                                                                      
##  [90] "Serum_Hyocholic_acid"                                                                                                                           
##  [91] "Serum_Glycoursodeoxycholic_acid_2"                                                                                                              
##  [92] "Serum_Glycoursodeoxycholic_acid_1"                                                                                                              
##  [93] "Serum_Glycoursodeoxycholic_acid"                                                                                                                
##  [94] "Serum_Glycolithocholic_acid"                                                                                                                    
##  [95] "Serum_Glycohyodeoxycholic_acid"                                                                                                                 
##  [96] "Serum_Glycohyocholic_acid"                                                                                                                      
##  [97] "Serum_Glycodeoxycholic_acid"                                                                                                                    
##  [98] "Serum_Glycocholic_acid"                                                                                                                         
##  [99] "Serum_Glycochenodeoxycholic_acid"                                                                                                               
## [100] "Serum_Deoxycholic_acid"                                                                                                                         
## [101] "Serum_Cholic_acid"                                                                                                                              
## [102] "Serum_Chenodeoxycholic_acid"                                                                                                                    
## [103] "Feces_Ursodeoxycholic_acid"                                                                                                                     
## [104] "Feces_Ursocholanic_acid"                                                                                                                        
## [105] "Feces_Tauroursodeoxycholic_acid"                                                                                                                
## [106] "Feces_Tauroursocholanic_acid"                                                                                                                   
## [107] "Feces_Taurolithocholic_acid"                                                                                                                    
## [108] "Feces_Taurohyodeoxycholic_acid"                                                                                                                 
## [109] "Feces_Taurohyocholic_acid"                                                                                                                      
## [110] "Feces_Taurodeoxycholic_acid"                                                                                                                    
## [111] "Feces_Taurocholic_acid"                                                                                                                         
## [112] "Feces_Taurochenodeoxycholic_acid"                                                                                                               
## [113] "Feces_Lithocholic_acid"                                                                                                                         
## [114] "Feces_Hyocholic_acid"                                                                                                                           
## [115] "Feces_Glycoursodeoxycholic_acid"                                                                                                                
## [116] "Feces_Glycolithocholic_acid"                                                                                                                    
## [117] "Feces_Glycohyodeoxycholic_acid"                                                                                                                 
## [118] "Feces_Glycohyocholic_acid"                                                                                                                      
## [119] "Feces_Glycodeoxycholic_acid"                                                                                                                    
## [120] "Feces_Glycocholic_acid"                                                                                                                         
## [121] "Feces_Glycochenodeoxycholic_acid"                                                                                                               
## [122] "Feces_Deoxycholic_acid"                                                                                                                         
## [123] "Feces_Cholic_acid"                                                                                                                              
## [124] "Feces_Chenodeoxycholic_Acid"                                                                                                                    
## [125] "Serum_secondary"                                                                                                                                
## [126] "Serum_primary"                                                                                                                                  
## [127] "Feces_secondary"                                                                                                                                
## [128] "Feces_primary"                                                                                                                                  
## [129] "Sample"                                                                                                                                         
## [130] "LB"                                                                                                                                             
## [131] "HB"                                                                                                                                             
## [132] "S0"                                                                                                                                             
## [133] "S1"                                                                                                                                             
## [134] "S2"                                                                                                                                             
## [135] "S3"                                                                                                                                             
## [136] "S4"                                                                                                                                             
## [137] "G0"                                                                                                                                             
## [138] "G1"                                                                                                                                             
## [139] "G2"                                                                                                                                             
## [140] "G3"                                                                                                                                             
## [141] "G4"                                                                                                                                             
## [142] "OM"                                                                                                                                             
## [143] "B"                                                                                                                                              
## [144] "CF"                                                                                                                                             
## [145] "AF"                                                                                                                                             
## [146] "Donor.ID"                                                                                                                                       
## [147] "Donor_StoolID"                                                                                                                                  
## [148] "fecal_twin"                                                                                                                                     
## [149] "fecal_twin_ID"                                                                                                                                  
## [150] "sample_sums"                                                                                                                                    
## [151] "Donor.ID3"                                                                                                                                      
## [152] "Donor.ID2"                                                                                                                                      
## [153] "SampleType2"                                                                                                                                    
## [154] "depth"                                                                                                                                          
## [155] "X.Bacteria.Bacteroidetes.Bacteroidia.Bacteroidales..Barnesiellaceae..NA.NA.5b0c52a1507deba61b41b7f9d8187386"                                    
## [156] "X.Bacteria.Bacteroidetes.Bacteroidia.Bacteroidales..Barnesiellaceae..NA.NA.9d0579ce6a317ca0e626a5c68981c725"                                    
## [157] "X.Bacteria.Bacteroidetes.Bacteroidia.Bacteroidales..Barnesiellaceae..NA.NA.e6cd2fee507eb45ff5f3c0a4191f3876"                                    
## [158] "X.Bacteria.Bacteroidetes.Bacteroidia.Bacteroidales..Barnesiellaceae..NA.NA.158782cefa6e28406a2a0aeb75220f0a"                                    
## [159] "X.Bacteria.Bacteroidetes.Bacteroidia.Bacteroidales.Porphyromonadaceae.Parabacteroides.NA.0d8b9177d01f328c2f5d5efa4c2acbc5"                      
## [160] "X.Bacteria.Bacteroidetes.Bacteroidia.Bacteroidales.Porphyromonadaceae.Parabacteroides.NA.e17d964cd0fd8c2ddbd1dacfa8b536e3"                      
## [161] "X.Bacteria.Bacteroidetes.Bacteroidia.Bacteroidales.Porphyromonadaceae.Parabacteroides.distasonis.2cdb259b754c2db622ed9fb5a6517a37"              
## [162] "X.Bacteria.Bacteroidetes.Bacteroidia.Bacteroidales.Porphyromonadaceae.Parabacteroides.distasonis.f4f297232da0f8d7dae7f9c432501e22"              
## [163] "X.Bacteria.Bacteroidetes.Bacteroidia.Bacteroidales.Porphyromonadaceae.Parabacteroides.distasonis.4844896c9c40eaca2fa0291163b9ca52"              
## [164] "X.Bacteria.Bacteroidetes.Bacteroidia.Bacteroidales.Bacteroidaceae.Bacteroides.ovatus.2c2018d4ecccfa3cb27b99a04c9222b1"                          
## [165] "X.Bacteria.Bacteroidetes.Bacteroidia.Bacteroidales.Bacteroidaceae.Bacteroides.ovatus.03af966ff07ddef2b87da992b85b600b"                          
## [166] "X.Bacteria.Bacteroidetes.Bacteroidia.Bacteroidales.Bacteroidaceae.Bacteroides.NA.51e441cbdcc80da0656e82293ae160b5"                              
## [167] "X.Bacteria.Bacteroidetes.Bacteroidia.Bacteroidales.Bacteroidaceae.Bacteroides.fragilis.9496d87b94d90dff068f0716603930bd"                        
## [168] "X.Bacteria.Bacteroidetes.Bacteroidia.Bacteroidales.Bacteroidaceae.Bacteroides.fragilis.35ffdd51464e2c68179717e5334a1d7e"                        
## [169] "X.Bacteria.Bacteroidetes.Bacteroidia.Bacteroidales.Bacteroidaceae.Bacteroides.caccae.ec4075339e16f5cd45fd5a7955596899"                          
## [170] "X.Bacteria.Bacteroidetes.Bacteroidia.Bacteroidales.Bacteroidaceae.Bacteroides.NA.25d727166a36df8d2f6a915a945bf5ac"                              
## [171] "X.Bacteria.Bacteroidetes.Bacteroidia.Bacteroidales.Bacteroidaceae.Bacteroides.NA.3b872d4cecb7a9437ce9e5add011b471"                              
## [172] "X.Bacteria.Bacteroidetes.Bacteroidia.Bacteroidales.Bacteroidaceae.Bacteroides.eggerthii.8ad4a56a5f526df40d97b371ba0ef33e"                       
## [173] "X.Bacteria.Bacteroidetes.Bacteroidia.Bacteroidales.Bacteroidaceae.Bacteroides.eggerthii.30dd9fd45122f82c12b166a042d1eaf9"                       
## [174] "X.Bacteria.Bacteroidetes.Bacteroidia.Bacteroidales.Bacteroidaceae.Bacteroides.uniformis.63b26504f32377cd78d6068bffb86b9a"                       
## [175] "X.Bacteria.Bacteroidetes.Bacteroidia.Bacteroidales.Bacteroidaceae.Bacteroides.uniformis.574ab9c17692ffd001643c930f6895f5"                       
## [176] "X.Bacteria.Bacteroidetes.Bacteroidia.Bacteroidales.Prevotellaceae.Prevotella.melaninogenica.d0b698c7298bf04110a6d2f220879bfb"                   
## [177] "X.Bacteria.Bacteroidetes.Bacteroidia.Bacteroidales..Paraprevotellaceae..NA.NA.85fddfaca6687f22a2b1c5ed45a6041c"                                 
## [178] "X.Bacteria.Bacteroidetes.Bacteroidia.Bacteroidales..Paraprevotellaceae..Paraprevotella.NA.37dac50f454a3cf754e83790eee77108"                     
## [179] "X.Bacteria.Bacteroidetes.Bacteroidia.Bacteroidales.Bacteroidaceae.Bacteroides.NA.b65eb19257f7a2bedb5a1c4b42aeb396"                              
## [180] "X.Bacteria.Bacteroidetes.Bacteroidia.Bacteroidales.Bacteroidaceae.Bacteroides.NA.a7a1a93ecfcef4cb45b42307a4fa3bca"                              
## [181] "X.Bacteria.Bacteroidetes.Bacteroidia.Bacteroidales.Bacteroidaceae.Bacteroides.NA.668fdb718997fc1589c7817655d4bb5f"                              
## [182] "X.Bacteria.Bacteroidetes.Bacteroidia.Bacteroidales..Odoribacteraceae..Butyricimonas.NA.0bb0e1802451aace7137a4d598f775f5"                        
## [183] "X.Bacteria.Bacteroidetes.Bacteroidia.Bacteroidales..Odoribacteraceae..Odoribacter.NA.3ea67969a01ec419ec1d4784d379ad1d"                          
## [184] "X.Bacteria.Bacteroidetes.Sphingobacteriia.Sphingobacteriales.Sphingobacteriaceae.Pedobacter.NA.e5e45fe5960b838c45765068621efcd5"                
## [185] "X.Bacteria.Bacteroidetes.Bacteroidia.Bacteroidales.Rikenellaceae.NA.NA.76d4dadc006ff1d530d38dd3cfac300c"                                        
## [186] "X.Bacteria.Bacteroidetes.Bacteroidia.Bacteroidales.Rikenellaceae.NA.NA.e47a63686b619f67783f9b9aa52b86bf"                                        
## [187] "X.Bacteria.Bacteroidetes.Bacteroidia.Bacteroidales.Rikenellaceae.NA.NA.619f64f2bf103286f4f70bfd89500ed4"                                        
## [188] "X.Bacteria.Bacteroidetes.Bacteroidia.Bacteroidales.Rikenellaceae.NA.NA.7534fb513a4b404419edc4e91920af3f"                                        
## [189] "X.Bacteria.Bacteroidetes.Bacteroidia.Bacteroidales.Rikenellaceae.NA.NA.a231814083060142816974207844eff2"                                        
## [190] "X.Bacteria.Bacteroidetes..Saprospirae...Saprospirales..Chitinophagaceae.Sediminibacterium.NA.372e9cfd6acc38cd00e88aac575f8afa"                  
## [191] "X.Bacteria.Bacteroidetes..Saprospirae...Saprospirales..Chitinophagaceae.Sediminibacterium.NA.a47ed77e3a3ab04be7139730ee5e5a98"                  
## [192] "X.Bacteria.Bacteroidetes..Saprospirae...Saprospirales..Chitinophagaceae.Sediminibacterium.NA.1ee27fee30b2e6832a2be5e28f0978f3"                  
## [193] "X.Bacteria.Chlamydiae.Chlamydiia.Chlamydiales.Parachlamydiaceae.Parachlamydia.NA.8a592a80ca5be07c6b8d28ae3f835dcd"                              
## [194] "X.Bacteria.Proteobacteria.Betaproteobacteria.Burkholderiales.Oxalobacteraceae.Ralstonia.NA.dde6d3e36af306c913854a9cf0ddf248"                    
## [195] "X.Bacteria.Proteobacteria.Betaproteobacteria.Burkholderiales.Alcaligenaceae.Sutterella.NA.0088553fbbbf2fbc8918ad224557d65c"                     
## [196] "X.Bacteria.Proteobacteria.Betaproteobacteria.Burkholderiales.Alcaligenaceae.Sutterella.NA.7d17d197d7978be0b6684209ce257e20"                     
## [197] "X.Bacteria.Proteobacteria.Gammaproteobacteria.Enterobacteriales.Enterobacteriaceae.NA.NA.677929eac52be081cdae08a0b9c70eb2"                      
## [198] "X.Bacteria.Proteobacteria.Gammaproteobacteria.Enterobacteriales.Enterobacteriaceae.NA.NA.bfe54af4c9180d37a0d76f6dafe79a5a"                      
## [199] "X.Bacteria.Proteobacteria.Gammaproteobacteria.Enterobacteriales.Enterobacteriaceae.Escherichia.coli.1b158b8b2922d4fcad5d9cea607cbb7d"           
## [200] "X.Bacteria.Proteobacteria.Gammaproteobacteria.Enterobacteriales.Enterobacteriaceae.Morganella.morganii.78056058faedd75d706633c5f55a975f"        
## [201] "X.Bacteria.Proteobacteria.Gammaproteobacteria.Xanthomonadales.Sinobacteraceae.NA.NA.09bedf1ee9478654b581fea611f919e7"                           
## [202] "X.Bacteria.Proteobacteria.Gammaproteobacteria.Pseudomonadales.Pseudomonadaceae.NA.NA.1f878f615fcfc8d7bd381a7841ac1e41"                          
## [203] "X.Bacteria.Proteobacteria.Gammaproteobacteria.Pseudomonadales.Pseudomonadaceae.Pseudomonas.veronii.402e5913597695a16d7cad415ffff02f"            
## [204] "X.Bacteria.Proteobacteria.Gammaproteobacteria.Xanthomonadales.Xanthomonadaceae.Stenotrophomonas.acidaminiphila.3588479e8e7443fdc3657f4b81cebf4c"
## [205] "X.Bacteria.Proteobacteria.Gammaproteobacteria.Xanthomonadales.Xanthomonadaceae.Stenotrophomonas.NA.891019e319f5d8d088ed4f9379fd63ef"            
## [206] "X.Bacteria.Proteobacteria.Deltaproteobacteria.Desulfovibrionales.Desulfovibrionaceae.Bilophila.NA.ece50a62168f85fc61385d8adb4c6494"             
## [207] "X.Bacteria.Proteobacteria.Deltaproteobacteria.Desulfovibrionales.Desulfovibrionaceae.Desulfovibrio.NA.dfd770d48651635c39bdafe13f62e75a"         
## [208] "X.Bacteria.Proteobacteria.Deltaproteobacteria.Myxococcales.0319.6G20.NA.NA.9f1414e2233d20717c5ed418bd88e872"                                    
## [209] "X.Bacteria.Actinobacteria.Coriobacteriia.Coriobacteriales.Coriobacteriaceae.Collinsella.aerofaciens.dfba68ef0fd0e712608eb2a0078013a7"           
## [210] "X.Bacteria.Actinobacteria.Coriobacteriia.Coriobacteriales.Coriobacteriaceae.Slackia.NA.c9a59a2ded38b324d6d58202a4137f9b"                        
## [211] "X.Bacteria.Actinobacteria.Coriobacteriia.Coriobacteriales.Coriobacteriaceae.Eggerthella.lenta.6bb6aad559c5eaeb22b2eef7874ef038"                 
## [212] "X.Bacteria.Actinobacteria.Coriobacteriia.Coriobacteriales.Coriobacteriaceae.NA.NA.23368c2d745ed62db9ac0a19c9dcec5d"                             
## [213] "X.Bacteria.Actinobacteria.Coriobacteriia.Coriobacteriales.Coriobacteriaceae.Atopobium.NA.5f92443932d0deb4cf0d196b6e4fe4c0"                      
## [214] "X.Bacteria.Actinobacteria.Actinobacteria.Actinomycetales.Brevibacteriaceae.Brevibacterium.aureum.2861b60feb98b96bfdfae4152ea84ae7"              
## [215] "X.Bacteria.Actinobacteria.Actinobacteria.Actinomycetales.NA.NA.NA.bb6c24bb90dd84f577f7488df6312451"                                             
## [216] "X.Bacteria.Actinobacteria.Actinobacteria.Bifidobacteriales.Bifidobacteriaceae.Bifidobacterium.NA.7b223719a0af567e7ea99f06f7ea1068"              
## [217] "X.Bacteria.Actinobacteria.Actinobacteria.Bifidobacteriales.Bifidobacteriaceae.Bifidobacterium.NA.69e611251f4d8582e312afa5737f033e"              
## [218] "X.Bacteria.Actinobacteria.Actinobacteria.Bifidobacteriales.Bifidobacteriaceae.Bifidobacterium.adolescentis.554c761996ebab999befda1b695fd81d"    
## [219] "X.Bacteria.Actinobacteria.Actinobacteria.Actinomycetales.Actinomycetaceae.Actinomyces.NA.608e6548b1b4cbb6176c8fce090991a5"                      
## [220] "X.Bacteria.Actinobacteria.Actinobacteria.Actinomycetales.Actinomycetaceae.Actinomyces.NA.a78fa572a60226d8150bfb0fc0fad652"                      
## [221] "X.Bacteria.Actinobacteria.Actinobacteria.Actinomycetales.Pseudonocardiaceae.Pseudonocardia.NA.78c07a3480fde312c999698b581e8c5f"                 
## [222] "X.Bacteria.Verrucomicrobia.Verrucomicrobiae.Verrucomicrobiales.Verrucomicrobiaceae.Akkermansia.muciniphila.8f98fb8693ed59c21399d83ce2d10724"    
## [223] "X.Bacteria.Firmicutes.Bacilli.Lactobacillales.Lactobacillaceae.Lactobacillus.zeae.5a4eda6fcdd6ab834dba0111fbe55d95"                             
## [224] "X.Bacteria.Firmicutes.Bacilli.Lactobacillales.Lactobacillaceae.Lactobacillus.zeae.45a68a9eee3cf83e27f4ea309d57ffc3"                             
## [225] "X.Bacteria.Firmicutes.Bacilli.Lactobacillales.Lactobacillaceae.Pediococcus.NA.3c133555aa2bbda4902d66bdceb138a3"                                 
## [226] "X.Bacteria.Firmicutes.Bacilli.Lactobacillales.Lactobacillaceae.Lactobacillus.salivarius.ff2bd29ff42e4dc25a31714e0b6c2dca"                       
## [227] "X.Bacteria.Firmicutes.Bacilli.Lactobacillales.Streptococcaceae.Lactococcus.NA.b39c338f5e964b6cb87e07f10badc6c4"                                 
## [228] "X.Bacteria.Firmicutes.Bacilli.Lactobacillales.Streptococcaceae.Streptococcus.NA.bd2ebc70501f7d867c204f94c4e483da"                               
## [229] "X.Bacteria.Firmicutes.Bacilli.Lactobacillales.Streptococcaceae.Streptococcus.NA.a5189f77a2cfeab3bc1602ff5c8ac3e9"                               
## [230] "X.Bacteria.Firmicutes.Bacilli.Lactobacillales.Streptococcaceae.Streptococcus.NA.5d6ee23084c6b9c96deb9a83295abc8a"                               
## [231] "X.Bacteria.Firmicutes.Bacilli.Lactobacillales.Streptococcaceae.Streptococcus.NA.73bf8d1a5983e34a0cb84e3cae127815"                               
## [232] "X.Bacteria.Firmicutes.Bacilli.Lactobacillales.Carnobacteriaceae.Granulicatella.NA.24a60c6448e70d9198ad6ba93520958c"                             
## [233] "X.Bacteria.Firmicutes.Bacilli.Lactobacillales.Aerococcaceae.Abiotrophia.NA.8114b1d0274e9e4bb6c91f6af1b8fac8"                                    
## [234] "X.Bacteria.Firmicutes.Bacilli.Gemellales.Gemellaceae.NA.NA.cda4e6f933bb3108ea3e92f9db411c00"                                                    
## [235] "X.Bacteria.Firmicutes.Clostridia.Clostridiales.Veillonellaceae.Phascolarctobacterium.NA.e5413f67faa6b8c0c3e63e48836c0b42"                       
## [236] "X.Bacteria.Firmicutes.Clostridia.Clostridiales.Veillonellaceae.Megasphaera.NA.2f93e58b78f2842e83abd5fde37ad276"                                 
## [237] "X.Bacteria.Firmicutes.Clostridia.Clostridiales.Veillonellaceae.Dialister.NA.520c77820886daeb8cf0d6497cfb1344"                                   
## [238] "X.Bacteria.Firmicutes.Clostridia.Clostridiales.Veillonellaceae.Veillonella.dispar.5608c3e6c9de9ceb79610e7786bd0ac4"                             
## [239] "X.Bacteria.Firmicutes.Clostridia.Clostridiales.Veillonellaceae.Veillonella.parvula.d3247c936f8f4735909a8526ebf2f49e"                            
## [240] "X.Bacteria.Firmicutes.Clostridia.Clostridiales.Veillonellaceae.Veillonella.dispar.5b4f8b625d8fbb1268863be7dbc4db5d"                             
## [241] "X.Bacteria.Firmicutes.Clostridia.Clostridiales.Veillonellaceae.NA.NA.983a0806857e311ab381e91c3ac211af"                                          
## [242] "X.Bacteria.Firmicutes.Clostridia.Clostridiales..Mogibacteriaceae..NA.NA.97b46cdc630fb1c3097f961bb4f627e5"                                       
## [243] "X.Bacteria.Firmicutes.Clostridia.Clostridiales..Mogibacteriaceae..NA.NA.4119ee9eb78e6baa92065d886a5e24ac"                                       
## [244] "X.Bacteria.Firmicutes.Clostridia.Clostridiales..Mogibacteriaceae..NA.NA.4260927e3b266486b5d210135f0ef4f6"                                       
## [245] "X.Bacteria.Firmicutes.Erysipelotrichi.Erysipelotrichales.Erysipelotrichaceae.NA.NA.216aca81784431f49e9567d23f7391e8"                            
## [246] "X.Bacteria.Firmicutes.Erysipelotrichi.Erysipelotrichales.Erysipelotrichaceae.NA.NA.228ae07feb0040900651f7580168bd27"                            
## [247] "X.Bacteria.Firmicutes.Erysipelotrichi.Erysipelotrichales.Erysipelotrichaceae.NA.NA.f95cab37fba4160de15015f4d520839f"                            
## [248] "X.Bacteria.Firmicutes.Erysipelotrichi.Erysipelotrichales.Erysipelotrichaceae.NA.NA.0c3887592dd60a361d43c78e501ba495"                            
## [249] "X.Bacteria.Firmicutes.Erysipelotrichi.Erysipelotrichales.Erysipelotrichaceae.Bulleidia.moorei.e083e2f58987c5f8db5d4dd16ddde91f"                 
## [250] "X.Bacteria.Firmicutes.Erysipelotrichi.Erysipelotrichales.Erysipelotrichaceae.Holdemania.NA.84e9e2afcdae1240b3ce3267067e1879"                    
## [251] "X.Bacteria.Firmicutes.Erysipelotrichi.Erysipelotrichales.Erysipelotrichaceae.Holdemania.NA.bc53814d35b2f862562a1e865e923b33"                    
## [252] "X.Bacteria.Firmicutes.Clostridia.Clostridiales.NA.NA.NA.cc4ff6e54743c750e2efa569086a38cf"                                                       
## [253] "X.Bacteria.Firmicutes.Clostridia.Clostridiales.NA.NA.NA.fd2130b6a8a1db45c0f5381012b2e5e7"                                                       
## [254] "X.Bacteria.Firmicutes.Clostridia.Clostridiales.NA.NA.NA.df15aa00cffdc5235078831e54deb6db"                                                       
## [255] "X.Bacteria.Firmicutes.Clostridia.Clostridiales.NA.NA.NA.6b40524e525db04087e414f058bc1a5e"                                                       
## [256] "X.Bacteria.Firmicutes.Clostridia.Clostridiales.NA.NA.NA.c838eb5b3b3d9fd0dfecdf75547c3c63"                                                       
## [257] "X.Bacteria.Firmicutes.Clostridia.Clostridiales.NA.NA.NA.d917386c5f307a4f652e389dadd7cef5"                                                       
## [258] "X.Bacteria.Firmicutes.Clostridia.Clostridiales.Clostridiaceae.Clostridium.NA.5c82dc47435864e490625ae01151570e"                                  
## [259] "X.Bacteria.Firmicutes.Clostridia.Clostridiales.Ruminococcaceae.NA.NA.0437c9910becc153a3d7838fd8eaa64b"                                          
## [260] "X.Bacteria.Firmicutes.Clostridia.Clostridiales.NA.NA.NA.dff390a58455e2599a43c5e400a35518"                                                       
## [261] "X.Bacteria.Firmicutes.Clostridia.Clostridiales.Ruminococcaceae.NA.NA.26f6853f46b06854fe5418317a261fb8"                                          
## [262] "X.Bacteria.Firmicutes.Clostridia.Clostridiales.Ruminococcaceae.NA.NA.6013b9e15b35c4e724eb0c323c05f9d3"                                          
## [263] "X.Bacteria.Firmicutes.Clostridia.Clostridiales.Ruminococcaceae.Oscillospira.NA.a7283edda8770d12b4e25d4dcce112c4"                                
## [264] "X.Bacteria.Firmicutes.Clostridia.Clostridiales.Ruminococcaceae.Oscillospira.NA.e13823e500387439450b3826ea191948"                                
## [265] "X.Bacteria.Firmicutes.Clostridia.Clostridiales.Ruminococcaceae.Oscillospira.NA.cb63ff338fde1171b1e985e8211b1929"                                
## [266] "X.Bacteria.Firmicutes.Clostridia.Clostridiales.Ruminococcaceae.Oscillospira.NA.d243b9b1c0b782aedbaa68fa5697193c"                                
## [267] "X.Bacteria.Firmicutes.Clostridia.Clostridiales.Ruminococcaceae.Oscillospira.NA.680d83cb233cffcc6405e08c46982042"                                
## [268] "X.Bacteria.Firmicutes.Clostridia.Clostridiales.Ruminococcaceae.Oscillospira.NA.7c93315259ad14510b6e9cae0670a048"                                
## [269] "X.Bacteria.Firmicutes.Clostridia.Clostridiales.Ruminococcaceae.NA.NA.e803ff46adaa0fa149ef151b082378a0"                                          
## [270] "X.Bacteria.Firmicutes.Clostridia.Clostridiales.Ruminococcaceae.NA.NA.63027647b049a274c881df23a4611b35"                                          
## [271] "X.Bacteria.Firmicutes.Clostridia.Clostridiales.Ruminococcaceae.NA.NA.c4995645e0c1545b0e1620144d03772b"                                          
## [272] "X.Bacteria.Firmicutes.Clostridia.Clostridiales.Ruminococcaceae.Oscillospira.NA.f2c958dfcac880c2f53a47e114d994d8"                                
## [273] "X.Bacteria.Firmicutes.Clostridia.Clostridiales.Ruminococcaceae.Oscillospira.NA.9e1d23e474a9f1e4e10387a23f601dde"                                
## [274] "X.Bacteria.Firmicutes.Clostridia.Clostridiales.Ruminococcaceae.Oscillospira.NA.4ef2b8e0ed22c2a986f53572f9e9405b"                                
## [275] "X.Bacteria.Firmicutes.Clostridia.Clostridiales.Ruminococcaceae.Oscillospira.NA.04a802674683d317a8c7484ce2407ce0"                                
## [276] "X.Bacteria.Firmicutes.Clostridia.Clostridiales.Ruminococcaceae.Oscillospira.NA.3e2bfdd1acae4723c96e344f91b262d8"                                
## [277] "X.Bacteria.Firmicutes.Clostridia.Clostridiales.Ruminococcaceae.NA.NA.2673143ffb9d8ff3ad82fa40e325973c"                                          
## [278] "X.Bacteria.Firmicutes.Clostridia.Clostridiales.Ruminococcaceae.Ruminococcus.bromii.fc02af89d869043b296114729009a5b0"                            
## [279] "X.Bacteria.Firmicutes.Clostridia.Clostridiales.Ruminococcaceae.Ruminococcus.NA.98ff1f2b1008b5e0971997cd5070fc03"                                
## [280] "X.Bacteria.Firmicutes.Clostridia.Clostridiales.Ruminococcaceae.Ruminococcus.NA.16ca411ff5a7131287f6deb7fc13b260"                                
## [281] "X.Bacteria.Firmicutes.Clostridia.Clostridiales.Ruminococcaceae.Ruminococcus.NA.153c48635771b1fdd766fc9beee8d0ab"                                
## [282] "X.Bacteria.Firmicutes.Clostridia.Clostridiales.Ruminococcaceae.Ruminococcus.NA.b61b28e1ab92f5cc8effcdde01c2ca90"                                
## [283] "X.Bacteria.Firmicutes.Clostridia.Clostridiales.Ruminococcaceae.Ruminococcus.NA.d048172b7b49bf1669197f10389312cd"                                
## [284] "X.Bacteria.Firmicutes.Clostridia.Clostridiales.Ruminococcaceae.Ruminococcus.NA.8aa94826eb4f90512d52fc7b796354c6"                                
## [285] "X.Bacteria.Firmicutes.Clostridia.Clostridiales.Ruminococcaceae.Faecalibacterium.prausnitzii.e553b9a0bb32467c71c89a4e97e55792"                   
## [286] "X.Bacteria.Firmicutes.Clostridia.Clostridiales.Ruminococcaceae.Faecalibacterium.prausnitzii.aed3f59201e3b9d21858f36557f42a80"                   
## [287] "X.Bacteria.Firmicutes.Clostridia.Clostridiales.Ruminococcaceae.Faecalibacterium.prausnitzii.a1a300c872047a7d8b6b294b17cfb63b"                   
## [288] "X.Bacteria.Firmicutes.Clostridia.Clostridiales.Ruminococcaceae.Faecalibacterium.prausnitzii.7859f0d526ad56fb4d8383d136b3bae6"                   
## [289] "X.Bacteria.Firmicutes.Clostridia.Clostridiales.Ruminococcaceae.Faecalibacterium.prausnitzii.23fed68c6c76ab10ba1be8a43e9176e7"                   
## [290] "X.Bacteria.Firmicutes.Clostridia.Clostridiales.Ruminococcaceae.Faecalibacterium.prausnitzii.c3bdda568b2c1580d5cce7407ef43909"                   
## [291] "X.Bacteria.Firmicutes.Clostridia.Clostridiales.Ruminococcaceae.Faecalibacterium.prausnitzii.a3f36ef32153f2fc2aaeac2feb23777f"                   
## [292] "X.Bacteria.Firmicutes.Clostridia.Clostridiales.Ruminococcaceae.NA.NA.c88e37c00a5abc483dada3afc5da38a3"                                          
## [293] "X.Bacteria.Firmicutes.Clostridia.Clostridiales.Ruminococcaceae.Ruminococcus.NA.98d5ae8840ae0914453cf0dd060d5f3a"                                
## [294] "X.Bacteria.Firmicutes.Clostridia.Clostridiales.Ruminococcaceae.Ruminococcus.NA.ee94b042fce1e0d75af093d7d1b1d340"                                
## [295] "X.Bacteria.Firmicutes.Clostridia.Clostridiales.Ruminococcaceae.Ruminococcus.NA.2308ced64b9c57f440230f7ddfeecda2"                                
## [296] "X.Bacteria.Firmicutes.Clostridia.Clostridiales.Ruminococcaceae.NA.NA.d4b257db9faad8674d68540e2c755d40"                                          
## [297] "X.Bacteria.Firmicutes.Clostridia.Clostridiales.Lachnospiraceae.NA.NA.bc8fb99545fba27899a16f1ade967276"                                          
## [298] "X.Bacteria.Firmicutes.Clostridia.Clostridiales.Lachnospiraceae..Ruminococcus..gnavus.90a05d597112b554e4480a8eaae4e0aa"                          
## [299] "X.Bacteria.Firmicutes.Clostridia.Clostridiales.Lachnospiraceae..Ruminococcus..gnavus.cc2d96099f530b503371e5ddca8c0a58"                          
## [300] "X.Bacteria.Firmicutes.Clostridia.Clostridiales.Lachnospiraceae.NA.NA.8b8b93b27e9c6cc58aacb0bf38248700"                                          
## [301] "X.Bacteria.Firmicutes.Clostridia.Clostridiales.Lachnospiraceae.Anaerostipes.NA.75622b8ee0f6a2b8a796bbfd264ca9fa"                                
## [302] "X.Bacteria.Firmicutes.Clostridia.Clostridiales.Lachnospiraceae.NA.NA.ac04fef6eb13ddf89756a0af35b512fe"                                          
## [303] "X.Bacteria.Firmicutes.Clostridia.Clostridiales.Lachnospiraceae.Roseburia.NA.c335a7c6e1e3f03c2c532274ec80a0c5"                                   
## [304] "X.Bacteria.Firmicutes.Clostridia.Clostridiales.Lachnospiraceae.Roseburia.NA.7d285be20e3ad3812eb21be379357ef1"                                   
## [305] "X.Bacteria.Firmicutes.Clostridia.Clostridiales.Lachnospiraceae.Roseburia.faecis.2d34c22edce4b1f2d8a5228ad78f8ea8"                               
## [306] "X.Bacteria.Firmicutes.Clostridia.Clostridiales.Lachnospiraceae.Coprococcus.NA.58c9d27620c4a8f749c75f945fce11a0"                                 
## [307] "X.Bacteria.Firmicutes.Clostridia.Clostridiales.Lachnospiraceae.Coprococcus.catus.fc2eeb02cc0e0e7f598b88912ae6c891"                              
## [308] "X.Bacteria.Firmicutes.Clostridia.Clostridiales.Lachnospiraceae.Coprococcus.eutactus.64032e2f2fa5269bd7d15eb19bdc94a9"                           
## [309] "X.Bacteria.Firmicutes.Clostridia.Clostridiales.Lachnospiraceae.Coprococcus.eutactus.53ee2a5c83806c73f3e5419690ac4241"                           
## [310] "X.Bacteria.Firmicutes.Clostridia.Clostridiales.Lachnospiraceae.Coprococcus.NA.cd287f4187715ce042afa01534b95a9b"                                 
## [311] "X.Bacteria.Firmicutes.Clostridia.Clostridiales.Lachnospiraceae.Lachnospira.NA.8e90ac2f0df6304022cff8cd278f8218"                                 
## [312] "X.Bacteria.Firmicutes.Clostridia.Clostridiales.Lachnospiraceae.Lachnospira.NA.04e9151eebd435f26965d2a465f88599"                                 
## [313] "X.Bacteria.Firmicutes.Clostridia.Clostridiales.Lachnospiraceae.Lachnospira.NA.5610b04f19aaac78ac2469a3feb707e9"                                 
## [314] "X.Bacteria.Firmicutes.Clostridia.Clostridiales.Lachnospiraceae.Lachnospira.NA.d32f24bb89dd024a38ac0a7a5962c771"                                 
## [315] "X.Bacteria.Firmicutes.Clostridia.Clostridiales.Lachnospiraceae.Lachnospira.NA.3cfa76e0da34a56a641ca33770efb588"                                 
## [316] "X.Bacteria.Firmicutes.Clostridia.Clostridiales.Lachnospiraceae.Coprococcus.NA.c9ea71f39bda8752713c8e90dff2b875"                                 
## [317] "X.Bacteria.Firmicutes.Clostridia.Clostridiales.Lachnospiraceae.Coprococcus.NA.00979c6b417b7978c1c656e263cdd054"                                 
## [318] "X.Bacteria.Firmicutes.Clostridia.Clostridiales.NA.NA.NA.504b5f0d2545c712940d4b9772a89711"                                                       
## [319] "X.Bacteria.Firmicutes.Clostridia.Clostridiales.Lachnospiraceae.NA.NA.fcd2073fc9628403d2dc4aa2b66b64a5"                                          
## [320] "X.Bacteria.Firmicutes.Clostridia.Clostridiales.Lachnospiraceae.NA.NA.fda53e1a26f7489a683cfe01228fca8f"                                          
## [321] "X.Bacteria.Firmicutes.Clostridia.Clostridiales.Lachnospiraceae.NA.NA.470dd02996e31255357eeeaa3672b469"                                          
## [322] "X.Bacteria.Firmicutes.Clostridia.Clostridiales.Lachnospiraceae.NA.NA.fb798da6858b8b3f34077164a4ec9315"                                          
## [323] "X.Bacteria.Firmicutes.Clostridia.Clostridiales.Lachnospiraceae.NA.NA.f54512749b97be9497134dc28c0af837"                                          
## [324] "X.Bacteria.Firmicutes.Clostridia.Clostridiales.Lachnospiraceae.NA.NA.51dd453b71a4aba20d9f349601ea5203"                                          
## [325] "X.Bacteria.Firmicutes.Clostridia.Clostridiales.Lachnospiraceae.NA.NA.dffa04ca171f459056b9466d7acfe601"                                          
## [326] "X.Bacteria.Firmicutes.Clostridia.Clostridiales.Lachnospiraceae.NA.NA.b20c095fd654b84cebdbfe4faa0a1409"                                          
## [327] "X.Bacteria.Firmicutes.Clostridia.Clostridiales.Lachnospiraceae.NA.NA.d5c7d97e6f4f5789d574d321dcca0992"                                          
## [328] "X.Bacteria.Firmicutes.Clostridia.Clostridiales.Lachnospiraceae..Ruminococcus..NA.00565f9981f176f2eae21aff895a72ff"                              
## [329] "X.Bacteria.Firmicutes.Clostridia.Clostridiales.Lachnospiraceae..Ruminococcus..NA.6a6fcf8f9b8bb1ab9e5f8456ee7fb109"                              
## [330] "X.Bacteria.Firmicutes.Clostridia.Clostridiales.Lachnospiraceae..Ruminococcus..torques.e59405a47acbc248ce61395366159d8d"                         
## [331] "X.Bacteria.Firmicutes.Clostridia.Clostridiales.Lachnospiraceae.NA.NA.694d61b8ec78349749c8b6ea59938e0b"                                          
## [332] "X.Bacteria.Firmicutes.Clostridia.Clostridiales.Lachnospiraceae.Blautia.obeum.ee293984c0110b2eeceb8427fdf448fb"                                  
## [333] "X.Bacteria.Firmicutes.Clostridia.Clostridiales.Lachnospiraceae.Blautia.NA.c1dc9ad5116d96b8ed863458fc0d0aec"                                     
## [334] "X.Bacteria.Firmicutes.Clostridia.Clostridiales.Lachnospiraceae.Blautia.NA.e9703768a50971c05b34b102810fd761"                                     
## [335] "X.Bacteria.Firmicutes.Clostridia.Clostridiales.Lachnospiraceae.Blautia.NA.9f8668eb1c5f9d9a992dd49245db090e"                                     
## [336] "X.Bacteria.Firmicutes.Clostridia.Clostridiales.Lachnospiraceae.Blautia.NA.7082034a5eec4c381d09cd380594242e"                                     
## [337] "X.Bacteria.Firmicutes.Clostridia.Clostridiales.Lachnospiraceae.Blautia.NA.a0a476401d529ced24788bff2ffd4515"                                     
## [338] "X.Bacteria.Firmicutes.Clostridia.Clostridiales.Lachnospiraceae.Blautia.NA.dc2721103659fe9f1d3ead56a11df243"                                     
## [339] "X.Bacteria.Firmicutes.Clostridia.Clostridiales.Lachnospiraceae.NA.NA.7e048b0e0c32d2f839e9f2098a4ebb35"                                          
## [340] "X.Bacteria.Firmicutes.Clostridia.Clostridiales.Lachnospiraceae.NA.NA.a4e94137d3eea12b54dec05caedafe47"                                          
## [341] "X.Bacteria.Firmicutes.Clostridia.Clostridiales.Lachnospiraceae.NA.NA.ebbe8017bca36f0a3b02532a2b4ed0d2"                                          
## [342] "X.Bacteria.Firmicutes.Clostridia.Clostridiales.Lachnospiraceae.NA.NA.eb61cae65bc6cdd2440323bbf603ba5c"                                          
## [343] "X.Bacteria.Firmicutes.Clostridia.Clostridiales.Lachnospiraceae.NA.NA.d9681bf8373e2635323f1ca0cc434ecf"                                          
## [344] "X.Bacteria.Firmicutes.Clostridia.Clostridiales.Lachnospiraceae.NA.NA.8a64e8e18be5e17d8ab9d76bb40f65d4"                                          
## [345] "X.Bacteria.Firmicutes.Clostridia.Clostridiales.Lachnospiraceae.NA.NA.78314aca268ae4422bc651192fbc986d"                                          
## [346] "X.Bacteria.Firmicutes.Clostridia.Clostridiales.Lachnospiraceae.NA.NA.c5465580c4c4cdd15a78de5a240dbd4e"                                          
## [347] "X.Bacteria.Firmicutes.Clostridia.Clostridiales.Lachnospiraceae.Dorea.formicigenerans.afd87e82de329a1ed75b98b5b606843c"                          
## [348] "X.Bacteria.Firmicutes.Clostridia.Clostridiales.Lachnospiraceae.Dorea.NA.a0b7d83fb64749f9a4b15b3728425b97"                                       
## [349] "X.Bacteria.Firmicutes.Clostridia.Clostridiales.Lachnospiraceae.Dorea.NA.9d21d7d00d7e938a7e1c374a4f4bb833"                                       
## [350] "X.Bacteria.Firmicutes.Clostridia.Clostridiales.Lachnospiraceae.NA.NA.752379aadaf3d2d89b4b240127b2e797"                                          
## [351] "X.Bacteria.Firmicutes.Clostridia.Clostridiales.Lachnospiraceae.NA.NA.392aa3c45e128c7432f91b3de84541f6"
```

```
meta_paired <- data_paired[,1:153]
IgA_paired <- data_paired[,154:350]

dim(IgA_paired)
```

```
## [1]  72 197
```

```
dim(meta_paired)
```

```
## [1]  72 153
```

```
IgA_paired_t <- as.data.frame(t(IgA_paired))

#Pre vs Post, full dataset

MW.p = apply(IgA_paired_t,1,
             function(x) wilcox.test(c(x)~meta_paired$SampleType, paired=TRUE)$p.value)
p.res = data.frame(row.names(IgA_paired_t),MW.p)
# Perform multiple comparison correction using a given method of choice
p.res$rel.fdr <- p.adjust(p.res$MW.p, method="fdr") #the same as benjamini-hochberg

hits.all.iga <- filter(p.res, rel.fdr<0.05)
hits.all.iga
```

```
##                                                                                                                                                                                                                       row.names.IgA_paired_t.
## depth                                                                                                                                                                                                                                   depth
## X.Bacteria.Firmicutes.Clostridia.Clostridiales.Ruminococcaceae.NA.NA.63027647b049a274c881df23a4611b35                                   X.Bacteria.Firmicutes.Clostridia.Clostridiales.Ruminococcaceae.NA.NA.63027647b049a274c881df23a4611b35
## X.Bacteria.Firmicutes.Clostridia.Clostridiales.Ruminococcaceae.Ruminococcus.NA.98ff1f2b1008b5e0971997cd5070fc03               X.Bacteria.Firmicutes.Clostridia.Clostridiales.Ruminococcaceae.Ruminococcus.NA.98ff1f2b1008b5e0971997cd5070fc03
## X.Bacteria.Firmicutes.Clostridia.Clostridiales.Lachnospiraceae..Ruminococcus..gnavus.cc2d96099f530b503371e5ddca8c0a58   X.Bacteria.Firmicutes.Clostridia.Clostridiales.Lachnospiraceae..Ruminococcus..gnavus.cc2d96099f530b503371e5ddca8c0a58
## X.Bacteria.Firmicutes.Clostridia.Clostridiales.Lachnospiraceae.NA.NA.ac04fef6eb13ddf89756a0af35b512fe                                   X.Bacteria.Firmicutes.Clostridia.Clostridiales.Lachnospiraceae.NA.NA.ac04fef6eb13ddf89756a0af35b512fe
## X.Bacteria.Firmicutes.Clostridia.Clostridiales.Lachnospiraceae.NA.NA.d5c7d97e6f4f5789d574d321dcca0992                                   X.Bacteria.Firmicutes.Clostridia.Clostridiales.Lachnospiraceae.NA.NA.d5c7d97e6f4f5789d574d321dcca0992
## X.Bacteria.Firmicutes.Clostridia.Clostridiales.Lachnospiraceae..Ruminococcus..torques.e59405a47acbc248ce61395366159d8d X.Bacteria.Firmicutes.Clostridia.Clostridiales.Lachnospiraceae..Ruminococcus..torques.e59405a47acbc248ce61395366159d8d
## X.Bacteria.Firmicutes.Clostridia.Clostridiales.Lachnospiraceae.Blautia.NA.9f8668eb1c5f9d9a992dd49245db090e                         X.Bacteria.Firmicutes.Clostridia.Clostridiales.Lachnospiraceae.Blautia.NA.9f8668eb1c5f9d9a992dd49245db090e
## X.Bacteria.Firmicutes.Clostridia.Clostridiales.Lachnospiraceae.Dorea.NA.a0b7d83fb64749f9a4b15b3728425b97                             X.Bacteria.Firmicutes.Clostridia.Clostridiales.Lachnospiraceae.Dorea.NA.a0b7d83fb64749f9a4b15b3728425b97
##                                                                                                                                MW.p
## depth                                                                                                                  8.471511e-05
## X.Bacteria.Firmicutes.Clostridia.Clostridiales.Ruminococcaceae.NA.NA.63027647b049a274c881df23a4611b35                  4.813508e-04
## X.Bacteria.Firmicutes.Clostridia.Clostridiales.Ruminococcaceae.Ruminococcus.NA.98ff1f2b1008b5e0971997cd5070fc03        9.750295e-04
## X.Bacteria.Firmicutes.Clostridia.Clostridiales.Lachnospiraceae..Ruminococcus..gnavus.cc2d96099f530b503371e5ddca8c0a58  1.969091e-03
## X.Bacteria.Firmicutes.Clostridia.Clostridiales.Lachnospiraceae.NA.NA.ac04fef6eb13ddf89756a0af35b512fe                  1.643188e-03
## X.Bacteria.Firmicutes.Clostridia.Clostridiales.Lachnospiraceae.NA.NA.d5c7d97e6f4f5789d574d321dcca0992                  1.862343e-03
## X.Bacteria.Firmicutes.Clostridia.Clostridiales.Lachnospiraceae..Ruminococcus..torques.e59405a47acbc248ce61395366159d8d 4.741700e-06
## X.Bacteria.Firmicutes.Clostridia.Clostridiales.Lachnospiraceae.Blautia.NA.9f8668eb1c5f9d9a992dd49245db090e             6.386773e-04
## X.Bacteria.Firmicutes.Clostridia.Clostridiales.Lachnospiraceae.Dorea.NA.a0b7d83fb64749f9a4b15b3728425b97               1.077432e-03
##                                                                                                                             rel.fdr
## depth                                                                                                                  0.0083444383
## X.Bacteria.Firmicutes.Clostridia.Clostridiales.Ruminococcaceae.NA.NA.63027647b049a274c881df23a4611b35                  0.0314548574
## X.Bacteria.Firmicutes.Clostridia.Clostridiales.Ruminococcaceae.Ruminococcus.NA.98ff1f2b1008b5e0971997cd5070fc03        0.0353756818
## X.Bacteria.Firmicutes.Clostridia.Clostridiales.Lachnospiraceae..Ruminococcus..gnavus.cc2d96099f530b503371e5ddca8c0a58  0.0431012134
## X.Bacteria.Firmicutes.Clostridia.Clostridiales.Lachnospiraceae.NA.NA.ac04fef6eb13ddf89756a0af35b512fe                  0.0431012134
## X.Bacteria.Firmicutes.Clostridia.Clostridiales.Lachnospiraceae.NA.NA.d5c7d97e6f4f5789d574d321dcca0992                  0.0431012134
## X.Bacteria.Firmicutes.Clostridia.Clostridiales.Lachnospiraceae..Ruminococcus..torques.e59405a47acbc248ce61395366159d8d 0.0009341149
## X.Bacteria.Firmicutes.Clostridia.Clostridiales.Lachnospiraceae.Blautia.NA.9f8668eb1c5f9d9a992dd49245db090e             0.0314548574
## X.Bacteria.Firmicutes.Clostridia.Clostridiales.Lachnospiraceae.Dorea.NA.a0b7d83fb64749f9a4b15b3728425b97               0.0353756818
```

```
hits.all.iga.relaxed <- filter(p.res, rel.fdr<0.1)
```

Plot hits

```
#Ruminococcaceae 630
pdata <- data.frame(IgA_paired$`X.Bacteria.Firmicutes.Clostridia.Clostridiales.Ruminococcaceae.NA.NA.63027647b049a274c881df23a4611b35`
                    ,meta_paired$SampleType2, meta_paired$Donor)
colnames(pdata) <- c("IgAIndex", "SampleType", "Donor")
p <- ggplot(pdata) + geom_boxplot(aes(x=SampleType, y=IgAIndex), 
                                  outlier.shape=NA, outlier.size=NA)
p <- p + geom_jitter(aes(x=SampleType, y=IgAIndex),
                     width=0.2, height=0, shape=1, size=3)
p <- p + ggtitle("Ruminococcaceae 6302")
p <- p + theme(axis.text.x  = element_text(angle=90, vjust=0.5, size=6), plot.title = element_text(size = 9))
p <- p + theme_bw(base_size=16)
#p <- p + scale_fill_manual(values=c("indianred3", "deepskyblue3"))
p <- p + ylim(c(-1, 1))
p <- p + annotate("text", x = 1.5, y=1, label = "*", size=4)
p <- p + annotate("segment", x=1, xend=2, y=0.9, yend=0.9)
p <- p + guides(colour=FALSE, size=FALSE, shape=FALSE, fill=FALSE)
p
```

```
#Ruminococcus 98ff
pdata <- data.frame(IgA_paired$`X.Bacteria.Firmicutes.Clostridia.Clostridiales.Ruminococcaceae.Ruminococcus.NA.98ff1f2b1008b5e0971997cd5070fc03`
                    ,meta_paired$SampleType2, meta_paired$Donor)
colnames(pdata) <- c("IgAIndex", "SampleType", "Donor")
p <- ggplot(pdata) + geom_boxplot(aes(x=SampleType, y=IgAIndex), 
                                  outlier.shape=NA, outlier.size=NA)
p <- p + geom_jitter(aes(x=SampleType, y=IgAIndex),
                     width=0.2, height=0, shape=1, size=3)
p <- p + ggtitle("Ruminococcus 98ff")
p <- p + theme(axis.text.x  = element_text(angle=90, vjust=0.5, size=6), plot.title = element_text(size = 9))
p <- p + theme_bw(base_size=16)
#p <- p + scale_fill_manual(values=c("indianred3", "deepskyblue3"))
p <- p + ylim(c(-1, 1))
p <- p + annotate("text", x = 1.5, y=1, label = "*", size=4)
p <- p + annotate("segment", x=1, xend=2, y=0.9, yend=0.9)
p <- p + guides(colour=FALSE, size=FALSE, shape=FALSE, fill=FALSE)
p
```

```
#Lachnospiraceae d5c
pdata <- data.frame(IgA_paired$`X.Bacteria.Firmicutes.Clostridia.Clostridiales.Lachnospiraceae.NA.NA.d5c7d97e6f4f5789d574d321dcca0992`
                    ,meta_paired$SampleType2, meta_paired$Donor)
colnames(pdata) <- c("IgAIndex", "SampleType", "Donor")
p <- ggplot(pdata) + geom_boxplot(aes(x=SampleType, y=IgAIndex), 
                                  outlier.shape=NA, outlier.size=NA)
p <- p + geom_jitter(aes(x=SampleType, y=IgAIndex),
                     width=0.2, height=0, shape=1, size=3)
p <- p + ggtitle("Lachnospiraceae d5c")
p <- p + theme(axis.text.x  = element_text(angle=90, vjust=0.5, size=6), plot.title = element_text(size = 9))
p <- p + theme_bw(base_size=16)
#p <- p + scale_fill_manual(values=c("indianred3", "deepskyblue3"))
p <- p + ylim(c(-1, 1))
p <- p + annotate("text", x = 1.5, y=1, label = "*", size=4)
p <- p + annotate("segment", x=1, xend=2, y=0.9, yend=0.9)
p <- p + guides(colour=FALSE, size=FALSE, shape=FALSE, fill=FALSE)
p
```

```
#Lachnospiraceae ac0
pdata <- data.frame(IgA_paired$`X.Bacteria.Firmicutes.Clostridia.Clostridiales.Lachnospiraceae.NA.NA.ac04fef6eb13ddf89756a0af35b512fe`
                    ,meta_paired$SampleType2, meta_paired$Donor)
colnames(pdata) <- c("IgAIndex", "SampleType", "Donor")
p <- ggplot(pdata) + geom_boxplot(aes(x=SampleType, y=IgAIndex), 
                                  outlier.shape=NA, outlier.size=NA)
p <- p + geom_jitter(aes(x=SampleType, y=IgAIndex),
                     width=0.2, height=0, shape=1, size=3)
p <- p + ggtitle("Lachnospiraceae ac0")
p <- p + theme(axis.text.x  = element_text(angle=90, vjust=0.5, size=6), plot.title = element_text(size = 9))
p <- p + theme_bw(base_size=16)
#p <- p + scale_fill_manual(values=c("indianred3", "deepskyblue3"))
p <- p + ylim(c(-1, 1))
p <- p + annotate("text", x = 1.5, y=1, label = "*", size=4)
p <- p + annotate("segment", x=1, xend=2, y=0.9, yend=0.9)
p <- p + guides(colour=FALSE, size=FALSE, shape=FALSE, fill=FALSE)
p
```

```
#Ruminococcus torques e59
pdata <- data.frame(IgA_paired$`X.Bacteria.Firmicutes.Clostridia.Clostridiales.Lachnospiraceae..Ruminococcus..torques.e59405a47acbc248ce61395366159d8d`
                    ,meta_paired$SampleType2, meta_paired$Donor)
colnames(pdata) <- c("IgAIndex", "SampleType", "Donor")
p <- ggplot(pdata) + geom_boxplot(aes(x=SampleType, y=IgAIndex), 
                                  outlier.shape=NA, outlier.size=NA)
p <- p + geom_jitter(aes(x=SampleType, y=IgAIndex),
                     width=0.2, height=0, shape=1, size=3)
p <- p + ggtitle("Ruminococcus torques e59")
p <- p + theme(axis.text.x  = element_text(angle=90, vjust=0.5, size=6), plot.title = element_text(size = 9))
p <- p + theme_bw(base_size=16)
#p <- p + scale_fill_manual(values=c("indianred3", "deepskyblue3"))
p <- p + ylim(c(-1, 1))
p <- p + annotate("text", x = 1.5, y=1, label = "**", size=4)
p <- p + annotate("segment", x=1, xend=2, y=0.9, yend=0.9)
p <- p + guides(colour=FALSE, size=FALSE, shape=FALSE, fill=FALSE)
p
```

# Differences in abundance estimate between pre & post transplant (paired analysis)

Test

```
row.names(Abund_settrim75_t)==gsub("-", ".", metaIgA$SampleID)
```

```
##  [1] TRUE TRUE TRUE TRUE TRUE TRUE TRUE TRUE TRUE TRUE TRUE TRUE TRUE TRUE TRUE
## [16] TRUE TRUE TRUE TRUE TRUE TRUE TRUE TRUE TRUE TRUE TRUE TRUE TRUE TRUE TRUE
## [31] TRUE TRUE TRUE TRUE TRUE TRUE TRUE TRUE TRUE TRUE TRUE TRUE TRUE TRUE TRUE
## [46] TRUE TRUE TRUE TRUE TRUE TRUE TRUE TRUE TRUE TRUE TRUE TRUE TRUE TRUE TRUE
## [61] TRUE TRUE TRUE TRUE TRUE TRUE TRUE TRUE TRUE TRUE TRUE TRUE TRUE TRUE TRUE
## [76] TRUE TRUE
```

```
data_adonis <- data.frame(metaIgA, Abund_settrim75_t)

data_paired <- data_adonis[which(data_adonis$Patient %in% metaIgAPre$Patient & data_adonis$Patient %in% metaIgAPost$Patient),]
data_paired <-  data_paired[order(data_paired$Patient),]
data_paired <-  data_paired[order(data_paired$SampleType),]
names(data_paired)
```

```
##   [1] "SampleID"                                                                                                                                       
##   [2] "Sort"                                                                                                                                           
##   [3] "Patient"                                                                                                                                        
##   [4] "Stool_no"                                                                                                                                       
##   [5] "SampleType"                                                                                                                                     
##   [6] "Patient_Number"                                                                                                                                 
##   [7] "Gender"                                                                                                                                         
##   [8] "DOB"                                                                                                                                            
##   [9] "Tx_Date"                                                                                                                                        
##  [10] "Donor"                                                                                                                                          
##  [11] "IBD"                                                                                                                                            
##  [12] "Delivery"                                                                                                                                       
##  [13] "Stool.no..t0"                                                                                                                                   
##  [14] "Stool.no..t4"                                                                                                                                   
##  [15] "Age_at_FMT"                                                                                                                                     
##  [16] "Height_.cm."                                                                                                                                    
##  [17] "Height_.m."                                                                                                                                     
##  [18] "FMT_Date"                                                                                                                                       
##  [19] "Cap_vs._Col"                                                                                                                                    
##  [20] "Wt_Post.CDI"                                                                                                                                    
##  [21] "Wt_Loss"                                                                                                                                        
##  [22] "Wt_Pre.CDI"                                                                                                                                     
##  [23] "X._Wt_Loss"                                                                                                                                     
##  [24] "BMI_Pre.CDI"                                                                                                                                    
##  [25] "Wt_Post.FMT_1"                                                                                                                                  
##  [26] "BMI_1"                                                                                                                                          
##  [27] "Wt_Post.FMT_4"                                                                                                                                  
##  [28] "BMI_4"                                                                                                                                          
##  [29] "Wt_Post.FMT_12"                                                                                                                                 
##  [30] "BMI_12"                                                                                                                                         
##  [31] "Wt_Post.FMT_24"                                                                                                                                 
##  [32] "BMI_24"                                                                                                                                         
##  [33] "Wt_Post.FMT_48"                                                                                                                                 
##  [34] "BMI_48"                                                                                                                                         
##  [35] "Data_collection"                                                                                                                                
##  [36] "Percent_IgA"                                                                                                                                    
##  [37] "Sort_Day"                                                                                                                                       
##  [38] "Column1"                                                                                                                                        
##  [39] "Column2"                                                                                                                                        
##  [40] "Cd_.ng.ml."                                                                                                                                     
##  [41] "Co_.ng.ml."                                                                                                                                     
##  [42] "Cu_.ng.ml."                                                                                                                                     
##  [43] "Fe_.ug.ml."                                                                                                                                     
##  [44] "Mg_.ug.ml."                                                                                                                                     
##  [45] "Mn_.ng.ml."                                                                                                                                     
##  [46] "Ni_.ng.ml."                                                                                                                                     
##  [47] "Pb_.ng.ml."                                                                                                                                     
##  [48] "Se_.ng.ml."                                                                                                                                     
##  [49] "Zn_.ug.ml."                                                                                                                                     
##  [50] "Visit"                                                                                                                                          
##  [51] "Randomization_Group"                                                                                                                            
##  [52] "Age_at_Tx"                                                                                                                                      
##  [53] "Sex"                                                                                                                                            
##  [54] "Cd2"                                                                                                                                            
##  [55] "Pb2"                                                                                                                                            
##  [56] "Original.ID"                                                                                                                                    
##  [57] "Participant"                                                                                                                                    
##  [58] "Timepoint"                                                                                                                                      
##  [59] "Serum_2.hydroxybutyrate"                                                                                                                        
##  [60] "Serum_2.methylbutyrate"                                                                                                                         
##  [61] "Serum_Acetate"                                                                                                                                  
##  [62] "Serum_Butyrate"                                                                                                                                 
##  [63] "Serum_Caproate"                                                                                                                                 
##  [64] "Serum_Isobutyrate"                                                                                                                              
##  [65] "Serum_Isovalerate"                                                                                                                              
##  [66] "Serum_Lactate"                                                                                                                                  
##  [67] "Serum_Propionate"                                                                                                                               
##  [68] "Serum_Total..without.valerate."                                                                                                                 
##  [69] "Serum_Valerate"                                                                                                                                 
##  [70] "Fecal_Acetate"                                                                                                                                  
##  [71] "Fecal_Butyrate"                                                                                                                                 
##  [72] "Fecal_Isobutyrate"                                                                                                                              
##  [73] "Fecal_Isovalerate"                                                                                                                              
##  [74] "Fecal_Propionate"                                                                                                                               
##  [75] "Fecal_Valerate"                                                                                                                                 
##  [76] "Fecal_Total.SCFAs"                                                                                                                              
##  [77] "Serum_Ursodeoxycholic_acid"                                                                                                                     
##  [78] "Serum_Ursocholanic_acid"                                                                                                                        
##  [79] "Serum_Tauroursodeoxycholic_acid"                                                                                                                
##  [80] "Serum_Taurolithocholic_acid"                                                                                                                    
##  [81] "Serum_Taurohyodeoxycholic_acid"                                                                                                                 
##  [82] "Serum_Taurohyocholic_acid"                                                                                                                      
##  [83] "Serum_Taurodeoxycholic_acid"                                                                                                                    
##  [84] "Serum_Taurocholic_acid"                                                                                                                         
##  [85] "Serum_Taurochenodeoxycholic_acid"                                                                                                               
##  [86] "Serum_Lithocholic_acid"                                                                                                                         
##  [87] "Serum_Lithocholenic_acid"                                                                                                                       
##  [88] "Serum_Isodeoxycholic_acid"                                                                                                                      
##  [89] "Serum_Hyodeoxycholic_acid"                                                                                                                      
##  [90] "Serum_Hyocholic_acid"                                                                                                                           
##  [91] "Serum_Glycoursodeoxycholic_acid_2"                                                                                                              
##  [92] "Serum_Glycoursodeoxycholic_acid_1"                                                                                                              
##  [93] "Serum_Glycoursodeoxycholic_acid"                                                                                                                
##  [94] "Serum_Glycolithocholic_acid"                                                                                                                    
##  [95] "Serum_Glycohyodeoxycholic_acid"                                                                                                                 
##  [96] "Serum_Glycohyocholic_acid"                                                                                                                      
##  [97] "Serum_Glycodeoxycholic_acid"                                                                                                                    
##  [98] "Serum_Glycocholic_acid"                                                                                                                         
##  [99] "Serum_Glycochenodeoxycholic_acid"                                                                                                               
## [100] "Serum_Deoxycholic_acid"                                                                                                                         
## [101] "Serum_Cholic_acid"                                                                                                                              
## [102] "Serum_Chenodeoxycholic_acid"                                                                                                                    
## [103] "Feces_Ursodeoxycholic_acid"                                                                                                                     
## [104] "Feces_Ursocholanic_acid"                                                                                                                        
## [105] "Feces_Tauroursodeoxycholic_acid"                                                                                                                
## [106] "Feces_Tauroursocholanic_acid"                                                                                                                   
## [107] "Feces_Taurolithocholic_acid"                                                                                                                    
## [108] "Feces_Taurohyodeoxycholic_acid"                                                                                                                 
## [109] "Feces_Taurohyocholic_acid"                                                                                                                      
## [110] "Feces_Taurodeoxycholic_acid"                                                                                                                    
## [111] "Feces_Taurocholic_acid"                                                                                                                         
## [112] "Feces_Taurochenodeoxycholic_acid"                                                                                                               
## [113] "Feces_Lithocholic_acid"                                                                                                                         
## [114] "Feces_Hyocholic_acid"                                                                                                                           
## [115] "Feces_Glycoursodeoxycholic_acid"                                                                                                                
## [116] "Feces_Glycolithocholic_acid"                                                                                                                    
## [117] "Feces_Glycohyodeoxycholic_acid"                                                                                                                 
## [118] "Feces_Glycohyocholic_acid"                                                                                                                      
## [119] "Feces_Glycodeoxycholic_acid"                                                                                                                    
## [120] "Feces_Glycocholic_acid"                                                                                                                         
## [121] "Feces_Glycochenodeoxycholic_acid"                                                                                                               
## [122] "Feces_Deoxycholic_acid"                                                                                                                         
## [123] "Feces_Cholic_acid"                                                                                                                              
## [124] "Feces_Chenodeoxycholic_Acid"                                                                                                                    
## [125] "Serum_secondary"                                                                                                                                
## [126] "Serum_primary"                                                                                                                                  
## [127] "Feces_secondary"                                                                                                                                
## [128] "Feces_primary"                                                                                                                                  
## [129] "Sample"                                                                                                                                         
## [130] "LB"                                                                                                                                             
## [131] "HB"                                                                                                                                             
## [132] "S0"                                                                                                                                             
## [133] "S1"                                                                                                                                             
## [134] "S2"                                                                                                                                             
## [135] "S3"                                                                                                                                             
## [136] "S4"                                                                                                                                             
## [137] "G0"                                                                                                                                             
## [138] "G1"                                                                                                                                             
## [139] "G2"                                                                                                                                             
## [140] "G3"                                                                                                                                             
## [141] "G4"                                                                                                                                             
## [142] "OM"                                                                                                                                             
## [143] "B"                                                                                                                                              
## [144] "CF"                                                                                                                                             
## [145] "AF"                                                                                                                                             
## [146] "Donor.ID"                                                                                                                                       
## [147] "Donor_StoolID"                                                                                                                                  
## [148] "fecal_twin"                                                                                                                                     
## [149] "fecal_twin_ID"                                                                                                                                  
## [150] "sample_sums"                                                                                                                                    
## [151] "Donor.ID3"                                                                                                                                      
## [152] "Donor.ID2"                                                                                                                                      
## [153] "SampleType2"                                                                                                                                    
## [154] "depth"                                                                                                                                          
## [155] "X.Bacteria.Bacteroidetes.Bacteroidia.Bacteroidales..Barnesiellaceae..NA.NA.5b0c52a1507deba61b41b7f9d8187386"                                    
## [156] "X.Bacteria.Bacteroidetes.Bacteroidia.Bacteroidales..Barnesiellaceae..NA.NA.9d0579ce6a317ca0e626a5c68981c725"                                    
## [157] "X.Bacteria.Bacteroidetes.Bacteroidia.Bacteroidales..Barnesiellaceae..NA.NA.e6cd2fee507eb45ff5f3c0a4191f3876"                                    
## [158] "X.Bacteria.Bacteroidetes.Bacteroidia.Bacteroidales..Barnesiellaceae..NA.NA.158782cefa6e28406a2a0aeb75220f0a"                                    
## [159] "X.Bacteria.Bacteroidetes.Bacteroidia.Bacteroidales.Porphyromonadaceae.Parabacteroides.NA.0d8b9177d01f328c2f5d5efa4c2acbc5"                      
## [160] "X.Bacteria.Bacteroidetes.Bacteroidia.Bacteroidales.Porphyromonadaceae.Parabacteroides.NA.e17d964cd0fd8c2ddbd1dacfa8b536e3"                      
## [161] "X.Bacteria.Bacteroidetes.Bacteroidia.Bacteroidales.Porphyromonadaceae.Parabacteroides.distasonis.2cdb259b754c2db622ed9fb5a6517a37"              
## [162] "X.Bacteria.Bacteroidetes.Bacteroidia.Bacteroidales.Porphyromonadaceae.Parabacteroides.distasonis.f4f297232da0f8d7dae7f9c432501e22"              
## [163] "X.Bacteria.Bacteroidetes.Bacteroidia.Bacteroidales.Porphyromonadaceae.Parabacteroides.distasonis.4844896c9c40eaca2fa0291163b9ca52"              
## [164] "X.Bacteria.Bacteroidetes.Bacteroidia.Bacteroidales.Bacteroidaceae.Bacteroides.ovatus.2c2018d4ecccfa3cb27b99a04c9222b1"                          
## [165] "X.Bacteria.Bacteroidetes.Bacteroidia.Bacteroidales.Bacteroidaceae.Bacteroides.ovatus.03af966ff07ddef2b87da992b85b600b"                          
## [166] "X.Bacteria.Bacteroidetes.Bacteroidia.Bacteroidales.Bacteroidaceae.Bacteroides.NA.51e441cbdcc80da0656e82293ae160b5"                              
## [167] "X.Bacteria.Bacteroidetes.Bacteroidia.Bacteroidales.Bacteroidaceae.Bacteroides.fragilis.9496d87b94d90dff068f0716603930bd"                        
## [168] "X.Bacteria.Bacteroidetes.Bacteroidia.Bacteroidales.Bacteroidaceae.Bacteroides.fragilis.35ffdd51464e2c68179717e5334a1d7e"                        
## [169] "X.Bacteria.Bacteroidetes.Bacteroidia.Bacteroidales.Bacteroidaceae.Bacteroides.caccae.ec4075339e16f5cd45fd5a7955596899"                          
## [170] "X.Bacteria.Bacteroidetes.Bacteroidia.Bacteroidales.Bacteroidaceae.Bacteroides.NA.25d727166a36df8d2f6a915a945bf5ac"                              
## [171] "X.Bacteria.Bacteroidetes.Bacteroidia.Bacteroidales.Bacteroidaceae.Bacteroides.NA.3b872d4cecb7a9437ce9e5add011b471"                              
## [172] "X.Bacteria.Bacteroidetes.Bacteroidia.Bacteroidales.Bacteroidaceae.Bacteroides.eggerthii.8ad4a56a5f526df40d97b371ba0ef33e"                       
## [173] "X.Bacteria.Bacteroidetes.Bacteroidia.Bacteroidales.Bacteroidaceae.Bacteroides.eggerthii.30dd9fd45122f82c12b166a042d1eaf9"                       
## [174] "X.Bacteria.Bacteroidetes.Bacteroidia.Bacteroidales.Bacteroidaceae.Bacteroides.uniformis.63b26504f32377cd78d6068bffb86b9a"                       
## [175] "X.Bacteria.Bacteroidetes.Bacteroidia.Bacteroidales.Bacteroidaceae.Bacteroides.uniformis.574ab9c17692ffd001643c930f6895f5"                       
## [176] "X.Bacteria.Bacteroidetes.Bacteroidia.Bacteroidales.Prevotellaceae.Prevotella.melaninogenica.d0b698c7298bf04110a6d2f220879bfb"                   
## [177] "X.Bacteria.Bacteroidetes.Bacteroidia.Bacteroidales..Paraprevotellaceae..NA.NA.85fddfaca6687f22a2b1c5ed45a6041c"                                 
## [178] "X.Bacteria.Bacteroidetes.Bacteroidia.Bacteroidales..Paraprevotellaceae..Paraprevotella.NA.37dac50f454a3cf754e83790eee77108"                     
## [179] "X.Bacteria.Bacteroidetes.Bacteroidia.Bacteroidales.Bacteroidaceae.Bacteroides.NA.b65eb19257f7a2bedb5a1c4b42aeb396"                              
## [180] "X.Bacteria.Bacteroidetes.Bacteroidia.Bacteroidales.Bacteroidaceae.Bacteroides.NA.a7a1a93ecfcef4cb45b42307a4fa3bca"                              
## [181] "X.Bacteria.Bacteroidetes.Bacteroidia.Bacteroidales.Bacteroidaceae.Bacteroides.NA.668fdb718997fc1589c7817655d4bb5f"                              
## [182] "X.Bacteria.Bacteroidetes.Bacteroidia.Bacteroidales..Odoribacteraceae..Butyricimonas.NA.0bb0e1802451aace7137a4d598f775f5"                        
## [183] "X.Bacteria.Bacteroidetes.Bacteroidia.Bacteroidales..Odoribacteraceae..Odoribacter.NA.3ea67969a01ec419ec1d4784d379ad1d"                          
## [184] "X.Bacteria.Bacteroidetes.Sphingobacteriia.Sphingobacteriales.Sphingobacteriaceae.Pedobacter.NA.e5e45fe5960b838c45765068621efcd5"                
## [185] "X.Bacteria.Bacteroidetes.Bacteroidia.Bacteroidales.Rikenellaceae.NA.NA.76d4dadc006ff1d530d38dd3cfac300c"                                        
## [186] "X.Bacteria.Bacteroidetes.Bacteroidia.Bacteroidales.Rikenellaceae.NA.NA.e47a63686b619f67783f9b9aa52b86bf"                                        
## [187] "X.Bacteria.Bacteroidetes.Bacteroidia.Bacteroidales.Rikenellaceae.NA.NA.619f64f2bf103286f4f70bfd89500ed4"                                        
## [188] "X.Bacteria.Bacteroidetes.Bacteroidia.Bacteroidales.Rikenellaceae.NA.NA.7534fb513a4b404419edc4e91920af3f"                                        
## [189] "X.Bacteria.Bacteroidetes.Bacteroidia.Bacteroidales.Rikenellaceae.NA.NA.a231814083060142816974207844eff2"                                        
## [190] "X.Bacteria.Bacteroidetes..Saprospirae...Saprospirales..Chitinophagaceae.Sediminibacterium.NA.372e9cfd6acc38cd00e88aac575f8afa"                  
## [191] "X.Bacteria.Bacteroidetes..Saprospirae...Saprospirales..Chitinophagaceae.Sediminibacterium.NA.a47ed77e3a3ab04be7139730ee5e5a98"                  
## [192] "X.Bacteria.Bacteroidetes..Saprospirae...Saprospirales..Chitinophagaceae.Sediminibacterium.NA.1ee27fee30b2e6832a2be5e28f0978f3"                  
## [193] "X.Bacteria.Chlamydiae.Chlamydiia.Chlamydiales.Parachlamydiaceae.Parachlamydia.NA.8a592a80ca5be07c6b8d28ae3f835dcd"                              
## [194] "X.Bacteria.Proteobacteria.Betaproteobacteria.Burkholderiales.Oxalobacteraceae.Ralstonia.NA.dde6d3e36af306c913854a9cf0ddf248"                    
## [195] "X.Bacteria.Proteobacteria.Betaproteobacteria.Burkholderiales.Alcaligenaceae.Sutterella.NA.0088553fbbbf2fbc8918ad224557d65c"                     
## [196] "X.Bacteria.Proteobacteria.Betaproteobacteria.Burkholderiales.Alcaligenaceae.Sutterella.NA.7d17d197d7978be0b6684209ce257e20"                     
## [197] "X.Bacteria.Proteobacteria.Gammaproteobacteria.Enterobacteriales.Enterobacteriaceae.NA.NA.677929eac52be081cdae08a0b9c70eb2"                      
## [198] "X.Bacteria.Proteobacteria.Gammaproteobacteria.Enterobacteriales.Enterobacteriaceae.NA.NA.bfe54af4c9180d37a0d76f6dafe79a5a"                      
## [199] "X.Bacteria.Proteobacteria.Gammaproteobacteria.Enterobacteriales.Enterobacteriaceae.Escherichia.coli.1b158b8b2922d4fcad5d9cea607cbb7d"           
## [200] "X.Bacteria.Proteobacteria.Gammaproteobacteria.Enterobacteriales.Enterobacteriaceae.Morganella.morganii.78056058faedd75d706633c5f55a975f"        
## [201] "X.Bacteria.Proteobacteria.Gammaproteobacteria.Xanthomonadales.Sinobacteraceae.NA.NA.09bedf1ee9478654b581fea611f919e7"                           
## [202] "X.Bacteria.Proteobacteria.Gammaproteobacteria.Pseudomonadales.Pseudomonadaceae.NA.NA.1f878f615fcfc8d7bd381a7841ac1e41"                          
## [203] "X.Bacteria.Proteobacteria.Gammaproteobacteria.Pseudomonadales.Pseudomonadaceae.Pseudomonas.veronii.402e5913597695a16d7cad415ffff02f"            
## [204] "X.Bacteria.Proteobacteria.Gammaproteobacteria.Xanthomonadales.Xanthomonadaceae.Stenotrophomonas.acidaminiphila.3588479e8e7443fdc3657f4b81cebf4c"
## [205] "X.Bacteria.Proteobacteria.Gammaproteobacteria.Xanthomonadales.Xanthomonadaceae.Stenotrophomonas.NA.891019e319f5d8d088ed4f9379fd63ef"            
## [206] "X.Bacteria.Proteobacteria.Deltaproteobacteria.Desulfovibrionales.Desulfovibrionaceae.Bilophila.NA.ece50a62168f85fc61385d8adb4c6494"             
## [207] "X.Bacteria.Proteobacteria.Deltaproteobacteria.Desulfovibrionales.Desulfovibrionaceae.Desulfovibrio.NA.dfd770d48651635c39bdafe13f62e75a"         
## [208] "X.Bacteria.Proteobacteria.Deltaproteobacteria.Myxococcales.0319.6G20.NA.NA.9f1414e2233d20717c5ed418bd88e872"                                    
## [209] "X.Bacteria.Actinobacteria.Coriobacteriia.Coriobacteriales.Coriobacteriaceae.Collinsella.aerofaciens.dfba68ef0fd0e712608eb2a0078013a7"           
## [210] "X.Bacteria.Actinobacteria.Coriobacteriia.Coriobacteriales.Coriobacteriaceae.Slackia.NA.c9a59a2ded38b324d6d58202a4137f9b"                        
## [211] "X.Bacteria.Actinobacteria.Coriobacteriia.Coriobacteriales.Coriobacteriaceae.Eggerthella.lenta.6bb6aad559c5eaeb22b2eef7874ef038"                 
## [212] "X.Bacteria.Actinobacteria.Coriobacteriia.Coriobacteriales.Coriobacteriaceae.NA.NA.23368c2d745ed62db9ac0a19c9dcec5d"                             
## [213] "X.Bacteria.Actinobacteria.Coriobacteriia.Coriobacteriales.Coriobacteriaceae.Atopobium.NA.5f92443932d0deb4cf0d196b6e4fe4c0"                      
## [214] "X.Bacteria.Actinobacteria.Actinobacteria.Actinomycetales.Brevibacteriaceae.Brevibacterium.aureum.2861b60feb98b96bfdfae4152ea84ae7"              
## [215] "X.Bacteria.Actinobacteria.Actinobacteria.Actinomycetales.NA.NA.NA.bb6c24bb90dd84f577f7488df6312451"                                             
## [216] "X.Bacteria.Actinobacteria.Actinobacteria.Bifidobacteriales.Bifidobacteriaceae.Bifidobacterium.NA.7b223719a0af567e7ea99f06f7ea1068"              
## [217] "X.Bacteria.Actinobacteria.Actinobacteria.Bifidobacteriales.Bifidobacteriaceae.Bifidobacterium.NA.69e611251f4d8582e312afa5737f033e"              
## [218] "X.Bacteria.Actinobacteria.Actinobacteria.Bifidobacteriales.Bifidobacteriaceae.Bifidobacterium.adolescentis.554c761996ebab999befda1b695fd81d"    
## [219] "X.Bacteria.Actinobacteria.Actinobacteria.Actinomycetales.Actinomycetaceae.Actinomyces.NA.608e6548b1b4cbb6176c8fce090991a5"                      
## [220] "X.Bacteria.Actinobacteria.Actinobacteria.Actinomycetales.Actinomycetaceae.Actinomyces.NA.a78fa572a60226d8150bfb0fc0fad652"                      
## [221] "X.Bacteria.Actinobacteria.Actinobacteria.Actinomycetales.Pseudonocardiaceae.Pseudonocardia.NA.78c07a3480fde312c999698b581e8c5f"                 
## [222] "X.Bacteria.Verrucomicrobia.Verrucomicrobiae.Verrucomicrobiales.Verrucomicrobiaceae.Akkermansia.muciniphila.8f98fb8693ed59c21399d83ce2d10724"    
## [223] "X.Bacteria.Firmicutes.Bacilli.Lactobacillales.Lactobacillaceae.Lactobacillus.zeae.5a4eda6fcdd6ab834dba0111fbe55d95"                             
## [224] "X.Bacteria.Firmicutes.Bacilli.Lactobacillales.Lactobacillaceae.Lactobacillus.zeae.45a68a9eee3cf83e27f4ea309d57ffc3"                             
## [225] "X.Bacteria.Firmicutes.Bacilli.Lactobacillales.Lactobacillaceae.Pediococcus.NA.3c133555aa2bbda4902d66bdceb138a3"                                 
## [226] "X.Bacteria.Firmicutes.Bacilli.Lactobacillales.Lactobacillaceae.Lactobacillus.salivarius.ff2bd29ff42e4dc25a31714e0b6c2dca"                       
## [227] "X.Bacteria.Firmicutes.Bacilli.Lactobacillales.Streptococcaceae.Lactococcus.NA.b39c338f5e964b6cb87e07f10badc6c4"                                 
## [228] "X.Bacteria.Firmicutes.Bacilli.Lactobacillales.Streptococcaceae.Streptococcus.NA.bd2ebc70501f7d867c204f94c4e483da"                               
## [229] "X.Bacteria.Firmicutes.Bacilli.Lactobacillales.Streptococcaceae.Streptococcus.NA.a5189f77a2cfeab3bc1602ff5c8ac3e9"                               
## [230] "X.Bacteria.Firmicutes.Bacilli.Lactobacillales.Streptococcaceae.Streptococcus.NA.5d6ee23084c6b9c96deb9a83295abc8a"                               
## [231] "X.Bacteria.Firmicutes.Bacilli.Lactobacillales.Streptococcaceae.Streptococcus.NA.73bf8d1a5983e34a0cb84e3cae127815"                               
## [232] "X.Bacteria.Firmicutes.Bacilli.Lactobacillales.Carnobacteriaceae.Granulicatella.NA.24a60c6448e70d9198ad6ba93520958c"                             
## [233] "X.Bacteria.Firmicutes.Bacilli.Lactobacillales.Aerococcaceae.Abiotrophia.NA.8114b1d0274e9e4bb6c91f6af1b8fac8"                                    
## [234] "X.Bacteria.Firmicutes.Bacilli.Gemellales.Gemellaceae.NA.NA.cda4e6f933bb3108ea3e92f9db411c00"                                                    
## [235] "X.Bacteria.Firmicutes.Clostridia.Clostridiales.Veillonellaceae.Phascolarctobacterium.NA.e5413f67faa6b8c0c3e63e48836c0b42"                       
## [236] "X.Bacteria.Firmicutes.Clostridia.Clostridiales.Veillonellaceae.Megasphaera.NA.2f93e58b78f2842e83abd5fde37ad276"                                 
## [237] "X.Bacteria.Firmicutes.Clostridia.Clostridiales.Veillonellaceae.Dialister.NA.520c77820886daeb8cf0d6497cfb1344"                                   
## [238] "X.Bacteria.Firmicutes.Clostridia.Clostridiales.Veillonellaceae.Veillonella.dispar.5608c3e6c9de9ceb79610e7786bd0ac4"                             
## [239] "X.Bacteria.Firmicutes.Clostridia.Clostridiales.Veillonellaceae.Veillonella.parvula.d3247c936f8f4735909a8526ebf2f49e"                            
## [240] "X.Bacteria.Firmicutes.Clostridia.Clostridiales.Veillonellaceae.Veillonella.dispar.5b4f8b625d8fbb1268863be7dbc4db5d"                             
## [241] "X.Bacteria.Firmicutes.Clostridia.Clostridiales.Veillonellaceae.NA.NA.983a0806857e311ab381e91c3ac211af"                                          
## [242] "X.Bacteria.Firmicutes.Clostridia.Clostridiales..Mogibacteriaceae..NA.NA.97b46cdc630fb1c3097f961bb4f627e5"                                       
## [243] "X.Bacteria.Firmicutes.Clostridia.Clostridiales..Mogibacteriaceae..NA.NA.4119ee9eb78e6baa92065d886a5e24ac"                                       
## [244] "X.Bacteria.Firmicutes.Clostridia.Clostridiales..Mogibacteriaceae..NA.NA.4260927e3b266486b5d210135f0ef4f6"                                       
## [245] "X.Bacteria.Firmicutes.Erysipelotrichi.Erysipelotrichales.Erysipelotrichaceae.NA.NA.216aca81784431f49e9567d23f7391e8"                            
## [246] "X.Bacteria.Firmicutes.Erysipelotrichi.Erysipelotrichales.Erysipelotrichaceae.NA.NA.228ae07feb0040900651f7580168bd27"                            
## [247] "X.Bacteria.Firmicutes.Erysipelotrichi.Erysipelotrichales.Erysipelotrichaceae.NA.NA.f95cab37fba4160de15015f4d520839f"                            
## [248] "X.Bacteria.Firmicutes.Erysipelotrichi.Erysipelotrichales.Erysipelotrichaceae.NA.NA.0c3887592dd60a361d43c78e501ba495"                            
## [249] "X.Bacteria.Firmicutes.Erysipelotrichi.Erysipelotrichales.Erysipelotrichaceae.Bulleidia.moorei.e083e2f58987c5f8db5d4dd16ddde91f"                 
## [250] "X.Bacteria.Firmicutes.Erysipelotrichi.Erysipelotrichales.Erysipelotrichaceae.Holdemania.NA.84e9e2afcdae1240b3ce3267067e1879"                    
## [251] "X.Bacteria.Firmicutes.Erysipelotrichi.Erysipelotrichales.Erysipelotrichaceae.Holdemania.NA.bc53814d35b2f862562a1e865e923b33"                    
## [252] "X.Bacteria.Firmicutes.Clostridia.Clostridiales.NA.NA.NA.cc4ff6e54743c750e2efa569086a38cf"                                                       
## [253] "X.Bacteria.Firmicutes.Clostridia.Clostridiales.NA.NA.NA.fd2130b6a8a1db45c0f5381012b2e5e7"                                                       
## [254] "X.Bacteria.Firmicutes.Clostridia.Clostridiales.NA.NA.NA.df15aa00cffdc5235078831e54deb6db"                                                       
## [255] "X.Bacteria.Firmicutes.Clostridia.Clostridiales.NA.NA.NA.6b40524e525db04087e414f058bc1a5e"                                                       
## [256] "X.Bacteria.Firmicutes.Clostridia.Clostridiales.NA.NA.NA.c838eb5b3b3d9fd0dfecdf75547c3c63"                                                       
## [257] "X.Bacteria.Firmicutes.Clostridia.Clostridiales.NA.NA.NA.d917386c5f307a4f652e389dadd7cef5"                                                       
## [258] "X.Bacteria.Firmicutes.Clostridia.Clostridiales.Clostridiaceae.Clostridium.NA.5c82dc47435864e490625ae01151570e"                                  
## [259] "X.Bacteria.Firmicutes.Clostridia.Clostridiales.Ruminococcaceae.NA.NA.0437c9910becc153a3d7838fd8eaa64b"                                          
## [260] "X.Bacteria.Firmicutes.Clostridia.Clostridiales.NA.NA.NA.dff390a58455e2599a43c5e400a35518"                                                       
## [261] "X.Bacteria.Firmicutes.Clostridia.Clostridiales.Ruminococcaceae.NA.NA.26f6853f46b06854fe5418317a261fb8"                                          
## [262] "X.Bacteria.Firmicutes.Clostridia.Clostridiales.Ruminococcaceae.NA.NA.6013b9e15b35c4e724eb0c323c05f9d3"                                          
## [263] "X.Bacteria.Firmicutes.Clostridia.Clostridiales.Ruminococcaceae.Oscillospira.NA.a7283edda8770d12b4e25d4dcce112c4"                                
## [264] "X.Bacteria.Firmicutes.Clostridia.Clostridiales.Ruminococcaceae.Oscillospira.NA.e13823e500387439450b3826ea191948"                                
## [265] "X.Bacteria.Firmicutes.Clostridia.Clostridiales.Ruminococcaceae.Oscillospira.NA.cb63ff338fde1171b1e985e8211b1929"                                
## [266] "X.Bacteria.Firmicutes.Clostridia.Clostridiales.Ruminococcaceae.Oscillospira.NA.d243b9b1c0b782aedbaa68fa5697193c"                                
## [267] "X.Bacteria.Firmicutes.Clostridia.Clostridiales.Ruminococcaceae.Oscillospira.NA.680d83cb233cffcc6405e08c46982042"                                
## [268] "X.Bacteria.Firmicutes.Clostridia.Clostridiales.Ruminococcaceae.Oscillospira.NA.7c93315259ad14510b6e9cae0670a048"                                
## [269] "X.Bacteria.Firmicutes.Clostridia.Clostridiales.Ruminococcaceae.NA.NA.e803ff46adaa0fa149ef151b082378a0"                                          
## [270] "X.Bacteria.Firmicutes.Clostridia.Clostridiales.Ruminococcaceae.NA.NA.63027647b049a274c881df23a4611b35"                                          
## [271] "X.Bacteria.Firmicutes.Clostridia.Clostridiales.Ruminococcaceae.NA.NA.c4995645e0c1545b0e1620144d03772b"                                          
## [272] "X.Bacteria.Firmicutes.Clostridia.Clostridiales.Ruminococcaceae.Oscillospira.NA.f2c958dfcac880c2f53a47e114d994d8"                                
## [273] "X.Bacteria.Firmicutes.Clostridia.Clostridiales.Ruminococcaceae.Oscillospira.NA.9e1d23e474a9f1e4e10387a23f601dde"                                
## [274] "X.Bacteria.Firmicutes.Clostridia.Clostridiales.Ruminococcaceae.Oscillospira.NA.4ef2b8e0ed22c2a986f53572f9e9405b"                                
## [275] "X.Bacteria.Firmicutes.Clostridia.Clostridiales.Ruminococcaceae.Oscillospira.NA.04a802674683d317a8c7484ce2407ce0"                                
## [276] "X.Bacteria.Firmicutes.Clostridia.Clostridiales.Ruminococcaceae.Oscillospira.NA.3e2bfdd1acae4723c96e344f91b262d8"                                
## [277] "X.Bacteria.Firmicutes.Clostridia.Clostridiales.Ruminococcaceae.NA.NA.2673143ffb9d8ff3ad82fa40e325973c"                                          
## [278] "X.Bacteria.Firmicutes.Clostridia.Clostridiales.Ruminococcaceae.Ruminococcus.bromii.fc02af89d869043b296114729009a5b0"                            
## [279] "X.Bacteria.Firmicutes.Clostridia.Clostridiales.Ruminococcaceae.Ruminococcus.NA.98ff1f2b1008b5e0971997cd5070fc03"                                
## [280] "X.Bacteria.Firmicutes.Clostridia.Clostridiales.Ruminococcaceae.Ruminococcus.NA.16ca411ff5a7131287f6deb7fc13b260"                                
## [281] "X.Bacteria.Firmicutes.Clostridia.Clostridiales.Ruminococcaceae.Ruminococcus.NA.153c48635771b1fdd766fc9beee8d0ab"                                
## [282] "X.Bacteria.Firmicutes.Clostridia.Clostridiales.Ruminococcaceae.Ruminococcus.NA.b61b28e1ab92f5cc8effcdde01c2ca90"                                
## [283] "X.Bacteria.Firmicutes.Clostridia.Clostridiales.Ruminococcaceae.Ruminococcus.NA.d048172b7b49bf1669197f10389312cd"                                
## [284] "X.Bacteria.Firmicutes.Clostridia.Clostridiales.Ruminococcaceae.Ruminococcus.NA.8aa94826eb4f90512d52fc7b796354c6"                                
## [285] "X.Bacteria.Firmicutes.Clostridia.Clostridiales.Ruminococcaceae.Faecalibacterium.prausnitzii.e553b9a0bb32467c71c89a4e97e55792"                   
## [286] "X.Bacteria.Firmicutes.Clostridia.Clostridiales.Ruminococcaceae.Faecalibacterium.prausnitzii.aed3f59201e3b9d21858f36557f42a80"                   
## [287] "X.Bacteria.Firmicutes.Clostridia.Clostridiales.Ruminococcaceae.Faecalibacterium.prausnitzii.a1a300c872047a7d8b6b294b17cfb63b"                   
## [288] "X.Bacteria.Firmicutes.Clostridia.Clostridiales.Ruminococcaceae.Faecalibacterium.prausnitzii.7859f0d526ad56fb4d8383d136b3bae6"                   
## [289] "X.Bacteria.Firmicutes.Clostridia.Clostridiales.Ruminococcaceae.Faecalibacterium.prausnitzii.23fed68c6c76ab10ba1be8a43e9176e7"                   
## [290] "X.Bacteria.Firmicutes.Clostridia.Clostridiales.Ruminococcaceae.Faecalibacterium.prausnitzii.c3bdda568b2c1580d5cce7407ef43909"                   
## [291] "X.Bacteria.Firmicutes.Clostridia.Clostridiales.Ruminococcaceae.Faecalibacterium.prausnitzii.a3f36ef32153f2fc2aaeac2feb23777f"                   
## [292] "X.Bacteria.Firmicutes.Clostridia.Clostridiales.Ruminococcaceae.NA.NA.c88e37c00a5abc483dada3afc5da38a3"                                          
## [293] "X.Bacteria.Firmicutes.Clostridia.Clostridiales.Ruminococcaceae.Ruminococcus.NA.98d5ae8840ae0914453cf0dd060d5f3a"                                
## [294] "X.Bacteria.Firmicutes.Clostridia.Clostridiales.Ruminococcaceae.Ruminococcus.NA.ee94b042fce1e0d75af093d7d1b1d340"                                
## [295] "X.Bacteria.Firmicutes.Clostridia.Clostridiales.Ruminococcaceae.Ruminococcus.NA.2308ced64b9c57f440230f7ddfeecda2"                                
## [296] "X.Bacteria.Firmicutes.Clostridia.Clostridiales.Ruminococcaceae.NA.NA.d4b257db9faad8674d68540e2c755d40"                                          
## [297] "X.Bacteria.Firmicutes.Clostridia.Clostridiales.Lachnospiraceae.NA.NA.bc8fb99545fba27899a16f1ade967276"                                          
## [298] "X.Bacteria.Firmicutes.Clostridia.Clostridiales.Lachnospiraceae..Ruminococcus..gnavus.90a05d597112b554e4480a8eaae4e0aa"                          
## [299] "X.Bacteria.Firmicutes.Clostridia.Clostridiales.Lachnospiraceae..Ruminococcus..gnavus.cc2d96099f530b503371e5ddca8c0a58"                          
## [300] "X.Bacteria.Firmicutes.Clostridia.Clostridiales.Lachnospiraceae.NA.NA.8b8b93b27e9c6cc58aacb0bf38248700"                                          
## [301] "X.Bacteria.Firmicutes.Clostridia.Clostridiales.Lachnospiraceae.Anaerostipes.NA.75622b8ee0f6a2b8a796bbfd264ca9fa"                                
## [302] "X.Bacteria.Firmicutes.Clostridia.Clostridiales.Lachnospiraceae.NA.NA.ac04fef6eb13ddf89756a0af35b512fe"                                          
## [303] "X.Bacteria.Firmicutes.Clostridia.Clostridiales.Lachnospiraceae.Roseburia.NA.c335a7c6e1e3f03c2c532274ec80a0c5"                                   
## [304] "X.Bacteria.Firmicutes.Clostridia.Clostridiales.Lachnospiraceae.Roseburia.NA.7d285be20e3ad3812eb21be379357ef1"                                   
## [305] "X.Bacteria.Firmicutes.Clostridia.Clostridiales.Lachnospiraceae.Roseburia.faecis.2d34c22edce4b1f2d8a5228ad78f8ea8"                               
## [306] "X.Bacteria.Firmicutes.Clostridia.Clostridiales.Lachnospiraceae.Coprococcus.NA.58c9d27620c4a8f749c75f945fce11a0"                                 
## [307] "X.Bacteria.Firmicutes.Clostridia.Clostridiales.Lachnospiraceae.Coprococcus.catus.fc2eeb02cc0e0e7f598b88912ae6c891"                              
## [308] "X.Bacteria.Firmicutes.Clostridia.Clostridiales.Lachnospiraceae.Coprococcus.eutactus.64032e2f2fa5269bd7d15eb19bdc94a9"                           
## [309] "X.Bacteria.Firmicutes.Clostridia.Clostridiales.Lachnospiraceae.Coprococcus.eutactus.53ee2a5c83806c73f3e5419690ac4241"                           
## [310] "X.Bacteria.Firmicutes.Clostridia.Clostridiales.Lachnospiraceae.Coprococcus.NA.cd287f4187715ce042afa01534b95a9b"                                 
## [311] "X.Bacteria.Firmicutes.Clostridia.Clostridiales.Lachnospiraceae.Lachnospira.NA.8e90ac2f0df6304022cff8cd278f8218"                                 
## [312] "X.Bacteria.Firmicutes.Clostridia.Clostridiales.Lachnospiraceae.Lachnospira.NA.04e9151eebd435f26965d2a465f88599"                                 
## [313] "X.Bacteria.Firmicutes.Clostridia.Clostridiales.Lachnospiraceae.Lachnospira.NA.5610b04f19aaac78ac2469a3feb707e9"                                 
## [314] "X.Bacteria.Firmicutes.Clostridia.Clostridiales.Lachnospiraceae.Lachnospira.NA.d32f24bb89dd024a38ac0a7a5962c771"                                 
## [315] "X.Bacteria.Firmicutes.Clostridia.Clostridiales.Lachnospiraceae.Lachnospira.NA.3cfa76e0da34a56a641ca33770efb588"                                 
## [316] "X.Bacteria.Firmicutes.Clostridia.Clostridiales.Lachnospiraceae.Coprococcus.NA.c9ea71f39bda8752713c8e90dff2b875"                                 
## [317] "X.Bacteria.Firmicutes.Clostridia.Clostridiales.Lachnospiraceae.Coprococcus.NA.00979c6b417b7978c1c656e263cdd054"                                 
## [318] "X.Bacteria.Firmicutes.Clostridia.Clostridiales.NA.NA.NA.504b5f0d2545c712940d4b9772a89711"                                                       
## [319] "X.Bacteria.Firmicutes.Clostridia.Clostridiales.Lachnospiraceae.NA.NA.fcd2073fc9628403d2dc4aa2b66b64a5"                                          
## [320] "X.Bacteria.Firmicutes.Clostridia.Clostridiales.Lachnospiraceae.NA.NA.fda53e1a26f7489a683cfe01228fca8f"                                          
## [321] "X.Bacteria.Firmicutes.Clostridia.Clostridiales.Lachnospiraceae.NA.NA.470dd02996e31255357eeeaa3672b469"                                          
## [322] "X.Bacteria.Firmicutes.Clostridia.Clostridiales.Lachnospiraceae.NA.NA.fb798da6858b8b3f34077164a4ec9315"                                          
## [323] "X.Bacteria.Firmicutes.Clostridia.Clostridiales.Lachnospiraceae.NA.NA.f54512749b97be9497134dc28c0af837"                                          
## [324] "X.Bacteria.Firmicutes.Clostridia.Clostridiales.Lachnospiraceae.NA.NA.51dd453b71a4aba20d9f349601ea5203"                                          
## [325] "X.Bacteria.Firmicutes.Clostridia.Clostridiales.Lachnospiraceae.NA.NA.dffa04ca171f459056b9466d7acfe601"                                          
## [326] "X.Bacteria.Firmicutes.Clostridia.Clostridiales.Lachnospiraceae.NA.NA.b20c095fd654b84cebdbfe4faa0a1409"                                          
## [327] "X.Bacteria.Firmicutes.Clostridia.Clostridiales.Lachnospiraceae.NA.NA.d5c7d97e6f4f5789d574d321dcca0992"                                          
## [328] "X.Bacteria.Firmicutes.Clostridia.Clostridiales.Lachnospiraceae..Ruminococcus..NA.00565f9981f176f2eae21aff895a72ff"                              
## [329] "X.Bacteria.Firmicutes.Clostridia.Clostridiales.Lachnospiraceae..Ruminococcus..NA.6a6fcf8f9b8bb1ab9e5f8456ee7fb109"                              
## [330] "X.Bacteria.Firmicutes.Clostridia.Clostridiales.Lachnospiraceae..Ruminococcus..torques.e59405a47acbc248ce61395366159d8d"                         
## [331] "X.Bacteria.Firmicutes.Clostridia.Clostridiales.Lachnospiraceae.NA.NA.694d61b8ec78349749c8b6ea59938e0b"                                          
## [332] "X.Bacteria.Firmicutes.Clostridia.Clostridiales.Lachnospiraceae.Blautia.obeum.ee293984c0110b2eeceb8427fdf448fb"                                  
## [333] "X.Bacteria.Firmicutes.Clostridia.Clostridiales.Lachnospiraceae.Blautia.NA.c1dc9ad5116d96b8ed863458fc0d0aec"                                     
## [334] "X.Bacteria.Firmicutes.Clostridia.Clostridiales.Lachnospiraceae.Blautia.NA.e9703768a50971c05b34b102810fd761"                                     
## [335] "X.Bacteria.Firmicutes.Clostridia.Clostridiales.Lachnospiraceae.Blautia.NA.9f8668eb1c5f9d9a992dd49245db090e"                                     
## [336] "X.Bacteria.Firmicutes.Clostridia.Clostridiales.Lachnospiraceae.Blautia.NA.7082034a5eec4c381d09cd380594242e"                                     
## [337] "X.Bacteria.Firmicutes.Clostridia.Clostridiales.Lachnospiraceae.Blautia.NA.a0a476401d529ced24788bff2ffd4515"                                     
## [338] "X.Bacteria.Firmicutes.Clostridia.Clostridiales.Lachnospiraceae.Blautia.NA.dc2721103659fe9f1d3ead56a11df243"                                     
## [339] "X.Bacteria.Firmicutes.Clostridia.Clostridiales.Lachnospiraceae.NA.NA.7e048b0e0c32d2f839e9f2098a4ebb35"                                          
## [340] "X.Bacteria.Firmicutes.Clostridia.Clostridiales.Lachnospiraceae.NA.NA.a4e94137d3eea12b54dec05caedafe47"                                          
## [341] "X.Bacteria.Firmicutes.Clostridia.Clostridiales.Lachnospiraceae.NA.NA.ebbe8017bca36f0a3b02532a2b4ed0d2"                                          
## [342] "X.Bacteria.Firmicutes.Clostridia.Clostridiales.Lachnospiraceae.NA.NA.eb61cae65bc6cdd2440323bbf603ba5c"                                          
## [343] "X.Bacteria.Firmicutes.Clostridia.Clostridiales.Lachnospiraceae.NA.NA.d9681bf8373e2635323f1ca0cc434ecf"                                          
## [344] "X.Bacteria.Firmicutes.Clostridia.Clostridiales.Lachnospiraceae.NA.NA.8a64e8e18be5e17d8ab9d76bb40f65d4"                                          
## [345] "X.Bacteria.Firmicutes.Clostridia.Clostridiales.Lachnospiraceae.NA.NA.78314aca268ae4422bc651192fbc986d"                                          
## [346] "X.Bacteria.Firmicutes.Clostridia.Clostridiales.Lachnospiraceae.NA.NA.c5465580c4c4cdd15a78de5a240dbd4e"                                          
## [347] "X.Bacteria.Firmicutes.Clostridia.Clostridiales.Lachnospiraceae.Dorea.formicigenerans.afd87e82de329a1ed75b98b5b606843c"                          
## [348] "X.Bacteria.Firmicutes.Clostridia.Clostridiales.Lachnospiraceae.Dorea.NA.a0b7d83fb64749f9a4b15b3728425b97"                                       
## [349] "X.Bacteria.Firmicutes.Clostridia.Clostridiales.Lachnospiraceae.Dorea.NA.9d21d7d00d7e938a7e1c374a4f4bb833"                                       
## [350] "X.Bacteria.Firmicutes.Clostridia.Clostridiales.Lachnospiraceae.NA.NA.752379aadaf3d2d89b4b240127b2e797"                                          
## [351] "X.Bacteria.Firmicutes.Clostridia.Clostridiales.Lachnospiraceae.NA.NA.392aa3c45e128c7432f91b3de84541f6"
```

```
meta_paired <- data_paired[,1:153]
Abund_paired <- data_paired[,154:350]

dim(Abund_paired)
```

```
## [1]  72 197
```

```
dim(meta_paired)
```

```
## [1]  72 153
```

```
Abund_paired_t <- as.data.frame(t(Abund_paired))

#Pre vs Post, full dataset

MW.p = apply(Abund_paired_t,1,
             function(x) wilcox.test(c(x)~meta_paired$SampleType, paired=TRUE)$p.value)
p.res = data.frame(Taxa=row.names(Abund_paired_t),p.val=MW.p)
# Perform multiple comparison correction using a given method of choice
p.res$rel.fdr <- p.adjust(p.res$p.val, method="fdr") #the same as benjamini-hochberg

hits.all.abund <- filter(p.res, rel.fdr<0.05)
hits.all.abund
```

```
##                                                                                                                                                                                                                                                                                    Taxa
## depth                                                                                                                                                                                                                                                                             depth
## X.Bacteria.Bacteroidetes.Bacteroidia.Bacteroidales..Barnesiellaceae..NA.NA.5b0c52a1507deba61b41b7f9d8187386                                                                 X.Bacteria.Bacteroidetes.Bacteroidia.Bacteroidales..Barnesiellaceae..NA.NA.5b0c52a1507deba61b41b7f9d8187386
## X.Bacteria.Bacteroidetes.Bacteroidia.Bacteroidales..Barnesiellaceae..NA.NA.9d0579ce6a317ca0e626a5c68981c725                                                                 X.Bacteria.Bacteroidetes.Bacteroidia.Bacteroidales..Barnesiellaceae..NA.NA.9d0579ce6a317ca0e626a5c68981c725
## X.Bacteria.Bacteroidetes.Bacteroidia.Bacteroidales..Barnesiellaceae..NA.NA.e6cd2fee507eb45ff5f3c0a4191f3876                                                                 X.Bacteria.Bacteroidetes.Bacteroidia.Bacteroidales..Barnesiellaceae..NA.NA.e6cd2fee507eb45ff5f3c0a4191f3876
## X.Bacteria.Bacteroidetes.Bacteroidia.Bacteroidales..Barnesiellaceae..NA.NA.158782cefa6e28406a2a0aeb75220f0a                                                                 X.Bacteria.Bacteroidetes.Bacteroidia.Bacteroidales..Barnesiellaceae..NA.NA.158782cefa6e28406a2a0aeb75220f0a
## X.Bacteria.Bacteroidetes.Bacteroidia.Bacteroidales.Porphyromonadaceae.Parabacteroides.NA.0d8b9177d01f328c2f5d5efa4c2acbc5                                     X.Bacteria.Bacteroidetes.Bacteroidia.Bacteroidales.Porphyromonadaceae.Parabacteroides.NA.0d8b9177d01f328c2f5d5efa4c2acbc5
## X.Bacteria.Bacteroidetes.Bacteroidia.Bacteroidales.Porphyromonadaceae.Parabacteroides.NA.e17d964cd0fd8c2ddbd1dacfa8b536e3                                     X.Bacteria.Bacteroidetes.Bacteroidia.Bacteroidales.Porphyromonadaceae.Parabacteroides.NA.e17d964cd0fd8c2ddbd1dacfa8b536e3
## X.Bacteria.Bacteroidetes.Bacteroidia.Bacteroidales.Porphyromonadaceae.Parabacteroides.distasonis.2cdb259b754c2db622ed9fb5a6517a37                     X.Bacteria.Bacteroidetes.Bacteroidia.Bacteroidales.Porphyromonadaceae.Parabacteroides.distasonis.2cdb259b754c2db622ed9fb5a6517a37
## X.Bacteria.Bacteroidetes.Bacteroidia.Bacteroidales.Porphyromonadaceae.Parabacteroides.distasonis.f4f297232da0f8d7dae7f9c432501e22                     X.Bacteria.Bacteroidetes.Bacteroidia.Bacteroidales.Porphyromonadaceae.Parabacteroides.distasonis.f4f297232da0f8d7dae7f9c432501e22
## X.Bacteria.Bacteroidetes.Bacteroidia.Bacteroidales.Porphyromonadaceae.Parabacteroides.distasonis.4844896c9c40eaca2fa0291163b9ca52                     X.Bacteria.Bacteroidetes.Bacteroidia.Bacteroidales.Porphyromonadaceae.Parabacteroides.distasonis.4844896c9c40eaca2fa0291163b9ca52
## X.Bacteria.Bacteroidetes.Bacteroidia.Bacteroidales.Bacteroidaceae.Bacteroides.ovatus.2c2018d4ecccfa3cb27b99a04c9222b1                                             X.Bacteria.Bacteroidetes.Bacteroidia.Bacteroidales.Bacteroidaceae.Bacteroides.ovatus.2c2018d4ecccfa3cb27b99a04c9222b1
## X.Bacteria.Bacteroidetes.Bacteroidia.Bacteroidales.Bacteroidaceae.Bacteroides.ovatus.03af966ff07ddef2b87da992b85b600b                                             X.Bacteria.Bacteroidetes.Bacteroidia.Bacteroidales.Bacteroidaceae.Bacteroides.ovatus.03af966ff07ddef2b87da992b85b600b
## X.Bacteria.Bacteroidetes.Bacteroidia.Bacteroidales.Bacteroidaceae.Bacteroides.NA.51e441cbdcc80da0656e82293ae160b5                                                     X.Bacteria.Bacteroidetes.Bacteroidia.Bacteroidales.Bacteroidaceae.Bacteroides.NA.51e441cbdcc80da0656e82293ae160b5
## X.Bacteria.Bacteroidetes.Bacteroidia.Bacteroidales.Bacteroidaceae.Bacteroides.fragilis.9496d87b94d90dff068f0716603930bd                                         X.Bacteria.Bacteroidetes.Bacteroidia.Bacteroidales.Bacteroidaceae.Bacteroides.fragilis.9496d87b94d90dff068f0716603930bd
## X.Bacteria.Bacteroidetes.Bacteroidia.Bacteroidales.Bacteroidaceae.Bacteroides.fragilis.35ffdd51464e2c68179717e5334a1d7e                                         X.Bacteria.Bacteroidetes.Bacteroidia.Bacteroidales.Bacteroidaceae.Bacteroides.fragilis.35ffdd51464e2c68179717e5334a1d7e
## X.Bacteria.Bacteroidetes.Bacteroidia.Bacteroidales.Bacteroidaceae.Bacteroides.caccae.ec4075339e16f5cd45fd5a7955596899                                             X.Bacteria.Bacteroidetes.Bacteroidia.Bacteroidales.Bacteroidaceae.Bacteroides.caccae.ec4075339e16f5cd45fd5a7955596899
## X.Bacteria.Bacteroidetes.Bacteroidia.Bacteroidales.Bacteroidaceae.Bacteroides.NA.25d727166a36df8d2f6a915a945bf5ac                                                     X.Bacteria.Bacteroidetes.Bacteroidia.Bacteroidales.Bacteroidaceae.Bacteroides.NA.25d727166a36df8d2f6a915a945bf5ac
## X.Bacteria.Bacteroidetes.Bacteroidia.Bacteroidales.Bacteroidaceae.Bacteroides.NA.3b872d4cecb7a9437ce9e5add011b471                                                     X.Bacteria.Bacteroidetes.Bacteroidia.Bacteroidales.Bacteroidaceae.Bacteroides.NA.3b872d4cecb7a9437ce9e5add011b471
## X.Bacteria.Bacteroidetes.Bacteroidia.Bacteroidales.Bacteroidaceae.Bacteroides.eggerthii.8ad4a56a5f526df40d97b371ba0ef33e                                       X.Bacteria.Bacteroidetes.Bacteroidia.Bacteroidales.Bacteroidaceae.Bacteroides.eggerthii.8ad4a56a5f526df40d97b371ba0ef33e
## X.Bacteria.Bacteroidetes.Bacteroidia.Bacteroidales.Bacteroidaceae.Bacteroides.eggerthii.30dd9fd45122f82c12b166a042d1eaf9                                       X.Bacteria.Bacteroidetes.Bacteroidia.Bacteroidales.Bacteroidaceae.Bacteroides.eggerthii.30dd9fd45122f82c12b166a042d1eaf9
## X.Bacteria.Bacteroidetes.Bacteroidia.Bacteroidales.Bacteroidaceae.Bacteroides.uniformis.63b26504f32377cd78d6068bffb86b9a                                       X.Bacteria.Bacteroidetes.Bacteroidia.Bacteroidales.Bacteroidaceae.Bacteroides.uniformis.63b26504f32377cd78d6068bffb86b9a
## X.Bacteria.Bacteroidetes.Bacteroidia.Bacteroidales.Bacteroidaceae.Bacteroides.uniformis.574ab9c17692ffd001643c930f6895f5                                       X.Bacteria.Bacteroidetes.Bacteroidia.Bacteroidales.Bacteroidaceae.Bacteroides.uniformis.574ab9c17692ffd001643c930f6895f5
## X.Bacteria.Bacteroidetes.Bacteroidia.Bacteroidales.Prevotellaceae.Prevotella.melaninogenica.d0b698c7298bf04110a6d2f220879bfb                               X.Bacteria.Bacteroidetes.Bacteroidia.Bacteroidales.Prevotellaceae.Prevotella.melaninogenica.d0b698c7298bf04110a6d2f220879bfb
## X.Bacteria.Bacteroidetes.Bacteroidia.Bacteroidales..Paraprevotellaceae..NA.NA.85fddfaca6687f22a2b1c5ed45a6041c                                                           X.Bacteria.Bacteroidetes.Bacteroidia.Bacteroidales..Paraprevotellaceae..NA.NA.85fddfaca6687f22a2b1c5ed45a6041c
## X.Bacteria.Bacteroidetes.Bacteroidia.Bacteroidales..Paraprevotellaceae..Paraprevotella.NA.37dac50f454a3cf754e83790eee77108                                   X.Bacteria.Bacteroidetes.Bacteroidia.Bacteroidales..Paraprevotellaceae..Paraprevotella.NA.37dac50f454a3cf754e83790eee77108
## X.Bacteria.Bacteroidetes.Bacteroidia.Bacteroidales.Bacteroidaceae.Bacteroides.NA.b65eb19257f7a2bedb5a1c4b42aeb396                                                     X.Bacteria.Bacteroidetes.Bacteroidia.Bacteroidales.Bacteroidaceae.Bacteroides.NA.b65eb19257f7a2bedb5a1c4b42aeb396
## X.Bacteria.Bacteroidetes.Bacteroidia.Bacteroidales.Bacteroidaceae.Bacteroides.NA.668fdb718997fc1589c7817655d4bb5f                                                     X.Bacteria.Bacteroidetes.Bacteroidia.Bacteroidales.Bacteroidaceae.Bacteroides.NA.668fdb718997fc1589c7817655d4bb5f
## X.Bacteria.Bacteroidetes.Bacteroidia.Bacteroidales..Odoribacteraceae..Butyricimonas.NA.0bb0e1802451aace7137a4d598f775f5                                         X.Bacteria.Bacteroidetes.Bacteroidia.Bacteroidales..Odoribacteraceae..Butyricimonas.NA.0bb0e1802451aace7137a4d598f775f5
## X.Bacteria.Bacteroidetes.Bacteroidia.Bacteroidales..Odoribacteraceae..Odoribacter.NA.3ea67969a01ec419ec1d4784d379ad1d                                             X.Bacteria.Bacteroidetes.Bacteroidia.Bacteroidales..Odoribacteraceae..Odoribacter.NA.3ea67969a01ec419ec1d4784d379ad1d
## X.Bacteria.Bacteroidetes.Bacteroidia.Bacteroidales.Rikenellaceae.NA.NA.76d4dadc006ff1d530d38dd3cfac300c                                                                         X.Bacteria.Bacteroidetes.Bacteroidia.Bacteroidales.Rikenellaceae.NA.NA.76d4dadc006ff1d530d38dd3cfac300c
## X.Bacteria.Bacteroidetes.Bacteroidia.Bacteroidales.Rikenellaceae.NA.NA.e47a63686b619f67783f9b9aa52b86bf                                                                         X.Bacteria.Bacteroidetes.Bacteroidia.Bacteroidales.Rikenellaceae.NA.NA.e47a63686b619f67783f9b9aa52b86bf
## X.Bacteria.Bacteroidetes.Bacteroidia.Bacteroidales.Rikenellaceae.NA.NA.619f64f2bf103286f4f70bfd89500ed4                                                                         X.Bacteria.Bacteroidetes.Bacteroidia.Bacteroidales.Rikenellaceae.NA.NA.619f64f2bf103286f4f70bfd89500ed4
## X.Bacteria.Bacteroidetes.Bacteroidia.Bacteroidales.Rikenellaceae.NA.NA.7534fb513a4b404419edc4e91920af3f                                                                         X.Bacteria.Bacteroidetes.Bacteroidia.Bacteroidales.Rikenellaceae.NA.NA.7534fb513a4b404419edc4e91920af3f
## X.Bacteria.Bacteroidetes.Bacteroidia.Bacteroidales.Rikenellaceae.NA.NA.a231814083060142816974207844eff2                                                                         X.Bacteria.Bacteroidetes.Bacteroidia.Bacteroidales.Rikenellaceae.NA.NA.a231814083060142816974207844eff2
## X.Bacteria.Proteobacteria.Gammaproteobacteria.Enterobacteriales.Enterobacteriaceae.NA.NA.bfe54af4c9180d37a0d76f6dafe79a5a                                     X.Bacteria.Proteobacteria.Gammaproteobacteria.Enterobacteriales.Enterobacteriaceae.NA.NA.bfe54af4c9180d37a0d76f6dafe79a5a
## X.Bacteria.Proteobacteria.Gammaproteobacteria.Enterobacteriales.Enterobacteriaceae.Escherichia.coli.1b158b8b2922d4fcad5d9cea607cbb7d               X.Bacteria.Proteobacteria.Gammaproteobacteria.Enterobacteriales.Enterobacteriaceae.Escherichia.coli.1b158b8b2922d4fcad5d9cea607cbb7d
## X.Bacteria.Proteobacteria.Gammaproteobacteria.Enterobacteriales.Enterobacteriaceae.Morganella.morganii.78056058faedd75d706633c5f55a975f         X.Bacteria.Proteobacteria.Gammaproteobacteria.Enterobacteriales.Enterobacteriaceae.Morganella.morganii.78056058faedd75d706633c5f55a975f
## X.Bacteria.Proteobacteria.Gammaproteobacteria.Xanthomonadales.Xanthomonadaceae.Stenotrophomonas.NA.891019e319f5d8d088ed4f9379fd63ef                 X.Bacteria.Proteobacteria.Gammaproteobacteria.Xanthomonadales.Xanthomonadaceae.Stenotrophomonas.NA.891019e319f5d8d088ed4f9379fd63ef
## X.Bacteria.Proteobacteria.Deltaproteobacteria.Desulfovibrionales.Desulfovibrionaceae.Bilophila.NA.ece50a62168f85fc61385d8adb4c6494                   X.Bacteria.Proteobacteria.Deltaproteobacteria.Desulfovibrionales.Desulfovibrionaceae.Bilophila.NA.ece50a62168f85fc61385d8adb4c6494
## X.Bacteria.Proteobacteria.Deltaproteobacteria.Desulfovibrionales.Desulfovibrionaceae.Desulfovibrio.NA.dfd770d48651635c39bdafe13f62e75a           X.Bacteria.Proteobacteria.Deltaproteobacteria.Desulfovibrionales.Desulfovibrionaceae.Desulfovibrio.NA.dfd770d48651635c39bdafe13f62e75a
## X.Bacteria.Actinobacteria.Coriobacteriia.Coriobacteriales.Coriobacteriaceae.Collinsella.aerofaciens.dfba68ef0fd0e712608eb2a0078013a7               X.Bacteria.Actinobacteria.Coriobacteriia.Coriobacteriales.Coriobacteriaceae.Collinsella.aerofaciens.dfba68ef0fd0e712608eb2a0078013a7
## X.Bacteria.Actinobacteria.Coriobacteriia.Coriobacteriales.Coriobacteriaceae.Slackia.NA.c9a59a2ded38b324d6d58202a4137f9b                                         X.Bacteria.Actinobacteria.Coriobacteriia.Coriobacteriales.Coriobacteriaceae.Slackia.NA.c9a59a2ded38b324d6d58202a4137f9b
## X.Bacteria.Actinobacteria.Coriobacteriia.Coriobacteriales.Coriobacteriaceae.Eggerthella.lenta.6bb6aad559c5eaeb22b2eef7874ef038                           X.Bacteria.Actinobacteria.Coriobacteriia.Coriobacteriales.Coriobacteriaceae.Eggerthella.lenta.6bb6aad559c5eaeb22b2eef7874ef038
## X.Bacteria.Actinobacteria.Coriobacteriia.Coriobacteriales.Coriobacteriaceae.NA.NA.23368c2d745ed62db9ac0a19c9dcec5d                                                   X.Bacteria.Actinobacteria.Coriobacteriia.Coriobacteriales.Coriobacteriaceae.NA.NA.23368c2d745ed62db9ac0a19c9dcec5d
## X.Bacteria.Actinobacteria.Actinobacteria.Bifidobacteriales.Bifidobacteriaceae.Bifidobacterium.NA.69e611251f4d8582e312afa5737f033e                     X.Bacteria.Actinobacteria.Actinobacteria.Bifidobacteriales.Bifidobacteriaceae.Bifidobacterium.NA.69e611251f4d8582e312afa5737f033e
## X.Bacteria.Actinobacteria.Actinobacteria.Bifidobacteriales.Bifidobacteriaceae.Bifidobacterium.adolescentis.554c761996ebab999befda1b695fd81d X.Bacteria.Actinobacteria.Actinobacteria.Bifidobacteriales.Bifidobacteriaceae.Bifidobacterium.adolescentis.554c761996ebab999befda1b695fd81d
## X.Bacteria.Actinobacteria.Actinobacteria.Actinomycetales.Actinomycetaceae.Actinomyces.NA.608e6548b1b4cbb6176c8fce090991a5                                     X.Bacteria.Actinobacteria.Actinobacteria.Actinomycetales.Actinomycetaceae.Actinomyces.NA.608e6548b1b4cbb6176c8fce090991a5
## X.Bacteria.Actinobacteria.Actinobacteria.Actinomycetales.Actinomycetaceae.Actinomyces.NA.a78fa572a60226d8150bfb0fc0fad652                                     X.Bacteria.Actinobacteria.Actinobacteria.Actinomycetales.Actinomycetaceae.Actinomyces.NA.a78fa572a60226d8150bfb0fc0fad652
## X.Bacteria.Verrucomicrobia.Verrucomicrobiae.Verrucomicrobiales.Verrucomicrobiaceae.Akkermansia.muciniphila.8f98fb8693ed59c21399d83ce2d10724 X.Bacteria.Verrucomicrobia.Verrucomicrobiae.Verrucomicrobiales.Verrucomicrobiaceae.Akkermansia.muciniphila.8f98fb8693ed59c21399d83ce2d10724
## X.Bacteria.Firmicutes.Bacilli.Lactobacillales.Lactobacillaceae.Lactobacillus.zeae.5a4eda6fcdd6ab834dba0111fbe55d95                                                   X.Bacteria.Firmicutes.Bacilli.Lactobacillales.Lactobacillaceae.Lactobacillus.zeae.5a4eda6fcdd6ab834dba0111fbe55d95
## X.Bacteria.Firmicutes.Bacilli.Lactobacillales.Lactobacillaceae.Lactobacillus.zeae.45a68a9eee3cf83e27f4ea309d57ffc3                                                   X.Bacteria.Firmicutes.Bacilli.Lactobacillales.Lactobacillaceae.Lactobacillus.zeae.45a68a9eee3cf83e27f4ea309d57ffc3
## X.Bacteria.Firmicutes.Bacilli.Lactobacillales.Lactobacillaceae.Pediococcus.NA.3c133555aa2bbda4902d66bdceb138a3                                                           X.Bacteria.Firmicutes.Bacilli.Lactobacillales.Lactobacillaceae.Pediococcus.NA.3c133555aa2bbda4902d66bdceb138a3
## X.Bacteria.Firmicutes.Bacilli.Lactobacillales.Lactobacillaceae.Lactobacillus.salivarius.ff2bd29ff42e4dc25a31714e0b6c2dca                                       X.Bacteria.Firmicutes.Bacilli.Lactobacillales.Lactobacillaceae.Lactobacillus.salivarius.ff2bd29ff42e4dc25a31714e0b6c2dca
## X.Bacteria.Firmicutes.Bacilli.Lactobacillales.Streptococcaceae.Streptococcus.NA.bd2ebc70501f7d867c204f94c4e483da                                                       X.Bacteria.Firmicutes.Bacilli.Lactobacillales.Streptococcaceae.Streptococcus.NA.bd2ebc70501f7d867c204f94c4e483da
## X.Bacteria.Firmicutes.Bacilli.Lactobacillales.Streptococcaceae.Streptococcus.NA.73bf8d1a5983e34a0cb84e3cae127815                                                       X.Bacteria.Firmicutes.Bacilli.Lactobacillales.Streptococcaceae.Streptococcus.NA.73bf8d1a5983e34a0cb84e3cae127815
## X.Bacteria.Firmicutes.Bacilli.Lactobacillales.Carnobacteriaceae.Granulicatella.NA.24a60c6448e70d9198ad6ba93520958c                                                   X.Bacteria.Firmicutes.Bacilli.Lactobacillales.Carnobacteriaceae.Granulicatella.NA.24a60c6448e70d9198ad6ba93520958c
## X.Bacteria.Firmicutes.Bacilli.Lactobacillales.Aerococcaceae.Abiotrophia.NA.8114b1d0274e9e4bb6c91f6af1b8fac8                                                                 X.Bacteria.Firmicutes.Bacilli.Lactobacillales.Aerococcaceae.Abiotrophia.NA.8114b1d0274e9e4bb6c91f6af1b8fac8
## X.Bacteria.Firmicutes.Bacilli.Gemellales.Gemellaceae.NA.NA.cda4e6f933bb3108ea3e92f9db411c00                                                                                                 X.Bacteria.Firmicutes.Bacilli.Gemellales.Gemellaceae.NA.NA.cda4e6f933bb3108ea3e92f9db411c00
## X.Bacteria.Firmicutes.Clostridia.Clostridiales.Veillonellaceae.Phascolarctobacterium.NA.e5413f67faa6b8c0c3e63e48836c0b42                                       X.Bacteria.Firmicutes.Clostridia.Clostridiales.Veillonellaceae.Phascolarctobacterium.NA.e5413f67faa6b8c0c3e63e48836c0b42
## X.Bacteria.Firmicutes.Clostridia.Clostridiales.Veillonellaceae.Megasphaera.NA.2f93e58b78f2842e83abd5fde37ad276                                                           X.Bacteria.Firmicutes.Clostridia.Clostridiales.Veillonellaceae.Megasphaera.NA.2f93e58b78f2842e83abd5fde37ad276
## X.Bacteria.Firmicutes.Clostridia.Clostridiales.Veillonellaceae.Dialister.NA.520c77820886daeb8cf0d6497cfb1344                                                               X.Bacteria.Firmicutes.Clostridia.Clostridiales.Veillonellaceae.Dialister.NA.520c77820886daeb8cf0d6497cfb1344
## X.Bacteria.Firmicutes.Clostridia.Clostridiales.Veillonellaceae.Veillonella.dispar.5608c3e6c9de9ceb79610e7786bd0ac4                                                   X.Bacteria.Firmicutes.Clostridia.Clostridiales.Veillonellaceae.Veillonella.dispar.5608c3e6c9de9ceb79610e7786bd0ac4
## X.Bacteria.Firmicutes.Clostridia.Clostridiales.Veillonellaceae.Veillonella.dispar.5b4f8b625d8fbb1268863be7dbc4db5d                                                   X.Bacteria.Firmicutes.Clostridia.Clostridiales.Veillonellaceae.Veillonella.dispar.5b4f8b625d8fbb1268863be7dbc4db5d
## X.Bacteria.Firmicutes.Clostridia.Clostridiales..Mogibacteriaceae..NA.NA.97b46cdc630fb1c3097f961bb4f627e5                                                                       X.Bacteria.Firmicutes.Clostridia.Clostridiales..Mogibacteriaceae..NA.NA.97b46cdc630fb1c3097f961bb4f627e5
## X.Bacteria.Firmicutes.Clostridia.Clostridiales..Mogibacteriaceae..NA.NA.4119ee9eb78e6baa92065d886a5e24ac                                                                       X.Bacteria.Firmicutes.Clostridia.Clostridiales..Mogibacteriaceae..NA.NA.4119ee9eb78e6baa92065d886a5e24ac
## X.Bacteria.Firmicutes.Clostridia.Clostridiales..Mogibacteriaceae..NA.NA.4260927e3b266486b5d210135f0ef4f6                                                                       X.Bacteria.Firmicutes.Clostridia.Clostridiales..Mogibacteriaceae..NA.NA.4260927e3b266486b5d210135f0ef4f6
## X.Bacteria.Firmicutes.Erysipelotrichi.Erysipelotrichales.Erysipelotrichaceae.NA.NA.216aca81784431f49e9567d23f7391e8                                                 X.Bacteria.Firmicutes.Erysipelotrichi.Erysipelotrichales.Erysipelotrichaceae.NA.NA.216aca81784431f49e9567d23f7391e8
## X.Bacteria.Firmicutes.Erysipelotrichi.Erysipelotrichales.Erysipelotrichaceae.NA.NA.f95cab37fba4160de15015f4d520839f                                                 X.Bacteria.Firmicutes.Erysipelotrichi.Erysipelotrichales.Erysipelotrichaceae.NA.NA.f95cab37fba4160de15015f4d520839f
## X.Bacteria.Firmicutes.Erysipelotrichi.Erysipelotrichales.Erysipelotrichaceae.NA.NA.0c3887592dd60a361d43c78e501ba495                                                 X.Bacteria.Firmicutes.Erysipelotrichi.Erysipelotrichales.Erysipelotrichaceae.NA.NA.0c3887592dd60a361d43c78e501ba495
## X.Bacteria.Firmicutes.Erysipelotrichi.Erysipelotrichales.Erysipelotrichaceae.Holdemania.NA.84e9e2afcdae1240b3ce3267067e1879                                 X.Bacteria.Firmicutes.Erysipelotrichi.Erysipelotrichales.Erysipelotrichaceae.Holdemania.NA.84e9e2afcdae1240b3ce3267067e1879
## X.Bacteria.Firmicutes.Erysipelotrichi.Erysipelotrichales.Erysipelotrichaceae.Holdemania.NA.bc53814d35b2f862562a1e865e923b33                                 X.Bacteria.Firmicutes.Erysipelotrichi.Erysipelotrichales.Erysipelotrichaceae.Holdemania.NA.bc53814d35b2f862562a1e865e923b33
## X.Bacteria.Firmicutes.Clostridia.Clostridiales.NA.NA.NA.cc4ff6e54743c750e2efa569086a38cf                                                                                                       X.Bacteria.Firmicutes.Clostridia.Clostridiales.NA.NA.NA.cc4ff6e54743c750e2efa569086a38cf
## X.Bacteria.Firmicutes.Clostridia.Clostridiales.NA.NA.NA.fd2130b6a8a1db45c0f5381012b2e5e7                                                                                                       X.Bacteria.Firmicutes.Clostridia.Clostridiales.NA.NA.NA.fd2130b6a8a1db45c0f5381012b2e5e7
## X.Bacteria.Firmicutes.Clostridia.Clostridiales.NA.NA.NA.df15aa00cffdc5235078831e54deb6db                                                                                                       X.Bacteria.Firmicutes.Clostridia.Clostridiales.NA.NA.NA.df15aa00cffdc5235078831e54deb6db
## X.Bacteria.Firmicutes.Clostridia.Clostridiales.NA.NA.NA.6b40524e525db04087e414f058bc1a5e                                                                                                       X.Bacteria.Firmicutes.Clostridia.Clostridiales.NA.NA.NA.6b40524e525db04087e414f058bc1a5e
## X.Bacteria.Firmicutes.Clostridia.Clostridiales.NA.NA.NA.c838eb5b3b3d9fd0dfecdf75547c3c63                                                                                                       X.Bacteria.Firmicutes.Clostridia.Clostridiales.NA.NA.NA.c838eb5b3b3d9fd0dfecdf75547c3c63
## X.Bacteria.Firmicutes.Clostridia.Clostridiales.NA.NA.NA.d917386c5f307a4f652e389dadd7cef5                                                                                                       X.Bacteria.Firmicutes.Clostridia.Clostridiales.NA.NA.NA.d917386c5f307a4f652e389dadd7cef5
## X.Bacteria.Firmicutes.Clostridia.Clostridiales.Clostridiaceae.Clostridium.NA.5c82dc47435864e490625ae01151570e                                                             X.Bacteria.Firmicutes.Clostridia.Clostridiales.Clostridiaceae.Clostridium.NA.5c82dc47435864e490625ae01151570e
## X.Bacteria.Firmicutes.Clostridia.Clostridiales.Ruminococcaceae.NA.NA.0437c9910becc153a3d7838fd8eaa64b                                                                             X.Bacteria.Firmicutes.Clostridia.Clostridiales.Ruminococcaceae.NA.NA.0437c9910becc153a3d7838fd8eaa64b
## X.Bacteria.Firmicutes.Clostridia.Clostridiales.NA.NA.NA.dff390a58455e2599a43c5e400a35518                                                                                                       X.Bacteria.Firmicutes.Clostridia.Clostridiales.NA.NA.NA.dff390a58455e2599a43c5e400a35518
## X.Bacteria.Firmicutes.Clostridia.Clostridiales.Ruminococcaceae.NA.NA.26f6853f46b06854fe5418317a261fb8                                                                             X.Bacteria.Firmicutes.Clostridia.Clostridiales.Ruminococcaceae.NA.NA.26f6853f46b06854fe5418317a261fb8
## X.Bacteria.Firmicutes.Clostridia.Clostridiales.Ruminococcaceae.NA.NA.6013b9e15b35c4e724eb0c323c05f9d3                                                                             X.Bacteria.Firmicutes.Clostridia.Clostridiales.Ruminococcaceae.NA.NA.6013b9e15b35c4e724eb0c323c05f9d3
## X.Bacteria.Firmicutes.Clostridia.Clostridiales.Ruminococcaceae.Oscillospira.NA.a7283edda8770d12b4e25d4dcce112c4                                                         X.Bacteria.Firmicutes.Clostridia.Clostridiales.Ruminococcaceae.Oscillospira.NA.a7283edda8770d12b4e25d4dcce112c4
## X.Bacteria.Firmicutes.Clostridia.Clostridiales.Ruminococcaceae.Oscillospira.NA.e13823e500387439450b3826ea191948                                                         X.Bacteria.Firmicutes.Clostridia.Clostridiales.Ruminococcaceae.Oscillospira.NA.e13823e500387439450b3826ea191948
## X.Bacteria.Firmicutes.Clostridia.Clostridiales.Ruminococcaceae.Oscillospira.NA.cb63ff338fde1171b1e985e8211b1929                                                         X.Bacteria.Firmicutes.Clostridia.Clostridiales.Ruminococcaceae.Oscillospira.NA.cb63ff338fde1171b1e985e8211b1929
## X.Bacteria.Firmicutes.Clostridia.Clostridiales.Ruminococcaceae.Oscillospira.NA.d243b9b1c0b782aedbaa68fa5697193c                                                         X.Bacteria.Firmicutes.Clostridia.Clostridiales.Ruminococcaceae.Oscillospira.NA.d243b9b1c0b782aedbaa68fa5697193c
## X.Bacteria.Firmicutes.Clostridia.Clostridiales.Ruminococcaceae.Oscillospira.NA.680d83cb233cffcc6405e08c46982042                                                         X.Bacteria.Firmicutes.Clostridia.Clostridiales.Ruminococcaceae.Oscillospira.NA.680d83cb233cffcc6405e08c46982042
## X.Bacteria.Firmicutes.Clostridia.Clostridiales.Ruminococcaceae.Oscillospira.NA.7c93315259ad14510b6e9cae0670a048                                                         X.Bacteria.Firmicutes.Clostridia.Clostridiales.Ruminococcaceae.Oscillospira.NA.7c93315259ad14510b6e9cae0670a048
## X.Bacteria.Firmicutes.Clostridia.Clostridiales.Ruminococcaceae.NA.NA.e803ff46adaa0fa149ef151b082378a0                                                                             X.Bacteria.Firmicutes.Clostridia.Clostridiales.Ruminococcaceae.NA.NA.e803ff46adaa0fa149ef151b082378a0
## X.Bacteria.Firmicutes.Clostridia.Clostridiales.Ruminococcaceae.NA.NA.63027647b049a274c881df23a4611b35                                                                             X.Bacteria.Firmicutes.Clostridia.Clostridiales.Ruminococcaceae.NA.NA.63027647b049a274c881df23a4611b35
## X.Bacteria.Firmicutes.Clostridia.Clostridiales.Ruminococcaceae.NA.NA.c4995645e0c1545b0e1620144d03772b                                                                             X.Bacteria.Firmicutes.Clostridia.Clostridiales.Ruminococcaceae.NA.NA.c4995645e0c1545b0e1620144d03772b
## X.Bacteria.Firmicutes.Clostridia.Clostridiales.Ruminococcaceae.Oscillospira.NA.f2c958dfcac880c2f53a47e114d994d8                                                         X.Bacteria.Firmicutes.Clostridia.Clostridiales.Ruminococcaceae.Oscillospira.NA.f2c958dfcac880c2f53a47e114d994d8
## X.Bacteria.Firmicutes.Clostridia.Clostridiales.Ruminococcaceae.Oscillospira.NA.9e1d23e474a9f1e4e10387a23f601dde                                                         X.Bacteria.Firmicutes.Clostridia.Clostridiales.Ruminococcaceae.Oscillospira.NA.9e1d23e474a9f1e4e10387a23f601dde
## X.Bacteria.Firmicutes.Clostridia.Clostridiales.Ruminococcaceae.Oscillospira.NA.4ef2b8e0ed22c2a986f53572f9e9405b                                                         X.Bacteria.Firmicutes.Clostridia.Clostridiales.Ruminococcaceae.Oscillospira.NA.4ef2b8e0ed22c2a986f53572f9e9405b
## X.Bacteria.Firmicutes.Clostridia.Clostridiales.Ruminococcaceae.Oscillospira.NA.04a802674683d317a8c7484ce2407ce0                                                         X.Bacteria.Firmicutes.Clostridia.Clostridiales.Ruminococcaceae.Oscillospira.NA.04a802674683d317a8c7484ce2407ce0
## X.Bacteria.Firmicutes.Clostridia.Clostridiales.Ruminococcaceae.Oscillospira.NA.3e2bfdd1acae4723c96e344f91b262d8                                                         X.Bacteria.Firmicutes.Clostridia.Clostridiales.Ruminococcaceae.Oscillospira.NA.3e2bfdd1acae4723c96e344f91b262d8
## X.Bacteria.Firmicutes.Clostridia.Clostridiales.Ruminococcaceae.NA.NA.2673143ffb9d8ff3ad82fa40e325973c                                                                             X.Bacteria.Firmicutes.Clostridia.Clostridiales.Ruminococcaceae.NA.NA.2673143ffb9d8ff3ad82fa40e325973c
## X.Bacteria.Firmicutes.Clostridia.Clostridiales.Ruminococcaceae.Ruminococcus.bromii.fc02af89d869043b296114729009a5b0                                                 X.Bacteria.Firmicutes.Clostridia.Clostridiales.Ruminococcaceae.Ruminococcus.bromii.fc02af89d869043b296114729009a5b0
## X.Bacteria.Firmicutes.Clostridia.Clostridiales.Ruminococcaceae.Ruminococcus.NA.98ff1f2b1008b5e0971997cd5070fc03                                                         X.Bacteria.Firmicutes.Clostridia.Clostridiales.Ruminococcaceae.Ruminococcus.NA.98ff1f2b1008b5e0971997cd5070fc03
## X.Bacteria.Firmicutes.Clostridia.Clostridiales.Ruminococcaceae.Ruminococcus.NA.16ca411ff5a7131287f6deb7fc13b260                                                         X.Bacteria.Firmicutes.Clostridia.Clostridiales.Ruminococcaceae.Ruminococcus.NA.16ca411ff5a7131287f6deb7fc13b260
## X.Bacteria.Firmicutes.Clostridia.Clostridiales.Ruminococcaceae.Ruminococcus.NA.153c48635771b1fdd766fc9beee8d0ab                                                         X.Bacteria.Firmicutes.Clostridia.Clostridiales.Ruminococcaceae.Ruminococcus.NA.153c48635771b1fdd766fc9beee8d0ab
## X.Bacteria.Firmicutes.Clostridia.Clostridiales.Ruminococcaceae.Ruminococcus.NA.b61b28e1ab92f5cc8effcdde01c2ca90                                                         X.Bacteria.Firmicutes.Clostridia.Clostridiales.Ruminococcaceae.Ruminococcus.NA.b61b28e1ab92f5cc8effcdde01c2ca90
## X.Bacteria.Firmicutes.Clostridia.Clostridiales.Ruminococcaceae.Ruminococcus.NA.d048172b7b49bf1669197f10389312cd                                                         X.Bacteria.Firmicutes.Clostridia.Clostridiales.Ruminococcaceae.Ruminococcus.NA.d048172b7b49bf1669197f10389312cd
## X.Bacteria.Firmicutes.Clostridia.Clostridiales.Ruminococcaceae.Ruminococcus.NA.8aa94826eb4f90512d52fc7b796354c6                                                         X.Bacteria.Firmicutes.Clostridia.Clostridiales.Ruminococcaceae.Ruminococcus.NA.8aa94826eb4f90512d52fc7b796354c6
## X.Bacteria.Firmicutes.Clostridia.Clostridiales.Ruminococcaceae.Faecalibacterium.prausnitzii.e553b9a0bb32467c71c89a4e97e55792                               X.Bacteria.Firmicutes.Clostridia.Clostridiales.Ruminococcaceae.Faecalibacterium.prausnitzii.e553b9a0bb32467c71c89a4e97e55792
## X.Bacteria.Firmicutes.Clostridia.Clostridiales.Ruminococcaceae.Faecalibacterium.prausnitzii.aed3f59201e3b9d21858f36557f42a80                               X.Bacteria.Firmicutes.Clostridia.Clostridiales.Ruminococcaceae.Faecalibacterium.prausnitzii.aed3f59201e3b9d21858f36557f42a80
## X.Bacteria.Firmicutes.Clostridia.Clostridiales.Ruminococcaceae.Faecalibacterium.prausnitzii.a1a300c872047a7d8b6b294b17cfb63b                               X.Bacteria.Firmicutes.Clostridia.Clostridiales.Ruminococcaceae.Faecalibacterium.prausnitzii.a1a300c872047a7d8b6b294b17cfb63b
## X.Bacteria.Firmicutes.Clostridia.Clostridiales.Ruminococcaceae.Faecalibacterium.prausnitzii.7859f0d526ad56fb4d8383d136b3bae6                               X.Bacteria.Firmicutes.Clostridia.Clostridiales.Ruminococcaceae.Faecalibacterium.prausnitzii.7859f0d526ad56fb4d8383d136b3bae6
## X.Bacteria.Firmicutes.Clostridia.Clostridiales.Ruminococcaceae.Faecalibacterium.prausnitzii.23fed68c6c76ab10ba1be8a43e9176e7                               X.Bacteria.Firmicutes.Clostridia.Clostridiales.Ruminococcaceae.Faecalibacterium.prausnitzii.23fed68c6c76ab10ba1be8a43e9176e7
## X.Bacteria.Firmicutes.Clostridia.Clostridiales.Ruminococcaceae.Faecalibacterium.prausnitzii.c3bdda568b2c1580d5cce7407ef43909                               X.Bacteria.Firmicutes.Clostridia.Clostridiales.Ruminococcaceae.Faecalibacterium.prausnitzii.c3bdda568b2c1580d5cce7407ef43909
## X.Bacteria.Firmicutes.Clostridia.Clostridiales.Ruminococcaceae.Faecalibacterium.prausnitzii.a3f36ef32153f2fc2aaeac2feb23777f                               X.Bacteria.Firmicutes.Clostridia.Clostridiales.Ruminococcaceae.Faecalibacterium.prausnitzii.a3f36ef32153f2fc2aaeac2feb23777f
## X.Bacteria.Firmicutes.Clostridia.Clostridiales.Ruminococcaceae.NA.NA.c88e37c00a5abc483dada3afc5da38a3                                                                             X.Bacteria.Firmicutes.Clostridia.Clostridiales.Ruminococcaceae.NA.NA.c88e37c00a5abc483dada3afc5da38a3
## X.Bacteria.Firmicutes.Clostridia.Clostridiales.Ruminococcaceae.Ruminococcus.NA.98d5ae8840ae0914453cf0dd060d5f3a                                                         X.Bacteria.Firmicutes.Clostridia.Clostridiales.Ruminococcaceae.Ruminococcus.NA.98d5ae8840ae0914453cf0dd060d5f3a
## X.Bacteria.Firmicutes.Clostridia.Clostridiales.Ruminococcaceae.Ruminococcus.NA.ee94b042fce1e0d75af093d7d1b1d340                                                         X.Bacteria.Firmicutes.Clostridia.Clostridiales.Ruminococcaceae.Ruminococcus.NA.ee94b042fce1e0d75af093d7d1b1d340
## X.Bacteria.Firmicutes.Clostridia.Clostridiales.Ruminococcaceae.Ruminococcus.NA.2308ced64b9c57f440230f7ddfeecda2                                                         X.Bacteria.Firmicutes.Clostridia.Clostridiales.Ruminococcaceae.Ruminococcus.NA.2308ced64b9c57f440230f7ddfeecda2
## X.Bacteria.Firmicutes.Clostridia.Clostridiales.Ruminococcaceae.NA.NA.d4b257db9faad8674d68540e2c755d40                                                                             X.Bacteria.Firmicutes.Clostridia.Clostridiales.Ruminococcaceae.NA.NA.d4b257db9faad8674d68540e2c755d40
## X.Bacteria.Firmicutes.Clostridia.Clostridiales.Lachnospiraceae.NA.NA.bc8fb99545fba27899a16f1ade967276                                                                             X.Bacteria.Firmicutes.Clostridia.Clostridiales.Lachnospiraceae.NA.NA.bc8fb99545fba27899a16f1ade967276
## X.Bacteria.Firmicutes.Clostridia.Clostridiales.Lachnospiraceae..Ruminococcus..gnavus.90a05d597112b554e4480a8eaae4e0aa                                             X.Bacteria.Firmicutes.Clostridia.Clostridiales.Lachnospiraceae..Ruminococcus..gnavus.90a05d597112b554e4480a8eaae4e0aa
## X.Bacteria.Firmicutes.Clostridia.Clostridiales.Lachnospiraceae..Ruminococcus..gnavus.cc2d96099f530b503371e5ddca8c0a58                                             X.Bacteria.Firmicutes.Clostridia.Clostridiales.Lachnospiraceae..Ruminococcus..gnavus.cc2d96099f530b503371e5ddca8c0a58
## X.Bacteria.Firmicutes.Clostridia.Clostridiales.Lachnospiraceae.NA.NA.8b8b93b27e9c6cc58aacb0bf38248700                                                                             X.Bacteria.Firmicutes.Clostridia.Clostridiales.Lachnospiraceae.NA.NA.8b8b93b27e9c6cc58aacb0bf38248700
## X.Bacteria.Firmicutes.Clostridia.Clostridiales.Lachnospiraceae.Anaerostipes.NA.75622b8ee0f6a2b8a796bbfd264ca9fa                                                         X.Bacteria.Firmicutes.Clostridia.Clostridiales.Lachnospiraceae.Anaerostipes.NA.75622b8ee0f6a2b8a796bbfd264ca9fa
## X.Bacteria.Firmicutes.Clostridia.Clostridiales.Lachnospiraceae.NA.NA.ac04fef6eb13ddf89756a0af35b512fe                                                                             X.Bacteria.Firmicutes.Clostridia.Clostridiales.Lachnospiraceae.NA.NA.ac04fef6eb13ddf89756a0af35b512fe
## X.Bacteria.Firmicutes.Clostridia.Clostridiales.Lachnospiraceae.Roseburia.NA.c335a7c6e1e3f03c2c532274ec80a0c5                                                               X.Bacteria.Firmicutes.Clostridia.Clostridiales.Lachnospiraceae.Roseburia.NA.c335a7c6e1e3f03c2c532274ec80a0c5
## X.Bacteria.Firmicutes.Clostridia.Clostridiales.Lachnospiraceae.Roseburia.NA.7d285be20e3ad3812eb21be379357ef1                                                               X.Bacteria.Firmicutes.Clostridia.Clostridiales.Lachnospiraceae.Roseburia.NA.7d285be20e3ad3812eb21be379357ef1
## X.Bacteria.Firmicutes.Clostridia.Clostridiales.Lachnospiraceae.Roseburia.faecis.2d34c22edce4b1f2d8a5228ad78f8ea8                                                       X.Bacteria.Firmicutes.Clostridia.Clostridiales.Lachnospiraceae.Roseburia.faecis.2d34c22edce4b1f2d8a5228ad78f8ea8
## X.Bacteria.Firmicutes.Clostridia.Clostridiales.Lachnospiraceae.Coprococcus.NA.58c9d27620c4a8f749c75f945fce11a0                                                           X.Bacteria.Firmicutes.Clostridia.Clostridiales.Lachnospiraceae.Coprococcus.NA.58c9d27620c4a8f749c75f945fce11a0
## X.Bacteria.Firmicutes.Clostridia.Clostridiales.Lachnospiraceae.Coprococcus.catus.fc2eeb02cc0e0e7f598b88912ae6c891                                                     X.Bacteria.Firmicutes.Clostridia.Clostridiales.Lachnospiraceae.Coprococcus.catus.fc2eeb02cc0e0e7f598b88912ae6c891
## X.Bacteria.Firmicutes.Clostridia.Clostridiales.Lachnospiraceae.Coprococcus.eutactus.64032e2f2fa5269bd7d15eb19bdc94a9                                               X.Bacteria.Firmicutes.Clostridia.Clostridiales.Lachnospiraceae.Coprococcus.eutactus.64032e2f2fa5269bd7d15eb19bdc94a9
## X.Bacteria.Firmicutes.Clostridia.Clostridiales.Lachnospiraceae.Coprococcus.eutactus.53ee2a5c83806c73f3e5419690ac4241                                               X.Bacteria.Firmicutes.Clostridia.Clostridiales.Lachnospiraceae.Coprococcus.eutactus.53ee2a5c83806c73f3e5419690ac4241
## X.Bacteria.Firmicutes.Clostridia.Clostridiales.Lachnospiraceae.Coprococcus.NA.cd287f4187715ce042afa01534b95a9b                                                           X.Bacteria.Firmicutes.Clostridia.Clostridiales.Lachnospiraceae.Coprococcus.NA.cd287f4187715ce042afa01534b95a9b
## X.Bacteria.Firmicutes.Clostridia.Clostridiales.Lachnospiraceae.Lachnospira.NA.8e90ac2f0df6304022cff8cd278f8218                                                           X.Bacteria.Firmicutes.Clostridia.Clostridiales.Lachnospiraceae.Lachnospira.NA.8e90ac2f0df6304022cff8cd278f8218
## X.Bacteria.Firmicutes.Clostridia.Clostridiales.Lachnospiraceae.Lachnospira.NA.04e9151eebd435f26965d2a465f88599                                                           X.Bacteria.Firmicutes.Clostridia.Clostridiales.Lachnospiraceae.Lachnospira.NA.04e9151eebd435f26965d2a465f88599
## X.Bacteria.Firmicutes.Clostridia.Clostridiales.Lachnospiraceae.Lachnospira.NA.5610b04f19aaac78ac2469a3feb707e9                                                           X.Bacteria.Firmicutes.Clostridia.Clostridiales.Lachnospiraceae.Lachnospira.NA.5610b04f19aaac78ac2469a3feb707e9
## X.Bacteria.Firmicutes.Clostridia.Clostridiales.Lachnospiraceae.Lachnospira.NA.d32f24bb89dd024a38ac0a7a5962c771                                                           X.Bacteria.Firmicutes.Clostridia.Clostridiales.Lachnospiraceae.Lachnospira.NA.d32f24bb89dd024a38ac0a7a5962c771
## X.Bacteria.Firmicutes.Clostridia.Clostridiales.Lachnospiraceae.Lachnospira.NA.3cfa76e0da34a56a641ca33770efb588                                                           X.Bacteria.Firmicutes.Clostridia.Clostridiales.Lachnospiraceae.Lachnospira.NA.3cfa76e0da34a56a641ca33770efb588
## X.Bacteria.Firmicutes.Clostridia.Clostridiales.Lachnospiraceae.Coprococcus.NA.c9ea71f39bda8752713c8e90dff2b875                                                           X.Bacteria.Firmicutes.Clostridia.Clostridiales.Lachnospiraceae.Coprococcus.NA.c9ea71f39bda8752713c8e90dff2b875
## X.Bacteria.Firmicutes.Clostridia.Clostridiales.Lachnospiraceae.Coprococcus.NA.00979c6b417b7978c1c656e263cdd054                                                           X.Bacteria.Firmicutes.Clostridia.Clostridiales.Lachnospiraceae.Coprococcus.NA.00979c6b417b7978c1c656e263cdd054
## X.Bacteria.Firmicutes.Clostridia.Clostridiales.NA.NA.NA.504b5f0d2545c712940d4b9772a89711                                                                                                       X.Bacteria.Firmicutes.Clostridia.Clostridiales.NA.NA.NA.504b5f0d2545c712940d4b9772a89711
## X.Bacteria.Firmicutes.Clostridia.Clostridiales.Lachnospiraceae.NA.NA.fcd2073fc9628403d2dc4aa2b66b64a5                                                                             X.Bacteria.Firmicutes.Clostridia.Clostridiales.Lachnospiraceae.NA.NA.fcd2073fc9628403d2dc4aa2b66b64a5
## X.Bacteria.Firmicutes.Clostridia.Clostridiales.Lachnospiraceae.NA.NA.fda53e1a26f7489a683cfe01228fca8f                                                                             X.Bacteria.Firmicutes.Clostridia.Clostridiales.Lachnospiraceae.NA.NA.fda53e1a26f7489a683cfe01228fca8f
## X.Bacteria.Firmicutes.Clostridia.Clostridiales.Lachnospiraceae.NA.NA.470dd02996e31255357eeeaa3672b469                                                                             X.Bacteria.Firmicutes.Clostridia.Clostridiales.Lachnospiraceae.NA.NA.470dd02996e31255357eeeaa3672b469
## X.Bacteria.Firmicutes.Clostridia.Clostridiales.Lachnospiraceae.NA.NA.fb798da6858b8b3f34077164a4ec9315                                                                             X.Bacteria.Firmicutes.Clostridia.Clostridiales.Lachnospiraceae.NA.NA.fb798da6858b8b3f34077164a4ec9315
## X.Bacteria.Firmicutes.Clostridia.Clostridiales.Lachnospiraceae.NA.NA.f54512749b97be9497134dc28c0af837                                                                             X.Bacteria.Firmicutes.Clostridia.Clostridiales.Lachnospiraceae.NA.NA.f54512749b97be9497134dc28c0af837
## X.Bacteria.Firmicutes.Clostridia.Clostridiales.Lachnospiraceae.NA.NA.51dd453b71a4aba20d9f349601ea5203                                                                             X.Bacteria.Firmicutes.Clostridia.Clostridiales.Lachnospiraceae.NA.NA.51dd453b71a4aba20d9f349601ea5203
## X.Bacteria.Firmicutes.Clostridia.Clostridiales.Lachnospiraceae.NA.NA.dffa04ca171f459056b9466d7acfe601                                                                             X.Bacteria.Firmicutes.Clostridia.Clostridiales.Lachnospiraceae.NA.NA.dffa04ca171f459056b9466d7acfe601
## X.Bacteria.Firmicutes.Clostridia.Clostridiales.Lachnospiraceae.NA.NA.b20c095fd654b84cebdbfe4faa0a1409                                                                             X.Bacteria.Firmicutes.Clostridia.Clostridiales.Lachnospiraceae.NA.NA.b20c095fd654b84cebdbfe4faa0a1409
## X.Bacteria.Firmicutes.Clostridia.Clostridiales.Lachnospiraceae.NA.NA.d5c7d97e6f4f5789d574d321dcca0992                                                                             X.Bacteria.Firmicutes.Clostridia.Clostridiales.Lachnospiraceae.NA.NA.d5c7d97e6f4f5789d574d321dcca0992
## X.Bacteria.Firmicutes.Clostridia.Clostridiales.Lachnospiraceae..Ruminococcus..NA.00565f9981f176f2eae21aff895a72ff                                                     X.Bacteria.Firmicutes.Clostridia.Clostridiales.Lachnospiraceae..Ruminococcus..NA.00565f9981f176f2eae21aff895a72ff
## X.Bacteria.Firmicutes.Clostridia.Clostridiales.Lachnospiraceae..Ruminococcus..NA.6a6fcf8f9b8bb1ab9e5f8456ee7fb109                                                     X.Bacteria.Firmicutes.Clostridia.Clostridiales.Lachnospiraceae..Ruminococcus..NA.6a6fcf8f9b8bb1ab9e5f8456ee7fb109
## X.Bacteria.Firmicutes.Clostridia.Clostridiales.Lachnospiraceae..Ruminococcus..torques.e59405a47acbc248ce61395366159d8d                                           X.Bacteria.Firmicutes.Clostridia.Clostridiales.Lachnospiraceae..Ruminococcus..torques.e59405a47acbc248ce61395366159d8d
## X.Bacteria.Firmicutes.Clostridia.Clostridiales.Lachnospiraceae.NA.NA.694d61b8ec78349749c8b6ea59938e0b                                                                             X.Bacteria.Firmicutes.Clostridia.Clostridiales.Lachnospiraceae.NA.NA.694d61b8ec78349749c8b6ea59938e0b
## X.Bacteria.Firmicutes.Clostridia.Clostridiales.Lachnospiraceae.Blautia.obeum.ee293984c0110b2eeceb8427fdf448fb                                                             X.Bacteria.Firmicutes.Clostridia.Clostridiales.Lachnospiraceae.Blautia.obeum.ee293984c0110b2eeceb8427fdf448fb
## X.Bacteria.Firmicutes.Clostridia.Clostridiales.Lachnospiraceae.Blautia.NA.c1dc9ad5116d96b8ed863458fc0d0aec                                                                   X.Bacteria.Firmicutes.Clostridia.Clostridiales.Lachnospiraceae.Blautia.NA.c1dc9ad5116d96b8ed863458fc0d0aec
## X.Bacteria.Firmicutes.Clostridia.Clostridiales.Lachnospiraceae.Blautia.NA.e9703768a50971c05b34b102810fd761                                                                   X.Bacteria.Firmicutes.Clostridia.Clostridiales.Lachnospiraceae.Blautia.NA.e9703768a50971c05b34b102810fd761
## X.Bacteria.Firmicutes.Clostridia.Clostridiales.Lachnospiraceae.Blautia.NA.9f8668eb1c5f9d9a992dd49245db090e                                                                   X.Bacteria.Firmicutes.Clostridia.Clostridiales.Lachnospiraceae.Blautia.NA.9f8668eb1c5f9d9a992dd49245db090e
## X.Bacteria.Firmicutes.Clostridia.Clostridiales.Lachnospiraceae.Blautia.NA.7082034a5eec4c381d09cd380594242e                                                                   X.Bacteria.Firmicutes.Clostridia.Clostridiales.Lachnospiraceae.Blautia.NA.7082034a5eec4c381d09cd380594242e
## X.Bacteria.Firmicutes.Clostridia.Clostridiales.Lachnospiraceae.Blautia.NA.a0a476401d529ced24788bff2ffd4515                                                                   X.Bacteria.Firmicutes.Clostridia.Clostridiales.Lachnospiraceae.Blautia.NA.a0a476401d529ced24788bff2ffd4515
## X.Bacteria.Firmicutes.Clostridia.Clostridiales.Lachnospiraceae.Blautia.NA.dc2721103659fe9f1d3ead56a11df243                                                                   X.Bacteria.Firmicutes.Clostridia.Clostridiales.Lachnospiraceae.Blautia.NA.dc2721103659fe9f1d3ead56a11df243
## X.Bacteria.Firmicutes.Clostridia.Clostridiales.Lachnospiraceae.NA.NA.7e048b0e0c32d2f839e9f2098a4ebb35                                                                             X.Bacteria.Firmicutes.Clostridia.Clostridiales.Lachnospiraceae.NA.NA.7e048b0e0c32d2f839e9f2098a4ebb35
## X.Bacteria.Firmicutes.Clostridia.Clostridiales.Lachnospiraceae.NA.NA.a4e94137d3eea12b54dec05caedafe47                                                                             X.Bacteria.Firmicutes.Clostridia.Clostridiales.Lachnospiraceae.NA.NA.a4e94137d3eea12b54dec05caedafe47
## X.Bacteria.Firmicutes.Clostridia.Clostridiales.Lachnospiraceae.NA.NA.ebbe8017bca36f0a3b02532a2b4ed0d2                                                                             X.Bacteria.Firmicutes.Clostridia.Clostridiales.Lachnospiraceae.NA.NA.ebbe8017bca36f0a3b02532a2b4ed0d2
## X.Bacteria.Firmicutes.Clostridia.Clostridiales.Lachnospiraceae.NA.NA.eb61cae65bc6cdd2440323bbf603ba5c                                                                             X.Bacteria.Firmicutes.Clostridia.Clostridiales.Lachnospiraceae.NA.NA.eb61cae65bc6cdd2440323bbf603ba5c
## X.Bacteria.Firmicutes.Clostridia.Clostridiales.Lachnospiraceae.NA.NA.d9681bf8373e2635323f1ca0cc434ecf                                                                             X.Bacteria.Firmicutes.Clostridia.Clostridiales.Lachnospiraceae.NA.NA.d9681bf8373e2635323f1ca0cc434ecf
## X.Bacteria.Firmicutes.Clostridia.Clostridiales.Lachnospiraceae.NA.NA.8a64e8e18be5e17d8ab9d76bb40f65d4                                                                             X.Bacteria.Firmicutes.Clostridia.Clostridiales.Lachnospiraceae.NA.NA.8a64e8e18be5e17d8ab9d76bb40f65d4
## X.Bacteria.Firmicutes.Clostridia.Clostridiales.Lachnospiraceae.NA.NA.78314aca268ae4422bc651192fbc986d                                                                             X.Bacteria.Firmicutes.Clostridia.Clostridiales.Lachnospiraceae.NA.NA.78314aca268ae4422bc651192fbc986d
## X.Bacteria.Firmicutes.Clostridia.Clostridiales.Lachnospiraceae.NA.NA.c5465580c4c4cdd15a78de5a240dbd4e                                                                             X.Bacteria.Firmicutes.Clostridia.Clostridiales.Lachnospiraceae.NA.NA.c5465580c4c4cdd15a78de5a240dbd4e
## X.Bacteria.Firmicutes.Clostridia.Clostridiales.Lachnospiraceae.Dorea.formicigenerans.afd87e82de329a1ed75b98b5b606843c                                             X.Bacteria.Firmicutes.Clostridia.Clostridiales.Lachnospiraceae.Dorea.formicigenerans.afd87e82de329a1ed75b98b5b606843c
## X.Bacteria.Firmicutes.Clostridia.Clostridiales.Lachnospiraceae.Dorea.NA.a0b7d83fb64749f9a4b15b3728425b97                                                                       X.Bacteria.Firmicutes.Clostridia.Clostridiales.Lachnospiraceae.Dorea.NA.a0b7d83fb64749f9a4b15b3728425b97
## X.Bacteria.Firmicutes.Clostridia.Clostridiales.Lachnospiraceae.Dorea.NA.9d21d7d00d7e938a7e1c374a4f4bb833                                                                       X.Bacteria.Firmicutes.Clostridia.Clostridiales.Lachnospiraceae.Dorea.NA.9d21d7d00d7e938a7e1c374a4f4bb833
## X.Bacteria.Firmicutes.Clostridia.Clostridiales.Lachnospiraceae.NA.NA.752379aadaf3d2d89b4b240127b2e797                                                                             X.Bacteria.Firmicutes.Clostridia.Clostridiales.Lachnospiraceae.NA.NA.752379aadaf3d2d89b4b240127b2e797
##                                                                                                                                                    p.val
## depth                                                                                                                                       8.471511e-05
## X.Bacteria.Bacteroidetes.Bacteroidia.Bacteroidales..Barnesiellaceae..NA.NA.5b0c52a1507deba61b41b7f9d8187386                                 1.045770e-02
## X.Bacteria.Bacteroidetes.Bacteroidia.Bacteroidales..Barnesiellaceae..NA.NA.9d0579ce6a317ca0e626a5c68981c725                                 7.377403e-03
## X.Bacteria.Bacteroidetes.Bacteroidia.Bacteroidales..Barnesiellaceae..NA.NA.e6cd2fee507eb45ff5f3c0a4191f3876                                 1.918059e-02
## X.Bacteria.Bacteroidetes.Bacteroidia.Bacteroidales..Barnesiellaceae..NA.NA.158782cefa6e28406a2a0aeb75220f0a                                 1.537900e-03
## X.Bacteria.Bacteroidetes.Bacteroidia.Bacteroidales.Porphyromonadaceae.Parabacteroides.NA.0d8b9177d01f328c2f5d5efa4c2acbc5                   2.548294e-05
## X.Bacteria.Bacteroidetes.Bacteroidia.Bacteroidales.Porphyromonadaceae.Parabacteroides.NA.e17d964cd0fd8c2ddbd1dacfa8b536e3                   6.253640e-04
## X.Bacteria.Bacteroidetes.Bacteroidia.Bacteroidales.Porphyromonadaceae.Parabacteroides.distasonis.2cdb259b754c2db622ed9fb5a6517a37           2.325303e-04
## X.Bacteria.Bacteroidetes.Bacteroidia.Bacteroidales.Porphyromonadaceae.Parabacteroides.distasonis.f4f297232da0f8d7dae7f9c432501e22           9.958980e-03
## X.Bacteria.Bacteroidetes.Bacteroidia.Bacteroidales.Porphyromonadaceae.Parabacteroides.distasonis.4844896c9c40eaca2fa0291163b9ca52           1.365948e-04
## X.Bacteria.Bacteroidetes.Bacteroidia.Bacteroidales.Bacteroidaceae.Bacteroides.ovatus.2c2018d4ecccfa3cb27b99a04c9222b1                       3.958528e-06
## X.Bacteria.Bacteroidetes.Bacteroidia.Bacteroidales.Bacteroidaceae.Bacteroides.ovatus.03af966ff07ddef2b87da992b85b600b                       4.074536e-10
## X.Bacteria.Bacteroidetes.Bacteroidia.Bacteroidales.Bacteroidaceae.Bacteroides.NA.51e441cbdcc80da0656e82293ae160b5                           1.448789e-07
## X.Bacteria.Bacteroidetes.Bacteroidia.Bacteroidales.Bacteroidaceae.Bacteroides.fragilis.9496d87b94d90dff068f0716603930bd                     4.331756e-05
## X.Bacteria.Bacteroidetes.Bacteroidia.Bacteroidales.Bacteroidaceae.Bacteroides.fragilis.35ffdd51464e2c68179717e5334a1d7e                     1.263215e-04
## X.Bacteria.Bacteroidetes.Bacteroidia.Bacteroidales.Bacteroidaceae.Bacteroides.caccae.ec4075339e16f5cd45fd5a7955596899                       3.304740e-07
## X.Bacteria.Bacteroidetes.Bacteroidia.Bacteroidales.Bacteroidaceae.Bacteroides.NA.25d727166a36df8d2f6a915a945bf5ac                           2.694191e-04
## X.Bacteria.Bacteroidetes.Bacteroidia.Bacteroidales.Bacteroidaceae.Bacteroides.NA.3b872d4cecb7a9437ce9e5add011b471                           1.537900e-03
## X.Bacteria.Bacteroidetes.Bacteroidia.Bacteroidales.Bacteroidaceae.Bacteroides.eggerthii.8ad4a56a5f526df40d97b371ba0ef33e                    3.594936e-04
## X.Bacteria.Bacteroidetes.Bacteroidia.Bacteroidales.Bacteroidaceae.Bacteroides.eggerthii.30dd9fd45122f82c12b166a042d1eaf9                    1.056596e-03
## X.Bacteria.Bacteroidetes.Bacteroidia.Bacteroidales.Bacteroidaceae.Bacteroides.uniformis.63b26504f32377cd78d6068bffb86b9a                    6.010581e-06
## X.Bacteria.Bacteroidetes.Bacteroidia.Bacteroidales.Bacteroidaceae.Bacteroides.uniformis.574ab9c17692ffd001643c930f6895f5                    9.910612e-04
## X.Bacteria.Bacteroidetes.Bacteroidia.Bacteroidales.Prevotellaceae.Prevotella.melaninogenica.d0b698c7298bf04110a6d2f220879bfb                2.205942e-03
## X.Bacteria.Bacteroidetes.Bacteroidia.Bacteroidales..Paraprevotellaceae..NA.NA.85fddfaca6687f22a2b1c5ed45a6041c                              2.694191e-04
## X.Bacteria.Bacteroidetes.Bacteroidia.Bacteroidales..Paraprevotellaceae..Paraprevotella.NA.37dac50f454a3cf754e83790eee77108                  3.347338e-04
## X.Bacteria.Bacteroidetes.Bacteroidia.Bacteroidales.Bacteroidaceae.Bacteroides.NA.b65eb19257f7a2bedb5a1c4b42aeb396                           1.078515e-04
## X.Bacteria.Bacteroidetes.Bacteroidia.Bacteroidales.Bacteroidaceae.Bacteroides.NA.668fdb718997fc1589c7817655d4bb5f                           9.604264e-10
## X.Bacteria.Bacteroidetes.Bacteroidia.Bacteroidales..Odoribacteraceae..Butyricimonas.NA.0bb0e1802451aace7137a4d598f775f5                     2.079277e-03
## X.Bacteria.Bacteroidetes.Bacteroidia.Bacteroidales..Odoribacteraceae..Odoribacter.NA.3ea67969a01ec419ec1d4784d379ad1d                       2.480008e-03
## X.Bacteria.Bacteroidetes.Bacteroidia.Bacteroidales.Rikenellaceae.NA.NA.76d4dadc006ff1d530d38dd3cfac300c                                     6.618496e-05
## X.Bacteria.Bacteroidetes.Bacteroidia.Bacteroidales.Rikenellaceae.NA.NA.e47a63686b619f67783f9b9aa52b86bf                                     5.820766e-11
## X.Bacteria.Bacteroidetes.Bacteroidia.Bacteroidales.Rikenellaceae.NA.NA.619f64f2bf103286f4f70bfd89500ed4                                     1.448789e-07
## X.Bacteria.Bacteroidetes.Bacteroidia.Bacteroidales.Rikenellaceae.NA.NA.7534fb513a4b404419edc4e91920af3f                                     1.460966e-05
## X.Bacteria.Bacteroidetes.Bacteroidia.Bacteroidales.Rikenellaceae.NA.NA.a231814083060142816974207844eff2                                     4.886941e-06
## X.Bacteria.Proteobacteria.Gammaproteobacteria.Enterobacteriales.Enterobacteriaceae.NA.NA.bfe54af4c9180d37a0d76f6dafe79a5a                   1.559965e-08
## X.Bacteria.Proteobacteria.Gammaproteobacteria.Enterobacteriales.Enterobacteriaceae.Escherichia.coli.1b158b8b2922d4fcad5d9cea607cbb7d        1.153676e-06
## X.Bacteria.Proteobacteria.Gammaproteobacteria.Enterobacteriales.Enterobacteriaceae.Morganella.morganii.78056058faedd75d706633c5f55a975f     1.267446e-02
## X.Bacteria.Proteobacteria.Gammaproteobacteria.Xanthomonadales.Xanthomonadaceae.Stenotrophomonas.NA.891019e319f5d8d088ed4f9379fd63ef         3.492007e-03
## X.Bacteria.Proteobacteria.Deltaproteobacteria.Desulfovibrionales.Desulfovibrionaceae.Bilophila.NA.ece50a62168f85fc61385d8adb4c6494          4.088388e-02
## X.Bacteria.Proteobacteria.Deltaproteobacteria.Desulfovibrionales.Desulfovibrionaceae.Desulfovibrio.NA.dfd770d48651635c39bdafe13f62e75a      2.783855e-03
## X.Bacteria.Actinobacteria.Coriobacteriia.Coriobacteriales.Coriobacteriaceae.Collinsella.aerofaciens.dfba68ef0fd0e712608eb2a0078013a7        6.912160e-08
## X.Bacteria.Actinobacteria.Coriobacteriia.Coriobacteriales.Coriobacteriaceae.Slackia.NA.c9a59a2ded38b324d6d58202a4137f9b                     9.291920e-04
## X.Bacteria.Actinobacteria.Coriobacteriia.Coriobacteriales.Coriobacteriaceae.Eggerthella.lenta.6bb6aad559c5eaeb22b2eef7874ef038              1.152018e-02
## X.Bacteria.Actinobacteria.Coriobacteriia.Coriobacteriales.Coriobacteriaceae.NA.NA.23368c2d745ed62db9ac0a19c9dcec5d                          1.537900e-03
## X.Bacteria.Actinobacteria.Actinobacteria.Bifidobacteriales.Bifidobacteriaceae.Bifidobacterium.NA.69e611251f4d8582e312afa5737f033e           2.783855e-03
## X.Bacteria.Actinobacteria.Actinobacteria.Bifidobacteriales.Bifidobacteriaceae.Bifidobacterium.adolescentis.554c761996ebab999befda1b695fd81d 9.480974e-03
## X.Bacteria.Actinobacteria.Actinobacteria.Actinomycetales.Actinomycetaceae.Actinomyces.NA.608e6548b1b4cbb6176c8fce090991a5                   1.459187e-02
## X.Bacteria.Actinobacteria.Actinobacteria.Actinomycetales.Actinomycetaceae.Actinomyces.NA.a78fa572a60226d8150bfb0fc0fad652                   3.301490e-03
## X.Bacteria.Verrucomicrobia.Verrucomicrobiae.Verrucomicrobiales.Verrucomicrobiaceae.Akkermansia.muciniphila.8f98fb8693ed59c21399d83ce2d10724 1.459187e-02
## X.Bacteria.Firmicutes.Bacilli.Lactobacillales.Lactobacillaceae.Lactobacillus.zeae.5a4eda6fcdd6ab834dba0111fbe55d95                          2.123261e-05
## X.Bacteria.Firmicutes.Bacilli.Lactobacillales.Lactobacillaceae.Lactobacillus.zeae.45a68a9eee3cf83e27f4ea309d57ffc3                          3.115251e-04
## X.Bacteria.Firmicutes.Bacilli.Lactobacillales.Lactobacillaceae.Pediococcus.NA.3c133555aa2bbda4902d66bdceb138a3                              9.958980e-03
## X.Bacteria.Firmicutes.Bacilli.Lactobacillales.Lactobacillaceae.Lactobacillus.salivarius.ff2bd29ff42e4dc25a31714e0b6c2dca                    1.045770e-02
## X.Bacteria.Firmicutes.Bacilli.Lactobacillales.Streptococcaceae.Streptococcus.NA.bd2ebc70501f7d867c204f94c4e483da                            3.775702e-02
## X.Bacteria.Firmicutes.Bacilli.Lactobacillales.Streptococcaceae.Streptococcus.NA.73bf8d1a5983e34a0cb84e3cae127815                            2.694191e-04
## X.Bacteria.Firmicutes.Bacilli.Lactobacillales.Carnobacteriaceae.Granulicatella.NA.24a60c6448e70d9198ad6ba93520958c                          4.354625e-03
## X.Bacteria.Firmicutes.Bacilli.Lactobacillales.Aerococcaceae.Abiotrophia.NA.8114b1d0274e9e4bb6c91f6af1b8fac8                                 6.657466e-03
## X.Bacteria.Firmicutes.Bacilli.Gemellales.Gemellaceae.NA.NA.cda4e6f933bb3108ea3e92f9db411c00                                                 5.692560e-03
## X.Bacteria.Firmicutes.Clostridia.Clostridiales.Veillonellaceae.Phascolarctobacterium.NA.e5413f67faa6b8c0c3e63e48836c0b42                    3.343982e-02
## X.Bacteria.Firmicutes.Clostridia.Clostridiales.Veillonellaceae.Megasphaera.NA.2f93e58b78f2842e83abd5fde37ad276                              7.365830e-06
## X.Bacteria.Firmicutes.Clostridia.Clostridiales.Veillonellaceae.Dialister.NA.520c77820886daeb8cf0d6497cfb1344                                1.460966e-05
## X.Bacteria.Firmicutes.Clostridia.Clostridiales.Veillonellaceae.Veillonella.dispar.5608c3e6c9de9ceb79610e7786bd0ac4                          1.460966e-05
## X.Bacteria.Firmicutes.Clostridia.Clostridiales.Veillonellaceae.Veillonella.dispar.5b4f8b625d8fbb1268863be7dbc4db5d                          9.022970e-03
## X.Bacteria.Firmicutes.Clostridia.Clostridiales..Mogibacteriaceae..NA.NA.97b46cdc630fb1c3097f961bb4f627e5                                    1.199425e-03
## X.Bacteria.Firmicutes.Clostridia.Clostridiales..Mogibacteriaceae..NA.NA.4119ee9eb78e6baa92065d886a5e24ac                                    7.807108e-05
## X.Bacteria.Firmicutes.Clostridia.Clostridiales..Mogibacteriaceae..NA.NA.4260927e3b266486b5d210135f0ef4f6                                    2.628042e-03
## X.Bacteria.Firmicutes.Erysipelotrichi.Erysipelotrichales.Erysipelotrichaceae.NA.NA.216aca81784431f49e9567d23f7391e8                         6.687742e-04
## X.Bacteria.Firmicutes.Erysipelotrichi.Erysipelotrichales.Erysipelotrichaceae.NA.NA.f95cab37fba4160de15015f4d520839f                         1.669687e-07
## X.Bacteria.Firmicutes.Erysipelotrichi.Erysipelotrichales.Erysipelotrichaceae.NA.NA.0c3887592dd60a361d43c78e501ba495                         2.005368e-02
## X.Bacteria.Firmicutes.Erysipelotrichi.Erysipelotrichales.Erysipelotrichaceae.Holdemania.NA.84e9e2afcdae1240b3ce3267067e1879                 6.618496e-05
## X.Bacteria.Firmicutes.Erysipelotrichi.Erysipelotrichales.Erysipelotrichaceae.Holdemania.NA.bc53814d35b2f862562a1e865e923b33                 6.283226e-07
## X.Bacteria.Firmicutes.Clostridia.Clostridiales.NA.NA.NA.cc4ff6e54743c750e2efa569086a38cf                                                    4.759390e-04
## X.Bacteria.Firmicutes.Clostridia.Clostridiales.NA.NA.NA.fd2130b6a8a1db45c0f5381012b2e5e7                                                    1.056596e-03
## X.Bacteria.Firmicutes.Clostridia.Clostridiales.NA.NA.NA.df15aa00cffdc5235078831e54deb6db                                                    3.115251e-04
## X.Bacteria.Firmicutes.Clostridia.Clostridiales.NA.NA.NA.6b40524e525db04087e414f058bc1a5e                                                    1.125985e-03
## X.Bacteria.Firmicutes.Clostridia.Clostridiales.NA.NA.NA.c838eb5b3b3d9fd0dfecdf75547c3c63                                                    9.910612e-04
## X.Bacteria.Firmicutes.Clostridia.Clostridiales.NA.NA.NA.d917386c5f307a4f652e389dadd7cef5                                                    3.115251e-04
## X.Bacteria.Firmicutes.Clostridia.Clostridiales.Clostridiaceae.Clostridium.NA.5c82dc47435864e490625ae01151570e                               6.024493e-09
## X.Bacteria.Firmicutes.Clostridia.Clostridiales.Ruminococcaceae.NA.NA.0437c9910becc153a3d7838fd8eaa64b                                       2.339416e-03
## X.Bacteria.Firmicutes.Clostridia.Clostridiales.NA.NA.NA.dff390a58455e2599a43c5e400a35518                                                    9.910612e-04
## X.Bacteria.Firmicutes.Clostridia.Clostridiales.Ruminococcaceae.NA.NA.26f6853f46b06854fe5418317a261fb8                                       4.440158e-04
## X.Bacteria.Firmicutes.Clostridia.Clostridiales.Ruminococcaceae.NA.NA.6013b9e15b35c4e724eb0c323c05f9d3                                       2.205942e-03
## X.Bacteria.Firmicutes.Clostridia.Clostridiales.Ruminococcaceae.Oscillospira.NA.a7283edda8770d12b4e25d4dcce112c4                             1.277118e-03
## X.Bacteria.Firmicutes.Clostridia.Clostridiales.Ruminococcaceae.Oscillospira.NA.e13823e500387439450b3826ea191948                             9.186950e-05
## X.Bacteria.Firmicutes.Clostridia.Clostridiales.Ruminococcaceae.Oscillospira.NA.cb63ff338fde1171b1e985e8211b1929                             2.205942e-03
## X.Bacteria.Firmicutes.Clostridia.Clostridiales.Ruminococcaceae.Oscillospira.NA.d243b9b1c0b782aedbaa68fa5697193c                             1.959117e-03
## X.Bacteria.Firmicutes.Clostridia.Clostridiales.Ruminococcaceae.Oscillospira.NA.680d83cb233cffcc6405e08c46982042                             6.912160e-08
## X.Bacteria.Firmicutes.Clostridia.Clostridiales.Ruminococcaceae.Oscillospira.NA.7c93315259ad14510b6e9cae0670a048                             8.708080e-04
## X.Bacteria.Firmicutes.Clostridia.Clostridiales.Ruminococcaceae.NA.NA.e803ff46adaa0fa149ef151b082378a0                                       5.099171e-04
## X.Bacteria.Firmicutes.Clostridia.Clostridiales.Ruminococcaceae.NA.NA.63027647b049a274c881df23a4611b35                                       2.123261e-05
## X.Bacteria.Firmicutes.Clostridia.Clostridiales.Ruminococcaceae.NA.NA.c4995645e0c1545b0e1620144d03772b                                       2.561137e-09
## X.Bacteria.Firmicutes.Clostridia.Clostridiales.Ruminococcaceae.Oscillospira.NA.f2c958dfcac880c2f53a47e114d994d8                             4.400412e-06
## X.Bacteria.Firmicutes.Clostridia.Clostridiales.Ruminococcaceae.Oscillospira.NA.9e1d23e474a9f1e4e10387a23f601dde                             1.297769e-06
## X.Bacteria.Firmicutes.Clostridia.Clostridiales.Ruminococcaceae.Oscillospira.NA.4ef2b8e0ed22c2a986f53572f9e9405b                             2.788709e-05
## X.Bacteria.Firmicutes.Clostridia.Clostridiales.Ruminococcaceae.Oscillospira.NA.04a802674683d317a8c7484ce2407ce0                             8.043717e-07
## X.Bacteria.Firmicutes.Clostridia.Clostridiales.Ruminococcaceae.Oscillospira.NA.3e2bfdd1acae4723c96e344f91b262d8                             4.354625e-03
## X.Bacteria.Firmicutes.Clostridia.Clostridiales.Ruminococcaceae.NA.NA.2673143ffb9d8ff3ad82fa40e325973c                                       2.480008e-03
## X.Bacteria.Firmicutes.Clostridia.Clostridiales.Ruminococcaceae.Ruminococcus.bromii.fc02af89d869043b296114729009a5b0                         2.205488e-07
## X.Bacteria.Firmicutes.Clostridia.Clostridiales.Ruminococcaceae.Ruminococcus.NA.98ff1f2b1008b5e0971997cd5070fc03                             9.604264e-10
## X.Bacteria.Firmicutes.Clostridia.Clostridiales.Ruminococcaceae.Ruminococcus.NA.16ca411ff5a7131287f6deb7fc13b260                             1.365948e-04
## X.Bacteria.Firmicutes.Clostridia.Clostridiales.Ruminococcaceae.Ruminococcus.NA.153c48635771b1fdd766fc9beee8d0ab                             4.252561e-02
## X.Bacteria.Firmicutes.Clostridia.Clostridiales.Ruminococcaceae.Ruminococcus.NA.b61b28e1ab92f5cc8effcdde01c2ca90                             1.365948e-04
## X.Bacteria.Firmicutes.Clostridia.Clostridiales.Ruminococcaceae.Ruminococcus.NA.d048172b7b49bf1669197f10389312cd                             1.721307e-04
[truncated: 160,198 more chars]
