# Supplementary figures and images for: Changes in IgA-targeted microbiota following fecal transplantation for recurrent Clostridioides difficile infection
Source: Gut Microbes. 2020 Dec 31;13(1):1862027. doi: 10.1080/19490976.2020.1862027 (PMC7781654; doi:10.1080/19490976.2020.1862027)

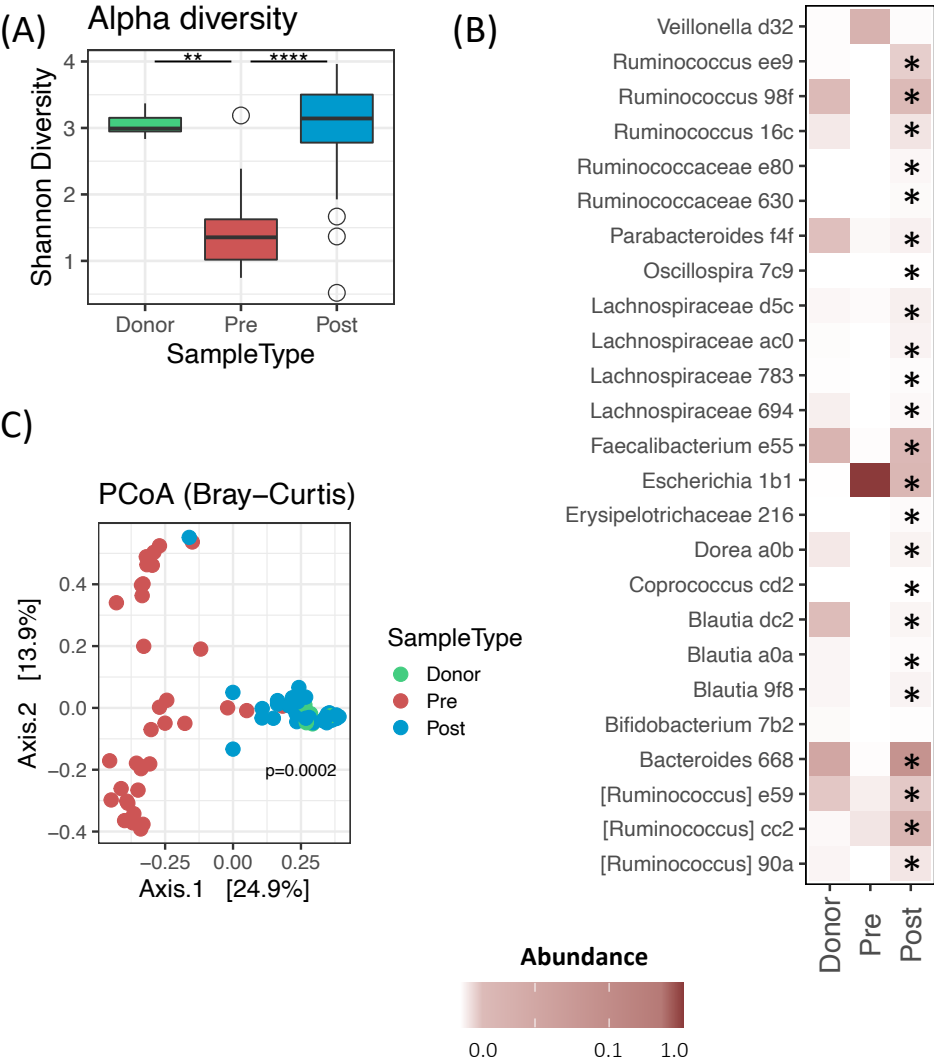

Fig S1.

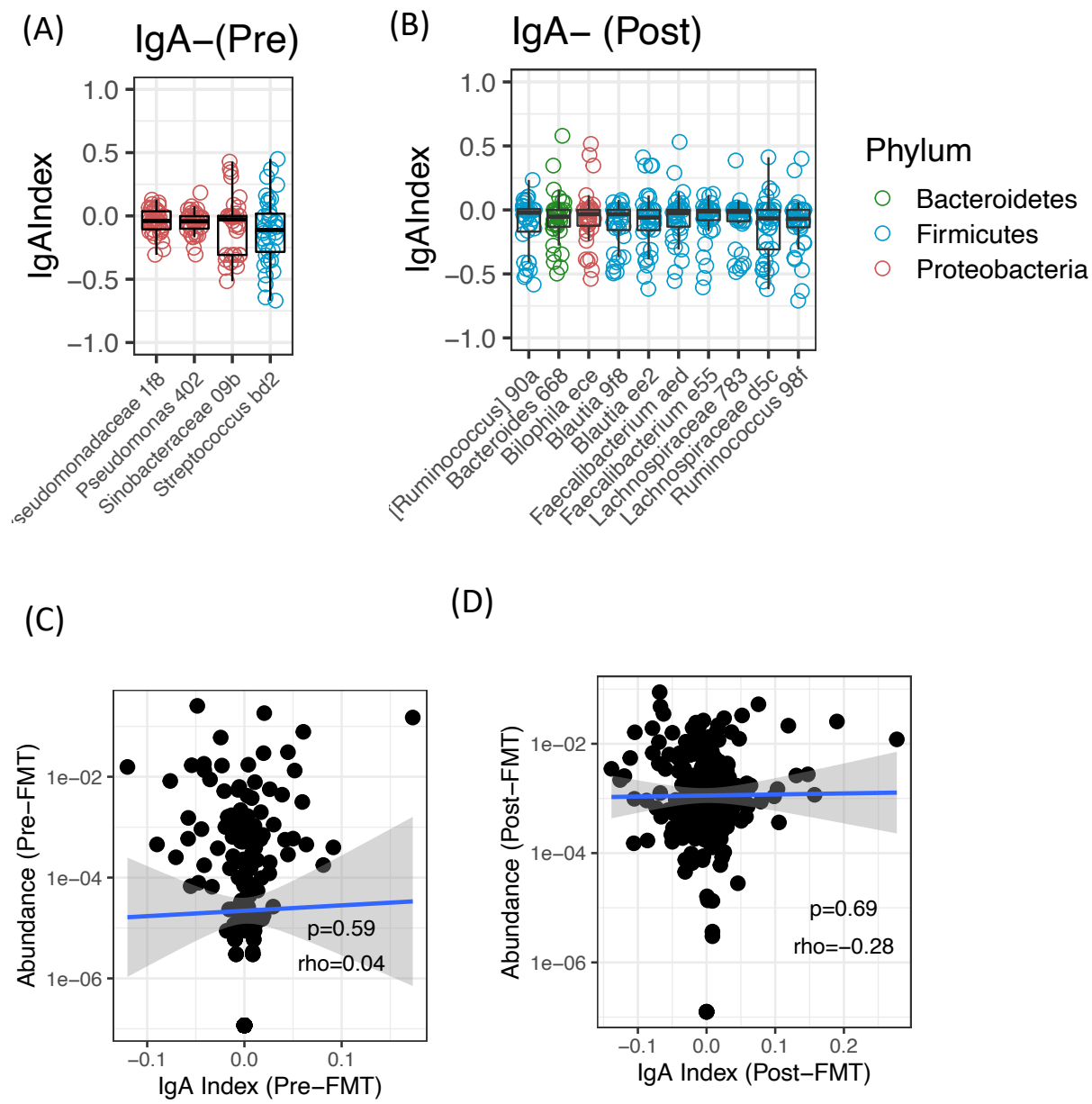

Fig S2.

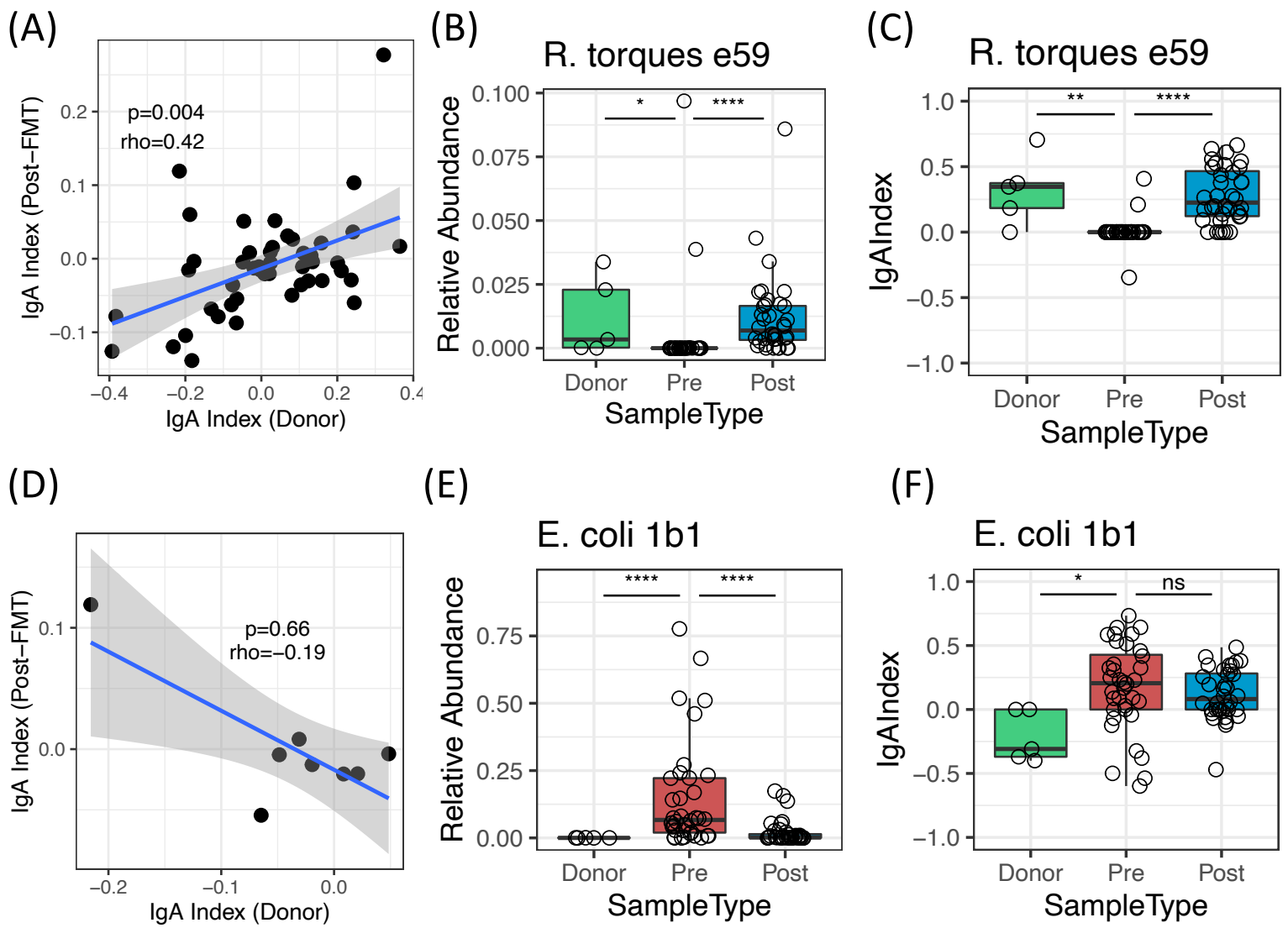

Fig S3.

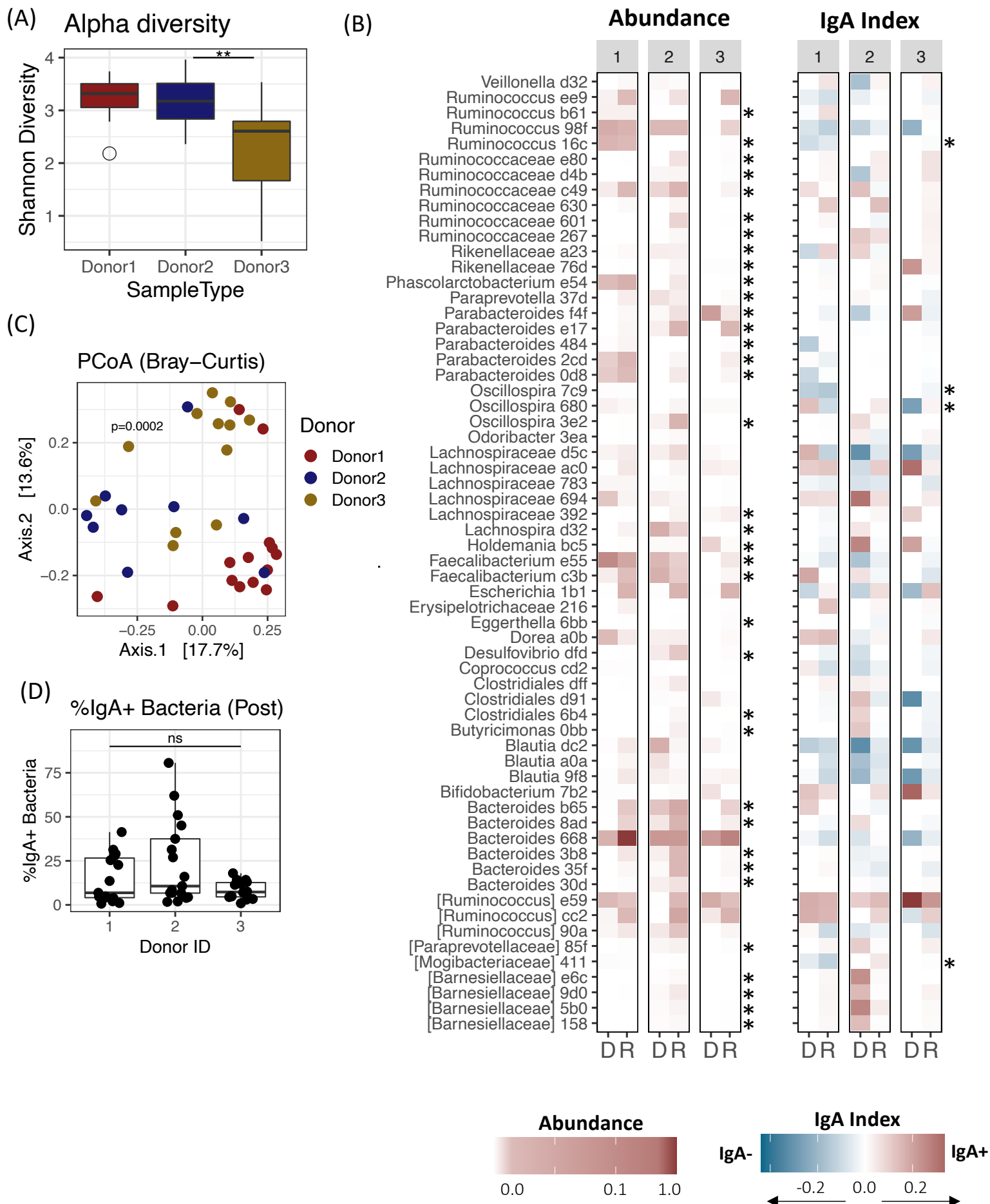

Fig S4.

Supplement: Supplemental Material [file KGMI_A_1862027_SM4814.zip › supplementary/Supplementary.pdf]
